# Prognostic Value of Plaque Volume in Patients With First Diagnosis of Coronary Artery Disease: A Substudy of the PROMISE Randomized Clinical Trial

**DOI:** 10.1001/jamacardio.2025.5520

**Published:** 2026-02-11

**Authors:** Júlia Karády, Thomas Mayrhofer, Jan M. Brendel, Márton Kolossváry, Marcel Langenbach, Isabel Langenbach, Vinit Baliyan, Audra K. Sturniolo, Neha Pagidipati, Michael T. Lu, Maros Ferencik, Svati Shah, Pamela S. Douglas, Borek Foldyna

**Affiliations:** 1 Cardiovascular Imaging Research Center, Radiology Department, Massachusetts General Hospital, Boston; 2 Department of Pediatrics, Boston Children’s Hospital, Boston, Massachusetts; 3 Heart and Vascular Center, Semmelweis University, Budapest, Hungary; 4 Center for Preventive Medicine and Digital Health, Heidelberg University, Mannheim, Germany; 5 Gottsegen National Cardiovascular Center, Budapest, Hungary; 6 Physiological Controls Research Center, University Research and Innovation Center, Óbuda University, Budapest, Hungary; 7 Duke Clinical Research Institute, Duke University School of Medicine, Durham, North Carolina; 8 Knight Cardiovascular Institute, Oregon Health & Science University, Portland

## Abstract

**Importance:**

Despite the increasing use of coronary computed tomographic angiography (CCTA) in patients with known or suspected coronary artery disease (CAD), comparatively little is known about its predictive value for adverse events or clinical applicability of volumetric plaque analysis.

**Objective:**

To assess the incremental prognostic value of quantitative CAD measures in symptomatic outpatients without known CAD.

**Design, Setting, and Participants:**

This post hoc analysis involved a prospective randomized clinical trial conducted across 193 clinical sites in North America. Participants were symptomatic outpatients without known CAD who were randomized to receive CCTA. The trial was conducted from July 27, 2010, to October 31, 2014, and the data analyzed for this report from January 2021 to July 2024.

**Exposures:**

Core laboratory-based quantitative plaque measures, including total plaque volume (TPV), calcified (CPV) and noncalcified (NCPV) plaque volume, low-attenuation plaque volume (LAPV), total plaque burden (TPB), and noncalcified plaque burden (NCPB), normalized with vessel volume.

**Main Outcomes and Measures:**

The primary outcome was major adverse cardiovascular events MACE (composite of death, nonfatal myocardial infarction, or hospitalization for unstable angina). Optimal predictive cut points for plaque measures were identified using Euclidean distance methods and tested in multivariable Cox regression models.

**Results:**

Among 4267 patients, the mean (SD) age was 60.4 (8.2) years; 2199 patients (51.5%) were female and 2068 (48.5%) were male. The median (IQR) TPV was 39.8 mm^3^ (0-167) mm^3^. Patients with TPV at the median or higher were older (mean [SD] age, 62.1 [8.4] vs 58.7 [7.5] years for those with lower than median TPV), more likely to be male (1286/2133 [60.3%] vs 782/2134 [36.6%], respectively), and had higher median (IQR) atherosclerotic cardiovascular disease risk scores (14.4 [8.8-24.0] vs 7.9 [4.5-13.4], respectively). TPB showed similar demographic associations. Both TPB and NCPB independently predicted MACE after adjusting for clinical risk factors, statin use, and qualitative CCTA findings (TPB: adjusted HR [aHR], 1.18; 95% CI, 1.05-1.34; *P* = .006; NCPB: aHR, 1.20; 95% CI, 1.05-1.37; *P* = .007). Optimal cutoffs of TPV 87 mm^3^ or greater, TPB 35% or greater, and NCPB 20% or greater were each associated with nearly a 2-fold increase in MACE risk (TPV: aHR, 2.07; 95% CI, 1.24-3.49; TPB: aHR, 1.96; 95% CI, 1.21-3.17; and NCPB: aHR, 1.77; 95% CI, 1.12-2.82).

**Conclusions and Relevance:**

In symptomatic patients without known CAD, coronary plaque volumes and burdens are low but are related to CAD risk factors and independently predictive of MACE. The clinical utility of quantitative CCTA-based cardiovascular risk estimation in early CAD requires prospective evaluation.

**Trial Registration:**

ClinicalTrials.gov Identifier: NCT01174550

## Key Points

**Question:**

Does quantitative plaque assessment improve prognostication of major adverse cardiovascular events (MACE) in patients with unknown history of coronary artery disease?

**Findings:**

In this substudy of the randomized clinical trial PROMISE, among 4267 symptomatic patients, higher total plaque volume (≥87 mm), total plaque burden (≥35%), and noncalcified plaque burden (≥20%) were associated with an increased risk of MACE, independent of atherosclerotic cardiovascular disease risk, statin use, 50% or more stenosis, coronary artery calcium score, and high-risk plaque.

**Meaning:**

Quantitative plaque measures on coronary computed tomographic angiography (CCTA) improve risk prediction for MACE and may enhance early cardiovascular risk assessment beyond clinical risk and routinely assessed CCTA metrics.

## Teaser

This secondary analysis of a randomized clinical trial examines plaque measures from coronary computed tomographic angiography to assess the incremental prognostic value of quantitative coronary artery disease measures among symptomatic patients.

## Introduction

Coronary computed tomography angiography (CCTA) is a first-line diagnostic tool in patients presenting with suspected coronary artery disease (CAD),^1,2^ uniquely providing direct visualization, characterization, and assessment of atherosclerosis. Prognostic information can be gleaned from coronary artery calcium (CAC) scores and CCTA plaque features, including obstructive stenosis 50% or more, high-risk plaque (HRP) features, and semiquantitative plaque burden estimates (ie, CT-adopted Leaman score),^3,4,5,6^ driving clinical adoption.

Advancements in CCTA image analysis allow for precise quantification of total plaque volume (TPV) and its subcomponents, including calcified (CPV), noncalcified (NCPV), and low attenuation plaque volume (LAPV). In patients with known or advanced disease, these more detailed measures of atherosclerotic plaque characteristics were shown to improve prediction of major adverse cardiovascular events (MACE) beyond traditional cardiovascular risk factors and qualitative CCTA findings.^7,8,9,10^ In fact, the US Food and Drug Administration (FDA) is currently considering validating plaque volume as a prognostic biomarker.^11^

However, there has been limited research on the value of quantitative plaque metrics among patients at lower risk for atherosclerotic cardiovascular disease (ASCVD) or those with early disease for whom risk assessment is an essential guide to the initiation and escalation of preventive therapies.^7,12^ In agreement with this knowledge gap, the recent Lancet Commission on CAD has called for new methods of diagnosis and risk prediction in the early stages of atherosclerosis.^13^

To address the need to establish the predictive value and clinical applicability of quantitative plaque analysis in early CAD, we leveraged the landmark Prospective Multicenter Imaging Study for Evaluation of Chest Pain (PROMISE) CCTA cohort. We assessed associations between quantitative plaque measures and incident adverse events, determining the added prognostic value compared with clinical risk factors and qualitative CCTA findings. Further, we established optimal prognostic cut points within this cohort that may be useful in guiding clinical care decision-making if more widely validated in persons with a first diagnosis of CAD.

## Methods

### Patient Population

The PROMISE trial was a pragmatic comparative effectiveness trial across 193 clinical sites in North America (ClinicalTrials.gov NCT01174550; see trial protocol in Supplement 1).^14,15^ A total of 10 003 outpatients without known CAD and requiring noninvasive cardiovascular testing for stable symptoms were randomized 1:1 to receive CCTA or functional testing. The current analysis includes CCTA patients, excluding those who did not receive a contrast CT or those for whom CCTA datasets were unavailable or nondiagnostic (eFigure in Supplement 2). Local or central institutional review boards approved the study protocol at each coordinating center and enrolling site. All participants provided written informed consent.

### CT Image Acquisition and Analysis

CT image acquisition was performed on 64-row-or-greater CT scanners, following guidelines current at the time of enrollment.^16^ The CT datasets were transferred to a central core laboratory for analysis by readers with level 3 training, using dedicated workstations (TeraRecon; Qangio CT, Medis BV).^17,18^

#### CAC Scoring and Qualitative CCTA Plaque Analysis

CAC was assessed by using the standard Agatston method.^19^ CCTA images with diagnostic image quality were assessed for the presence of plaque on a per-segment level. Stenosis severity in segments with plaque was graded (0%, 1%-29%, 30%-49%, 50%-69%, or ≥70% stenosis)^20^ and the presence of HRP features was assessed, including low attenuation (<30 Hounsfield units [HU]), positive remodeling (remodeling index >1.1), and napkin ring sign (plaque with low CT attenuation central core and ring-like higher attenuation peripheral rim).^5^ CT-adapted Leaman score was determined as detailed in the eMethods in Supplement 2.^6^

#### Quantitative CCTA Plaque Analysis

The coronary tree and vessel centerlines were automatically detected and adjusted manually if needed. For each coronary plaque, proximal and distal reference points were placed to define the region of interest. Following automated coronary lumen and outer coronary artery wall detection, manual refinements were applied as needed. Total PV (mm^3^) was defined as the volume of all voxels between the luminal and outer vessel wall contours. Subcomponent PVs were determined based on predefined fixed CT attenuation thresholds (CPV ≥350 HU, NCPV <350 HU, and LAPV <30 HU).^8,21^ PVs were summed and reported at the patient level. Plaque burden (PB), expressed in % (range, 0-100), was calculated by normalizing PV with the sum of the corresponding vessel volume within the measured region of interest.^22,23^ Interobserver reproducibility for TPV was assessed blindly on 40 CCTA datasets (intraclass correlation coefficient 0.91; 95% CI, 0.81-0.96; *P* < .001).

### End Points

The primary end point was MACE, defined as the composite of all-cause death, nonfatal myocardial infarction (MI), or hospitalization for unstable angina, the original PROMISE primary end point. A sensitivity analysis included cardiovascular outcomes (nonfatal MI or cardiovascular death). Events were adjudicated by a blinded independent committee according to predetermined definitions throughout a median (IQR) follow-up of 25 (18-34) months.^14,15^

### Statistical Analysis

Continuous variables are reported as mean (SD) or median (IQR), and categorical variables as absolute and relative frequencies (%). Group comparisons used 2-sample *t* tests or Wilcoxon rank sum tests (continuous variables) and Fisher exact tests (categorical variables). Kaplan-Meier estimates were used for cumulative event rates, stratified by median PV or PB. Cox proportional hazard models assessed associations between quartiles and continuous PV and PB measures and time to MACE (or censoring). To account for differences among participants, regression models were adjusted as follows: model 1 included age, sex, race, ASCVD risk score,^24^ and statin use; model 2 was further adjusted for continuous CAC score, stenosis 50% or more, and HRP features; additional exploratory models (models 3-6) included model 1 and individual CCTA features. As a robustness check, we conducted Cox-restricted cubic spline regression analyses to examine the associations between continuous quantitative plaque measures and event risk. We performed a subgroup analysis focusing on patient with CAC score of 0. Optimal TPV, TPB, and NCPB cutoffs were identified by minimizing the Euclidean distance from the receiver operating characteristics curve to the point of perfect sensitivity and specificity. Improvements in MACE prediction were tested by comparing the C statistic and likelihood ratio between a base model (age, sex, race, ASCVD risk score, statin use, continuous CAC score, stenosis ≥50%, and HRP features) and full models including individual PV and PB cutoffs. To address potential overfitting, we conducted an internal validation based on bootstrapping. Bootstrapped C statistics were similar to apparent C statistics and are thus not shown. Because of the exploratory character of this study, inferences were guided by a 2-sided 5% false-positive error rate without adjustment for multiple comparisons. As a sensitivity analysis, we provide results of assessing the prognostic performance of low-attenuation plaque burden (LAPB) more than 4% as a threshold in the data supplement. Statistical analyses were conducted in Stata version 18.0 (StataCorp).

## Results

### Study Population

Baseline characteristics of the 4267 study participants with CCTA data are summarized in Table 1. Among 4267 patients, the mean (SD) age was 60.4 (8.2) years; 2199 patients (51.5%) were female and 2068 (48.5%) were male. The cohort was middle-aged, gender-balanced, and majority White (Asian patients: 128/4223 [3.0%]; Black: 427/4223 [10.1%]; White: 3289/4223 [78%]; self-reported racial or ethnic minority group: 952/4241 [22.5%]), and had a moderate ASCVD 10-year risk estimate (median [IQR], 11.0% [6.1%-19.1%]).

**Table 1.** Baseline Demographics Overall and Stratified by Median Total Plaque Volume

Characteristic	No./total No. (%)			*P* value
	All patients (n = 4267)	By median total plaque volume		
		<39.8 mm^3^ (n = 2134)	≥39.8 mm^3^ (n = 2133)	
**Demographic**				
Age, mean (SD), y	60.4 (8.2)	58.7 (7.5)	62.1 (8.4)	<.001
Sex				
Female	2199/4267 (51.5)	1352/2134 (63.4)	847/2133 (39.7)	<.001
Male	2068/4267 (48.5)	782/2134 (36.6)	1286/2133 (60.3)	
Race^a^				
Asian	128/4223 (3.0)	66/2115 (3.1)	62/2108 (2.9)	.79
Black	427/4223 (10.1)	266/2115 (12.6)	161/2108 (7.6)	<.001
White	3289/4223 (77.9)	1574/2115 (74.4)	1725/2108 (81.4)	<.001
Racial or ethnic minority	952/4241 (22.5)	547/2121 (26.1)	405/2120 (19.1)	<.001
Ethnicity				
Hispanic or Latino	304/4223 (7.2)	171/2115 (8.1)	133/2108 (6.3)	.03
Not Hispanic or Latino	3919/4223 (92.8)	1944/2115 (91.9)	1975/2108 (93.7)	.03
**Cardiac risk factors**				
BMI, mean (SD)^b^	30.3 (5.9)	30.4 (6.1)	30.2 (5.6)	.43
Hypertension	2716/4267 (63.7)	1278/2134 (59.7)	1438/2133 (67.4)	<.001
Diabetes	865/4267 (20.3)	350/2134 (16.4)	515/2133 (23.1)	<.001
Dyslipidemia	2862/4267 (67.1)	1379/2134 (64.6)	1483/2133 (69.5)	<.001
Smoking (ever)	2184/4266 (51.2)	954/2133 (44.7)	1230/2133 (57.7)	<.001
Family history of premature CAD	1400/4253 (32.9)	661/2128 (31.1)	739/2125 (34.8)	.01
History of depression	840/4267 (19.7)	441/2134 (20.7)	399/2133 (18.7)	.11
Participate in physical activity	2209/4258 (51.9)	1092/2129 (51.3)	1117/2129 (52.5)	.46
Peripheral artery disease	211/4266 (5.0)	89/2134 (4.2)	122/2133 (5.7)	.02
CAD equivalent	1018/4267 (23.9)	421/2134 (19.7)	597/2133 (28.0)	<.001
Sedentary lifestyle	2049/4258 (48.1)	1037/2129 (48.7)	1012/2129 (47.5)	.46
Metabolic syndrome	1558/4267 (36.5)	719/2134 (33.7)	839/2133 (39.3)	<.001
No risk factor	111/4267 (2.6)	56/2134 (2.6)	55/2133 (2.6)	>.99
Risk factor burden	2.35 (2.32-2.38)	2.17 (2.12-2.21)	2.53 (2.49-2.58)	<.001
Medication use				
Aspirin	1840/4080 (45.1)	794/2020 (39.3)	1046/2060 (50.8)	<.001
Statin	1857/4080 (45.5)	820/2020 (40.6)	1037/2060 (50.3)	<.001
β-Blocker	996/4080 (24.4)	477/2020 (23.6)	519/2060 (25.2)	.24
ACEi or ARB	1738/4080 (42.6)	777/2020 (38.5)	961/2060 (46.7)	<.001
ASCVD risk score				
ASCVD (2013), median (IQR), %	11.0 (6.1-19.1)	7.9 (4.5-13.4)	14.4 (8.8-24.0)	<.001
ASCVD categories				
<5%	795/4221 (18.8)	610/2116 (28.8)	185/2105 (8.8)	<.001
5% to <7.5%	614/4221 (14.6)	399/2116 (18.9)	215/2105 (10.2)	
7.5% to <20%	1846/4221 (43.7)	841/2116 (39.7)	1005/2105 (47.7)	
≥20%	966/4221 (22.9)	266/2116 (12.6)	700/2105 (33.3)	

Abbreviations: ACEi, angiotensin converting enzyme inhibitor; ARB, angiotensin II receptor blocker; ASCVD, atherosclerotic cardiovascular disease; BMI, body mass index; CAD, coronary artery disease.

^a^ Racial or ethnic minority group was self-reported, with the status of “minority” being defined by the patient.

^b^ Calculated as weight in kilograms divided by height in meters squared.

### Clinical Correlates of Higher Plaque Volume and Burden

Baseline characteristics stratified by median (IQR) TPV (39.8 [0.00-167] mm^3^) (Table 2) showed that patients with TPV 39.8 mm^3^ or greater were at higher cardiovascular risk: they were older (mean [SD] age, 62.1 [8.4] vs 58.7 [7.5] years; *P* < .001), more likely to be men (782/2134 [36.6%] vs 1286/2133 [60.3%]; *P* < .001), and, among other differences, had higher median (IQR) ASCVD risk scores (14.4% [8.8%-24.0%] vs 7.9% [4.5%-13.4%]; *P* < .001). Similar patterns were observed when stratifying by the median TPB of 27.0% (eTable 1 in Supplement 2).

**Table 2.** Univariable and Multivariable Assessment of the Association Between Quantitative Plaque Volume and Burden Measures and MACE (n = 4267)

Measure	Univariable		Multivariable model 1^a^		Multivariable model 2^b^	
	HR (95% CI)	*P* value	HR (95% CI)	*P* value	HR (95% CI)	*P* value
**Plaque volume (per 100 mm^3^)**						
Total	1.09 (1.06-1.12)	<.001	1.07 (1.03-1.10)	<.001	1.00 (0.94-1.08)	.93
Calcified	1.14 (1.08-1.20)	<.001	1.11 (1.04-1.18)	.002	0.99 (0.85-1.17)	.95
Noncalcified	1.16 (1.10-1.21)	<.001	1.11 (1.05-1.18)	<.001	1.01 (0.92-1.11)	.88
Low attenuation	3.75 (2.60-5.41)	<.001	3.00 (1.68-5.33)	<.001	1.84 (0.91-3.74)	.09
**Plaque burden (per 10%)**						
Total	1.38 (1.26-1.50)	<.001	1.31 (1.19-1.44)	<.001	1.18 (1.05-1.34)	.006
Calcified	1.39 (1.20-1.62)	<.001	1.29 (1.08-1.52)	.004	1.10 (0.88-1.36)	.40
Noncalcified	1.40 (1.27-1.54)	<.001	1.33 (1.19-1.48)	<.001	1.20 (1.05-1.37)	.007
Low attenuation	2.38 (1.50-3.75)	<.001	2.11 (1.21-3.66)	.008	1.64 (0.80-3.37)	.18

Abbreviations: HR, hazard ratio; MACE, composite of all-cause death, nonfatal myocardial infarction, or hospitalization for unstable angina.

^a^ Model 1 was adjusted for age, sex, race, atherosclerotic cardiovascular disease risk, and statin use.

^b^ Model 2 was adjusted for components of model 1 plus continuous coronary artery calcium, stenosis ≥50%, and high-risk plaque features.

### Volume and Burden of Plaque Subtypes and Relationships to Qualitative CT Findings

PVs and PBs for the overall cohort and the subgroup of patients with any detectable plaque are detailed in eTable 2 in Supplement 2. In the overall cohort, including those with no plaque, median (IQR) TPV was 39.8 (0.00-167) mm^3^, median (IQR) NCPV was 22.5 (0.00-101) mm^3^, and median (IQR) LAPV was 0.03 (0.00-2.0) mm^3^. On average, 64.7% of TPV was noncalcified, and 2.6% was low-attenuation plaque. The median (IQR) plaque burdens were as follows: TPB, 27.0% (0.00%-43%); NCPB, 14.1% (0.00%-29%); and LAPB, 0.02% (0.00%-0.52%). Higher TPV and TPB were both associated with a greater burden of cardiovascular risk factors (eTable 3 in Supplement 2). Quantitative plaque measures were significantly correlated with all qualitative CT findings, including CAC score, luminal stenosis severity, HRP features, and the CT-adapted Leaman score (eTable 4 in Supplement 2).

### Association of Quantitative Plaque With Events

The primary composite MACE occurred in 121 of 4267 patients (2.8%), including all-cause death (n = 56; 1.3%), cardiovascular death (n = 30; 0.7%), nonfatal MI (n = 23; 0.5%), and hospitalization for unstable angina (n = 44; 1.0%).

Highest quartiles of all quantitative plaque measures were significantly associated with incident MACE after full adjustment (eTable 5 in Supplement 2). Continuous quantitative plaque measures, including TPV and TPB and their subcomponents, predicted MACE in the unadjusted analyses and remained significant after adjustment for age, sex, race, ASCVD risk, and statin use (Table 2; model 1), with only negligible change in hazard ratios (HRs). However, only TPB (adjusted hazard ratio [aHR] per 10%, 1.18; 95% CI, 1.05-1.34; *P* = .006) and NCPB (aHR per 10%, 1.20; 95% CI, 1.05-1.37; *P* = .007) remained significantly associated with MACE after further adjustment for qualitative CCTA findings, including continuous CAC score, stenosis 50% or more, HRP features (model 2). These associations also remained significant after adjusting for each of the individual qualitative CCTA findings (eTable 6 in Supplement 2, models 3-6). Continuous TPV, CPV, NCPV, LAPV, CPB, and LAPB were not significantly associated with events after adjustment for clinical risk and qualitative CCTA findings (Table 2).

Sensitivity analyses, investigating hard cardiovascular events, including nonfatal MI and cardiovascular death, demonstrated that both total TPB (aHR, 1.22; 95% CI, 1.06-1.40; *P* = .005) and NCPB (aHR, 1.26; 95% CI, 1.08-1.48; *P* = .004) were significantly associated with CV events in model 1 (eTable 7 in Supplement 2). While these associations were attenuated when adjusting for all qualitative CCTA findings (model 2), NCPB remained a significant predictor of cardiovascular events when adjusting individually for continuous CAC (aHR, 1.28; 95% CI, 1.07-1.53; *P* = .006), stenosis 50% or more (aHR, 1.20; 95% CI, 1.01-1.43; *P* = .04), or HRP features (aHR, 1.20; 95% CI, 1.01-1.43; *P* = .04) (models 3-6).

In a subgroup analysis of patients with CAC score of 0, MACE risk increased across higher quartiles of NCPV (4/20 patients [20%]). The strength of this observation is attenuated by the small number of events among a few patients in this quartile. Lesser MACE risks were observed across the top quartiles of TPV (1/11 [9.1%]), CPV (0 patients), and LAPV (4/15 [7.8%]) (eTable 8 in Supplement 2). Among patients with CAC score 0, NCPV (HR, 1.73; 95% CI, 1.31-2.29; *P* < .001), NCPB (HR, 1.48; 95% CI, 1.22-1.80; *P* < .001), and LAPB (HR, 3.40; 95% CI, 1.41-8.26; *P* = .007) were associated with MACE (eTable 9 in Supplement 2).

### Optimal Plaque Volume and Burden Cutoffs to Predict Adverse Events

**Figure 1.**
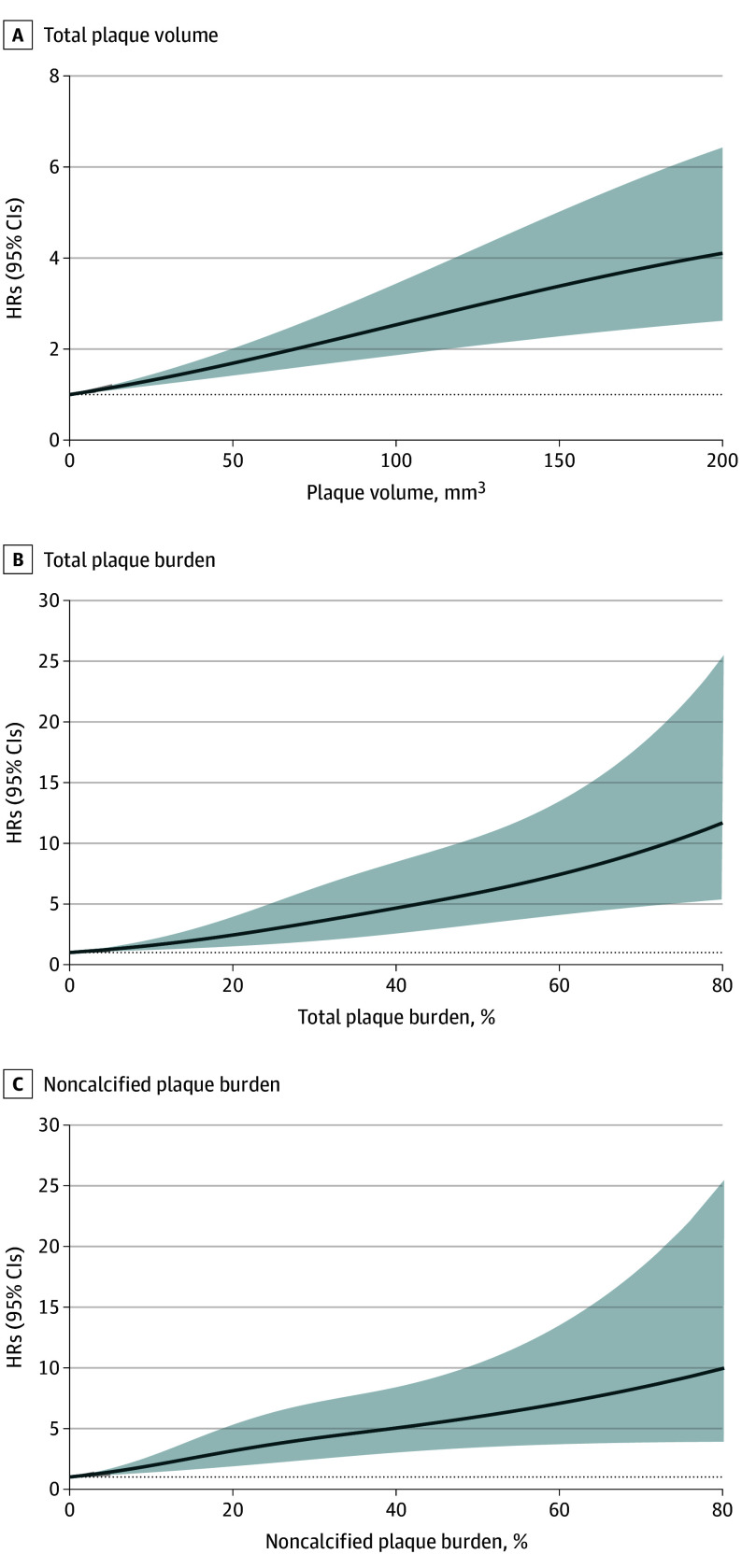
Cox Cubic Spline Regressions HRs indicates hazard ratios.

**Figure 2.**
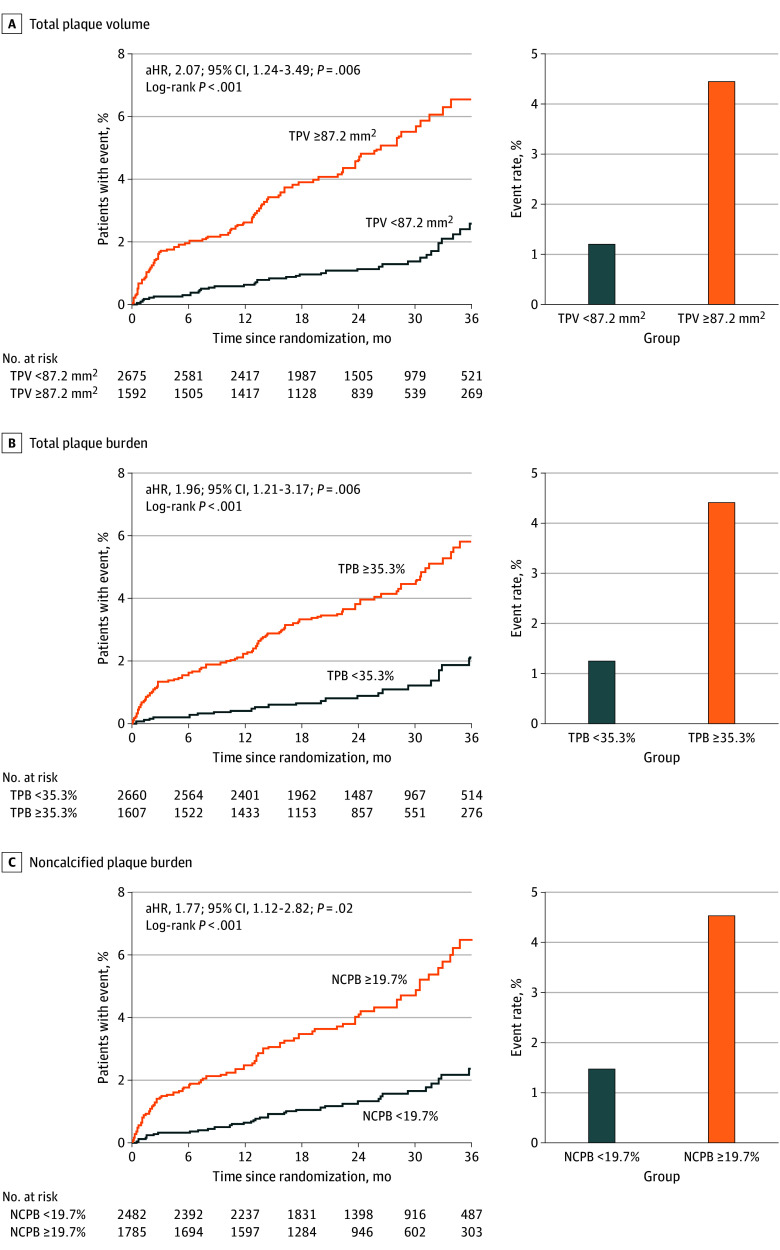
Event Rates Based on Quantitative Plaque Metric Thresholds Illustration of time-to-event curves on the left and absolute event rates on the right for total plaque volume (TPV), total plaque burden (TPB), and noncalcified plaque burden (NCPB) at the data-driven thresholds. aHR indicates adjusted hazard ratio.

Increasing TPV, TPB, and NCPB values were each associated with a progressively higher risk for MACE in a relatively linear fashion (Figure 1). Optimal TPV cutoff for MACE prediction in this cohort was 87.2 mm^3^ (compared with the cohort median of 39.8 mm^3^). Patients with a TPV 87.2 mm^3^ or greater had significantly higher cumulative event rates at a 2.5-year median follow-up period (Figure 2 and Table 3) and a substantially increased risk of MACE after adjustment for clinical risk factors and statin use (aHR, 2.89; 95% CI, 1.90-4.38; *P* < .001). This association remained significant after additional adjustment for qualitative CCTA plaque findings (aHR, 2.07; 95% CI, 1.24-3.49; *P* = .006) and when accounting for each qualitative CCTA feature individually (aHRs ranging from 1.94 to 3.11) (eTable 10 in Supplement 2). The C statistic demonstrated significant improvement vs the base model when adding TPV cutoff (C statistic: 0.765 [95% CI, 0.762-0.768] vs 0.750 [95% CI, 0.747-0.753] for the base model; *P* = .005).

**Table 3.** Univariable and Multivariable Assessment of Quantitative Plaque Volume/Burden Data-Driven Thresholds and MACE (n = 4267)

Measure	Univariable		Multivariable model 1^a^		Multivariable model 2^b^	
	HR (95% CI)	*P* value	HR (95% CI)	*P* value	HR (95% CI)	*P* value
TPV ≥87.2 mm^3^	3.52 (2.41-5.14)	<.001	2.89 (1.90-4.38)	<.001	2.07 (1.24-3.49)	.006
TPB ≥35.3%	3.45 (2.35-5.03)	<.001	2.76 (1.86-4.09)	<.001	1.96 (1.21-3.17)	.006
NCPB ≥19.7%	3.02 (2.06-4.41)	<.001	2.46 (1.65-3.65)	<.001	1.77 (1.12-2.82)	.02

Abbreviations: HR, hazard ratio; MACE, composite of all-cause death, nonfatal myocardial infarction, or hospitalization for unstable angina; NCPB, noncalcified plaque burden; TPB, total plaque burden; TPV, total plaque volume.

^a^ Model 1 was adjusted for age, sex, race, atherosclerotic cardiovascular disease risk, and statin use.

^b^ Model 2 was adjusted for components of model 1 plus continuous coronary artery calcium, stenosis ≥50%, and high-risk plaque features.

For TPB, an optimal cutoff in this cohort of 35.3% (vs median of 27.0%) was identified. Patients with TPB 35.3% or greater had significantly higher cumulative event rates (Figure 2) and remained at significantly increased risk for MACE after adjusting for clinical risk factors and statin use (aHR, 2.76; 95% CI, 1.86-4.09; *P* < .001) and after including qualitative plaque variables (aHR, 1.96; 95% CI, 1.21-3.17; *P* = .006) (Table 3). The C statistic showed significant improvement in MACE prediction when adding TPB compared with the base model (C statistic: 0.764 [95% CI, 0.761-0.767] vs 0.750 [95% CI, 0.747-0.753] for the base model; *P* = .006).

An NCPB cutoff of 19.7% or greater was associated with significantly higher cumulative event rates (Figure 2) and independently predicted MACE (aHR, 1.77; 95% CI, 1.12-2.82; *P* = .02) after controlling for clinical risk, statin use, and qualitative CCTA plaque findings (Table 3). Prediction of MACE was significantly improved by adding NCPB (C statistic, 0.761 [95% CI, 0.758-0.764] vs 0.750 [95% CI, 0.747-0.753] for the base model; *P* = .01).

## Discussion

In this large cohort of stable, symptomatic outpatients with no known CAD, we demonstrate that CCTA-derived coronary PVs and PBs are low but associated with a higher cardiovascular risk profile, more severe qualitative CCTA-derived CAD findings, and incident MACE. Our data suggest that among people with a first evaluation for CAD, TPB and NCPB may be predictive of MACE after adjustment for cardiovascular risk factors, statin use, and established qualitative CCTA findings, such as CAC score, obstructive stenosis 50% or more, and HRP features. Clinical utility in our cohort was suggested by the 2-fold to 3-fold increase in MACE risk above thresholds derived from the ROC curve intrinsic to this dataset of TPV 87.2 mm^3^ or greater, TPB 35.2% or greater, and NCPB 19.7% or greater. These relatively low thresholds need to be validated in other populations to substantiate their broader clinical utility.

The PROMISE trial offers a unique opportunity to study quantitative coronary plaque measures in a large, well-characterized population (median ASCVD risk, 11%), stable symptoms, and no known CAD. This patient population is critical to assess as it represents a substantial number of patients encountered in everyday practice, for whom current risk stratification methods are imperfect and preventive therapies are often underused. The value of CCTA-based coronary plaque quantification has been proven in higher-risk populations and those with established CAD,^7,12,25,26,27,28,29^ yet lower-risk patients with early CAD, like those in PROMISE, are understudied, although they are arguably the group in whom additional prognostic data and more aggressive treatment may be most beneficial.^13^ There are several reasons for this. Historically, people without obstructive CAD were considered to be at low risk. Despite data from PROMISE, CONFIRM, and other studies showing that even nonobstructive plaque increases the likelihood of events, guidelines rarely offer recommendations for intensifying preventive strategies based on the findings of nonobstructive CAD.^1,2,4^ However, a call for shifting the paradigm from events to early detection and treatment of plaque has been highlighted.^13^ Barriers to implementing this shift go beyond the faulty assumption that very low plaque volume and burden are relatively benign early in the disease course to the reasonable question of whether or not it is possible to identify prognostic cut points in those with early stages of CAD. There are few large, lower-risk populations with CCTA data and adjudicated outcomes that provide opportunities to explore both foundational questions.

Our study addresses these concerns: in this cohort with a first diagnosis of CAD, PVs are low relative to previously studied populations with established disease, but we show that they can be reliably quantifiable (with the possible exception of low-attenuation plaque). Further, PVs are related to clinical risk factors and other qualitative CCTA findings, confirming their clinical importance. Most importantly, however, after normalizing them to vessel volume, TPB and NCPB carry meaningful independent prognostic value beyond that provided by clinical risk factors and currently utilized qualitative features on CCTA. These results may support the role of assessing CCTA-based plaque among early CAD populations and in drug development, where NCPV is currently under consideration as a prognostic biomarker in the FDA’s biomarker qualification program.^11^ There was a higher incidence of MACE events (20%) among the highest quartile of NCPB patients within the subgroup who had CAC score 0, though the number of events in this subgroup was very small (4 events among 20 patients). In the Incident Coronary Syndromes Identified by Computed Tomography (ICONIC) study, a nested case-control study of patients within the dynamic CONFIRM registry, patients with acute coronary syndrome were propensity matched for risk factors, and CCTA evaluated obstructive CAD with control patients who had not experienced a coronary event. Among 234 matched pairs, patients with acute coronary syndrome had significantly higher values of NCPV and maximal cross-sectional plaque burden. Patient-level percent diameter stenosis, cross-sectional plaque burden, fibrofatty and necrotic core volume, and HRP features were each individually associated with a higher aHR of acute coronary syndrome. Of note, the TPV among controls in the ICONIC population was more than twice that of PROMISE participants (267 mm^3^ vs 139 mm^3^).^10,12^ The investigators concluded that certain plaque features, beyond percent stenosis, could identify high-risk patients. Our results corroborate and advance these findings by showing that even lower PVs and associated PBs are prognostically important. We also sought to identify potentially clinically relevant, data-driven threshold definitions, which will require external validation. Using plaque-based cut points to risk-stratify the more than 50% of patients with a low (<15%) pretest probability for obstructive CAD in PROMISE may provide an approach to improve application of preventive therapies.^30^

The Scottish Computed Tomography of the Heart (SCOT-HEART) trial (N = 1769) investigated a cohort with a 25% prevalence of obstructive CAD (compared with 9% in PROMISE).^7^ Although both studies examined outpatients with stable chest pain, the more advanced disease found in SCOT-HEART may be attributed to higher cardiovascular risk profiles, such as a greater proportion of men (59% vs 48%) and the inclusion of patients with known CAD. Also, events were defined differently in the 2 trials, limiting the comparison.

SCOT-HEART did not describe PVs but reported PBs substantially higher than our findings (TPB 39% vs 27%, NCPB 36% vs14%, LAPB 0.4% vs 0.02%), all of which were associated with adverse outcomes. Although these measurements were also associated with outcomes in PROMISE, the relationship with LAPB became nonsignificant after adjusting for qualitative CT findings. In SCOT-HEART, LAPB more than 4% was independently associated with MI (aHR, 4.65; 95% CI, 2.06-10.5).^7^ LAPV has been linked to advanced lesions, such as large thin-cap atheroma, often seen as an HRP component (eg, napkin-ring sign and low-attenuation plaque) on CCTA,^31,32^ making it an attractive target. In PROMISE, LAPB more than 4% was associated with MACE, but with less than half of the HR observed in SCOT-HEART (aHR, 1.96 vs 4.65). Most importantly, the LAPV and LAPB values were too low in PROMISE to model an optimal cut point for prognosis. Specifically, in PROMISE, LAPV represented only 2.6% of total plaque volume, and only 4.6% of the cohort had LAPB more than 4%, essentially precluding its use as a prognostic marker in lower-risk populations with first diagnosis of CAD.

### Limitations

Because the parent trial did not show that clinical outcomes were improved with an initial strategy of CCTA vs functional testing, the findings here should be considered hypothesis generating. This study has several limitations. First, although plaque quantification was performed in a central core laboratory using validated software and expert readers, volumetric analysis remains time- and resource-intensive, which may hinder its routine availability in clinical practice. Wider adoption would require streamlined workflows and automated tools. Moreover, plaque quantification to this date remains vendor-specific, and the field lacks standardized parameters for the measurement and reporting of quantitative plaque burden. Second, the PROMISE trial included a North American outpatient population undergoing initial evaluation for suspected CAD, such that the findings may not be generalizable to asymptomatic individuals, higher-risk cohorts, or those with prior CAD. Third, the follow-up period (median 25 months) may underestimate the long-term prognostic value of plaque quantification, especially for slower-progressing CAD. Fourth, estimated glomerular filtration rate was not included in the regression models. Lastly, the exploratory nature of this analysis precluded multiple testing and suggests the need for our findings to be confirmed in other cohorts as well as inherently raises the possibility of type I error, given the lack of statistical adjustments for multiple testing. This also limits the interpretation of the identified thresholds that were derived within this dataset, are only exploratory in nature, and warrant further validation.

## Conclusions

In this large cohort of symptomatic outpatients without known CAD, plaque volumes and burden were strongly associated with traditional cardiovascular risk factors, qualitative CCTA plaque findings, and MACE. Continuous TPB and NCPB independently predicted MACE after adjusting for traditional cardiovascular risk factors, statin use, and established CT findings, including CAC, obstructive stenosis 50% or more, and HRP features. Although there was a signal of excess MACE among persons in the highest quartile of NCPB within the subgroup with CAC score 0, the number of events was too small to draw firm conclusions. The clinical importance of these findings may lie in their independent prediction of MACE and in their relatively low quantitative thresholds for higher risk, though further validation is required. This secondary analysis (not prespecified) supports the need for prospective investigation of the clinical utility of CCTA-based quantitative risk estimation in early CAD.

## Notes

Supplement 1. Trial protocol

Supplement 2.
eMethods

eResults
**eTable 1.** Baseline demographics by the median total plaque burden. **eTable 2.** Description of quantitative plaque measures. **eTable 3. **Baseline characteristics by plaque volume and burden measures **eTable 4.** Median PV measures and qualitative CT measures **eTable 5. **Multivariable assessment of quantitative plaque volume/burden quartiles and MACE. **eTable 6. **Multivariable assessment of quantitative plaque volume/burden and MACE **eTable 7.** Univariable and multivariable assessment of quantitative plaque volume/burden and non-fatal MI and cardiovascular death. **eTable 8.** Crude MACE Rates (All-cause death, MI, UAP) stratified by Plaque Volume/Burden Quartiles and CAC categories **eTable 9**. Cox regression hazard ratios (All-cause death, MI, UAP) for continuous plaque volume and plaque burden across CAC categories **eTable 10. **Univariable and multivariable assessment of quantitative plaque volume/burden data-driven thresholds and MACE. **eFigure. **Consort diagram.

Supplement 3. Data sharing statement
