# Supplementary material for: Prognostic Value of Plaque Volume in Patients With First Diagnosis of Coronary Artery Disease: A Substudy of the PROMISE Randomized Clinical Trial
Source: JAMA Cardiol. 2026 Feb 11;11(3):259–67. doi: 10.1001/jamacardio.2025.5520 (PMC12895320; doi:10.1001/jamacardio.2025.5520)
Supplement: Supplement 1. — Trial protocol [file jamacardiol-e255520-s001.pdf]

Protocol

This trial protocol has been provided by the authors to give readers additional information about their work.

Protocol for: Douglas PS, Hoffmann U, Patel MR, et al. Outcomes of anatomical versus functional testing for coronary artery disease. N Engl J Med 2015;372:1291-300. DOI: 10.1056/NEJMoa1415516

This supplement contains the following items:

1. Original protocol, final protocol, summary of changes.
2. Statistical analysis plan (no changes).

The **PROMISE** Trial FINAL Protocol

PROspective Multicenter Imaging Study for Evaluation of Chest Pain

**Sponsor:**	National Heart, Lung, and Blood Institute (NHLBI)
**Clinical Coordinating Center**	
**Principal Investigator:**	Pamela S. Douglas, MD
**Co- Investigators:**	Rowena Dolor, MD<br>Alan Go, MD<br>Daniel Mudrick, MD<br>Manesh R. Patel, MD<br>Eric J. Velazquez, MD
**Statistical / Data Coordinating Center**	
**Principal Investigator:**	
**Co- Investigators:**	Kerry L. Lee, PhD<br>Andrzej S. Kosinski, PhD<br>Hussein R. Al-Khalidi, PhD
**Diagnostic Testing Coordinating Center**	
**Principal Investigator:**	Udo Hoffmann, MD, MPH
**Co- Investigators:**	Mitchell Krucoff, MD<br>Michael Picard, MD<br>James Udelson, MD
**Economics / Quality of Life**	
**Principal Investigator:**	Daniel B. Mark, MD, MPH
**Co- Investigator:**	Kevin Anstrom, PhD
**Investigation of**	Diagnostic testing strategies for suspected CAD including use of coronary CT angiography (CTA)

FINAL Date: **April 12, 2010**
Amended:
Administrative Change:

CONFIDENTIAL

This document is confidential and the property of Duke Clinical Research Institute. No part of it may be transmitted, reproduced, published, or used by other persons without prior written authorization from the DCRI. This document may be disclosed to the appropriate IECs / IRBs or to duly authorized representatives of the US Food and Drug Administration or other regulatory authority under the condition that they maintain confidentiality.

Investigator Protocol Signature Page

I have read and understand the protocol and agree that it contains all the ethical, legal and scientific information necessary to conduct this study. I will personally conduct the study as described. I will provide copies of the protocol to all physicians, nurses and other professional personnel responsible to me who will participate in the study. I am aware that this protocol must be approved by the Institutional Review Board or Ethics Committee. I agree to adhere strictly to the attached protocol. I agree that clinical data entered on case report forms by me and my staff will be supplied to the DCRI and may be utilized by the DCRI in various ways such as for submission to governmental regulatory authorities and/or in combination with clinical data gathered from other research sites, whenever applicable. I agree to allow DCRI monitors and auditors full access to all medical records at the research facility for subjects screened or randomized in the study. I agree to provide all subjects with informed consent forms, as required by government regulations and International Conference on Harmonization guidelines.

Version Date: April 12, 2010

__________________________________ __________________________

Principal Investigator (print name) **Site Name and Number**

__________________________________ ________________________

Principal Investigator (signature) **Date**

TABLE OF CONTENTS

A INTRODUCTION 7

A1 STUDY SYNOPSIS 7
A2 PRIMARY HYPOTHESIS 8
A3 SIGNIFICANCE OF THE STUDY 9

B BACKGROUND 9

B1 PRIOR LITERATURE AND STUDIES 9
B2 RATIONALE: THE URGENT NEED FOR A RANDOMIZED TRIAL OF DIAGNOSTIC STRATEGIES IN SUBJECTS WITH SUSPECTED CAD 12

C STUDY OVERVIEW AND OBJECTIVES 13

C1 OVERVIEW OF THE PROMISE TRIAL 13
C2 PRIMARY AIM 14
C3 SECONDARY AIMS 14
C4 RATIONALE FOR THE SELECTION OF OUTCOME MEASURES 14
C5 RATIONALE FOR SELECTION OF TESTING IN EACH EXPERIMENTAL ARM 15

D INVESTIGATIONAL PLAN 16

D1 OVERVIEW OF TRIAL DESIGN 16
D2 SUBJECT POPULATION 17
D3 DIAGNOSTIC TESTING 20

E STUDY PROCEDURES 23

E1 SCREENING FOR ELIGIBILITY 23
E2 SCHEDULE OF ASSESSMENTS 23
E3 SAFETY 26
E4 STUDY OUTCOME MEASUREMENTS AND ASCERTAINMENT 27
E5 INDEPENDENT CLINICAL EVENT ADJUDICATION COMMITTEE 28
E6 QUALITY OF LIFE ASSESSMENTS 28
E7 ECONOMIC ASSESSMENTS 29
E8 PROMISE BIOREPOSITORIES 29

F STATISTICAL PLAN 30

F1 SAMPLE SIZE DETERMINATION AND STATISTICAL POWER 30
F2 STATISTICAL ANALYSIS PLAN 31
F3 INTERIM ANALYSES 36

G DATA HANDLING AND RECORD KEEPING 38

G1 CONFIDENTIALITY AND SECURITY 38
G2 TRAINING 39
G3 ELECTRONIC CASE REPORT FORM (ECRF) 39
G4 RECORDS RETENTION 39

H STUDY MONITORING, AUDITING, AND INSPECTING 39

H1 STUDY MONITORING PLAN 39
H2 AUDITING AND INSPECTING 39

I STUDY ADMINISTRATION 39

I1 ORGANIZATION AND PARTICIPATING CENTERS 39
I2 FUNDING SOURCE 40
I3 STUDY TIMETABLE 40

J REFERENCES 41

GLOSSARY

ABI	Ankle Brachial Index
ACC	American College of Cardiology
ACRIN	American College of Radiology Imaging Network
ACS	Acute Coronary Syndrome
AHA	American Heart Association
BARI	Bypass Angioplasty Revascularization Investigation
BMI	Body Mass Index
CABG	Coronary Artery Bypass Graft
CAD	Coronary Artery Disease
CC	Coordinating Center
CCC	Clinical Coordinating Center
CCS	Canadian Cardiovascular Society
CEC	Clinical Events Classification Committee
CES-D	Center for Epidemiologic Studies-Depression
CK	Creatine Kinase
CK-MB	Creatine Kinase MB
COCATS	ACC Core Cardiology Training Symposium
CPK	Creatine Phosphokinase
CRA	Clinical Research Associate
CTA	Coronary Tomographic Angiography
DCRI	Duke Clinical Research Institute
DOFG	Duke Clinical Research Institute Outcomes and Follow-up Group
DSMB	Data and Safety Monitoring Board
DTCC	Diagnostic Testing Coordinating Center
EAC	External Advisory Committee
ECG	Electrocardiogram
e-CRF	Electronic Case Report Form
EDC	Electronic Data Capture
EQOL	Economics and Quality of Life
ER	Emergency Room
ERES	Electronic Records/Electronic Signatures
EuroQOL	European Quality of Life
FDA	Food and Drug Administration
FWA	Federal Wide Assurance
g/l	Grams Per Liter
HDL	High Density Lipoprotein
IRB	Institutional Review Board
IV	Intravenous

IVRS	Interactive Voice Response System
LDL	Low Density Lipoprotein
LVEF	Left Ventricular Ejection Fraction
MACE	Major Adverse Cardiovascular Events
MI	Myocardial Infarction
mSv	MilliSievert
NHLBI	National Heart, Lung and Blood Institute
NYHA	New York Heart Association
PAD	Peripheral Arterial Disease
PCI	Percutaneous Coronary Intervention
PI	Principal Investigator
QOL	Quality of Life
SDCC	Statistical and Data Coordinating Center

A Introduction

A1 *Study Synopsis*

**Sponsor(s)**	National Heart, Lung, and Blood Institute (NHLBI)
**Protocol Title**	The PROMISE Trial - **PRO**spective **M**ulticenter **I**maging **S**tudy for **E**valuation of Chest Pain
**Diagnosis and Main Criterion for Inclusion**	Symptomatic subjects without known coronary artery disease (CAD) for whom a non-emergent, noninvasive cardiovascular diagnostic test for CAD is planned.
**Primary Study Objective**	To determine whether an initial non-invasive anatomic imaging strategy with coronary CT angiography (CTA) will improve clinical outcomes in subjects with symptoms concerning for coronary artery disease relative to an initial functional testing strategy (usual care).
**Primary Endpoint**	Time to first event using the composite of the following major cardiovascular events:<br>• Death<br>• Myocardial infarction<br>• Major complications from cardiovascular procedures including testing (stroke, major bleeding, anaphylaxis and renal failure)<br>• Unstable angina hospitalization
**Secondary Endpoints**	• Death or myocardial infarction or unstable angina hospitalization<br>• Death or myocardial infarction<br>• Major complications from cardiovascular procedures and testing (stroke, major bleeding, anaphylaxis, and renal failure)<br>• Medical costs, resource use, and incremental cost effectiveness<br>• Health related quality of life
**Secondary Safety Endpoint**	• Cumulative radiation exposure
**Primary Hypothesis**	An initial anatomic testing strategy will provide information that will result in superior long-term health outcomes as compared to an initial functional testing strategy.

**Study Design**	Pragmatic randomized trial of clinical effectiveness of diagnostic testing strategies for CAD, to be performed in outpatient settings including urgent care, primary care, and cardiology offices. Qualifying patients presenting with new or worsening symptoms suspicious for clinically significant CAD who require diagnostic testing and have not been previously evaluated for this episode of symptoms will be randomized to an initial strategy of either anatomic or functional testing. All subsequent decisions regarding additional testing, medications and/or procedures will be at the discretion of the responsible clinical care team.<br>Within the functional testing arm, the subject's care team will select the specific test to be performed (exercise ECG, stress nuclear, or stress echo) consistent with 'usual care' in that practice setting. The subject's care team will be provided with 'Information sheets' summarizing current standards for test interpretation and preventive care, but specific medical treatment will not be mandated by the trial.
**Duration of Study Participation**	The trial will have an approximate 2-year enrollment period and 2-year minimum follow up period. All subjects will be followed from enrollment until either death or the end of the follow-up period.
**End-of-Study Definition**	2 year minimum follow up of all patients, with expected 30-month average follow up for clinical status assessment.
**Number of Subjects**	Approximately 10,000 subjects will be enrolled, which is expected to provide 90% power to detect a 20% reduction in the primary composite event rate in the anatomic testing arm as compared to functional testing.
**Number of Sites**	~200-250
**Study Follow-up**	The sites will conduct the initial study follow up at 60 days. Follow-up contact at 6 months post randomization and every 6 months thereafter will be conducted centrally by the DCRI.

A2 *Primary Hypothesis*

PROMISE's primary hypothesis is that an initial anatomic testing strategy utilizing 64-slice or better coronary CT angiography (CTA) technology in subjects with low to intermediate risk for coronary artery disease (CAD) will reduce the composite primary endpoint (all cause death, myocardial infarction, major peri-procedural complications and hospitalization for unstable angina) when compared with an initial functional testing strategy over an average of 2.5 years of follow-up.

A3 *Significance of the Study*

PROMISE is the first large randomized trial comparing the two major alternative non-invasive diagnostic strategies for the initial assessment of stable symptoms suspicious for possible CAD. Community-based practices are anticipated to contribute substantially to subject enrollment which will enhance generalizability of findings in contrast to previous smaller studies which relied on 'expert' centers staffed by cardiologists and radiologists. This trial will provide an unbiased comparison of usual care testing strategies with new CTA technology that is expected to yield definitive and unique evidence regarding the benefits and risks of these alternative approaches, with the primary endpoint being superior health outcomes.

B Background

B1 *Prior Literature and Studies*

1.a Current Management of Patients with Chest Pain: Extent of the Problem

Chest pain is a common presentation, with ~4 million Americans newly diagnosed with angina[1] and >6 million presenting to emergency rooms with chest pain annually.[2] The annual incidence of new angina ranges from 1% in middle aged women to nearly 4% in elderly men in the US,[1] with rates in diabetics of up to 10%.[3] Overall mortality is doubled compared to the general population, and rates range from less than 1% per year for those ultimately diagnosed as 'non-anginal' to almost 10% per year for those with CAD and an unfavorable clinical risk profile.[4-8] (See Table 1) These data suggest that between 4 and 5 million Americans require noninvasive testing for chest pain symptoms every year. The high event rates in unselected but symptomatic populations support performance of a randomized trial to define optimal testing strategies in this at-risk group and to guide care.

Table 1: Event Rates in Populations with New Onset Chest Pain

Study	Population	Endpoint	Lowest event rates	Highest event rates	Follow up
Daly BMJ 2006 [4]	3,031 (58%M)	Death, MI	2.3% overall	3.9% with angiographic CAD	1 year
Mudrick Circ 2009[8]	98,872	Death, MI, Stroke	1.07%	N/A	1 year
Sekhri Heart 2007[9]	8,762 (52%M)	Death, MI, Unstable angina hospitalization	0.83% noncardiac CP	8.62% angina	1 year
			2.73% noncardiac CP	16.52% angina	3 years
Sekhri BMJ 2008 [5]	8,176 (53%M)	Coronary death, MI	3-9% nonspecific CP	16-19% typical CP	2.5 years
Clayton BMJ 2005 [6]	7,311 (79%M)	Death, MI, stroke	4% lowest risk decile	35% highest risk decile	5 years

In patients presenting with symptoms suggestive of CAD, such as chest pain or exertional dyspnea, the clinical risk profile and presentation are often insufficient to definitively exclude CAD and/or are not sufficient to implement invasive evaluation or revascularization without additional information. Thus, noninvasive diagnostic testing is required in the majority of such patients. Consistent with these data, an exercise ECG was performed in 65% of all British chest pain patients,[7] rising to over 90% in those

eventually diagnosed with angina. In the Euro Heart Survey of Chronic Stable Angina, 76% of patients received an exercise ECG while 18% had a stress imaging study and 64% had a resting echocardiogram. A coronary angiogram was performed or planned in 41%.[10]

1.b Current Diagnostic Strategies Using Functional Tests are Inadequate

ACC/AHA Guidelines and current non-invasive diagnostic testing includes exercise ECG, stress echocardiography and stress nuclear imaging, all of which are based on detecting stress-induced myocardial ischemia caused by obstructive CAD. [11-13] All are also associated with high rates of false positive and false negative results in the detection of significant CAD (defined as a left main obstruction >50% and/or any major epicardial vessel obstruction >70%), limited prognostic discrimination, relatively short "warranty period" for a negative test, and an inability to detect possibly significant non-obstructive CAD. The literature regarding functional test characteristics is summarized below in Table 2, along with reports of clinical event rates after testing.

False negative test results may relay needed treatments and lead to worse clinical outcomes, while false positive test results may lead to unnecessary treatment and procedures. Evidence of the inadequacies of current diagnostic strategies includes the occurrence of undetected MI (2.1%) and unstable angina (2.3%) in patients with suspected acute coronary syndrome who are discharged from the emergency room.[14] A missed diagnosis of MI was associated with a 9.8% 30-day mortality vs. only 5.7% in hospitalized patients or a relative risk of 1.9. Other relevant evidence comes from an analysis of the large ACC National Cardiovascular Data Registry which suggests that misdiagnosis of chest pain is common in community practice. [15] Of nearly 400,000 patients without known CAD who underwent elective diagnostic catheterization, 84% had prior non-invasive testing, which was positive in 82%. Nevertheless, only 38% of patients overall, and 41% of those with positive tests, had obstructive CAD (left main stenosis >50%; major epicardial vessel >70%). Thus current clinical practice, as guided by functional stress testing, results in a pattern of referral to invasive angiography, which does not lead to revascularization in the majority of patients, despite the inherent risks and expense of an invasive test.

1.c CTA is Highly Accurate in Detecting and Excluding Obstructive and Non-obstructive CAD

The recent technological advance of coronary CT angiography (CTA) permits direct visualization of the coronary arteries, allowing non-invasive detection of significant stenoses and with great accuracy.[16-19] CTA also extends the spectrum of disease by detecting non-obstructive lesions and visualizing coronary plaque, important prognostic predictors. CTA test characteristics and associated event rates are shown in Table 2 below. The superior ability of CTA to detect obstructive CAD means that patients at greatest risk will be more accurately identified and treated with life saving therapies proven to reduce death and MI by about 30%,[20] while those without disease will not be subjected to unnecessary additional, perhaps invasive, testing.

Table 2: Diagnostic Test Characteristics and Resulting Event Rates				
	**Stress Nuclear**	**Stress Echo**	**Exercise ECG**	**CTA**
**TEST CHARACTERISTICS** (CAD with stenosis ≥70%; LM≥ 50%)				
Sensitivity	76-94% [21-24]	87-88% [21-24]	52% [21]	85-99% [16,17,19,24,25]
Specificity	62-88% [21-24]	77-84% [21-24]	71% [21]	82-95% [16,24,25]
Event Rate after Negative Study (Prognostic Accuracy)	0.5-2.0%/y [26-30]	0.5-2.0%/y [26-30]	3%/y [5]	<0.3%/y [31,32]
Detection of Non-Obstructive CAD	N/A	N/A	N/A	Similar to Obstructive CAD
**OVERALL EVENT RATES (1 yr; all patients)**				
Death	0.45-2.5% [33-36]	2.9-6.6% [37-39]	0.5-5.0% [40-42]	0.3-2.2% [31,32,43-46]
Non-Fatal MI	0.9-1.3% [33-36]	0.9-1.3% [37-39]	-	0.4-0.6% [31,32,43-46]
Unstable Angina Hospitalization	3.8%	-	-	0.2-4.2%

1.d Additional Important Considerations in Comparing Test Characteristics

The evidence base to guide diagnostic testing is limited: The majority of the studies discussed above were conducted in single academic centers, and are plagued by numerous sources of bias (verification, selection, etc), making evaluation of true accuracy very difficult.[47,48] Data from academic research settings provide only a rough indication of actual performance in the real world.[49-51] Even these data, however, are based on convenience samples of subjects already referred for a particular test rather than the true population of interest.[51] The absence of high quality unbiased comparative data on test performance and outcomes has led to the unusual situation of conflicting ACC/AHA Guidelines, with each of these relevant documents recommending the initial use of a different test: treadmill ECG, stress echocardiography or stress perfusion imaging.[11-13,52,53] Further, very few studies have addressed Fryback and Thornberry's highest levels of evidence for assessing diagnostic testing: subject outcome efficacy and societal efficacy.[54] This situation has resulted in clinical confusion, performance of multiple tests and marked variation in clinical practice that can only be addressed by high quality effectiveness research based on improving patient outcomes.

Obstructive CAD is relatively uncommon, but nonobstructive CAD is common, clinically important and difficult to detect with functional tests: The NCDR analysis shows that the absence of significant obstructive disease is common in patients undergoing diagnostic catheterization. Of the 62% of patients without significant CAD, 2/3 had non-obstructive CAD (20-70% epicardial and less than 50% left main) and 1/3 had no CAD (defined as stenosis <20%).[15] A similar pattern has been noted by others: in 400 patients at low-intermediate risk for CAD undergoing CTA, 47% had nonobstructive CAD, 13% had obstructive stenoses, and 27% had no disease, with 13% of scans being inconclusive.[46] These data are similar to Ostrom et al[32] and the Michigan Advanced Cardiovascular Imaging Consortium findings. In this registry of over

4000 subjects very similar to PROMISE's target population, 40% had nonobstructive disease, 41% had normal anatomy, and only 14% had lesions $\geq$70%.[55] Finally, only 14% of the subjects in a multicenter CTA study, Assessment by Coronary Computed Tomographic Angiography of Individuals Undergoing Invasive Coronary Angiography (ACCURACY), had obstructive disease (defined as stenosis $\geq$70%) on cath.[16] Thus, current clinical data suggest that non-obstructive CAD will be frequent and obstructive CAD relatively uncommon in the PROMISE cohort.

Long-term outcomes in subjects with non-obstructive disease on angiography, such as was found in 39% of the NCDR cohort,[15] have been found to be intermediate between subjects with obstructive CAD or with no disease.[56,57] This is consistent with the known prognostic power of coronary calcium scoring,[57] and an observed 2x increase in death in patients with non-obstructive disease compared to those with normal coronary anatomy on CTA.[32,46] The knowledge that most myocardial infarctions arise from non-obstructive lesions[58,59] provides biologic plausibility to these observed differences in outcomes. Since functional testing relies upon inducing ischemia due to limited coronary flow reserve in the presence of obstructive lesions, it therefore cannot detect potentially important non-obstructive disease. Patients receiving these tests will have undetected non-obstructive disease. In contrast, non-obstructive disease is routinely detected by CTA, yielding information that may be important for properly adjusting the intensity of preventive therapy to reflect the presence of coronary plaque.

Therefore, in response to these needs, the "**PRO**spective **M**ulticenter **I**maging **S**tudy for **E**valuation of Chest Pain (PROMISE)" Trial, a multicenter, randomized pragmatic trial is comparing two state-of-the-art diagnostic strategies in symptomatic subjects at clinically determined, low-intermediate risk for CAD who require non-urgent testing. The investigational arm uses an "anatomic" testing strategy with coronary CTA ($\geq$64 slice) as the initial test. The usual care arm uses a "functional" stress testing strategy using physician-selected stress imaging (echocardiography or nuclear) or exercise electrocardiography as the initial test. Ten thousand subjects (10,000) will be randomized over approximately 24 months and followed for a minimum of 2 years (average 2.5 years). All subsequent diagnostic and therapeutic management decisions will be based on the latest clinical practice guideline recommendations and will be at the discretion of the treating care team.

B2 *Rationale: The Urgent Need for a Randomized Trial of Diagnostic Strategies in Subjects with Suspected CAD*

Millions of patients undergo different diagnostic testing for suspected CAD in the US every year despite little or no evidence that it will improve their outcomes. The remarkable imprecision and inefficiency of current evaluation strategies is perhaps best documented by the high rate (up to 60%) of invasive coronary angiograms in which no significant CAD is detected.[15] while the US economic burden of caring for patients with chest pain exceeds $75 billion/yr.[60] Limitations of current tests, an inadequate evidence base, and escalating costs have led all major professional societies, Centers for Medicare and Medicaid Services (CMS) and NIH to publicly demand the development of new evidence to optimize the clinical use of diagnostic testing, reduce unnecessary invasive procedures, and control costs. Further, all stakeholders have called for further investigation of the appropriate role of coronary CTA, a novel diagnostic imaging technology which may potentially change the standard of care in this common but challenging situation.[47,50,61-63]

The problem at hand must be addressed by an effectiveness trial, as examination of efficacy only (i.e., optimal performance at expert sites) would fail to capture the actual performance of diagnostic strategies in the community where the vast majority of these tests are performed and where future clinical decisions based on trial results will impact patient outcomes. The concept of a pragmatic clinical trial, as proposed by Tunis et al[64] is an ideal format to acquire the quality of evidence required to address the needs of clinical decision makers. The PROMISE study embodies all the characteristics of such a trial including: (1) incorporation of clinically relevant alternatives including usual clinical testing and usual clinical care; (2) a diverse study population; (3) heterogeneous practice settings; and (4) use of a broad range of health outcomes rather than focusing solely on cost. Each of these features is essential to establish an evidence-based standard-of-care for the large number of patients who require diagnostic testing for symptoms of CAD.

Thus, the ideal approach to addressing these problems is a randomized trial of different testing strategies that is adequately powered to demonstrate superior clinical outcomes, as only improved patient health would be sufficiently motivating to change practice and justify the routine use and reimbursement of a new technology. Because test reimbursement policies can be expected to evolve and change in response to many non-medical forces in the economy, conclusions based primarily on the differential costs of alternative testing strategies are unstable over the long-term. The ideal trial must also directly target the clinical outcomes that are most relevant to assessing value. Finally, the ideal trial must ensure the broadest possible applicability of its results by demonstrating real world effectiveness across the appropriate spectrum of tests, practice settings, and caregiver specialties and expertise, which are pertinent to the clinical decisions needed to care for the vast majority of chest pain patients nationwide. The PROMISE trial design has been shaped by these objectives.

C Study Overview and Objectives

C1 *Overview of the PROMISE Trial*

PROMISE is a multicenter, randomized pragmatic trial comparing two state-of-the-art diagnostic strategies in approximately 10,000 symptomatic, low to intermediate risk subjects with suspected CAD who require non-urgent testing. The investigational arm will use an "anatomic" testing strategy with coronary CTA ($\geq$64 slice) as the initial test. The usual care or "functional" testing strategy will use either stress imaging (echocardiography or nuclear) or exercise ECG as the initial test. All subsequent diagnostic and therapeutic management will be at the discretion of the treating care team. The trial will encourage adherence to evidence based practice and document actual therapies used, but will not mandate specific care plans which will be left to the discretion of the clinical care team. Subjects will be randomized over approximately 24 months and followed for 24-48 months at 200-250 North American primary care, cardiology and acute care practice sites, reflecting the physician specialties and community settings where the vast majority of chest pain patients receive care.

C2 *Primary Aim*

The primary aim of PROMISE is to determine if an initial anatomic testing strategy with CTA (≥ 64 slice; investigational care) in symptomatic subjects with low to intermediate risk for CAD will reduce the composite primary endpoint (all cause death, myocardial infarction, major peri-procedural complications and hospitalization for unstable angina) when compared to an initial functional testing strategy (stress echo, stress nuclear or exercise ECG; usual care), over an average of 2.5 years of follow-up (range approximately 2 to 4 years).

The primary endpoint is time to the first event in a composite of major cardiovascular events including:

- Death
- Myocardial infarction
- Major complications from cardiovascular procedures and testing (stroke, major bleeding, anaphylaxis, and renal failure requiring dialysis)
- Unstable angina hospitalization

C3 *Secondary Aims*

The Secondary Aims are to compare the following clinical and economic outcomes in subjects randomized to initial anatomic versus functional diagnostic testing:

- Death or myocardial infarction or unstable angina hospitalization
- Death or myocardial infarction
- Major complications from cardiovascular procedures and testing (stroke, major bleeding, anaphylaxis, and renal failure requiring dialysis)
- Medical costs, resource use, and incremental cost effectiveness
- Health related quality of life

In addition cumulative radiation exposure will be assessed as a secondary safety endpoint.

C4 *Rationale for the Selection of Outcome Measures*

Rationale for Clinical Assessments

Because patients with CAD remain at risk for major adverse events related to their disease over their lifetimes, consideration of long-term outcomes is essential to exploring the impact of test selection on health outcomes and costs. The endpoints of all cause death, non-fatal MI, major complications related to cardiovascular procedures and unstable angina hospitalizations are clinically relevant and together reflect the success or failure of the diagnostic testing strategy to accurately detect or exclude disease, guide procedural care and provide useful long term prognostic information with high confidence. Any event that could potentially be influenced by the anatomic information provided by CTA, such as PCI, is excluded as an endpoint regardless of when during the follow up period it occurred. Conversely, major peri-procedural complications are included as these are a potentially important cause of harm resulting from a need for an invasive study due to inaccurate initial diagnostic tests. The use of a composite clinical

endpoint and a subject follow-up of at least 2 years are essential to testing PROMISE's hypothesis of clinical superiority for CTA.

Rationale for Economic and Quality of Life Assessments

The use of noninvasive diagnostic testing for the assessment of CAD has grown at a far faster rate than other medical care, including related procedures such as cardiac catheterization or revascularization.[65] Cardiac diagnostic testing now costs over $4 billion annually for Medicare Part B alone.[66] Although these unsustainable testing costs mandate the development of better evidence, recent efforts by payers to reduce costs have largely addressed the number of tests performed, or payment per test, rather than the value derived for guiding patient care. Thus testing is increasingly driven by onerous administrative practices such as prior authorization designed to discourage physicians from providing indicated care,[67] or drastic reductions in reimbursement. This default approach is not in the best interests of either patients or our increasingly dysfunctional health care system.

The studies that have examined the costs and cost effectiveness of functional vs. anatomic testing strategies to date are limited by the lack of reliable long-term effectiveness and cost data.[24,43,68,69] Thus, a formal examination of the incremental cost of testing and its value for patients is urgently needed and is an essential part of the PROMISE trial.

C5 *Rationale for Selection of Testing in Each Experimental Arm*

Usual care or functional testing arm: Stress nuclear, stress echocardiography and exercise ECG. Each of these procedures is a well-established and accepted strategy to diagnose CAD. Each has also been in routine clinical use for over 20 years and is well supported by class I, level of evidence B ACC/AHA practice guideline recommendations for use in suspected CAD. The guidelines further document specific indications as well as diagnostic and prognostic performance. (See also Table 2.) A national claims database (United HealthCare) documenting contemporary US testing patterns shows that stress nuclear exams constitute 61% of tests, with stress echo at 18% and exercise ECG at 21%. CTA and stress MRI comprised <1% each. Seventy-nine percent of tests were performed with exercise stress[8] The lack of a single dominant diagnostic strategy for current practice, even in this insured population, highlights the essential need for flexibility and breadth in the choice of functional tests to fully reflect real-world diagnostic practices and maximize generalizability and clinical impact. Thus, it is essential that exercise ECG, stress echocardiography and stress nuclear imaging are all included in the usual care or functional testing arm.

Experimental or anatomic testing arm: CTA. Because CTA is quick, robust, readily available and accurate, it may lead to a major practice shift in the evaluation of chest pain where a strategy of imaging coronary anatomy is preferred over the current practice of first searching for inducible ischemia using functional testing. In this sense it's widespread adoption would represent substantial change as it may improve diagnostic testing in an unexpected way (through anatomic rather than functional information) and may alter the indications for testing, thereby expanding the patient population being tested. Further, there is evidence of increasing physician confidence over time,[25] a hallmark of an improved diagnostic testing strategy.[70] This growing experience indicates that the greatest strength of CTA may lie in excluding obstructive CAD in lower

prevalence cohorts—exactly the symptomatic population being studied in PROMISE.[71] Finally, because of its visual strength, CTA has an inherent important motivational ability which has been shown to improve prescription and adherence to effective preventive strategies, an important goal of any testing strategy.[72] On the other hand, CTA cannot be performed in all patients as calcified lesions and temporal and spatial resolution can be limiting. However, evidence suggests that CTA is superior to functional testing in reducing false positive and false negative test results, identifying non-obstructive disease, improving the prognostic accuracy of a negative test and extending the 'warranty period' of test results, all of which strongly suggest clinical superiority over functional testing.

D Investigational Plan

D1 *Overview of Trial Design*

PROMISE is a multicenter, randomized pragmatic trial comparing two state-of-the-art diagnostic strategies in approximately 10,000 symptomatic, low to intermediate CAD risk subjects with suspected CAD who require non-urgent testing. The following discussion includes trial assessments, outcomes, substudies, organization and operations, sites and site management, timeline and potential criticisms. See the figure below for a schematic summary of the trial design.

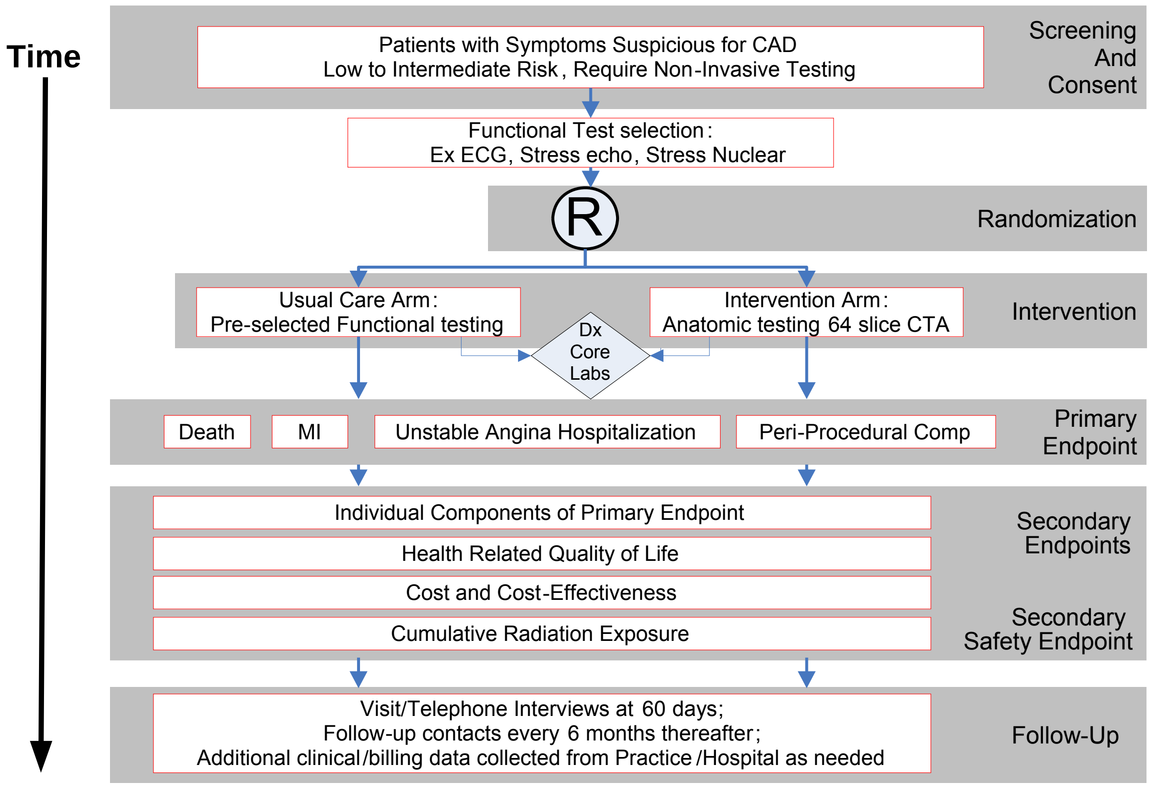

D2 *Subject Population*

To optimize the generalizability of the study results and reflect current patterns of care, subjects considered for enrollment into PROMISE will be outpatients without known CAD who are symptomatic, and whose physician has decided that the subject requires non-urgent, non-invasive cardiovascular testing to further evaluate suspected CAD. This population is purposefully and carefully chosen to be directly reflective of the population in which elective noninvasive cardiac diagnostic testing is currently being used.

PROMISE's inclusion criteria stipulate that all subjects will be symptomatic and will, in the judgment of the physician caring for the subject, require an elective noninvasive test for symptoms suspicious of CAD. Thus, whether or not the subject chooses to participate in the trial, he/she will likely undergo testing. All of the modalities in PROMISE are clinically well established and performed routinely and safely across the U.S. No experimental treatment or testing is involved. **The trial intervention is simply the random assignment of the initial test.**

Justification of population: Since subjects are symptomatic and have already been determined to require outpatient noninvasive testing, this is an ideal population in which to compare the results of different testing strategies. We include subjects presenting in primary care offices, cardiology offices and urgent care settings without suspected ACS to ensure inclusion of all subject profiles, however patients undergoing testing as part of a rule out ACS protocol are not eligible. To ensure broad generalizability we are using a patient-centric design and not limiting enrollment to a single care setting or single type of provider (cardiology). PROMISE reflects the care settings, patient groups and providers who will eventually apply its results.

2.a **Inclusion Criteria**

1. New or worsening chest pain syndrome or equivalent symptoms suspicious for clinically significant CAD
2. No prior evaluation for this episode of symptoms
3. Planned non-invasive testing for diagnosis
4. Men age ≥ 55 and women age ≥65 years
5. If age in men 45 - 54 or women 50 - 64 years, then must have increased probability of CAD due to EITHER:

    A. Diabetes Mellitus (DM) requiring medical treatment OR

    Peripheral Arterial Disease (PAD) defined as documented ≥50% peripheral arterial stenosis treated medically or invasively OR cerebrovascular disease (stroke, documented ≥ 50% carotid stenosis treated medically or invasively)

    OR

    B. At least one of the following cardiovascular risk factors:

    - Ongoing tobacco use
    - Hypertension
    - Abnormal ABI defined as less than <0.9
    - Dyslipidemia
6. Serum creatinine ≤ 1.5 mg/dL within the past 90 days

7. Negative urine/serum pregnancy test for female subjects of child-bearing potential

2.b Exclusion Criteria

1. Diagnosed or suspected ACS requiring hospitalization or urgent or emergent testing; Elevated troponin or CK-MB
2. Hemodynamically or clinically unstable condition (systolic BP < 90 mmHg, atrial or ventricular arrhythmias, or persistent resting chest pain felt to be ischemic despite adequate therapy)
3. Known CAD with prior MI, PCI, CABG or any angiographic evidence of CAD ≥50% lesion in a major epicardial vessel.
4. Any invasive coronary angiography or non-invasive anatomic or functional CV test for detection of CAD, including CTA and exercise ECG, within the previous twelve (12) months.
5. Known significant congenital, valvular (≥ moderate) or cardiomyopathic process (hypertrophic cardiomyopathy or reduced systolic left ventricular function (LVEF ≤ 40%)) which could explain cardiac symptoms.
6. Contraindication to undergoing a CTA, including but not limited to:
    a. Allergy to iodinated contrast agent
    b. Unable to receive beta blockers unless heart rate ≤ 65 beats per minute
    c. Pregnancy
7. Life expectancy < 2 years
8. Unable to provide written informed consent or participate in long–term follow-up

2.c Ethical Considerations

Human subjects' involvement and characteristics: All human adult subjects who meet inclusion criteria and who do not meet any of the exclusion criteria will be considered eligible for this trial. Subjects who are within vulnerable populations will be included at the discretion of the site Institutional Review Boards (IRBs).

2.d Subject Recruitment Plans and Consent Process

Diagnostic testing for the assessment of CAD symptoms is ordered by physicians of all specialties, is performed in multiple settings including physician offices, hospital outpatient departments and diagnostic testing facilities, and is interpreted by physicians of multiple specialties, including primary care physicians, cardiologists and radiologists. Therefore, subjects will be recruited from multiple clinical settings, including outpatient clinics, urgent care centers, and testing facilities. Sites that do not perform the full range of diagnostic testing, including CTA, will be paired with regional referral centers for echo, nuclear and/or CTA testing where subjects may undergo the necessary testing. Existing clinical referral and research networks will be used to identify and enroll subjects.

Subjects will be recruited from 200-250 clinical sites. All potential PROMISE sites will obtain IRB/EC approval of the protocol and the associated consent form, and any recruitment tools. Written informed consent will be obtained from each subject prior to any study procedures. Assessment of CAD risk will begin during screening to ensure

eligibility. It will include medical history, physical exam and laboratory testing for the presence/absence of major cardiac risk factors including blood pressure/hypertension, diabetes, cholesterol (LDL, HDL), smoking, family history, sedentary life style, obesity, cerebrovascular and peripheral arterial disease history and ankle-brachial index.

Subjects will be identified and enrolled in a step-wise process as follows:

1. All subjects being considered for outpatient non-invasive testing for the initial evaluation of symptoms suspicious for clinically significant obstructive CAD will be screened;
2. Subjects meeting inclusion criteria and not having any of the exclusion criteria will be approached to participate in the study and have the study explained to them;
3. Subjects agreeing to participate after having all their questions answered will be asked to document their agreement on the study-specific IRB-approved consent form with a signature..

2.e Subject Randomization

Eligible subjects who have given written informed consent and meet all inclusion and no exclusion criteria will be randomly assigned in equal proportions (1:1) to either the anatomic or functional diagnostic testing arm of the trial. A computer-generated permuted block randomization schedule with stratification by clinical site will be used in the trial. Prior to randomization, the managing caregiver, site investigator or authorized designee will be asked to indicate the functional test s/he would plan to use if the subject were randomized to the functional testing arm. This information will be tracked and used as another stratification factor in the randomization scheme to facilitate comparisons of anatomic vs. functional testing according to the type of functional test. Subject randomization will be accomplished by telephone through a centralized toll-free Interactive Voice Response System (IVRS).

If a subject is randomized but does not undergo the planned initial diagnostic test, that subject will still be followed and included in the intention-to-treat statistical comparisons according to their randomized treatment assignment.

2.f Risks and Benefits

Potential risks. Protocol-specific risks associated with the PROMISE trial are minimal. All PROMISE enrolled subjects will, by inclusion criteria, require noninvasive testing for their symptoms. Thus, all subjects will have compelling medical reasons for performance of the noninvasive test arm to which they are randomized. All care in PROMISE is part of routine clinical practice and, because the only intervention being performed in PROMISE is the random assignment of initial diagnostic test, safety considerations and adverse events are limited to complications arising from initial testing. Reporting of these safety events will be the responsibility of the site investigator.

Drawing Blood: Risks associated with drawing blood include momentary discomfort and/or bruising, infection, excess bleeding, clotting, or fainting.

Potential Loss of Confidentiality: In any clinical trial, there is a possible risk to subjects as to the potential loss of confidentiality. To prevent this from occurring, the Clinical Coordinating Center (CCC) and the Statistical and Data Coordinating Center (SDCC) at the Duke Clinical Research Institute (DCRI) have strict procedures in place to ensure that all study data are confidential and anonymized except as required for data collection by the Economics and Quality of Life Coordinating Center (EQOL CC) and the

DCRI Outcomes and Follow-up Group (DOFG). For all data received by DCRI, subjects will be identified only by unique code numbers. The link to these codes will be maintained at the SDCC. Trial records that identify subjects will be kept secure and confidential as required by law. Federal Privacy Regulations provide safeguards for privacy, security, and authorized access.

If the DOFG finds that a subject has undergone diagnostic testing, a diagnostic or therapeutic procedure or has been hospitalized, the DOFG will obtain the subject's test results or discharge summary, and the EQOL CC will obtain relevant medical billing information on behalf of the trial. Once received at the SDCC, these documents will be anonymized removing the subjects' name, personal identifiers and local physicians' name, and will be identified by unique study enrollment numbers.

Potential Benefits: The PROMISE trial results should improve the care of future subjects requiring diagnostic testing for suspected CAD. In addition, the trial will deliver high quality data on radiation exposure, incidental findings and other clinically important 'side effects' of the testing strategies which will be examined in a large real world experience.

D3 *Diagnostic Testing*

3.a Approach to Diagnostic Testing

An important goal of PROMISE is the broadest possible applicability of its results. Therefore, all commonly used tests are included in the 'usual care' arm, a designation which generally implies a multiplicity of approaches. This inclusiveness will facilitate enrollment by ensuring that all eligible subjects will have ready access to high quality testing in both arms. Because the preferred type of functional test will be recorded by the IVRS prior to randomization, the trial subjects can later be divided into three (3) cohorts for pre-specified subgroup analyses, each of which has been selected by their managing clinician to undergo a different form of functional testing. In each cohort, half will be randomized to undergo that pre-specified functional test, while the other half will be randomized to undergo CTA, forming paired CTA-functional test subgroups for each functional test type

We will ensure that the standard of care of testing and imaging performance and interpretation consistent with good medical practice is maintained throughout the trial by requiring that sites have expertise and qualify in all modalities, by providing recommended imaging protocols and by providing timely quality feedback and expert over-reading in a subset of studies, again for all modalities.

3.b Description of Testing To Be Performed

Subjects will initially be evaluated according to their physician's specific protocol for management of subjects with an indication for non-urgent, non-invasive cardiovascular testing. Typically, the standard evaluation will include the relevant past and current medical history, a physical examination and a resting 12-lead ECG, and may include cardiac biomarkers (troponin and/or CK-MB) as well as other routinely obtained blood testing. Subjects that meet eligibility criteria will have their physician's preferred functional test designated as part of the stratification requirements for randomization.

Usual Care Arm: For subjects randomized to usual care, the pre-selected functional test will be performed as the initial test. This includes either stress nuclear imaging, or stress echocardiography, or exercise ECG.

Intervention Arm: In subjects randomized to the intervention arm, a contrast enhanced coronary CTA will be performed as the initial test.

Subsequent care: The results of all tests will be provided to the care team in the usual manner for that testing laboratory, and, depending on the results, subjects may or may not undergo medical treatment and/or additional noninvasive functional, anatomic or invasive testing (coronary angiography), and/or coronary revascularization at the care team's discretion. It is assumed that patient management will be additionally informed and guided by the test findings. The local physician will resume care of the subject and make all subsequent clinical decisions (e.g., need for further evaluation or admission) based upon his/her cumulative clinical assessment of the subject, including findings revealed on the noninvasive testing.

3.c Functional and Anatomic Testing

Equipment and Protocols: Criteria for qualification of participating sites or referral laboratories will include use of standard equipment for usual care testing (stress echo, stress nuclear, and exercise ECG) as defined in current practice guidelines, and ≥64-slice MDCT technology for coronary CTA.[73-77] Similarly, all test acquisition protocols will adhere to best practice standards as defined in current national practice guidelines.[73-77] Sites will be allowed to use their own standard acquisition protocols as long as they fall within the standard of care guidelines. Sample protocols are provided in the Manual of Operations and Procedures (MOP).

Interpretation: All studies will be interpreted by qualified physicians who have at least ACC COCATS (Core Cardiology Training Symposium) level 2 training[78] or equivalent. For nuclear studies, certification by the Certification Board of Nuclear Cardiology (CBNC) or Board Certification in Nuclear Medicine or Radiology is also sufficient to qualify as a reader. Coronary CTA will be interpreted by physicians at least COCATS level 2 trained or equivalent either Society of Cardiovascular Computed Tomography level 2 or the Certification Board of Computed Cardiovascular Tomography (CBCCT). However, because coronary CTA is a relatively new technology and practice requirements and expertise have not evolved to the same level as other diagnostic tests, reader certification may include online review and evaluation of clinical cases consisting of paired coronary CTA and invasive angiography datasets.

All diagnostic tests will be interpreted and reported by diagnosticians at the sites in real time according to current guidelines to ensure timely availability of results for patient management. The diagnostic test reports will capture the major findings including:

1. Presence and extent of coronary artery disease (CTA).

2. Resting left ventricular function and perfusion (echo and nuclear scans) as either: normal, global dysfunction, regional dysfunction/scar, both or not interpretable. Ejection fraction will be quantified. This information will be optionally gathered for CTA.

3. Functional capacity will be determined for exercise ECG, stress echo, or stress nuclear.

3.d Test Transfer and Storage

Test transfer will be accomplished by transmission of paper recordings for stress ECGs and the ECG component of echo and nuclear studies. For digital images, transmission may be accomplished by CD or over the web. For web transmission, an image transfer system will be deployed at the site. The data transfer software will provide encryption, lossless compression, and transmission capabilities for the submission of de-identified

data sets via the web to the central Test Data Repository hosted by the American College of Radiology Imaging Network (ACRIN) in Philadelphia.

Upon arrival in the central data repository, datasets will be processed as needed: paper ECGs converted to pdfs, CDs uploaded and web images decompressed, de-encrypted and imported into the central imaging database, a networked long-term image archiving and storage system. This will ensure maximal site flexibility as well as seamless and efficient transfer of imaging data from sites into a central data repository, and will allow real-time quality control and feedback.

3.e Quality Assurance of Diagnostic Testing

Prior to beginning enrollment, eligible sites and readers will be certified by the Diagnostic Testing Coordinating Center (DTCC) based on site and reader surveys, on successful transfer of one or more complete data set(s) with sufficient image quality and completeness for each modality, and participation in the CTA case review. Final site certification will be issued by the DTCC for each modality prior to subject enrollment.

During the study, technical quality assessment of image and test acquisition will be accomplished on all studies by central repository research technicians trained by the DTCC. This ongoing review will ensure the adequate quality and completeness of data sets (see details and definitions in the MOP) and will monitor radiation exposure in the CTA arm throughout the trial.

Expert over reading of the initial, randomized noninvasive tests will be performed in approximately 20% of subjects by modality experts in the DTCC. To ensure protocol compliance early on and to account for low accrual sites, the first two studies and ~10% of the remaining studies from each site will be over read in each modality. These will be selected by block randomization stratified by testing site and test modality.

Feedback to the clinical sites, remediation and disqualification: Sites whose performance does not consistently meet the quality criteria for both QA and radiation exposure will be asked to undergo protocol review and retraining. Sites which fail to improve may be recommended for discontinuation of enrollment.

3.f Subsequent Medical Care

Care following the imaging studies will be provided by the local care physicians at their discretion. However, they will be encouraged to follow established guidelines for the management of CAD or for primary prevention. To this end, Test Information Sheets specific to each modality will be created and supplied to each enrolling site and MD and to relevant imaging site staff. These will consist of a brief literature review of diagnostic and prognostic indications of various test results.

In addition, primary and secondary prevention information sheets will be created and provided to each enrolling site and MD at the beginning of the trial. These will consist of brief summaries of relevant ACC/AHA Guidelines and will be referenced to the Test Information Sheets' results.

3.g Testing Risk and Benefits

To be eligible for entry into the study, each subject and his/her health care provider will have considered the risks and benefits of noninvasive testing and will have determined that the incremental information gained outweighs the potential risks. For example, although diagnostic testing provides important diagnostic and prognostic information and is noninvasive, it cannot be used indiscriminately. Associated risks include maximal exercise testing, use of pharmacologic stress and contrast agents and radiation exposure on the order of 7-17 mSv for stress nuclear and CTA (although aggressive dose reduction strategies can reduce this to ~2-6 mSv in CTA). CTA also often involves the use of beta blockers to lower heart rate and an angiographic contrast agent.[79-81] (For comparison, the radiation exposure from a single view chest X-ray is about 0.03 mSv.)

Because the long term risks and benefits of one form of testing versus another are unknown, the PROMISE trial is collecting all relevant information to address these knowledge deficiencies, including test complications and estimated total biological radiation exposure for cardiovascular procedures during the duration of the trial. For radiation, doses will be estimated whenever possible using the site's specific scanning protocols for diagnostic testing and fluoroscopic time for invasive procedures. However, if these are unavailable or deemed unreliable, modeled estimates from the sites with such data or from the relevant literature will be used. We will also record the presence of incidental findings as reported on all initial tests and follow-up procedures or testing performed to further evaluate any resulting new diagnoses of significant non-cardiac disease including surgical procedures.

E Study Procedures

E1 *Screening for Eligibility*

Screening of subjects for this trial will be conducted by the site investigator (or authorized designees) at each participating site. Written, informed consent documentation will be obtained from each prospective trial subject once study eligibility is confirmed and prior to the first study procedure.

E2 *Schedule of Assessments*

Subjects will be screened and randomized at or before time of enrollment. Baseline medical history, blood work, and quality of life will be assessed. Subsequently, subjects will have either a telephone call or clinic visit at 60-days for outcome evaluation and recording of any test complications. After that, subjects will be contacted by the DOFG at 6 months post randomization and at 6-month intervals for subsequent follow up assessments until death, withdrawal or the end of the trial.

Screening assessments are described below.

TABLE OF ASSESSMENTS	Screening/ Day 1	Day 1-30	Day 60	6 mos	12 mos	18 mos	24 mos	30 mos	36 mos	42 mos	48+ mos
Informed Consent	X										
Confidential Patient Information Form	X										
Medical History	X		X[1]	X[1]	X[1]	X[1]	X[1]	X[1]	X[1]	X[1]	X[1]
Concomitant Medications	X		X	X	X	X	X	X	X	X	X
CV Risk	X			X	X	X	X	X	X	X	X
Pregnancy Test[2]	X										
Creatinine[3]	X										
Resting 12-lead ECG[4]	X										
Quality of Life	X			X	X		X		X		X
Economics	X			X	X	X	X	X	X	X	X
Biomarker Banking	X										
Randomization	X										
Initial Diagnostic Test		X									
Clinical Assessment			X	X	X	X	X	X	X	X	X
Endpoint Assessments			X	X	X	X	X	X	X	X	X
Test Safety Assessment			X								

[1] During medical history review, if subjects have received an additional diagnostic test, a cardiovascular procedure or have been hospitalized since the last visit, additional data will be collected from the practice or institution with subject consent

[2] Pregnancy test required only for female subjects of child-bearing potential

[3] Creatinine blood draw required only for subjects without a recent normal value (within previous 90 days)

[4] Resting 12-lead ECG required if none available within past 30 days

Day 0/1 Screening/Randomization:

- The subject's relevant medical history (including concomitant medications) will be obtained
- CAD risk factors will be assessed including blood pressure/hypertension, peripheral arterial disease (ratio of arm to leg systolic blood pressures or formal ABI), cerebrovascular disease (carotid bruits), diabetes, cholesterol (LDL,HDL), smoking, family history, sedentary life style, obesity
- Factors increasing probability of CAD (including diabetes, PVD, and/or other CAD risk factors) will be documented
- Cardiac symptoms and chest pain descriptors will be assessed
- Eligible subjects will be asked to give consent for study participation
- If the subject is of childbearing potential, a pregnancy test will be performed
- Creatinine blood draw if there is no creatinine measurement within the previous 90 days

- Subjects will receive a 12-lead resting ECG unless one obtained within the past 30 days is available
- Subjects will be administered baseline Quality of Life questionnaires at the time of randomization
- A blood sample will be drawn on subjects participating in biomarker and/or genomic banking (See Section E8)
- The subject will be randomized to the functional test of Investigator or caring provider choice or CTA. (*Note: functional test of choice must be selected and documented prior to placing the call to randomize the subject.)*

Days 1-30:

- The functional diagnostic test or CTA will be performed according to the randomized assignment

Day 60 Post-Randomization Follow-up Assessment– site clinic visit or telephone call:

- Relevant interval medical history (including symptoms and concomitant medications) since last assessment will be obtained, including death, myocardial infarction, major complications from CV procedure or testing (stroke, major bleeding, anaphylaxis, renal failure), unstable angina hospitalization
- Test images and stress ECGs from the first test (and the randomized test, if different) will be uploaded to the DTCC. If applicable, the subject's first invasive coronary angiography report and images will be uploaded to the DTCC. All clinical test reports will be sent to the CCC at the DCRI.
- The results of any additional noninvasive tests or invasive catheterizations performed within the first 60 days will be collected
- Radiation exposure will be assessed
- Interval resource consumption including hospitalizations will be assessed
- Patient satisfaction will be assessed

Follow-up at 6 months post-randomization and every 6 months thereafter will be conducted by DOFG staff:

- Relevant interval medical history (including symptoms and concomitant medications) since last assessment will be obtained including death, myocardial infarction, major complications from study-related CV procedures (e.g. catheterization, PCI, CABG, CTA, stress echo, stress nuclear) or testing (stroke, major bleeding, anaphylaxis, renal failure), unstable angina hospitalization
- Interval radiation exposure will be assessed
- Interval resource consumption including hospitalizations will be assessed
- CV risk modification will be recorded
- Quality of Life questionnaires will be completed at 6 months and 12 months post-randomization and yearly thereafter

E3 *Safety*

3.a Data and Safety Monitoring Board

A Data and Safety Monitoring Board (DSMB) will be appointed by the NHLBI to monitor subject safety and to review performance of the protocol. A DSMB charter that outlines the operating guidelines for the committee and the procedures for the interim evaluations of study data will be developed by the NHLBI and agreed upon at the initial meeting of the DSMB. Reports will be prepared regularly by the DCRI as requested by the DSMB chair. Depending upon the operational plan established by the DSMB, the report might include recruitment and retention rates, interim analyses, primary and secondary endpoints, and other information as requested by the committee. After each meeting, the DSMB will make recommendations to the NHLBI and the trial leadership about the continuation of the study.

3.b Study Coordinating Center or FDA Notification by Investigator

No reporting (other than through the InFORM system) is required.

3.c Definitions of Safety Events and Reporting

Because the only intervention in the trial is the randomized assignment of the initial test to be performed in a symptomatic subject with clinically indicated testing, the only safety events arising from the study are related to the initial test. Mild safety events are considered as related to testing only up to 24 hours after the initial randomized test, and will be collected and reported by site personnel. The site investigator/designee will document the safety events listed below and occurring within 24 hours of initial testing, and report them in the InFORM system:

In contrast, those severe events/complications related to cardiovascular testing or cardiovascular procedures which are also trial endpoints (e.g. peri-procedural MI, major bleeding, renal failure, and anaphylaxis requiring circulatory or respiratory support) will be collected throughout the duration of the trial and will be considered to be related to testing or a procedure if occurring within 72 hours.

For CTA:

1. Mild contrast reaction such as rash and hives (Severe reactions including anaphylaxis or death are part of the primary endpoint)
2. Extravasation of contrast into the surrounding tissue of the extremity where the IV was placed and contrast administered
3. Hemodynamic instability, including symptomatic bradycardia or hypotension, due to the beta blockade or nitrates given for the CTA scan acquisition
4. Acute bronchospasm due to the beta blockade given for the CTA scan

For exercise testing during exercise ECG, stress echo or stress nuclear:

1. Exercise-induced hypotension with systolic BP fall > 20mmHg
2. Stress induced symptoms that do not resolve within 20 minutes
3. Rapid atrial fibrillation that does not slow or convert with treatment
4. Ventricular tachycardia
5. Hemodynamic instability defined as systolic BP <80 mmHg

6. Hospital admission not otherwise captured by the primary endpoint, including that precipitated by any symptomatic event (chest pain, dyspnea, etc), persistent or worsening ischemic ECG changes, any bradycardic or tachycardic arrhythmia or any hemodynamic changes (hyper- or hypo-tension)

For stress nuclear:

1. The above events for exercise testing
2. Any events potentially related to the use of vasodilators such as dypridamole or adenosine, including an anaphylactic reaction to contrast agent not requiring circulatory or respiratory support

For stress echo:

1. The above events for exercise testing
2. Stress induced wall motion abnormality that does not resolve within 20 minutes (despite treatment)
3. Any anaphylactic reaction to contrast agent not requiring circulatory or respiratory support

E4 ***Study Outcome Measurements and Ascertainment***

All cause mortality: All cause mortality is used rather than cardiac mortality to eliminate the need for possibly difficult adjudication of causes of death, especially given the relatively low mortality expected.

Myocardial infarction: defined as either 1) an abnormal cardiac biomarker level > institutional ULN (either troponin or CK-MB), and either ischemic discomfort lasting > 10 minutes or ECG changes indicative of ischemia or infarction, or 2) new abnormal Q waves consistent with infarction. Additionally *peri-procedural infarctions* are defined as >3x upper limit of normal for serum CK-MB for PCI and >5x upper limit of normal for CABG.

Unstable angina hospitalization: defined as 1) ischemic discomfort or equivalent symptoms requiring hospitalization within 48 hours of symptoms, 2) lasting ≥ 10 minutes at rest, or in an accelerating pattern, 3) accompanied by dynamic ST depression, ischemia on stress testing or significant epicardial coronary artery stenosis, and 4) which is considered to be myocardial ischemia upon final diagnosis.

Major complications from cardiovascular procedures and diagnostic testing which occur within 72 hours, defined as:

- Stroke is defined as an acute focal neurological deficit of sudden onset, not reversible within 24 hours, or that resolves in <24 hrs with clear evidence of a new stroke on cerebral imaging.
- Bleeding is defined as major based on one or more of the following:
    - Transfusion of ≥ 2 units heterologous packed red blood cells or whole blood
    - Decrease in hemoglobin level by ≥ 2.0 g/l
    - Need for re-operation or invasive intervention (*e.g.* evacuation of wound hematoma)

- o Bleeding at a critical anatomic site (intracranial, intraspinal, intraocular, retroperitoneal, intra-articular, pericardial, or intramuscular with compartment syndrome)

- Renal Failure is defined as new requirement for renal replacement therapy
- Anaphylaxis is defined as a severe contrast reaction requiring emergency respiratory and/or circulatory support

E5 *Independent Clinical Event Adjudication Committee*

An independent clinical event adjudication committee (CEC) will review and adjudicate all primary endpoint events in a blinded fashion based on the definitions presented above. The clinical secondary endpoint events and cumulative radiation exposure will not be adjudicated.

Primary outcome events will be documented through 60 days after randomization by the site Investigator or authorized designees. After that, the DCRI Outcomes and Follow-up Group (DOFG) will be responsible for documenting study events from 6 months through at least 48 months or the last date the subject participated in the study.

E6 *Quality of Life Assessments*

Baseline quality of life interviews will be administered to subjects by site personnel as soon as possible after consent, preferably before the subject's randomization. Completed questionnaires will be sent directly to the EQOL CC for data processing. Follow-up quality of life questionnaires will be administered to subjects via structured telephone interview by DOFG interviewers at 6 and 12 months post-randomization, and annually thereafter for up to 4 years following enrollment. Proxy Quality of Life questionnaires will be used when a subject has died in the follow-up interval or has become incapacitated, and will include items that can be reliably obtained from a relative, caretaker or medical record.

Content of Health-related Quality of Life Questionnaires

A battery of validated instruments will be used that build on a disease specific core supplemented with generic measures to provide a comprehensive but brief assessment of health-related quality of life.

Chest-pain-specific symptoms will be measured using the **Seattle Angina Questionnaire (SAQ)**, a 19-item instrument that assesses 5 dimensions of the impact of chest pain on QOL: physical limitations, angina stability, angina frequency, treatment satisfaction, and disease perception. The **Duke Activity Status Index (DASI)**, a 12-item scale that has been validated in cardiac patients against maximal oxygen uptake measured at exercise (VO_2 max) will be used as a disease-specific functional status assessment. The 4-item **Rose Dyspnea Scale** will be used to assess patients' level of dyspnea with common activities.

The generic core instrument to be used is the **Medical Outcomes Study Short Form (SF-12)**. The SF-12 is composed of 8 scales (physical function, role function-physical, role function-emotion, general health, bodily pain, social function, psychological well-being/mental health, and vitality), a health transitions item, and two summary scores. Additionally, the entire scales for general health, psychological well being, vitality, and social functioning from the SF-36 will be used to provide better resolution of any treatment differences in these domains.

To assess effects of the two diagnostic strategies on the prevalence of depression, we will employ the **Patient Health Questionnaire (PHQ)**, a 9-item depression scale that has demonstrated good agreement with the clinical diagnosis of depression.

Patient-specific utilities will be assessed using the **EuroQoL-5D (EQ-5D)**, a standardized generic health status measure that links specific health states to general population-based utilities. The EQ-5D consists of a 5-dimension health state assessment, which allows for definition of 243 discrete health states that can be mapped to population utility weights, and a self-rating (0-100) "thermometer" of current health-related quality of life. The EQ-5D will be collected as part of all quality of life questionnaires.

Employment/productivity will include time lost from work and reduced productivity while at work as measured by the 6-item **Stanford Presenteeism Scale** and questions adapted from the NHLBI Bypass Angioplasty Revascularization Investigation Substudy in Economics and Quality of Life.

E7 *Economic Assessments*

Resource use data to be collected on the study CRF will include hospitalizations, ED visits, selected cardiac procedures and tests. Hospital bills (detailed, summary ledger and UB 04) will be collected by the EQOL CC at the DCRI for all hospitalizations identified throughout the length of the study. They will include care at clinical sites and at institutions not participating in PROMISE. In addition, cost to charge ratios (RCCs) will be obtained from each hospital where a PROMISE follow-up hospitalization is reported.

E8 *PROMISE Biorepositories*

8.a Imaging and ECGs

Since future developments in image interpretation and integration with clinical data will remain important in the diagnosis and management of chronic diseases of CAD, PROMISE will create a unique anonymized ‘Image and ECG Data Warehouse’ with all initial noninvasive test images, ECG and initial catheterization films which will be linked to clinical information. Collection and release of data and images will be at the discretion of the NHLBI and is included as part of the subjects’ initial consent.

8.b Blood Biomarkers and Genomics

The PROMISE Investigators also believe that an integrated approach to disease characterization and therapeutic responses will play an increasingly important role in the diagnosis and management of chronic diseases such as CAD. Accordingly, all subjects will be asked to provide a blood sample for deposit into a biomarker repository at the time of randomization for future assessment of advanced molecular biomarkers (plasma, serum) such as troponin and hsCRP relevant to disease characterization, risk stratification, characterization of treatment response and adverse effects. In addition, subjects will be requested to separately consent to allow use of the biorepository sample for genomic testing (RNA, DNA).

F Statistical Plan

F1 *Sample Size Determination and Statistical Power*

Several design factors and research objectives were considered in selecting the target sample size for the study. First, the number of subjects was determined so there would be a sufficient number of endpoints to provide a high degree of power ($\geq$ 90%) for testing the primary superiority hypothesis. Second, the statistical power for secondary endpoints was considered, including the composite clinical endpoints and the economic and quality of life endpoints. Third, it was considered important for the sample size to be large enough to permit a prudent examination of diagnostic testing effects in selected subgroups of subjects where anatomic testing might be particularly advantageous, or where the question of a benefit from CTA is particularly relevant. Important pre-specified subgroups of interest in this study include those defined by age, sex, race, comorbidity, cardiovascular risk factors, the pre-randomization choice of functional test, and characteristics of the precipitating symptoms. Fourth, the sample size was selected to provide a reasonable level of confidence for detecting clinically important outcome differences between the anatomic and functional testing strategies even if current projections of event rates and the hypothesized differences in clinical outcomes between the two arms prove to be optimistic. A fifth consideration was the adequacy of the sample size for assessing non-inferiority of anatomic testing compared to functional testing in the event that the anatomic testing strategy is not demonstrated to be statistically superior to functional testing. Finally, although the study objectives are expressed in terms of testing specific hypotheses (i.e., that the anatomic testing strategy is superior (and if not superior, then non-inferior) to functional testing, another important objective of the trial is to estimate the magnitude of the difference in outcomes to within an acceptable level of statistical precision, regardless of whether either testing strategy is proven to be superior. Thus, the precision of the estimated difference in outcomes between the two arms of the trial (i.e., width of the confidence interval) has been considered in addition to the statistical power for the hypothesis tests.

Based on the distribution of coronary disease expected in this patient population (approximately 15% obstructive disease (i.e., $\geq$50% stenosis of the left main coronary artery or $\geq$70% stenosis of one or more of the other major epicardial coronary arteries), 40% non-obstructive disease, and 45% normal coronary arteries), and based on national claims data or published information from other databases, the event rate at 2.5 years (the average length of follow-up in PROMISE) for the primary composite endpoint in subjects randomized to the functional testing strategy was projected to be approximately 9%.

With this event-rate projection in the functional testing arm, a key driver of the sample size is the magnitude of benefit that can reasonably be expected to be achieved with the anatomic testing strategy. This determination requires careful consideration of multiple characteristics of CTA, including its likely incremental diagnostic and prognostic accuracy, and its ability to more effectively detect non-obstructive CAD. A careful assessment of the impact of the advantages of CTA with respect to these test characteristics translates to a projection (hypothesis) that CTA will reduce the primary composite endpoint by 20% (from 9% to 7.2% at 2.5 years).

Based on the event rates for each arm discussed above, sample size requirements were formulated to provide high power for detecting the postulated 20% relative risk reduction. Recognizing, however, that the actual event rates and the outcome differences between

the two testing strategies in PROMISE may vary somewhat from these estimates, sample size requirements were calculated for several different combinations of event rates, effect sizes, and power levels in order to examine the sensitivity of the sample size to different event rates and outcome scenarios that might conceivably arise in this trial.

Since the primary treatment comparisons in this study will be based on time-to-event methodology using the log-rank test[82] or equivalently, the Cox proportional hazards model,[83] the approach used for calculating sample size requirements for PROMISE was based on the sample size methodology for the proportional hazards regression model outlined in Schoenfeld.[84]

To provide an adequate number of subjects for the trial that will be relatively robust in providing (1) excellent statistical power under various assumptions about the event rates in the functional testing arm and the magnitude of the benefit of anatomic testing compared to functional testing for reducing the primary endpoint, (2) adequate power for selected secondary endpoints, (3) adequate power for assessing non-inferiority in the event that superiority is not demonstrated, and (4) a relatively high degree of precision for estimating the true effect of an anatomic vs. functional testing strategy regardless of whether the final result has a p-value that is statistically significant, the study will enroll 10,000 subjects (5,000 per arm). This number will provide >90% power for detecting a 20% reduction in the primary clinical endpoint if the 2.5-year event rate in the functional testing arm is 8% or higher and 80% power if the event rate is as low as 6%. This number of patients will also provide adequate power for detecting a smaller (16-17%) reduction if the event rate in the functional testing arm is 8-9% or higher and acceptable power for selected secondary endpoints, allowing for up to a 3% loss to follow-up. Ten thousand patients will also provide 90% power for testing non-inferiority with a margin of 1.10 (expressed as a hazard ratio of CTA vs. functional testing) if the functional testing arm event rate is 9%, 86% power if the event rate is 8%, and 81% power if the event rate is 7%, assuming that anatomic testing is only better than functional testing by 10%, an assumption that was felt to be reasonable for the non-inferiority assessment. That is, we will have excellent power for demonstrating that anatomic testing is not worse than functional testing by more than 10% under these various assumptions.

In summary, 10,000 patients will provide excellent and robust statistical power for assessing clinically relevant and realistic outcome differences between the two testing strategies being studied in this trial.

F2 ***Statistical Analysis Plan***

Statistical analysis will be performed at the PROMISE Statistical and Data Coordinating Center (SDCC) at DCRI. All major treatment comparisons between the randomized groups will be performed according to the principle of "intention-to-treat;" that is, subjects will be analyzed (and endpoints attributed) according to the diagnostic testing strategy to which subjects were randomized, regardless of subsequent additional testing or post-randomization treatment and medical care. Statistical comparisons will be performed using two-sided significance tests. Additional perspective regarding the interpretation of the data will be provided through extensive use of confidence intervals[85] and graphical displays.

2.a Analysis for the Primary Endpoint

The statistical comparison of the two randomized arms (anatomic versus functional diagnostic testing) with respect to the primary composite endpoint (death, myocardial infarction, major peri-procedural complications, or hospitalization for unstable angina) will be a “time-to event” analysis, and therefore will be based on the time from randomization to the first occurrence of any of the components of the primary composite endpoint. The Cox proportional hazards model will be the primary analytic tool for assessing outcome differences between the two randomized arms.[83] To appropriately account for heterogeneity among the subjects, the overall comparison will be adjusted for a selected set of prognostically important baseline covariates that will be carefully defined and pre-specified in the statistical analysis plan. The level of significance for the assessment of the primary endpoint will be $\alpha=0.05$.

In addition to the statistical hypothesis testing, Kaplan-Meier ‘survival’ (or event-free) estimates[86] will be calculated for each randomized arm as a function of follow-up time to display the event rates graphically. A hazard ratio and 95% confidence interval for descriptively summarizing the difference in outcome between the two study arms will be computed using the Cox model.[83]

If the data provide evidence of an overall difference in outcome between the randomized arms, an assessment will be made of whether the effect is similar for all patients, or whether it varies according to specific patient characteristics. In particular, this analysis will focus on whether the relative benefit differs according to subject age, sex, race, co-morbidity, selected risk factors, characteristics of the precipitating symptoms, and the pre-randomization specification of the functional test that would be used if the subject was randomized to the functional testing arm. This latter factor is built into the randomization scheme. For subjects where the pre-randomization choice for functional testing (if the subject was assigned to the functional testing arm) was stress nuclear imaging (for example), the outcomes of these subjects in the CTA arm will be compared to the outcomes of corresponding subjects randomized to functional testing. In this way, CTA can be compared to stress nuclear imaging, as well as to each of the other functional testing modalities with the benefits of randomization in each comparison maintained by virtue of the stratified randomization scheme. These analyses will utilize the Cox model by testing for interactions between the randomized testing strategy and these specific baseline variables. In addition to the formal assessment of testing strategy by covariate interactions, effects of the diagnostic testing strategy characterized by a hazard ratio and 95% confidence interval will be calculated and displayed for prospectively-defined subgroups of subjects defined by the variables listed above. These descriptive hazard ratios will be carefully interpreted in conjunction with the formal interaction tests.

If the data do not provide statistical evidence that the CTA testing strategy is superior to functional testing with respect to the primary endpoint, a test for non-inferiority of the anatomic testing strategy will be performed. This assessment will be based on a non-inferiority margin of 1.10 (expressed as a hazard ratio for CTA vs. functional testing). The non-inferiority assessment will be performed by comparing the upper limit of the 95% confidence interval for the hazard ratio with the non-inferiority margin. If the upper limit of the confidence interval falls below 1.10, non-inferiority will have been demonstrated. We emphasize that the superiority hypothesis will be assessed first, and if significant, the non-inferiority assessment will not be performed. Only if superiority is not demonstrated will the non-inferiority analysis then be performed.

2.b Analysis of Secondary Endpoints

Secondary endpoints that will be evaluated include (1) a composite endpoint consisting of death, myocardial infarction, or hospitalization for unstable angina; (2) death or myocardial infarction; (3) major peri-procedural complications (stroke, major bleeding, renal failure, anaphylaxis); (4) resource use patterns, medical care costs and incremental cost effectiveness; and (5) quality of life. (6) In addition, major adverse cardiac events (events other than the endpoints listed above) will be monitored and reported.

The analysis of secondary endpoints 1 through 3 will be similar to that outlined for the superiority assessment of the primary endpoint, using time from randomization until the first occurrence of any component of the specific secondary endpoint (or censoring) as the response variable, and assessing group differences using the Cox proportional hazards model. The effect of the diagnostic testing strategy on these secondary clinical endpoints will be descriptively summarized using hazard ratios (with associated confidence intervals) computed from the Cox model. Kaplan-Meier curves will be computed to graphically display the cumulative event rates of the two randomized arms as a function of time from randomization. We note that the analysis of secondary endpoint (3) above (major peri-procedural complications) will have to be interpreted cautiously, particularly if there should be a higher death rate in one arm compared to the other. To deal with this complexity and clarify and enhance the interpretation of this comparison, the analysis of this endpoint will be supplemented with further analyses by considering major peri-procedural complications and death as a combined endpoint.

Plans for the analysis of the quality of life and economic endpoints are addressed below in Sections F2.g and F2.h

2.c Analysis of Diagnostic Testing Core Data

The DTCC will implement robust quality assurance (QA) programs to ensure uniformity and high quality testing in support of the primary aims of PROMISE.

Two important components of information from the diagnostic test QA activity will be analyzed by the SDCC. The DTCC will review every subject's initial diagnostic test for technical quality, and each test will be assigned a quality assessment using an ordinal categorical scale (for example, excellent, good, fair, poor, uninterpretable). Using simple frequency counts, the SDCC will tabulate a description of the distribution of this scale on an ongoing basis by type of test and by testing site in order to provide feedback to the sites on their performance, to inform the study leadership and the DSMB as to the quality of the diagnostic testing, and to flag potential problem areas, whether by testing site or across a given testing modality, for remedial attention. This quality measure will be compared among the different testing modalities using rank-based tests (e.g., Kruskal-Wallis nonparametric analysis of variance) and ordinal logistic regression to characterize, describe, and assess any differences in overall quality among the various testing modalities. In particular, the technical quality of the anatomic (CTA) tests will be compared with the quality of the functional tests. This comparison may be helpful in interpreting the comparisons of the two randomized arms with respect to the clinical outcomes.

The second important component of information from the diagnostic test QA activity is the quality of test interpretations. Approximately 20% of tests will be over-read by the DTCC using a categorical level of coronary disease risk. The site interpretation of level

of risk will be compared with the level of risk using the same scale assessed by the core lab's over-read of the test, and overall summary statistics of the agreement between the site and core lab assessments will be computed. Raw proportions of agreement (perfect agreement, and agreement differing by $\leq$ 1 category of risk) will be tabulated, and Kappa statistics will be used to characterize the level of agreement. The Kappa statistics are "chance corrected" (i.e., adjusted for agreement due to chance), and both unweighted and weighted Kappa statistics will be used in these analyses since more substantial disagreements in the assessment of risk for a given subject would be more serious than small disagreements. These agreement statistics will then be compared across the different testing modalities to determine whether the level of agreement between the site readings and the core lab readings varies with the type of test.

2.d Analysis of Diagnostic Accuracy

Although PROMISE is designed with the primary objective of evaluating initial anatomic vs. functional testing strategies with respect to clinical outcomes and thus represents a different paradigm than the traditional design to assess diagnostic accuracy, performance of supplementary analyses of diagnostic accuracy are prespecified. This cannot be done in a conventional manner as not all subjects will undergo invasive angiography (the "gold standard"), and those that do will not undergo angiography by random selection. Instead, the decision to verify disease will be based on test results and other characteristics of the subject. This non-random selection process will likely result in a strong verification bias,[87] often characterized by higher than true sensitivity and lower than true specificity results. (Of note, the trial can not require angiography in a subset of subjects for purposes of determining testing accuracy, as this would provide additional information to these subjects' physicians and care givers above and beyond that provided by the randomized testing strategy, and therefore invalidate the trial results). Correction of the verification bias is possible if the process leading to verification with angiography is known. However, this is rarely achievable, but the process can be modeled under the Missing at Random (MAR) assumption,[88] namely that disease status affects referral to angiography only through measured covariates, and not the disease status itself. In view of the inherent limitations, the following steps will be taken with respect to evaluating diagnostic accuracy:

Accuracy rates will be assessed using conventional measures (sensitivity, specificity, and Receiver Operating Characteristic (ROC) curves) in subjects undergoing cardiac catheterization, bringing to bear where applicable the latest statistical methods for dealing with verification bias. The probability of verification will be modeled with a logistic regression model using covariates that are predictors of referral to angiography. Corrected values of sensitivity, specificity and the ROC curve will be estimated.[88,89] Assessment of the impact of potential departures from the MAR assumption on sensitivity and specificity will utilize the "test ignorance region" approach[88]. Alternatively, we will use the clinical outcome as the "gold standard" (rather than coronary angiography), such that measures of diagnostic accuracy will be computed, including time-dependent ROC curves, to describe and statistically compare the two arms of the trial with respect to these measures. A subgroup analysis of diagnostic accuracy will be performed comparing results at sites with high volume and extensive experience or expertise in diagnostic testing to results in less experienced or lower volume sites.

Imaging prognostic performance will also be evaluated by comparing the association of test results to subsequent clinical events, including the primary endpoint of the trial. Finally as described in Section F2.c above, concordance between the site interpretations of studies and core lab interpretations will be assessed.

We emphasize that all of these analyses of diagnostic accuracy will be strictly supplementary to the analysis of the primary and secondary clinical endpoints outlined in Sections F2.a and F2.b above.

2.e Analysis of Safety Events, Morbidity, Radiation Dose, and Incidental Findings

The frequency with which major safety cardiac events occur (events other than the primary and secondary endpoints) will be carefully tabulated and descriptively summarized. Statistical comparisons of the randomized arms with respect to these events will use chi-square or other appropriate two-sample methods depending on the nature of the event, interpreting such comparisons in the context of differences between the two randomized arms in the primary and major secondary clinical endpoints and bringing to bear clinical judgment as to the relative seriousness of these events.

Of particular interest in this trial is the amount of radiation exposure to which subjects are subjected in each of the randomized arms of the trial. Radiation exposure for the various tests will be collected as elements of the eCRF and through the documentation of radiation exposure submitted to the DTCC. All cardiac diagnostic testing involving radiation exposure performed during the entire follow-up period (e.g., CTA, stress nuclear, cardiac catheterization) will be captured so that cumulative radiation exposure for each patient can be calculated. The distribution of radiation exposure in each arm will be summarized (using medians and percentiles) and compared between the randomized arms and different testing modalities using the Wilcoxon rank-sum test.

Incidental findings (e.g., such things as lung nodules) that may be discovered with the anatomic (CTA) testing strategy in contrast to the functional testing modalities will be captured as part of the data collection and tabulated descriptively.

2.f Assessment of Prognostic Factors

With the large database of information that will be collected on the 10,000 subjects enrolled in this study, extensive regression modeling analyses will be performed, using primarily the Cox regression model, to identify and assess the factors (predictors) that are associated with the clinical outcomes of these subjects. These analyses will comprehensively evaluate the strength and shape of the relationships of numerous clinical factors with the clinical outcomes. While these analyses are more exploratory than the rigorous pre-specified primary and secondary comparisons of the randomized arms of the trial, they will nonetheless be helpful in elucidating relationships and identifying the key factors that impact patient outcomes and any observed differences in outcomes between the diagnostic testing arms.

2.g Quality of Life (QOL) Analyses

For each of the QOL measures, data analysis will proceed in several stages. We will start by providing simple descriptive and comparative analyses by intention-to-treat. A nonparametric bootstrap will be used to estimate treatment differences with 95% confidence intervals (CI) and p-values. Because there is currently no consensus in the statistical literature about the best way to deal with the multiple comparisons problem arising from testing each individual scale at each time point separately, we propose two complementary approaches. First, we will pre-specify the angina frequency and disease perception/QOL scales from the SAQ as the CAD-specific measures of primary interest,

since these measures most directly quantify the therapeutic goal of coronary diagnosis and therapy: to minimize symptoms and optimize patients' QOL. We will also specify cardiac functional status measured with the Duke Activity Status Index (DASI) as a primary outcome measure of interest. Other disease-specific and generic QOL measures will be assigned to a secondary (descriptive) status in our analyses. Second, we will fit a mixed effect longitudinal proportional odds model[90] that makes use of all available QOL data at each study assessment point to model the time profile (fixed effect) using a restricted cubic spline function. Using the fitted model, we can estimate the overall difference in the QOL measures as well as test the global hypothesis of no difference over time. We can also estimate the difference in the areas under the two QOL treatment curves (and test the hypothesis of no difference on average). In addition, we can estimate differences in QOL at the end of the study or at intermediate points such as at 1 year. Statistical power estimates for this part of our analysis show that we should have in excess of 90% power to detect ¼ standard deviation differences in the 3 principal QOL endpoints.

2.h **Economic Analyses**

The health economic analyses for PROMISE will consist of two major parts, namely an empirical intention to treat cost comparison, and a cost effectiveness analysis. Primary statistical comparisons between the two treatment arms of empirical costs will be performed by intention-to-treat analysis. Confidence limits around the observed cost differences will be constructed using bootstrap methods.

The cost-effectiveness analyses will estimate the incremental cost required to add an extra life year with the investigational anatomic arm relative to the control functional testing arm. In secondary analyses, we will incorporate utility weights to estimate the incremental cost per quality adjusted life year gained with the CTA anatomic strategy, relative to the functional testing strategy. These analyses will be conducted from a societal perspective and will use a lifetime time horizon so that the estimated incremental cost-effectiveness and cost-utility ratios can be compared with societal benchmarks. We will also calculate within-trial cost-effectiveness/cost-utility ratios, although these ratios are limited in their value due to their failure to account for long-term benefits and costs and the absence of comparative benchmarks. Costs will be adjusted for inflation and both costs and life expectancy will be discounted to present value at a 3% annual discount rate. Adjustments for censored data due to staggered entry will be made following the approach of Bang and Tsiatis.[91] Extensive sensitivity analyses will be performed.

F3 *Interim Analyses*

For safety and ethical reasons, interim examinations of key safety and endpoint data will be performed at regular intervals during the course of the trial. The primary objective of these analyses will be to evaluate the accumulating data for an unacceptably high frequency of negative clinical outcomes in either of the two randomized arms. In addition, the interim monitoring will also involve a review of subject recruitment, compliance with the study protocol, status of data collection, and other factors, which reflect the overall progress and integrity of the study. The results of the interim analyses and status reports will be carefully and confidentially reviewed by the NHLBI-appointed DSMB. The DSMB will meet at approximately 6-month intervals to review the accumulating data.

To properly account for the repeated interim testing in PROMISE, a group sequential method similar to that proposed by O'Brien and Fleming[92] will be used as a guide for interpreting the interim analyses. This procedure requires large critical values early in the study, but relaxes (i.e., decreases) the critical value as the trial progresses. Because of the conservatism early in the trial, the critical value at the final analysis is near the "nominal" critical value. The actual method for this interim monitoring that will be employed in PROMISE is the “spending function” approach to group sequential testing developed by Lan and DeMets.[93] The Lan-DeMets approach only requires specification of the rate at which the Type I error (which in this trial will be α=0.05 for the primary endpoint) will be "spent". This procedure allows "spending" a portion of α at each interim analysis in such a way that at the end of the study, the total Type I error does not exceed 0.05. One such spending function generates boundaries that are nearly identical to the O'Brien-Fleming[92] boundaries. It is this approach that will be used in PROMISE, namely two-sided, symmetric O'Brien-Fleming[92] type boundaries generated using the flexible Lan-DeMets[93] approach to group sequential testing. Since the number of looks and the increments between looks need not be predetermined, it allows considerable flexibility in the monitoring process for accommodating additional comparative examinations of the data in response to concerns of the DSMB that may arise during the course of the trial.

The analytic approach that will be used at the interim analyses for assessing outcome differences between the randomized arms will be the time-to-event analysis methods described previously, except that interpretation of statistical significance will be guided using the group sequential monitoring boundaries outlined above. At each interim analysis, the monitoring boundaries will be calculated using the fraction of the total number of primary events expected by the end of the trial.

Judgment concerning the continuation or termination of the study will involve not only the degree of statistical significance observed at the interim analyses, but also careful consideration of many other factors reflecting the progress and integrity of the trial, including how well event rates in the functional testing arm are tracking with the rates considered in the power calculations. As an aid in the interim assessments, the SDCC will supplement the group sequential analyses outlined above with calculations of conditional power, namely the conditional probability that a particular statistical comparison will or will not be significant at the end of the trial at the α level used in the design, given the hypothesized outcome difference between the randomized arms and the data observed to date. After a sufficient number of primary events have occurred (approximately 50% of the total projected events), conditional power for the primary treatment comparison will be computed and provided to the DSMB as a regular part of the interim study reports.

It should be emphasized that regardless of whether CTA demonstrates a statistically significant benefit compared to functional testing, the results of the study will be important to future clinical practice. Indeed, a non-statistically significant effect for the primary endpoint is not necessarily a negative result in this study. The range of experience and expertise of the DSMB will enable them to put all important considerations into proper perspective, including the precision of estimates of clinical outcome differences between the testing strategies, and make well-reasoned recommendations to the NHLBI regarding continuation of the study.

G Data Handling and Record Keeping

Study Data Collection -- All Subjects

The full study dataset will be collected for subjects who enter the randomized phase of the study. The primary data collection system for PROMISE will use the electronic data capture (EDC) system, InForm™.

Data Management and Quality

Any out-of-range values and missing or inconsistent key variables are flagged and addressed/answered at the site in real time during the data entry process. When a query is generated on a particular variable, a flag is set in a field in the database enabling the system to track the queries and produce reports of outstanding queries. Queries can also be generated from manual review of the data forms. These queries will be entered into the database and tracked in the same manner as the computer-generated queries. At regular intervals, all data will be transferred from InForm™ to SAS for statistical summarization, data description, and data analysis. Further cross-checking of the data will be performed in SAS, and discrepant observations flagged and appropriately resolved through a data query system.

The Statistical and Data Coordinating Center will perform internal database quality-control checks, and data audits throughout the course of the trial.

G1 *Confidentiality and Security*

All study data will be stored in locked, secure locations. Computerized data are accessible only by password, and a centralized monitoring system records and reports all access to data. The DCRI computer network is protected by a firewall. Electronic CRFs (eCRFs) will be identified by study number only, to insure subject anonymity. No subject identifiers will be used in the presentation of data. Study records that might identify subjects will be kept confidential as required by law. Except when required by law, subjects will not be identified by name, social security number, address, telephone number, or any other direct personal identifier in study records. This information will be retained by each individual center and will not be disclosed to the coordinating center except as needed for DCRI centralized clinical, quality of life and economic follow-up of the subjects. Subjects will be informed that the study physician and his/her study team will report the results of study-related tests to the Coordinating Center and to the NIH. Subjects will be informed that their records may be reviewed in order to meet federal or state regulations. Reviewers may include the FDA, IRBs/ECs, or the NIH. Subjects will be informed that if their research record is reviewed, their entire medical record may also need to be reviewed. If an adverse event occurs, management of the event and subsequent care will be according to appropriate care practices at that site and will be implemented under the direction of the treating physician. All of the tests used in this study currently represent a reasonable standard of care for the subject population as demonstrated by ACC/AHA practice standards.

G2 *Training*

All investigator staff authorized to enter PROMISE Study data will receive training on the InForm™ system.

G3 *Electronic Case Report Form (eCRF)*

This study will use web-based electronic CRFs (e-CRFs) developed through a validated, Electronic Record, Electronic Signatures (ERES) compliant platform (21 CFR Part 11). Data will be entered into the InForm™ eCRF by authorized Investigator personnel, the American College of Radiology Imaging Network (ACRIN) and the diagnostic testing core labs.

G4 *Records Retention*

Study records will be maintained by the site investigators for a period of three (3) years following the expiration of the grant or length of time as required by local regulations.

H Study Monitoring, Auditing, and Inspecting

H1 *Study Monitoring Plan*

See E3.a

H2 *Auditing and Inspecting*

NHLBI personnel or their designees may perform an audit at any time during or after completion of this study. All original study-related documentation will be made available to the designated auditor as required. A representative of the NIH or other government agency may choose to inspect a study center at any time prior to, during, or after completion of the clinical study. All pertinent original study data will be made available to responsible regulatory authorities for verification, audit, or inspection purposes.

I Study Administration

I1 *Organization and Participating Centers*

The PROMISE trial will be conducted under the following PI leadership: Dr. Pamela Douglas as the overall and CCC PI, Dr. Kerry Lee as the SDCC PI, Dr. Daniel Mark as the EQOL CC PI, and Dr. Udo Hoffmann at Massachusetts General Hopsital as the DTCC PI. The organization of the **PROMISE** trial and inter-relationships between the NHLBI, the CCC, the SDCC, the various committees, and the clinical sites are outlined in the Figure below.

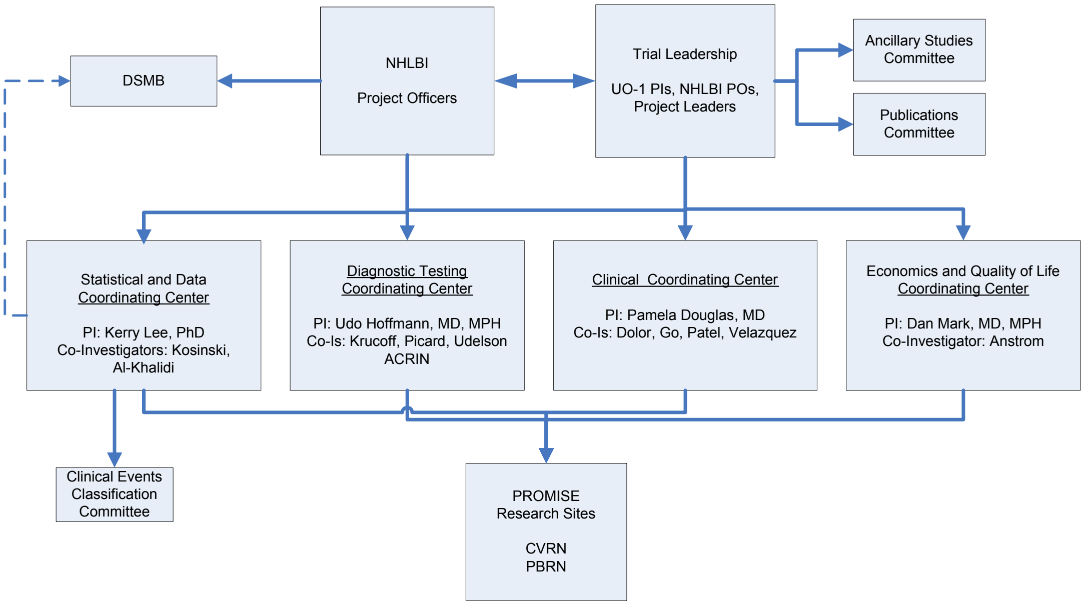

I2 *Funding Source*

The PROMISE trial is funded by the National Heart, Lung, and Blood Institute, National Institutes of Health.

I3 *Study Timetable*

The PROMISE timeline includes a 6-month start up period, followed by approximately 24 months of enrollment, approximately 24 months of follow up and 6 months of close out and data analysis.

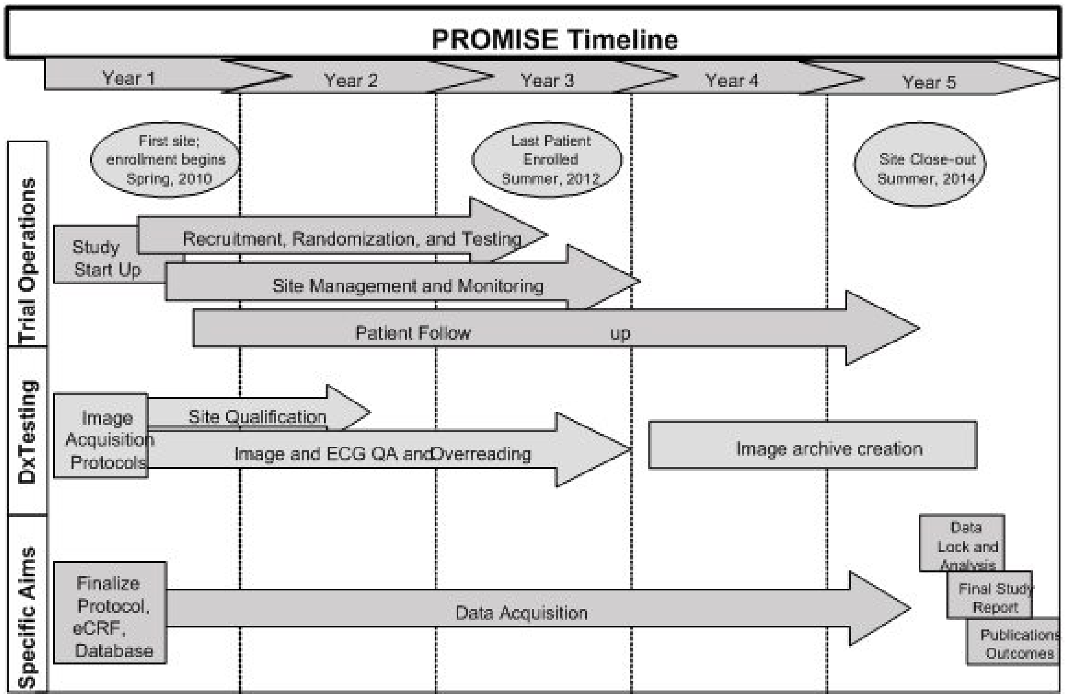

J References

1. American Heart Association. Heart disease and stroke statistics-2008 update http://circ.ahajournals.org/cgi/content/full/117/4/e25 (Accessed December 23, 2009).

2. Selker HP, Zalenski RJ, Antman EM, et al. An evaluation of technologies for identifying acute cardiac ischemia in the emergency department: a report from a National Heart Attack Alert Program Working Group. Ann Emerg Med 1997;29:13-87.

3. Hemingway H, McCallum A, Shipley M, Manderbacka K, Martikainen P, Keskimaki I. Incidence and prognostic implications of stable angina pectoris among women and men. JAMA 2006;295:1404-11.

4. Daly CA, De Stavola B, Sendon JL, et al. Predicting prognosis in stable angina--results from the Euro heart survey of stable angina: prospective observational study. BMJ 2006;332:262-7.

5. Sekhri N, Feder GS, Junghans C, et al. Incremental prognostic value of the exercise electrocardiogram in the initial assessment of patients with suspected angina: cohort study. BMJ 2008;337:a2240.

6. Clayton TC, Lubsen J, Pocock SJ, et al. Risk score for predicting death, myocardial infarction, and stroke in patients with stable angina, based on a large randomised trial cohort of patients. BMJ 2005;331:869.

7. A Report from the NCCSDO: Are rapid access chest pain clinics effective and fair? http://www.sdo.nihr.ac.uk/files/project/32-final-report.pdf (Accessed December 23, 2009).

8. Mudrick DW, Shah BR, Cowper P, et al. Downstream Care and Outcomes Following Outpatient Stress Testing in the United States: How Low is Too Low Risk? Circulation 2009;120:S393.

9. Sekhri N, Feder GS, Junghans C, Hemingway H, Timmis AD. How effective are rapid access chest pain clinics? Prognosis of incident angina and non-cardiac chest pain in 8762 consecutive patients. Heart 2007;93:458-63.

10. Daly CA, Clemens F, Sendon JL, et al. The clinical characteristics and investigations planned in patients with stable angina presenting to cardiologists in Europe: from the Euro Heart Survey of Stable Angina. Eur Heart J 2005;26:996-1010.

11. Cheitlin MD, Armstrong WF, Aurigemma GP, et al. ACC/AHA/ASE 2003 Guideline update for the clinical application of echocardiography--summary article: a report of the American College of Cardiology/American Heart Association Task Force on Practice Guidelines (ACC/AHA/ASE Committee to Update the 1997 Guidelines for the Clinical Application of Echocardiography). J Am Coll Cardiol 2003;42:954-70.

12. Gibbons RJ, Balady GJ, Bricker JT, et al. ACC/AHA 2002 Guideline update for exercise testing: summary article: a report of the American College of Cardiology/American Heart Association Task Force on Practice Guidelines (Committee to Update the 1997 Exercise Testing Guidelines). Circulation 2002;106:1883-92.

13. Klocke FJ, Baird MG, Lorell BH, et al. ACC/AHA/ASNC Guidelines for the clinical use of cardiac radionuclide imaging--executive summary: a report of the American College of Cardiology/American Heart Association Task Force on Practice Guidelines (ACC/AHA/ASNC Committee to Revise the 1995 Guidelines for the Clinical Use of Cardiac Radionuclide Imaging). J Am Coll Cardiol 2003;42:1318-33.

14. Pope JH, Aufderheide TP, Ruthazer R, et al. Missed diagnoses of acute cardiac ischemia in the emergency department. N Engl J Med 2000;342:1163-70.

15. Patel MR, Peterson ED, Dai D, et al. Low diagnostic yield of elective coronary angiography. New England Journal of Medicine 2010;362:886-95.

16. Budoff MJ, Dowe D, Jollis JG, et al. Diagnostic performance of 64-multidetector row coronary computed tomographic angiography for evaluation of coronary artery stenosis in individuals without known coronary artery disease: results from the prospective multicenter ACCURACY (Assessment by Coronary Computed Tomographic Angiography of Individuals Undergoing Invasive Coronary Angiography) trial. J Am Coll Cardiol 2008;52:1724-32.

17. Miller JM, Rochitte CE, Dewey M, et al. Diagnostic performance of coronary angiography by 64-Row CT. N Engl J Med 2008;359:2324-2336.

18. Stein PD, Yaekoub AY, Matta F, Sostman HD. 64-slice CT for diagnosis of coronary artery disease: a systematic review. Am J Med 2008;121:715-25.

19. Mowatt G, Cook JA, Hillis GS, et al. 64-slice computed tomography angiography in the diagnosis and assessment of coronary artery disease: systematic review and meta-analysis. Heart 2008;94:1386-93.

20. LaRosa JC, He J, Vupputuri S. Effect of statins on risk of coronary disease: a meta-analysis of randomized controlled trials. JAMA 1999;282:2340-6.

21. Fleischmann KE, Hunink MG, Kuntz KM, Douglas PS. Exercise echocardiography or exercise SPECT imaging? A meta-analysis of diagnostic test performance. JAMA 1998;280:913-20.

22. Schuijf JD, Poldermans D, Shaw LJ, et al. Diagnostic and prognostic value of non-invasive imaging in known or suspected coronary artery disease. European Journal of Medicine Molecular Imaging:93-104.

23. Garber AM, Solomon NA. Cost-effectiveness of alternative test strategies for the diagnosis of coronary artery disease. Ann Intern Med 1999;130:719-28.

24. Institute for Clinical and Economic Review. Coronary computed tomographic angiography for detection of coronary artery disease. http://www.icer-review.org/index.php/ccta.html (Accessed December 23, 2009).

25. Chow B, Abraham A, Wells G, et al. Diagnostic accuracy and impact of computed tomographic coronary angiography on utilization of invasive coronary angiography. CircCardiovasc Imaging 2009;2:16-23.

26. Marcassa C, Bax JJ, Bengel F, et al. Clinical value, cost-effectiveness, and safety of myocardial perfusion scintigraphy: a position statement. Eur Heart J 2008;29:557-63.

27. Mahenthiran J, Bangalore S, Yao SS, Chaudhry FA. Comparison of prognostic value of stress echocardiography versus stress electrocardiography in patients with suspected coronary artery disease. Am J Cardiol 2005;96:628-34.

28. Yao SS, Qureshi E, Sherrid MV, Chaudhry FA. Practical applications in stress echocardiography: risk stratification and prognosis in patients with known or suspected ischemic heart disease. J Am Coll Cardiol 2003;42:1084-90.

29. Hachamovitch R, Hayes S, Friedman JD, et al. Determinants of risk and its temporal variation in patients with normal stress myocardial perfusion scans: what is the warranty period of a normal scan? J Am Coll Cardiol 2003;41:1329-40.

30. Metz LD, Beattie M, Hom R, Redberg RF, Grady D, Fleischmann KE. The prognostic value of normal exercise myocardial perfusion imaging and exercise echocardiography: a meta-analysis. J Am Coll Cardiol 2007;49:227-37.

31. Min JK, Shaw LJ, Devereux RB, et al. Prognostic value of multidetector coronary computed tomographic angiography for prediction of all-cause mortality. J Am Coll Cardiol 2007;50:1161-70.

32. Ostrom MP, Gopal A, Ahmadi N, et al. Mortality incidence and the severity of coronary atherosclerosis assessed by computed tomography angiography. J Am Coll Cardiol 2008;52:1335-43.

33. Hachamovitch R, Berman DS, Kiat H, et al. Exercise myocardial perfusion SPECT in patients without known coronary artery disease: incremental prognostic value and use in risk stratification. Circulation 1996;93:905-14.

34. Berman DS, Kang X, Hayes SW, et al. Adenosine myocardial perfusion single-photon emission computed tomography in women compared with men. Impact of diabetes mellitus on incremental prognostic value and effect on patient management. J Am Coll Cardiol 2003;41:1125-33.

35. Galassi AR, Azzarelli S, Tomaselli A, et al. Incremental prognostic value of technetium-99m-tetrofosmin exercise myocardial perfusion imaging for predicting outcomes in patients with suspected or known coronary artery disease. Am J Cardiol 2001;88:101-6.

36. Hachamovitch R, Berman DS, Shaw LJ, et al. Incremental prognostic value of myocardial perfusion single photon emission computed tomography for the prediction of cardiac death: differential stratification for risk of cardiac death and myocardial infarction. Circulation 1998;97:535-43.

37. Picano E, Severi S, Michelassi C, et al. Prognostic importance of dipyridamole-echocardiography test in coronary artery disease. Circulation 1989;80:450-7.

38. Poldermans D, Fioretti PM, Boersma E, et al. Dobutamine-atropine stress echocardiography and clinical data for predicting late cardiac events in patients with suspected coronary artery disease. Am J Med 1994;97:119-25.

39. Cortigiani L, Dodi C, Paolini EA, Bernardi D, Bruno G, Nannini E. Prognostic value of pharmacological stress echocardiography in women with chest pain and unknown coronary artery disease. J Am Coll Cardiol 1998;32:1975-81.

40. Weiner DA, Ryan TJ, McCabe CH, et al. Prognostic importance of a clinical profile and exercise test in medically treated patients with coronary artery disease. J Am Coll Cardiol 1984;3:772-9.

41. Mark DB, Hlatky MA, Harrell FE, Jr., Lee KL, Califf RM, Pryor DB. Exercise treadmill score for predicting prognosis in coronary artery disease. Ann Intern Med 1987;106:793-800.

42. Mark DB, Shaw L, Harrell FE, Jr., et al. Prognostic value of a treadmill exercise score in outpatients with suspected coronary artery disease. N Engl J Med 1991;325:849-53.

43. Min JK, Shaw LJ, Berman DS, Gilmore A, Kang N. Costs and clinical outcomes in individuals without known coronary artery disease undergoing coronary computed tomographic angiography from an analysis of Medicare category III transaction codes. Am J Cardiol 2008;102:672-8.

44. Min JK, Kang N, Shaw LJ, et al. Costs and clinical outcomes after coronary multidetector CT angiography in patients without known coronary artery disease: comparison to myocardial perfusion SPECT. Radiology 2008;249:62-70.

45. Koenig W, Bamberg F, Lee H, et al. High-sensitivity troponin reliably excludes acute coronary syndrome in patients with acute chest pain: Results from the rule out myocardial infarction by computed tomography (ROMICAT) Study. Circulation S637.

46. Hoffmann U, Moselewski F, Nieman K, et al. Noninvasive assessment of plaque morphology and composition in culprit and stable lesions in acute coronary syndrome and stable lesions in stable angina by multidetector computed tomography. J Am Coll Cardiol 2006;47:1655-62.

47. Shah BR, Patel MR, Peterson ED, Douglas PS. Defining optimal research study design for cardiovascular imaging using computed tomography angiography as a model. Am J Cardiol 2008;102:943-8.

48. Hachamovitch R, Di Carli MF. Methods and limitations of assessing new noninvasive tests: part I: Anatomy-based validation of noninvasive testing. Circulation 2008;117:2684-90.

49. Douglas PS. Improving imaging: our professional imperative. J Am Coll Cardiol 2006;48:2152-5.

50. Douglas PS, Taylor AJ, Bild DE, et al. Outcomes Research in Cardiovascular Imaging: Report of a workshop sponsored by the National Heart, Lung and Blood Institute. J Am Col Card: Cardiovascular Imaging 2009;(in press).

51. Bonow RO. Sixth Annual Mario S. Verani, MD Memorial Lecture: Cardiovascular imaging--added value or added cost? J Nucl Cardiol 2008;15:170-7.

52. Douglas PS, Khandheria B, Stainback RF, et al. ACCF/ASE/ACEP/AHA/ASNC/SCAI/SCCT/SCMR 2008 Appropriateness criteria for stress echocardiography: a report of the American College of Cardiology Foundation Appropriateness Criteria Task Force, American Society of Echocardiography, American College of Emergency Physicians, American Heart Association, American Society of Nuclear Cardiology, Society for Cardiovascular Angiography and Interventions, Society of Cardiovascular Computed Tomography, and Society for Cardiovascular Magnetic Resonance endorsed by the Heart Rhythm Society and the Society of Critical Care Medicine. J Am Coll Cardiol 2008;51:1127-47.

53. Brindis RG, Douglas PS, Hendel RC, et al. ACCF/ASNC Appropriateness criteria for single-photon emission computed tomography myocardial perfusion imaging (SPECT MPI): a report of the American College of Cardiology Foundation Quality Strategic Directions Committee Appropriateness Criteria Working Group and the American Society of Nuclear Cardiology endorsed by the American Heart Association. J Am Coll Cardiol 2005;46:1587-605.

54. Fryback DG, Thornbury JR. The efficacy of diagnostic imaging. Med Decis Making 1991;11:88-94.

55. G Raff. personal communication.

56. Bairey Merz CN, Shaw LJ, Reis SE, et al. Insights from the NHLBI-Sponsored Women's Ischemia Syndrome Evaluation (WISE) Study: Part II: gender differences in presentation, diagnosis, and outcome with regard to gender-based pathophysiology of atherosclerosis and macrovascular and microvascular coronary disease. J Am Coll Cardiol 2006;47:S21-9.

57. Detrano R, Guerci AD, Carr JJ, et al. Coronary calcium as a predictor of coronary events in four racial or ethnic groups. N Engl J Med 2008;358:1336-45.

58. Falk E, Shah PK, Fuster V. Coronary plaque disruption. Circulation 1995;92:657-71.

59. Kern MJ, Meier B. Evaluation of the culprit plaque and the physiological significance of coronary atherosclerotic narrowings. Circulation 2001;103:3142-9.

60. Javitz HS, Ward MM, Watson JB, Jaana M. Cost of illness of chronic angina. Am J Manag Care 2004;10:S358-69.

61. Douglas P, Iskandrian AE, Krumholz HM, et al. Achieving Quality in Cardiovascular Imaging: Proceedings from the American College of Cardiology-Duke University Medical Center Think Tank on quality in cardiovascular imaging. J Am Coll Cardiol 2006;48:2141-51.

62. Douglas P, Chen J, Gillam L, et al. Achieving Quality in Cardiovascular Imaging II: Proceedings from the Second American College of Cardiology - Duke University Medical Center Think Tank on quality in cardiovascular imaging. J Am Col Card: Cardiovascular Imaging 2009;2:231-240.

63. Lauer MS. CT Angiography: First things first. Circ Cardiovasc Imaging 2009;2:1-3.

64. Tunis SR, Stryer DB, Clancy CM. Practical clinical trials: increasing the value of clinical research for decision making in clinical and health policy. JAMA 2003;290:1624-32.

65. Lucas FL, DeLorenzo MA, Siewers AE, Wennberg DE. Temporal trends in the utilization of diagnostic testing and treatments for cardiovascular disease in the United States, 1993-2001. Circulation 2006;113:374-9.

66. Government Accountability Office. Medicare Part B imaging services: Rapid spending growth and shift to physician offices indicate need for CMS to consider additional management practices. www.gao.gov/new.items/d08452.pdf (Accessed December 23, 2009).

67. Hendel R. Utilization management of cardiovascular imaging: Pre-certification and appropriateness. J Am Col Card: Cardiovascular Imaging 2008:1(2);241-248.

68. Sharples L, Hughes V, Crean A, et al. Cost-effectiveness of functional cardiac testing in the diagnosis and management of coronary artery disease: a randomised controlled trial. The CECaT trial. Health Technol Assess 2007;11:iii-iv, ix-115.

69. Ladapo JA, Hoffmann U, Bamberg F, et al. Cost-effectiveness of coronary MDCT in the triage of patients with acute chest pain. Am J Roentgenol 2008;191:455-63.

70. Hunink MG, Krestin GP. Study design for concurrent development, assessment, and implementation of new diagnostic imaging technology. Radiology 2002;222:604-14.

71. Meijboom WB, van Mieghem CA, Mollet NR, et al. 64-slice computed tomography coronary angiography in patients with high, intermediate, or low pretest probability of significant coronary artery disease. J Am Coll Cardiol 2007;50:1469-75.

72. Taylor AJ. Finding the pathway to improving patient outcomes with atherosclerosis imaging: who's motivated? Am Heart J 2007;154:1008-10.

73. Gibbons RJ, Balady GJ, Bricker JT, et al. ACC/AHA 2002 guideline update for exercise testing: summary article. A report of the American College of Cardiology/American Heart Association Task Force on Practice Guidelines (Committee to Update the 1997 Exercise Testing Guidelines). Journal of the American College of Cardiology 2002;40:1531-40.

74. Pellikka PA, Nagueh SF, Elhendy AA, Kuehl CA, Sawada SG. American Society of Echocardiography recommendations for performance, interpretation, and application of stress echocardiography. Journal of the American Society of Echocardiography 2007;20:1021-41.

75. Ficaro EP, Hansen CL, et.al. American Society of Nuclear Cardiology Imaging Guidelines for Nuclear Cardiology Procedures Journal of Nuclear Cardiology 2007 Nov/Dec:61-78.

76. Abbara S, Arbab-Zadeh A, Callister TQ, et al. SCCT guidelines for performance of coronary computed tomographic angiography: a report of the Society of Cardiovascular Computed Tomography Guidelines Committee. J Cardiovasc Comput Tomogr 2009;3:190-204.

77. Raff GL, Abidov A, Achenbach S, et al. SCCT guidelines for the interpretation and reporting of coronary computed tomographic angiography. J Cardiovasc Comput Tomogr 2009;3:122-36.

78. Beller GA, Bonow RO, Fuster V. ACCF 2008 Recommendations for Training in Adult Cardiovascular Medicine Core Cardiology Training (COCATS 3) (revision of the 2002 COCATS Training Statement). Journal of the American College of Cardiology 2008;51:335-8.

79. Ficaro EP, Zanzonico P, Stabin MG, et al. Variability in radiation dose estimates from nuclear and computed tomography diagnostic imaging. Journal of Nuclear Cardiology 2009;16:161.

80. Bedetti G, Botto N, Andreassi MG, Traino C, Vano E, Picano E. Cumulative patient effective dose in cardiology. Br J Radiol 2008;81:699-705.

81. Einstein AJ, Henzlova MJ, Rajagopalan S. Estimating risk of cancer associated with radiation exposure from 64-slice computed tomography coronary angiography. JAMA 2007;298:317-23.

82. Kalbfleisch JD, Prentice RL. The Statistical Analysis of Failure Time Data, Second Edition. New York, NY: John Wiley & Sons, Inc., 2002.

83. Cox D. Regression models and life-tables (with discussion). J Royal Statist Soc B 1972:187-220.

84. Schoenfeld DA. Sample-size formula for the proportional-hazards regression model. Biometrics 1983;39:499-503.

85. Borenstein M. The case for confidence intervals in controlled clinical trials. Controlled Clinical Trials 1994;15:411-28.

86. Kaplan EL, Meier P. Nonparametric estimation from incomplete observations. J Am Statist Assn 1958:457-481.

87. Begg CB, Greenes RA. Assessment of diagnostic tests when disease verification is subject to selection bias. Biometrics 1983;39:207-15.

88. Kosinski AS, Barnhart HX. A global sensitivity analysis of performance of a medical diagnostic test when verification bias is present. Statistics in Medicine 2003;22:2711 - 2721.

89. Alonzo TA, Pepe MS. Assessing accuracy of a continuous screening test in the presence of verification bias. Applied Statistics 2005;54:173-190.

90. Hedeker D, Gibbons RD. A random-effects ordinal regression model for multilevel analysis. Biometrics 1994;50:933-44.

91. Bang H, Tsiatis AA. Median regression with censored cost data. Biometrics 2002;58:643-9.

92. O'Brien PC, Fleming TR. A multiple testing procedure for clinical trials. Biometrics 1979;35:549-56.

93. Lan KKG, DeMets DL. Discrete sequential boundaries for clinical trials. Biometrika 1983;70:659-663.

The **PROMISE** Trial FINAL Protocol

PROspective **M**ulticenter **I**maging **S**tudy for **E**valuation of Chest Pain

**Sponsor:**	National Heart Lung and Blood Institute (NHLBI)
**Clinical Coordinating Center**	
**Principal Investigator:**	Pamela S. Douglas, MD
**Co-Investigators:**	Rowena Dolor, MD<br>Alan Go, MD<br>Daniel Mudrick, MD<br>Manesh R. Patel, MD<br>Eric J. Velazquez, MD
**Statistical/Data Coordinating Center**	
**Principal Investigator:**	Kerry L. Lee, PhD
**Co-Investigators:**	Andrzej S. Kosinski, PhD<br>Hussein R. Al-Khalidi, PhD
**Diagnostic Testing Coordinating Center**	
**Principal Investigator:**	Udo Hoffmann, MD, MPH
**Co-Investigators:**	Mitchell Krucoff, MD<br>Michael Picard, MD<br>James Udelson, MD
**Economics/Quality of Life**	
**Principal Investigator:**	Daniel B. Mark, MD, MPH
**Co-Investigator:**	Kevin Anstrom, PhD
**Investigation of:**	Diagnostic testing strategies for suspected coronary artery disease (CAD), including use of coronary computed tomographic angiography (CTA)
**FINAL Date:**	**April 12, 2010**
**Amended:**	**February 22, 2011**
**Administrative Change:**	

CONFIDENTIAL

This document is confidential and the property of Duke Clinical Research Institute (DCRI). No part of it may be transmitted, reproduced, published, or used by other persons without prior written authorization from the DCRI. This document may be disclosed to the appropriate institutional ethics committees/institutional review boards or to duly authorized representatives of the U.S. Food and Drug Administration or other regulatory authority under the condition that they maintain confidentiality.

Investigator Protocol Signature Page

I have read and understand the protocol and agree that it contains all the ethical, legal, and scientific information necessary to conduct this study. I will personally conduct the study as described. I will provide copies of the protocol to all physicians, nurses, and other professional personnel responsible to me who will participate in the study. I am aware that this protocol must be approved by the Institutional Review Board or Ethics Committee. I agree to adhere strictly to the attached protocol. I agree that clinical data entered on case report forms by me and my staff will be supplied to the Duke Clinical Research Institute (DCRI) and may be utilized by the DCRI in various ways, such as for submission to governmental regulatory authorities and/or in combination with clinical data gathered from other research sites, whenever applicable. I agree to allow DCRI monitors and auditors full access to all medical records at the research facility for subjects screened or randomized in the study. I agree to provide all subjects with informed consent forms, as required by government regulations and International Conference on Harmonisation guidelines.

Version Date: Amendment 1

______________________________	______________________________
**Principal Investigator (print name)**	**Site Name and Number**
______________________________	______________________________
**Principal Investigator (signature)**	**Date**

Protocol Version and Amendment Tracking

Version Number/Amendment	Approval Date
1.0	April 12, 2010
Amendment 1	February 22, 2011

TABLE OF CONTENTS

A INTRODUCTION ... 24

A1 STUDY SYNOPSIS ... 24
A2 PRIMARY HYPOTHESIS ... 25
A3 SIGNIFICANCE OF THE STUDY ... 26

B BACKGROUND ... 27

B1 PRIOR LITERATURE AND STUDIES ... 27
1.a Current Management of Patients with Chest Pain: Extent of the Problem ... 27
1.b Current Diagnostic Strategies Using Functional Tests are Inadequate ... 27
1.c Computed Tomographic Angiography is Highly Accurate in Detecting and Excluding Obstructive and Nonobstructive Coronary Artery Disease ... 28
1.d Additional Important Considerations in Comparing Test Characteristics ... 29
B2 RATIONALE: THE URGENT NEED FOR A RANDOMIZED TRIAL OF DIAGNOSTIC STRATEGIES IN SUBJECTS WITH SUSPECTED CORONARY ARTERY DISEASE ... 30

C STUDY OVERVIEW AND OBJECTIVES ... 31

C1 OVERVIEW OF THE PROMISE TRIAL ... 31
C2 PRIMARY AIM ... 32
C3 SECONDARY AIMS ... 32
C4 RATIONALE FOR THE SELECTION OF OUTCOME MEASURES ... 32
C5 RATIONALE FOR SELECTION OF TESTING IN EACH EXPERIMENTAL ARM ... 33

D INVESTIGATIONAL PLAN ... 35

D1 OVERVIEW OF TRIAL DESIGN ... 35
D2 SUBJECT POPULATION ... 35
2.a Inclusion Criteria ... 36
2.b Exclusion Criteria ... 36
2.c Ethical Considerations ... 37
2.d Subject Recruitment Plans and Consent Process ... 37
2.e Subject Randomization ... 38
2.f Risks and Benefits ... 38
D3 DIAGNOSTIC TESTING ... 39
3.a Approach to Diagnostic Testing ... 39
3.b Description of Testing To Be Performed ... 40
3.c Functional and Anatomic Testing ... 40
3.d Test Transfer and Storage ... 41
3.e Quality Assurance of Diagnostic Testing ... 41
3.f Subsequent Medical Care ... 42
3.g Testing Risk and Benefits ... 42

E STUDY PROCEDURES ... 43

E1 SCREENING FOR ELIGIBILITY ... 43
E2 SCHEDULE OF ASSESSMENTS ... 43
E3 SAFETY ... 46
3.a Data and Safety Monitoring Board ... 46
3.b Study Coordinating Center or Food and Drug Administration Notification by Investigator ... 46
3.c Definitions of Safety Events and Reporting ... 46
E4 STUDY OUTCOME MEASUREMENTS AND ASCERTAINMENT ... 47
E5 INDEPENDENT CLINICAL EVENT ADJUDICATION COMMITTEE ... 49
E6 QUALITY-OF-LIFE ASSESSMENTS ... 49

E7 ECONOMIC ASSESSMENTS 50
E8 PROMISE BIOREPOSITORIES 50
8.a Imaging and Electrocardiograms 50
8.b Blood Biomarkers and Genomics 50

F STATISTICAL PLAN 51

F1 SAMPLE SIZE DETERMINATION AND STATISTICAL POWER 51
F2 STATISTICAL ANALYSIS PLAN 52
2.a Analysis for the Primary Endpoint 53
2.b Analysis of Secondary Endpoints 54
2.c Analysis of Diagnostic Testing Core Data 54
2.d Analysis of Diagnostic Accuracy 55
2.e Analysis of Safety Events, Morbidity, Radiation Dose, and Incidental Findings 56
2.f Assessment of Prognostic Factors 56
2.g Quality-of-life Analyses 56
2.h Economic Analyses 57
F3 INTERIM ANALYSES 57

G DATA HANDLING AND RECORD KEEPING 59

G1 CONFIDENTIALITY AND SECURITY 59
G2 TRAINING 60
G3 ELECTRONIC CASE REPORT FORM 60
G4 RECORDS RETENTION 60

H STUDY MONITORING, AUDITING, AND INSPECTING 61

H1 STUDY MONITORING PLAN 61
H2 AUDITING AND INSPECTING 61

I STUDY ADMINISTRATION 62

I1 ORGANIZATION AND PARTICIPATING CENTERS 62
I2 FUNDING SOURCE 62
I3 STUDY TIMETABLE 62

J REFERENCES 64

Table of Revisions to NHLBI PROMISE Protocol, Version 1.0

Section	Original	Revision	Rationale
Title page; Investigator Protocol Signature Page; Protocol Version and Amendment Tracking page	version number: 1.0<br>date: April 12, 2010	version number: Amendment 1<br>date: February 22, 2011	Self-evident
A1—Primary Study Objective	To determine whether an initial non-invasive anatomic imaging strategy with coronary CT angiography (CTA) will improve clinical outcomes in subjects with symptoms concerning for coronary artery disease relative to an initial functional testing strategy (usual care).	To determine whether an initial noninvasive anatomic imaging strategy with coronary computed tomographic angiography (CTA) will improve clinical outcomes in subjects with symptoms concerning for CAD relative to an initial functional testing strategy.	Remove "usual care" and retain "functional testing" as more commonly used terminology; consistency.
A1—Study Follow-up	The sites will conduct the initial study follow up at 60 days. Follow-up contact at 6 months post randomization and every 6 months thereafter will be conducted centrally by the DCRI.	The sites will conduct the initial study follow-up at 60 (+/- 14) days. Follow-up contact at 6 months postrandomization and every 6 months thereafter will be conducted centrally by the DCRI or its designees.	Allow greater flexibility
A1—Duration of Study Participation	The trial will have an approximate 2-year enrollment period and 2-year minimum follow up period. All subjects will be followed from enrollment until either death or the end of the follow-up period.	The trial will have an approximate 42-month enrollment period and 2-year minimum follow-up period. All subjects will be followed from enrollment until either death or the end of the follow-up period.	To allow for additional time, if needed, to attain the desired number of subjects and/or events
B1 1.c Table 2	Event Rate after Negative Study (Prognostic Accuracy)<br><br>CTA: < 0.3%/yr [31,32]	Event Rate after Negative Study (Prognostic Accuracy)<br><br>CTA: < 0.3-0.4%/yr without plaque; 1.1% without significant stenosis[31,32,93]	New data

Section	Original	Revision	Rationale
B1 1.c Table 2		Event Rate after Positive Study (Prognostic Accuracy)<br>CTA: 1.6%/yr with any plaque; 11.9% with any stenosis	New data
B1 1.c Table 2	Detection of Non-Obstructive CAD	Detection of Non-Obstructive CAD<br>CTA: Yes	New data
B1 1.c Table 2	TEST CHARACTERISTICS (CAD with stenosis ≥ 70%; LM ≥ 50%)<br>CTA Sensitivity: 85-99%[16,17,19,24,25]<br>CTA Specificity: 82-95%[16,24,25]	TEST CHARACTERISTICS (CAD with stenosis ≥ 70%; LM ≥ 50%)<br>CTA Sensitivity: 97.2% (CI 96.2%–98.0%)[94]<br>CTA Specificity: 87.4% (CI 84.5%–89.8%)[94]	Updated reference
B1 1.c Table 2		TEST CHARACTERISTICS (suspected CAD)<br>CTA Sensitivity: 97.6% (CI 96.1%–98.5%)[94]<br>CTA Specificity: 89.2% (CI 86.0%–91.8%)[94]	New data

Section	Original	Revision	Rationale
B1 1.d	*Obstructive CAD is relatively uncommon, but nonobstructive CAD is common, clinically important and difficult to detect with functional tests:*	*Obstructive CAD Relatively Uncommon; Nonobstructive CAD Common, Clinically Important, and Difficult to Detect with Functional Tests*	
	In this registry of over 4000 subjects very similar to PROMISE's target population, 40% had nonobstructive disease, 41% had normal anatomy, and only 14% had lesions ≥ to 70%.[55]	In this registry of over 4000 subjects very similar to PROMISE's target population, it was noted by the investigators that 40% had nonobstructive disease, 41% had normal anatomy, and only 14% had lesions greater than or equal to 70% (written communication from Gil Raff, 2008).	To insert this reference to a personal communication in the text and remove it from the list of references.
	The investigational arm uses an "anatomic" testing strategy with coronary CTA (≥64 slice) as the initial test. The usual care arm uses a "functional" stress testing strategy using physician-selected stress imaging (echocardiography or nuclear) or exercise electrocardiography as the initial test.	The "anatomic" testing strategy uses coronary CTA (greater than or equal to 64-slice) as the initial test. The "functional" stress testing strategy uses physician-selected stress imaging (ECHO or nuclear) or exercise ECG as the initial test.	CTA arm is not investigational so for clarity, CTA will be defined as the "anatomic" testing strategy; remove "usual care arm" and retain "functional" test strategy as more commonly used terminology; consistency.
	Ten thousand subjects (10,000) will be randomized over approximately 24 months and followed for a minimum of 2 years (average 2.5 years).	Ten thousand subjects will be randomized over approximately 42 months and followed for a minimum of 2 years (average 2.5 years).	To allow for additional time, if needed, to attain the desired number of subjects and/or events
C1	The investigational arm will use an "anatomic" testing strategy with coronary CTA (≥64 slice) as the initial test. The usual care or "functional" testing strategy will use either	One testing arm will use an "anatomic" testing strategy with coronary CTA (greater than or equal to 64-slice) as the initial test. The other arm, or "functional" testing	CTA arm is not investigational so for clarity, CTA will be defined as the "anatomic"

Section	Original	Revision	Rationale
	stress imaging (echocardiography or nuclear) or exercise ECG as the initial test	strategy, will use either stress imaging (echocardiography or nuclear) or exercise ECG as the initial test.	testing strategy; remove "usual care arm" and retain "functional" test strategy as more commonly used terminology; consistency
	Subjects will be randomized over approximately 24 months and followed for 24-48 months at 200-250 North American primary care, cardiology and acute care practice sites, reflecting the physician specialties and community settings where the vast majority of chest pain patients receive care.	Subjects will be randomized over approximately 42 months and followed for 24 to 60 months (or until the study ends) at 200 to 250 North American primary-care, cardiology, and acute-care practice sites, reflecting the physician specialties and community settings where the vast majority of chest-pain patients receive care.	To allow for additional time, if needed, to attain the desired number of subjects and/or events

Section	Original	Revision	Rationale
C2	The primary aim of PROMISE is to determine if an initial anatomic testing strategy with CTA (≥ 64 slice; investigational care) in symptomatic subjects with low to intermediate risk for CAD will reduce the composite primary endpoint (all cause death, myocardial infarction, major peri-procedural complications and hospitalization for unstable angina) when compared to an initial functional testing strategy (stress echo, stress nuclear, or exercise ECG; usual care), over an average of 2.5 years of follow-up (range approximately 2–4 years).	The primary aim of PROMISE is to determine if an initial anatomic testing strategy with CTA (greater than or equal to 64 slice; anatomic care) in symptomatic subjects with low-to-intermediate risk for CAD will reduce the composite primary endpoint (all-cause death, MI, major peri-procedural complications, and hospitalization for unstable angina) when compared with an initial functional testing strategy (stress ECHO, stress nuclear, or exercise ECG), over an average of 2.5 years of follow-up (range approximately 2–4 years, or until the end of the study).	To insert more commonly used terminology; to allow for additional time, if needed, to attain the desired number of subjects and/or events
C5	*Usual care or functional testing arm: Stress nuclear, stress echocardiography and exercise ECG*	*Functional Testing Arm; Stress Nuclear, Stress ECHO, and Exercise ECG*	To insert more commonly used terminology; consistency
C5	Thus, it is essential that exercise ECG, stress echocardiography and stress nuclear imaging are all included in the usual care or functional testing arm.	Thus it is essential that exercise ECG, stress ECHO, and stress nuclear imaging are all included in the functional testing arm.	To reflect more commonly used terminology; consistency
C5	*Experimental or anatomic testing Arm: CTA.*	*Anatomic Testing Arm: CTA*	CTA arm is not experimental so for clarity will be defined as the anatomic testing arm
D1<br>Figure	Testing level: Changed “Intervention” to “Testing”; deleted “Usual Care Arm” and “Intervention Arm” headings in boxes<br><br>Follow-up level: Added “(+/- 14)” after “60”		Clarification; “intervention” objected to by some of the local IRBs because they felt that it is tied to the CTA<br><br>Allow greater flexibility

Section	Original	Revision	Rationale
D2	All of the modalities in PROMISE are clinically well established and performed routinely and safely across the U.S. No experimental treatment or testing is involved.	All of the modalities in PROMISE are clinically well established and performed routinely and safely across the U.S. No experimental treatment or testing is involved, and there should be equipoise about the feasibility to perform either functional OR anatomic testing.	Clarification; subject and physician should be at equipoise about the feasibility to perform either functional OR anatomic testing
D2 2.a	2. No prior evaluation for this episode of symptoms	2. No prior cardiac evaluation for this episode of symptoms	Clarification; prior evaluation for noncardiac causes does not disqualify subject participation
D2 2.a	5. If age in men 45–54 or women 50–64 years, then must have increased probability of CAD due to EITHER:	5. If age in men 45–54 years or women 50–64 years, then must have increased probability of CAD due to 1 or more of the following risk factors:	Clarification
D2 2.b	1. Diagnosed or suspected ACS requiring hospitalization or urgent or emergent testing; Elevated troponin or CK-MB	1. Diagnosed or suspected ACS requiring hospitalization or urgent or emergent testing; elevated troponin or creatine kinase-myocardial band (CK-MB); outpatients who have completed a rule-out ACS protocol are eligible provided they have negative biomarkers x 2 and a nondiagnostic or normal ECG.	Allow greater flexibility and clarification
D2 2.b	2. Hemodynamically or clinically unstable condition (systolic BP < 90 mm Hg, atrial or ventricular arrhythmias, or persistent resting chest pain felt to be ischemic despite adequate therapy)	2. Hemodynamically or clinically unstable condition (systolic blood pressure [BP] less than 90 mm Hg, severe atrial or ventricular arrhythmias, or persistent resting chest pain felt to be ischemic despite adequate therapy)	Clarification
D2 2.b	3. Known CAD with prior MI, PCI, CABG or any angiographic evidence of CAD ≥ 50% lesion in a major	3. Known CAD with prior clinical history of MI, PCI, coronary artery bypass graft (CABG) or any angiographic evidence of	Clarification

Section	Original	Revision	Rationale
	epicardial vessel	CAD greater than or equal to 50% lesion in a major epicardial vessel	
D2 2.b	4. Any invasive coronary angiography or non-invasive anatomic or functional CV test for detection of CAD, including CTA and exercise ECG, within the previous twelve (12) months.	4. Any invasive coronary angiography or noninvasive anatomic or functional cardiovascular test for detection of CAD, including CTA and exercise ECG, within the previous 12 months (+/- 30 days); prior resting ECG and/or resting ECHO do not constitute an exclusion to participation	Allow greater flexibility and clarification
D2 2.b	5. Known significant congenital, valvular (≥moderate) or cardiomyopathic process (hypertrophic cardiomyopathy or reduced systolic left ventricular function [LVEF ≤ 40%)) that could explain cardiac symptoms.	5. Known significant congenital, valvular (greater than or equal to moderate) or cardiomyopathic process (hypertrophic cardiomyopathy or reduced systolic left ventricular [LV] function [LV ejection fraction less than 40%]) that could explain cardiac symptoms	Correct error
D2 2.b	6. Contraindication to undergoing a CTA, including but not limited to:<br>a. Allergy to iodinated contrast agent<br>b. Unable to receive beta blockers unless heart rate ≤ 65 beats per minute<br>c. Pregnancy	6. Contraindication to a CTA, including, but not limited to:<br>a. Allergy to iodinated contrast agent<br>b. Pregnancy	Remove 1 contraindication

Section	Original	Revision	Rationale
D2 2.b		7. Any other contraindications that would preclude performing a CTA per local site practice, such as 1 or more of the following:<br>a. Inability to receive beta blockers if heart rate is greater than 65 beats per minute<br>b. Agatston score greater than 800<br>c. Body mass index (BMI) greater than 40<br>d. Cardiac arrhythmia	Delineate separately the contraindications that can be determined by the local site
D3 3.a	Therefore, all commonly used tests are included in the 'usual care' arm, a designation which generally implies a multiplicity of approaches.	Therefore, all commonly used tests are included in the functional testing arm, a designation that generally implies a multiplicity of approaches.	To insert more commonly used terminology; consistency
D3 3.b	Usual Care Arm:	Functional Testing Arm:	To insert more commonly used terminology; consistency
D3 3.b	Intervention Arm: In subjects randomized to the intervention arm, a contrast-enhanced coronary CTA will be performed as the initial test.	Anatomic Testing Arm: In subjects randomized to the anatomic testing arm, a contrast-enhanced coronary CTA will be performed as the initial test.	Clarification

Section	Original	Revision	Rationale
D3 3.c	However, because coronary CTA is a relatively new technology and practice requirements and expertise have not evolved to the same level as other diagnostic tests, reader certification may include online review and evaluation of clinical cases consisting of paired coronary CTA and invasive angiography datasets.	However, because coronary CTA is a relatively new technology and practice requirements and expertise may not have evolved to the same level as other diagnostic tests, reader qualification may include online review and evaluation of clinical cases consisting of paired coronary CTA and invasive angiography datasets.	Accuracy
D3 3.e	Prior to beginning enrollment, eligible sites and readers will be certified by the Diagnostic Testing Coordinating Center (DTCC) based on site and reader surveys, on successful transfer of 1 or more complete data set(s) with sufficient image quality and completeness for each modality, and participation in the CTA case review. Final site certification will be issued by the DTCC for each modality before subject enrollment.	Before beginning enrollment, eligible sites and readers will be qualified by the Diagnostic Testing Coordinating Center (DTCC) based on site and reader surveys, on successful transfer of 1 or more complete data sets with sufficient image quality and completeness for each modality, and participation in the CTA case review. Final site qualification will be issued by the DTCC for each modality before subject enrollment.	Accuracy

Section	Original	Revision	Rationale
E2	Subsequently, subjects will have either a telephone call or clinic visit at 60-days for outcome evaluation and recording of any test complications. After that, subjects will be contacted by the DOFG at 6 months post randomization and at 6-month intervals for subsequent follow up assessments until death, withdrawal or the end of the trial.<br><br>Day 60 Post-Randomization Follow-up Assessment—site clinic visit or telephone call:<br><br>Follow-up at 6 months post-randomization and every 6 months thereafter will be conducted by DOFG staff:	Subsequently, subjects will have either a telephone call or clinic visit at 60 (+/- 14) days for outcome evaluation and recording of any test complications. After that, subjects will be contacted under DOFG supervision by either DOFG or its trained designees at 6 months postrandomization and at 6-month intervals for subsequent follow-up assessments until death, withdrawal, or the end of the trial.<br><br>Day 60 (+/- 14 days) Postrandomization Follow-up Assessment—site clinic visit or telephone call:<br><br>Follow-up at 6 months postrandomization and every 6 months thereafter will be conducted by DOFG staff or its trained designees:	Allow greater flexibility

Section	Original	Revision	Rationale
E4		***Radiation Exposure Safety Endpoint***<br>Cumulative radiation exposure will be collected as follows:<br>• CTA: The actual administered dose (computed tomography dose index volume and dose length product for CTA) will be recorded by sites and confirmed by extracting information directly from the images.<br>• Nuclear imaging: The injected/administered contrast agent dose will be recorded and standard tables used to convert into equivalent doses for appropriate comparison with CTA.<br>• Invasive coronary angiography and intervention: The actual administered radiation dose or fluoroscopy time will be recorded by sites and standard tables used to convert into equivalent doses for appropriate comparison with CT.<br>These measures will ensure accurate calculation of the actual cumulative radiation exposure in each arm for tests performed for the diagnostic work-up during the 60 days following enrollment. In addition, we will capture all cardiac diagnostic testing involving radiation (CTA, nuclear cardiology, catheterization) performed during the entire follow-up period and will estimate cumulative radiation exposure over the entire trial using our original data collection (average dose per test for each site) to extrapolate radiation exposure during follow-up.	Ensure accurate calculation of the actual radiation exposure for each participant/each arm of the study for tests performed for the diagnostic work-up during the 60 days following enrollment

Section	Original	Revision	Rationale
E5	Primary outcome events will be documented through 60 days after randomization by the site investigator or authorized designees. After that, the DCRI Outcomes and Follow-up Group (DOFG) will be responsible for documenting study events from 6 months through at least 48 months or the last date the subject participated in the study.	Primary outcome events will be documented through 60 (+/- 14) days after randomization by the site investigator or authorized designees. After that, the DOFG or its designees will be responsible for documenting study events from 6 months until the end of the study.	To allow greater flexibility and for clarification; ensure all subjects receive minimum 2 years of follow-up and all subjects will be followed until the last subject reaches 2 years, at which point the study will end
E6	Follow-up quality of life questionnaires will be administered to subjects via structured telephone interview by DOFG interviewers at 6 and 12 months post-randomization, and annually thereafter for up to 4 years following enrollment.	Follow-up QOL questionnaires will be administered to subjects via structured telephone interview by DOFG trained and supervised interviewers at 6 and 12 months postrandomization and annually thereafter until the end of the study.	Clarification; ensure all subjects receive minimum 2 years of follow-up and all subjects will be followed until the last subject reaches 2 years, at which point the study will end; DOFG trained, supervised interviewers allows for greater flexibility
E6		Proxy questionnaires will be used if the subject is unable to participate in follow-ups via telephone interview.	To allow an alternative for subjects who might otherwise drop out of the study

Section	Original	Revision	Rationale
E8 8.b	Accordingly, all subjects will be asked to provide a blood sample for deposit into a biomarker repository at the time of randomization for future assessment of advanced molecular biomarkers (plasma, serum) such as troponin and hsCRP relevant to disease characterization, risk stratification, characterization of treatment response and adverse effects.	Accordingly, subjects will be asked to provide a blood sample for deposit into a biomarker repository at the time of randomization for future assessment of advanced molecular biomarkers (plasma, serum) such as troponin and high-sensitivity C-reactive protein relevant to disease characterization, risk stratification, characterization of treatment response and adverse effects.	Clarification; not all sites are participating in the biomarker repository so not all subjects can be asked to participate
E8 8.b	In addition, subjects will be requested to separately consent to allow use of the biorepository sample for genomic testing (RNA, DNA).	In addition, subjects will be requested to separately consent to allow use of the biorepository sample for genetic testing (DNA).	Accuracy
G4	Study records will be maintained by the site investigators for a period of three (3) years following the expiration of the grant or length of time as required by local regulations.	Study records will be maintained by the site investigators for a period of 6 years following the expiration of the grant or length of time as required by local regulations.	Accuracy
I3	The PROMISE timeline includes a 6-month start up period, followed by approximately 24 months of enrollment, approximately 24 months of follow up, and 6 months of close out and data analysis.	The PROMISE timeline includes a 9-month start-up period, followed by approximately 42 months of enrollment, approximately 30 months of follow-up after last subject enrolled, and 6 months of closeout and data analysis.	To allow for additional time, if needed, to attain the desired number of subjects and/or events and to clarify that the study will end after the 24-month follow-up for the last subject enrolled
J		Removed # 55	Moved this unpublished reference to the body of the protocol
J		Added #s 25 and 31	References for new data in Table 2

Section	Original	Revision	Rationale
J	The following corrections were also made to the references:<br><br>1. American Heart Association. Heart disease and stroke statistics-2008 update http://circ.ahajournals.org/cgi/content/full/117/4/e25 (Accessed December 23, 2009).<br>corrected to:<br>1. American Heart Association. Heart disease and stroke statistics-2008 update http://circ.ahajournals.org/cgi/content/full/117/4/e25 (Accessed December 23, 2009).<br><br>7. A Report from the NCCSDO: Are rapid access chest pain clinics effective and fair? http://www.sdo.nihr.ac.uk/files/project/32-final-report.pdf (Accessed December 23, 2009).<br>corrected to:<br>7. Timmis AD, Feder GS, Sekhri N, Hemingway H. Are rapid access chest pain clinics effective and fair? Characteristics and outcomes of patients from six centres. National Co-ordinating Centre for NHS Service Delivery and Organisation R & D; 2005:228. Available at: http://www.sdo.nihr.ac.uk/files/project/32-final-report.pdf [Accessed February 10, 2011].<br><br>8. Mudrick DW, Shah BR, Cowper P, et al. Downstream Care and Outcomes Following Outpatient Stress Testing in the United States: How Low is Too Low Risk? Circulation 2009;120:S393.<br>corrected to:<br>8. Abstract 800: Downstream Care and Outcomes Following Outpatient Stress Testing in the United States: How Low is Too Low Risk? 2009. Available at: http://circ.ahajournals.org/cgi/content/meeting_abstract/120/18_MeetingAbstracts/S393?maxtoshow=&hits=10&RESULTFORMAT=&fulltext=mudrick&searchid=1&FIRSTINDEX=0&volume=120&issue=18+Supplement&resourcetype=HWCIT [Accessed February 10, 2011].		Corrected to adhere to American Medical Association style

<table>
<tr><th>Section</th><th>Original</th><th>Revision</th><th>Rationale</th></tr>
<tr><td></td><td colspan="2">24. Institute for Clinical and Economic Review. Coronary computed tomographic angiography for detection of coronary artery disease. http://www.icer-review.org/index.php/ccta.html (Accessed December 23, 2009).
corrected to:
24. CORONARY COMPUTED TOMOGRAPHIC ANGIOGRAPHY
FOR DETECTION OF CORONARY ARTERY DISEASE. 2008. Available at: http://www.icer-review.org/index.php/CCTA/View-category.html [Accessed February 10, 2011].

45. Koenig W, Bamberg F, Lee H, et al. High-sensitivity troponin reliably excludes acute coronary syndrome in patients with acute chest pain: Results from the rule out myocardial infarction by computed tomography (ROMICAT) Study. Circulation S637.
corrected to:
47. Abstract 981: High-Sensitivity Troponin Reliably Excludes Acute Coronary Syndrome in Patients with Acute Chest Pain: Results from the Rule Out Myocardial Infarction by Computed Tomography (ROMICAT) Study. 2008. Available at: http://circ.ahajournals.org/cgi/content/meeting_abstract/118/18_MeetingAbstracts/S_637 [Accessed February 10, 2011].

66. Government Accountability Office. Medicare Part B imaging services: Rapid spending growth and shift to physician offices indicate need for CMS to consider additional management practices. www.gao.gov/new.items/d08452.pdf (Accessed December 23, 2009).
corrected to:
66. Medicare Part B imaging services. Rapid spending growth and shift to physician offices indicate need for CMS to consider additional management practices. Washington, DC: Government Accountability Office; 2008:55. Available at: http://www.gao.gov/new.items/d08452.pdf [Accessed February 10, 2011].

75. Ficaro EP, Hansen CL, et.al. American Society of Nuclear Cardiology Imaging Guidelines for Nuclear Cardiology Procedures Journal of Nuclear Cardiology 2007 Nov/Dec:61-78
corrected to:
Imaging Guidelines for Nuclear Cardiology Procedures: SPECT and Instrumentation. Journal of Nuclear Cardiology. 2007;14(6):913-913.</td><td>Corrected to adhere to American Medical Association style</td></tr>
<tr><td>J</td><td colspan="2">Many reference numbers changed due to the addition and deletion of references.</td><td>Self-evident</td></tr>
</table>

Section	Original	Revision	Rationale
Throughout the document	Made grammatical and spelling corrections		Correctness

TABLE OF ABBREVIATIONS

ABI	ankle brachial index
ACC	American College of Cardiology
ACRIN	American College of Radiology Imaging Network
ACS	acute coronary syndrome
AHA	American Heart Association
BMI	body mass index
BP	blood pressure
CABG	coronary artery bypass graft
CAD	coronary artery disease
CCC	Clinical Coordinating Center
CD	compact disc
CI	confidence interval
CK-MB	creatine kinase-myocardial band
COCATS	Core Cardiology Training Symposium
CTA	coronary tomographic angiography
DASI	Duke Activity Status Index
DCRI	Duke Clinical Research Institute
DOFG	Duke Clinical Research Institute Outcomes and Follow-up Group
DSMB	Data and Safety Monitoring Board
DTCC	Diagnostic Testing Coordinating Center
ECG	electrocardiogram
ECHO	echocardiogram
eCRF	electronic case report form
EQOL CC	Economics and Quality of Life Coordinating Center
g/L	grams per liter
HDL	high-density lipoprotein
IEC	institutional ethics committee
IRB	institutional review board
IVRS	interactive voice response system
LDL	low-density lipoprotein
LV	left ventricular
MAR	missing at random
MI	myocardial infarction

MOP	manual of procedures
mSv	millisievert
NCDR	National Cardiovascular Data Registry
NHLBI	National Heart Lung and Blood Institute (U.S.)
PAD	peripheral arterial disease
PCI	percutaneous coronary intervention
PI	principal investigator
PROMISE	PROspective Multicenter Imaging Study for Evaluation of Chest Pain
QA	quality assurance
QOL	quality of life
ROC	receiver operating characteristic
SAQ	Seattle Angina Questionnaire
SDCC	Statistical and Data Coordinating Center
SF-12	Medical Outcomes Study Short Form
ULN	upper limit of normal

A Introduction

A1 *Study Synopsis*

**Sponsor**	National Heart Lung and Blood Institute (NHLBI)
**Protocol Title**	The PROMISE Trial - **PRO**spective **M**ulticenter **I**maging **S**tudy for **E**valuation of Chest Pain
**Diagnosis and Main Criterion for Inclusion**	Symptomatic subjects without known coronary artery disease (CAD) for whom a nonemergent, noninvasive cardiovascular diagnostic test for CAD is planned.
**Primary Study Objective**	To determine whether an initial noninvasive anatomic imaging strategy with coronary computed tomographic angiography (CTA) will improve clinical outcomes in subjects with symptoms concerning for CAD relative to an initial functional testing strategy.
**Primary Endpoint**	Time to first event using the composite of the following major cardiovascular events:<br>• Death<br>• Myocardial infarction (MI)<br>• Major complications from cardiovascular procedures including testing (stroke, major bleeding, anaphylaxis and renal failure)<br>• Unstable angina hospitalization
**Secondary Endpoints**	• Death or MI or unstable angina hospitalization<br>• Death or MI<br>• Major complications from cardiovascular procedures and testing (stroke, major bleeding, anaphylaxis, and renal failure)<br>• Medical costs, resource use, and incremental cost effectiveness<br>• Health-related quality of life
**Secondary Safety Endpoint**	Cumulative radiation exposure
**Primary Hypothesis**	An initial anatomic testing strategy will provide information that will result in superior long-term health outcomes as compared with an initial functional testing strategy.

**Study Design**	Pragmatic randomized trial of clinical effectiveness of diagnostic testing strategies for CAD, to be performed in outpatient settings, including urgent care, primary care, and cardiology offices. Qualifying patients presenting with new or worsening symptoms suspicious for clinically significant CAD who require diagnostic testing and have not been previously evaluated for this episode of symptoms will be randomized to an initial strategy of either anatomic or functional testing. All subsequent decisions regarding additional testing, medications, and/or procedures will be at the discretion of the responsible clinical care team.<br>Within the functional testing arm, the subject's care team will select the specific test to be performed (exercise electrocardiogram, stress nuclear imaging, or stress echocardiogram) consistent with "usual care" in that practice setting. The subject's care team will be provided with "Information sheets" summarizing current standards for test interpretation and preventive care, but specific medical treatment will not be mandated by the trial.
**Duration of Study Participation**	The trial will have an approximate 42-month enrollment period and 2-year minimum follow-up period. All subjects will be followed from enrollment until either death or the end of the follow-up period.
**End-of-Study Definition**	2-year minimum follow-up of all patients, with expected 30-month average follow-up for clinical status assessment.
**Number of Subjects**	Approximately 10,000 subjects will be enrolled, which is expected to provide 90% power to detect a 20% reduction in the primary composite event rate in the anatomic testing arm as compared with functional testing.
**Number of Sites**	~200-250
**Study Follow-up**	The sites will conduct the initial study follow-up at 60 (+/-14) days. Follow-up contact at 6 months postrandomization and every 6 months thereafter will be conducted centrally by the Duke Clinical Research Institute or its designees.

A2 *Primary Hypothesis*

The PROspective Multicenter Imaging Study for Evaluation of Chest Pain (PROMISE) hypothesis is that an initial anatomic testing strategy utilizing 64-slice or better coronary computed tomographic angiography (CTA) technology in subjects with low-to-intermediate risk for coronary artery disease (CAD) will reduce the composite primary endpoint (all-cause death, myocardial infarction [MI], major peri-procedural complications, and hospitalization for unstable angina) when compared with an initial functional testing strategy over an average of 2.5 years of follow-up.

A3 *Significance of the Study*

PROMISE is the first large randomized trial comparing the 2 major alternative noninvasive diagnostic strategies for the initial assessment of stable symptoms suspicious for possible CAD. Community-based practices are anticipated to contribute substantially to subject enrollment, which will enhance generalizability of findings in contrast to previous smaller studies that relied on "expert" centers staffed by cardiologists and radiologists. This trial will provide an unbiased comparison of usual-care testing strategies with new CTA technology that is expected to yield definitive and unique evidence regarding the benefits and risks of these alternative approaches, with the primary endpoint being superior health outcomes.

B Background

B1 *Prior Literature and Studies*

1.a Current Management of Patients with Chest Pain: Extent of the Problem

Chest pain is a common presentation, with ~4 million Americans newly diagnosed with angina[1] and more than 6 million presenting to emergency rooms with chest pain annually.[2] The annual incidence of new angina ranges from 1% in middle-aged women to nearly 4% in elderly men in the U.S.,[1] with rates in diabetics of up to 10%.[3] Overall mortality is doubled compared with the general population, and rates range from less than 1% per year for those ultimately diagnosed as "nonanginal" to almost 10% per year for those with CAD and an unfavorable clinical risk profile.[4-8] (See Table 1.) These data suggest that between 4 and 5 million Americans require noninvasive testing for chest-pain symptoms every year. The high event rates in unselected but symptomatic populations support performance of a randomized trial to define optimal testing strategies in this at-risk group and to guide care.

Table 1: Event Rates in Populations with New Onset Chest Pain

Study	Population	Endpoint	Lowest event rates	Highest event rates	Follow-up
Daly BMJ 2006[4]	3,031 (58%M)	Death, MI	2.3% overall	3.9% with angiographic CAD	1 year
Mudrick Circ 2009[8]	98,872	Death, MI, Stroke	1.07%	N/A	1 year
Sekhri Heart 2007[9]	8,762 (52%M)	Death, MI, Unstable angina hospitalization	0.83% noncardiac CP	8.62% angina	1 year
			2.73% noncardiac CP	16.52% angina	3 years
Sekhri BMJ 2008[5]	8,176 (53%M)	Coronary death, MI	3-9% nonspecific CP	16-19% typical CP	2.5 years
Clayton BMJ 2005[6]	7,311 (79%M)	Death, MI, stroke	4% lowest risk decile	35% highest risk decile	5 years

MI=myocardial infarction; CAD=coronary artery disease; N/A=not applicable; CP=chest pain

In patients presenting with symptoms suggestive of CAD, such as chest pain or exertional dyspnea, the clinical risk profile and presentation are often insufficient to definitively exclude CAD and/or are not sufficient to implement invasive evaluation or revascularization without additional information. Thus, noninvasive diagnostic testing is required in the majority of such patients. Consistent with these data, an exercise electrocardiogram (ECG) was performed in 65% of all British chest-pain patients,[7] rising to over 90% in those eventually diagnosed with angina. In the Euro Heart Survey of Chronic Stable Angina, 76% of patients received an exercise ECG, while 18% had a stress imaging study, and 64% had a resting echocardiogram (ECHO). A coronary angiogram was performed or planned in 41%.[10]

1.b Current Diagnostic Strategies Using Functional Tests are Inadequate

American College of Cardiology (ACC)/American Heart Association (AHA) Guidelines and current noninvasive diagnostic testing includes exercise ECG, stress ECHO, and stress nuclear imaging, all of which are based on detecting stress-induced myocardial ischemia caused by obstructive CAD.[11-13] All are also associated with high rates of

false-positive and false-negative results in the detection of significant CAD (defined as a left main obstruction greater than or equal to 50% and/or any major epicardial vessel obstruction greater than or equal to 70%), limited prognostic discrimination, relatively short “warranty period” for a negative test, and an inability to detect possibly significant nonobstructive CAD. The literature regarding functional test characteristics is summarized below in Table 2, along with reports of clinical event rates after testing.

False-negative test results may delay needed treatments and lead to worse clinical outcomes, while false-positive test results may lead to unnecessary treatment and procedures. Evidence of the inadequacies of current diagnostic strategies includes the occurrence of undetected MI (2.1%) and unstable angina (2.3%) in patients with suspected acute coronary syndrome (ACS) who are discharged from the emergency room.[14] A missed diagnosis of MI was associated with a 9.8% 30-day mortality vs. only 5.7% in hospitalized patients, or a relative risk of 1.9. Other relevant evidence comes from an analysis of the large ACC National Cardiovascular Data Registry, which suggests that misdiagnosis of chest pain is common in community practice.[15] Of nearly 400,000 patients without known CAD who underwent elective diagnostic catheterization, 84% had prior noninvasive testing, which was positive in 82%. Nevertheless, only 38% of patients overall, and 41% of those with positive tests, had obstructive CAD (left main stenosis greater than or equal to 50%; major epicardial vessel greater than or equal to 70%). Thus current clinical practice, as guided by functional stress testing, results in a pattern of referral to invasive angiography, which does not lead to revascularization in the majority of patients, despite the inherent risks and expense of an invasive test.

1.c Computed Tomographic Angiography is Highly Accurate in Detecting and Excluding Obstructive and Nonobstructive Coronary Artery Disease

The recent technological advance of coronary CTA permits direct visualization of the coronary arteries, allowing noninvasive detection of significant stenoses and with great accuracy.[16-19] Computed tomographic angiography also extends the spectrum of disease by detecting nonobstructive lesions and visualizing coronary plaque, important prognostic predictors. Computed tomographic angiography test characteristics and associated event rates are shown in Table 2 below. The superior ability of CTA to detect obstructive CAD means that patients at greatest risk will be more accurately identified and treated with life-saving therapies proven to reduce death and MI by about 30%,[20] while those without disease will not be subjected to unnecessary additional, perhaps invasive, testing.

Table 2: Diagnostic Test Characteristics and Resulting Event Rates

	Stress Nuclear	Stress ECHO	Exercise ECG	CTA
**TEST CHARACTERISTICS** (CAD with stenosis ≥ 70%; LM ≥ 50%)				
Sensitivity	76%–94%[21-24]	87%–88%[21-24]	52%[21]	85%–99%[25]
Specificity	62%-88%[21-24]	77%-84%[21-24]	71%[21]	82%–95%[25]

Event Rate after Negative Study (Prognostic Accuracy)	0.5%–2.0%/yr[26-30]	0.5%–2.0%/yr[26-30]	3%/yr[5]	< 0.3%–0.4%/yr without plaque; 1.1%without significant stenosis[31-33]
Event Rate after Positive Study (Prognostic Accuracy)				1.6%/yr with any plaque; 11.9% with any stenosis[31]
Detection of Nonobstructive CAD	N/A	N/A	N/A	Yes[31]
**OVERALL EVENT RATES** (1 yr; all patients)				
Death	0.45%–2.5%[34-37]	2.9%–6.6%[38-40]	0.5%–5.0%[41-43]	0.3%–2.2%[32,33,44-47]
Nonfatal MI	0.9%–1.3%[34-37]	0.9%–1.3%[38-40]	-	0.4%–0.6%[32,33,44-47]
Unstable Angina Hospitalization	3.8%	-	-	0.2&–4.2%

ECHO=echocardiogram; ECG=electrocardiogram; CTA=computed tomographic angiography; CAD=coronary artery disease; LM=left main; yr=year; N/A=not applicable; MI=myocardial infarction

1.d Additional Important Considerations in Comparing Test Characteristics

Limited Evidence Base to Guide Diagnostic Testing

The majority of the studies discussed above were conducted in single academic centers and are plagued by numerous sources of bias (verification, selection, etc.), making evaluation of true accuracy very difficult.[48,49] Data from academic research settings provide only a rough indication of actual performance in the real world.[50-52] Even these data, however, are based on convenience samples of subjects already referred for a particular test rather than the true population of interest.[50] The absence of high-quality unbiased comparative data on test performance and outcomes has led to the unusual situation of conflicting ACC/AHA Guidelines, with each of these relevant documents recommending the initial use of a different test: treadmill ECG, stress ECHO, or stress perfusion imaging.[11-13,53,54] Further, very few studies have addressed Fryback and Thornberry's highest levels of evidence for assessing diagnostic testing: subject outcome efficacy and societal efficacy.[55] This situation has resulted in clinical confusion, performance of multiple tests, and marked variation in clinical practice that can only be addressed by high-quality effectiveness research based on improving patient outcomes.

Obstructive CAD Relatively Uncommon; Nonobstructive CAD Common, Clinically Important, and Difficult to Detect with Functional Tests

The National Cardiovascular Data Registry (NCDR) analysis shows that the absence of significant obstructive disease is common in patients undergoing diagnostic

catheterization. Of the 62% of patients without significant CAD, two-thirds had nonobstructive CAD (20%-70% epicardial and less than 50% left main) and one-third had no CAD (defined as stenosis less than 20%).[15] A similar pattern has been noted by others: in 400 patients at low-intermediate risk for CAD undergoing CTA, 47% had nonobstructive CAD, 13% had obstructive stenoses, and 27% had no disease, with 13% of scans being inconclusive.[45] These data are similar to Ostrom et al[33] and the Michigan Advanced Cardiovascular Imaging Consortium findings. In this registry of over 4000 subjects very similar to PROMISE's target population, it was noted by the investigators that 40% had nonobstructive disease, 41% had normal anatomy, and only 14% had lesions greater than or equal to 70% (written communication from Gil Raff, 2008). Finally, only 14% of the subjects in a multicenter CTA study, Assessment by Coronary Computed Tomographic Angiography of Individuals Undergoing Invasive Coronary Angiography (ACCURACY), had obstructive disease (defined as stenosis greater than or equal to 70%) on catheterization.[16] Thus, current clinical data suggest that nonobstructive CAD will be frequent and obstructive CAD relatively uncommon in the PROMISE cohort.

Long-term outcomes in subjects with nonobstructive disease on angiography, such as was found in 39% of the NCDR cohort,[15] have been found to be intermediate between subjects with obstructive CAD or with no disease.[56,57] This is consistent with the known prognostic power of coronary calcium scoring,[56] and an observed 2x increase in death in patients with nonobstructive disease compared with those with normal coronary anatomy on CTA.[33,45] The knowledge that most MIs arise from nonobstructive lesions[58,59] provides biologic plausibility to these observed differences in outcomes. Since functional testing relies upon inducing ischemia due to limited coronary flow reserve in the presence of obstructive lesions, it cannot detect potentially important nonobstructive disease. Patients receiving these tests will have undetected nonobstructive disease. In contrast, nonobstructive disease is routinely detected by CTA, yielding information that may be important for properly adjusting the intensity of preventive therapy to reflect the presence of coronary plaque.

Therefore, in response to these needs, the PROMISE trial, a multicenter, randomized, pragmatic trial, is comparing 2 state-of-the-art diagnostic strategies in symptomatic subjects at clinically determined, low-intermediate risk for CAD who require nonurgent testing. The "anatomic" testing strategy uses coronary CTA (greater than or equal to 64-slice) as the initial test. The "functional" stress testing strategy uses physician-selected stress imaging (ECHO or nuclear) or exercise ECG as the initial test. Ten thousand subjects will be randomized over approximately 42 months and followed for a minimum of 2 years (average 2.5 years). All subsequent diagnostic and therapeutic management decisions will be based on the latest clinical practice guideline recommendations and will be at the discretion of the treating care team.

B2 *Rationale: The Urgent Need for a Randomized Trial of Diagnostic Strategies in Subjects with Suspected Coronary Artery Disease*

Millions of patients undergo different diagnostic testing for suspected CAD in the U.S. every year despite little or no evidence that it will improve their outcomes. The remarkable imprecision and inefficiency of current evaluation strategies is perhaps best documented by the high rate (up to 60%) of invasive coronary angiograms in which no

significant CAD is detected,[15] while the U.S. economic burden of caring for patients with chest pain exceeds $75 billion/year.[60] Limitations of current tests, an inadequate evidence base, and escalating costs have led all major professional societies, Centers for Medicare and Medicaid Services, and National Institutes of Health to publicly demand the development of new evidence to optimize the clinical use of diagnostic testing, reduce unnecessary invasive procedures and control costs. Further, all stakeholders have called for further investigation of the appropriate role of coronary CTA, a novel diagnostic imaging technology that may potentially change the standard of care in this common but challenging situation.[48,52,61-63]

The problem at hand must be addressed by an effectiveness trial, as examination of efficacy only (i.e., optimal performance at expert sites) would fail to capture the actual performance of diagnostic strategies in the community where the vast majority of these tests are performed and where future clinical decisions based on trial results will impact patient outcomes. The concept of a pragmatic clinical trial, as proposed by Tunis et l,[64] is an ideal format to acquire the quality of evidence required to address the needs of clinical decision makers. The PROMISE study embodies all the characteristics of such a trial, including (1) incorporation of clinically relevant alternatives, including usual clinical testing and usual clinical care; (2) a diverse study population; (3) heterogeneous practice settings; and (4) use of a broad range of health outcomes rather than focusing solely on cost. Each of these features is essential to establish an evidence-based standard of care for the large number of patients who require diagnostic testing for symptoms of CAD.

Thus, the ideal approach to addressing these problems is a randomized trial of different testing strategies that is adequately powered to demonstrate superior clinical outcomes, as only improved patient health would be sufficiently motivating to change practice and justify the routine use and reimbursement of a new technology. Because test-reimbursement policies can be expected to evolve and change in response to many nonmedical forces in the economy, conclusions based primarily on the differential costs of alternative testing strategies are unstable over the long term. The ideal trial must also directly target the clinical outcomes that are most relevant to assessing value. Finally, the ideal trial must ensure the broadest possible applicability of its results by demonstrating real-world effectiveness across the appropriate spectrum of tests, practice settings, and caregiver specialties and expertise, which are pertinent to the clinical decisions needed to care for the vast majority of chest-pain patients nationwide. The PROMISE trial design has been shaped by these objectives.

C Study Overview and Objectives

C1 *Overview of the PROMISE Trial*

PROMISE is a multicenter, randomized, pragmatic trial comparing 2 state-of-the-art diagnostic strategies in approximately 10,000 symptomatic, low-to-intermediate–risk subjects with suspected CAD who require nonurgent testing. One testing arm will use an "anatomic" testing strategy with coronary CTA (greater than or equal to 64-slice) as the initial test. The other arm, or "functional" testing strategy, will use either stress imaging (ECHO or nuclear) or exercise ECG as the initial test. All subsequent diagnostic and therapeutic management will be at the discretion of the treating care team. The trial will encourage adherence to evidence-based practice and document actual therapies used, but will not mandate specific care plans, which will be left to the discretion of the clinical

care team. Subjects will be randomized over approximately 42 months and followed for 24 to 48 months (or until the study ends) at 200 to 250 North American primary-care, cardiology, and acute-care practice sites, reflecting the physician specialties and community settings where the vast majority of chest-pain patients receive care.

C2 *Primary Aim*

The primary aim of PROMISE is to determine if an initial anatomic testing strategy with CTA (greater than or equal to 64-slice; anatomic care) in symptomatic subjects with low-to-intermediate risk for CAD will reduce the composite primary endpoint (all-cause death, MI, major peri-procedural complications, and hospitalization for unstable angina) when compared with an initial functional testing strategy (stress ECHO, stress nuclear, or exercise ECG), over an average of 2.5 years of follow-up (range approximately 2–4 years, or until the end of the study).

The primary endpoint is time to the first event in a composite of major cardiovascular events including:

- Death
- MI
- Major complications from cardiovascular procedures and testing (stroke, major bleeding, anaphylaxis, and renal failure requiring dialysis)
- Unstable angina hospitalization

C3 *Secondary Aims*

The secondary aims are to compare the following clinical and economic outcomes in subjects randomized to initial anatomic versus functional diagnostic testing:

- Death or MI or unstable angina hospitalization
- Death or MI
- Major complications from cardiovascular procedures and testing (stroke, major bleeding, anaphylaxis, and renal failure requiring dialysis)
- Medical costs, resource use, and incremental cost effectiveness
- Health-related quality of life (QOL)

In addition, cumulative radiation exposure will be assessed as a secondary safety endpoint.

C4 *Rationale for the Selection of Outcome Measures*

Rationale for Clinical Assessments

Because patients with CAD remain at risk for major adverse events related to their disease over their lifetimes, consideration of long-term outcomes is essential to exploring the impact of test selection on health outcomes and costs. The endpoints of all-cause death, nonfatal MI, major complications related to cardiovascular procedures, and

unstable angina hospitalizations are clinically relevant and together reflect the success or failure of the diagnostic testing strategy to accurately detect or exclude disease, guide procedural care, and provide useful long-term prognostic information with high confidence. Any event that could potentially be influenced by the anatomic information provided by CTA, such as percutaneous coronary intervention (PCI), is excluded as an endpoint regardless of when during the follow-up period it occurred. Conversely, major peri-procedural complications are included, as these are a potentially important cause of harm resulting from a need for an invasive study due to inaccurate initial diagnostic tests. The use of a composite clinical endpoint and a subject follow-up of at least 2 years are essential to testing PROMISE's hypothesis of clinical superiority of CTA.

Rationale for Economic and QOL Assessments

The use of noninvasive diagnostic testing for the assessment of CAD has grown at a far faster rate than other medical care, including related procedures such as cardiac catheterization or revascularization.[65] Cardiac diagnostic testing now costs over $4 billion annually for Medicare Part B alone.[66] Although these unsustainable testing costs mandate the development of better evidence, recent efforts by payers to reduce costs have largely addressed the number of tests performed, or payment per test, rather than the value derived for guiding patient care. Thus testing is increasingly driven by onerous administrative practices such as prior authorization designed to discourage physicians from providing indicated care[67] or drastic reductions in reimbursement. This default approach is not in the best interests of either patients or our increasingly dysfunctional health care system.

The studies that have examined the costs and cost effectiveness of functional vs. anatomic testing strategies to date are limited by the lack of reliable long-term effectiveness and cost data.[24,44,68,69] Thus, a formal examination of the incremental cost of testing and its value for patients is urgently needed and is an essential part of the PROMISE trial.

C5 *Rationale for Selection of Testing in Each Experimental Arm*

Functional Testing Arm: Stress Nuclear, Stress ECHO, and Exercise ECG

Each of these procedures is a well-established and accepted strategy to diagnose CAD. Each has also been in routine clinical use for over 20 years and is well supported by class I, level of evidence B ACC/AHA practice guideline recommendations for use in suspected CAD. The guidelines further document specific indications as well as diagnostic and prognostic performance. (See also Table 2.) A national claims database (United HealthCare) documenting contemporary U.S. testing patterns shows that stress nuclear exams constitute 61% of tests, with stress ECHO at 18% and exercise ECG at 21%. Computed tomographic angiography and stress magnetic resonance imaging comprised less than 1% each. Seventy-nine percent of tests were performed with exercise stress.[8] The lack of a single dominant diagnostic strategy for current practice, even in this insured population, highlights the essential need for flexibility and breadth in the choice of functional tests to fully reflect real-world diagnostic practices and maximize generalizability and clinical impact. Thus it is essential that exercise ECG, stress ECHO, and stress nuclear imaging are all included in the functional testing arm.

Anatomic Testing Arm: CTA

Because CTA is quick, robust, readily available, and accurate, it may lead to a major practice shift in the evaluation of chest pain where a strategy of imaging coronary anatomy is preferred over the current practice of first searching for inducible ischemia using functional testing. In this sense its widespread adoption would represent substantial change, as it may improve diagnostic testing in an unexpected way (through anatomic rather than functional information) and may alter the indications for testing, thereby expanding the patient population being tested. Further, there is evidence of increasing physician confidence over time,[70] a hallmark of an improved diagnostic testing strategy.[71] This growing experience indicates that the greatest strength of CTA may lie in excluding obstructive CAD in lower-prevalence cohorts—exactly the symptomatic population being studied in PROMISE.[72] Finally, because of its visual strength, CTA has an inherent important motivational ability that has been shown to improve prescription and adherence to effective preventive strategies, an important goal of any testing strategy.[73] On the other hand, CTA cannot be performed in all patients, as calcified lesions and temporal and spatial resolution can be limiting. However, evidence suggests that CTA is superior to functional testing in reducing false-positive and false-negative test results, identifying nonobstructive disease, improving the prognostic accuracy of a negative test, and extending the “warranty period” of test results, all of which strongly suggest clinical superiority over functional testing.

D Investigational Plan

D1 *Overview of Trial Design*

PROMISE is a multicenter, randomized, pragmatic trial comparing 2 state-of-the-art diagnostic strategies in approximately 10,000 symptomatic, low-to-intermediate CAD-risk subjects with suspected CAD who require nonurgent testing. The following discussion includes trial assessments, outcomes, substudies, organization and operations, sites and site management, timeline, and potential criticisms. See the figure below for a schematic summary of the trial design.

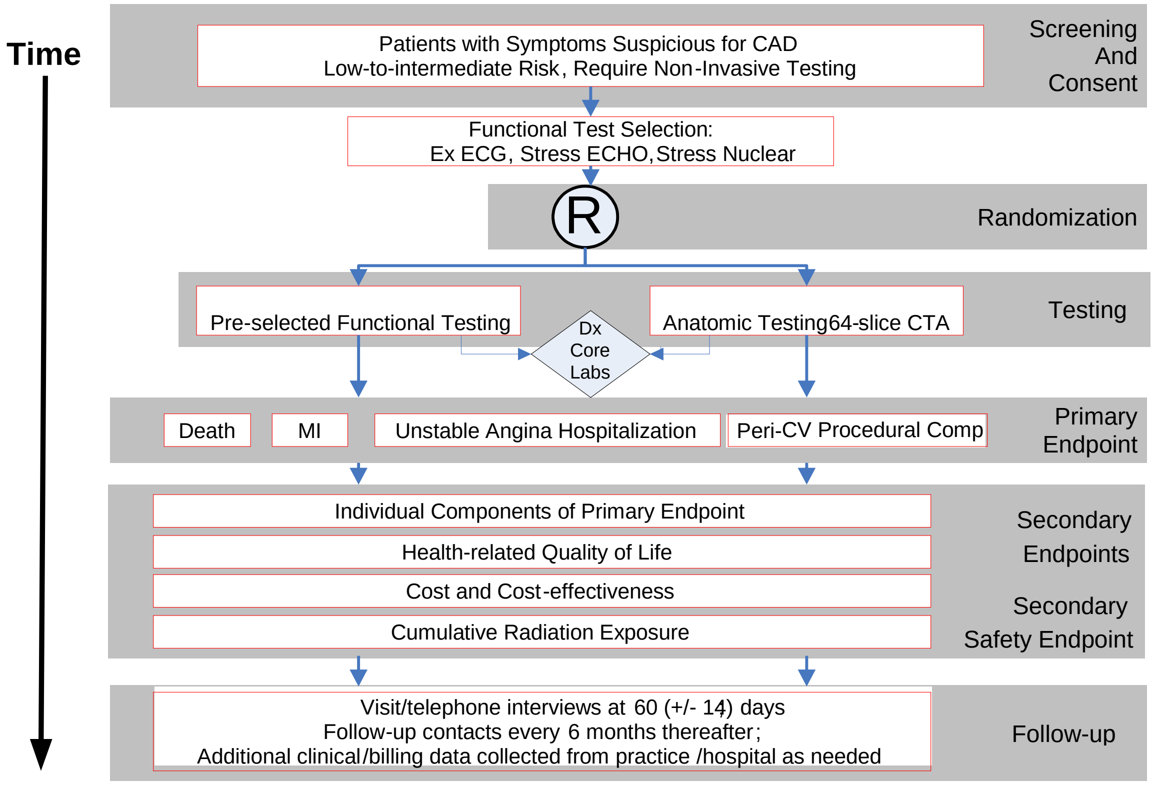

D2 *Subject Population*

To optimize the generalizability of the study results and reflect current patterns of care, subjects considered for enrollment into PROMISE will be outpatients without known CAD who are symptomatic, and whose physician has decided that the subject requires nonurgent, noninvasive cardiovascular testing to further evaluate suspected CAD. This population is purposefully and carefully chosen to be directly reflective of the population in which elective noninvasive cardiac diagnostic testing is currently being used.

PROMISE's inclusion criteria stipulate that all subjects will be symptomatic and will, in the judgment of the physician caring for the subject, require an elective noninvasive test for symptoms suspicious for CAD. Thus, whether or not the subject chooses to participate in the trial, he or she will likely undergo testing. All of the modalities in PROMISE are clinically well established and performed routinely and safely across the

U.S. No experimental treatment or testing is involved, and there should be equipoise about the feasibility to perform either functional OR anatomic testing. **The trial intervention is simply the random assignment of the initial test.**

Justification of Population

Since subjects are symptomatic and have already been determined to require outpatient noninvasive testing, this is an ideal population in which to compare the results of different testing strategies. We include subjects presenting in primary care offices, cardiology offices, and urgent care settings without suspected ACS to ensure inclusion of all subject profiles; however, patients undergoing testing as part of a rule-out-ACS protocol are not eligible. To ensure broad generalizability, we are using a patient-centric design and not limiting enrollment to a single care setting or single type of provider (cardiology). PROMISE reflects the care settings, patient groups, and providers who will eventually apply its results.

2.a Inclusion Criteria

1. New or worsening chest-pain syndrome or equivalent symptoms suspicious for clinically significant CAD
2. No prior cardiac evaluation for this episode of symptoms
3. Planned noninvasive testing for diagnosis
4. Men age greater than or equal to 55 years and women age greater than or equal to 65 years
5. If age in men 45–54 years or women 50–64 years, then must have increased probability of CAD due to 1 or more of the following risk factors:
    - Diabetes mellitus requiring medical treatment
    - Peripheral arterial disease (PAD), defined as documented peripheral arterial stenosis greater than or equal to 50%, treated medically or invasively
    - Cerebrovascular disease (stroke), defined as documented carotid stenosis greater than or equal to 50%, treated medically or invasively
    - Ongoing tobacco use
    - Hypertension
    - Abnormal ankle-brachial index (ABI), defined as less than 0.9
    - Dyslipidemia
6. Serum creatinine less than or equal to 1.5 mg/dL within the past 90 days
7. Negative urine/serum pregnancy test for female subjects of childbearing potential

2.b Exclusion Criteria

1. Diagnosed or suspected ACS requiring hospitalization or urgent or emergent testing; elevated troponin or creatine kinase-myocardial band (CK-MB); outpatients who have completed a rule-out ACS protocol are eligible provided they have negative biomarkers x 2 and a nondiagnostic or normal ECG.

2. Hemodynamically or clinically unstable condition (systolic blood pressure [BP] less than 90 mm Hg, severe atrial or ventricular arrhythmias, or persistent resting chest pain felt to be ischemic despite adequate therapy)
3. Known CAD with prior clinical history of MI, PCI, coronary artery bypass graft (CABG) or any angiographic evidence of CAD greater than or equal to 50% lesion in a major epicardial vessel
4. Any invasive coronary angiography or noninvasive anatomic or functional cardiovascular test for detection of CAD, including CTA and exercise ECG, within the previous 12 months (+/- 30 days); prior resting ECG and/or resting ECHO do not constitute an exclusion to participation
5. Known significant congenital, valvular (greater than or equal to moderate) or cardiomyopathic process (hypertrophic cardiomyopathy or reduced systolic left ventricular [LV] function [LV ejection fraction less than 40%]) that could explain cardiac symptoms
6. Contraindication to a CTA, including, but not limited to:
    a. Allergy to iodinated contrast agent
    b. Pregnancy
7. Any other contraindications that would preclude performing a CTA per local site practice, such as 1 or more of the following:
    a. Inability to receive beta blockers if heart rate is greater than 65 beats per minute
    b. Agatston score greater than 800
    c. Body mass index (BMI) greater than 40
    d. Cardiac arrhythmia
8. Life expectancy less than 2 years
9. Unable to provide written informed consent or participate in long-term–follow-up

2.c Ethical Considerations

Human Subjects' Involvement and Characteristics

All human adult subjects who meet inclusion criteria and who do not meet any of the exclusion criteria will be considered eligible for this trial. Subjects who are within vulnerable populations will be included at the discretion of the site institutional review boards (IRBs).

2.d Subject Recruitment Plans and Consent Process

Diagnostic testing for the assessment of CAD symptoms is ordered by physicians of all specialties; is performed in multiple settings, including physician offices, hospital outpatient departments, and diagnostic testing facilities; and is interpreted by physicians of multiple specialties, including primary care physicians, cardiologists, and radiologists. Therefore, subjects will be recruited from multiple clinical settings, including outpatient clinics, urgent care centers, and testing facilities. Sites that do not perform the full range of diagnostic testing, including CTA, will be paired with regional referral centers for ECHO, nuclear, and/or CTA testing where subjects may undergo the necessary testing.

Existing clinical referral and research networks will be used to identify and enroll subjects.

Subjects will be recruited from 200 to 250 clinical sites. All potential PROMISE sites will obtain IRB/Institutional Ethics Committee (IEC) approval of the protocol and the associated consent form and any recruitment tools. Written informed consent will be obtained from each subject before to any study procedures are performed. Assessment of CAD risk will begin during screening to ensure eligibility. It will include medical history and physical exam and laboratory testing for the presence/absence of major cardiac risk factors, including BP/hypertension, diabetes, cholesterol (low-density lipoprotein [LDL], high-density lipoprotein [HDL]), smoking, family history, sedentary life style, obesity, cerebrovascular and PAD history, and ABI.

Subjects will be identified and enrolled in a step-wise process as follows:

1. All subjects being considered for outpatient noninvasive testing for the initial evaluation of symptoms suspicious for clinically significant obstructive CAD will be screened.
2. Subjects meeting inclusion criteria and not having any of the exclusion criteria will be approached to participate in the study and have the study explained to them.
3. Subjects agreeing to participate after having all their questions answered will be asked to document their agreement on the study-specific IRB-approved consent form with a signature.

2.e Subject Randomization

Eligible subjects who have given written informed consent and meet all inclusion and no exclusion criteria will be randomly assigned in equal proportions (1:1) to either the anatomic or functional diagnostic testing arm of the trial. A computer-generated permuted block randomization schedule with stratification by clinical site will be used in the trial. Before randomization, the managing caregiver, site investigator, or authorized designee will be asked to indicate the functional test he or she would plan to use if the subject were randomized to the functional testing arm. This information will be tracked and used as another stratification factor in the randomization scheme to facilitate comparisons of anatomic vs. functional testing according to the type of functional test. Subject randomization will be accomplished by telephone through a centralized toll-free interactive voice response system (IVRS).

If a subject is randomized but does not undergo the planned initial diagnostic test within 30 days of randomization, that subject will still be followed and included in the intention-to-treat statistical comparisons according to his or her randomized treatment assignment.

2.f Risks and Benefits

Potential Risks

Protocol-specific risks associated with the PROMISE trial are minimal. All PROMISE enrolled subjects will, by inclusion criteria, require noninvasive testing for their symptoms. Thus all subjects will have compelling medical reasons for performance of the noninvasive test arm to which they are randomized. All care in PROMISE is part of routine clinical practice and, because the only intervention being performed in PROMISE

is the random assignment of initial diagnostic test, safety considerations and adverse events are limited to complications arising from initial testing. Reporting of these safety events will be the responsibility of the site investigator.

Drawing Blood

Risks associated with drawing blood include momentary discomfort and/or bruising, infection, excess bleeding, clotting, or fainting.

Potential Loss of Confidentiality

In any clinical trial, there is a possible risk to subjects as to the potential loss of confidentiality. To prevent this from occurring, the Clinical Coordinating Center (CCC) and the Statistical and Data Coordinating Center (SDCC) at the Duke Clinical Research Institute (DCRI) have strict procedures in place to ensure that all study data are confidential and anonymized except as required for data collection by the Economics and Quality of Life Coordinating Center (EQOL CC) and the DCRI Outcomes and Follow-up Group (DOFG). For all data received by DCRI, subjects will be identified only by unique code numbers. The link to these codes will be maintained at the SDCC. Trial records that identify subjects will be kept secure and confidential as required by law. Federal privacy regulations provide safeguards for privacy, security, and authorized access.

If the DOFG finds that a subject has undergone diagnostic testing or a diagnostic or therapeutic procedure or has been hospitalized, the DOFG will obtain the subject’s test results or discharge summary, and the EQOL CC will obtain relevant medical billing information on behalf of the trial. Once received at the SDCC, these documents will be anonymized, removing the subject’s name, personal identifiers, and local physician’s name and will be identified by unique study enrollment numbers.

Potential Benefits

The PROMISE trial results should improve the care of future subjects requiring diagnostic testing for suspected CAD. In addition, the trial will deliver high-quality data on radiation exposure, incidental findings, and other clinically important “side effects” of the testing strategies that will be examined in a large real-world experience.

D3 *Diagnostic Testing*

3.a Approach to Diagnostic Testing

An important goal of PROMISE is the broadest possible applicability of its results. Therefore, all commonly used tests are included in the functional testing arm, a designation that generally implies a multiplicity of approaches. This inclusiveness will facilitate enrollment by ensuring that all eligible subjects will have ready access to high-quality testing in both arms. Because the preferred type of functional test will be recorded by the IVRS before randomization, the trial subjects, each of whom has been selected by his or her managing clinician to undergo a different form of functional testing, can later be divided into 3 cohorts for prespecified subgroup analyses. In each cohort, half will be randomized to undergo that prespecified functional test, while the other half will be randomized to undergo CTA, forming paired CTA-functional test subgroups for each functional test type.

We will ensure that the standard of care of testing and imaging performance and interpretation consistent with good medical practice is maintained throughout the trial by requiring that sites have expertise and qualify in all modalities, by providing recommended imaging protocols and by providing timely quality feedback and expert overreading in a subset of studies, again for all modalities.

3.b Description of Testing To Be Performed

Subjects will initially be evaluated according to their physicians' specific protocols for management of subjects with an indication for nonurgent, noninvasive cardiovascular testing. Typically, the standard evaluation will include the relevant past and current medical history, a physical examination, and a resting 12-lead ECG and may include cardiac biomarkers (troponin and/or CK-MB) as well as other routinely obtained blood testing. Subjects who meet eligibility criteria will have their physicians' preferred functional tests designated as part of the stratification requirements for randomization.

Functional Testing Arm

For subjects randomized to usual care, the preselected functional test will be performed as the initial test. This includes stress nuclear imaging, stress ECHO, or exercise ECG.

Anatomic Testing Arm

In subjects randomized to the anatomic testing arm, a contrast-enhanced coronary CTA will be performed as the initial test.

Subsequent Care

The results of all tests will be provided to the care team in the usual manner for that testing laboratory, and, depending on the results, subjects may or may not undergo medical treatment and/or additional noninvasive functional, anatomic, or invasive testing (coronary angiography), and/or coronary revascularization, at the care team's discretion. It is assumed that patient management will be additionally informed and guided by the test findings. The local physician will resume care of the subject and make all subsequent clinical decisions (e.g., need for further evaluation or admission) based upon his or her cumulative clinical assessment of the subject, including findings revealed on the noninvasive testing.

3.c Functional and Anatomic Testing

Equipment and Protocols

Criteria for qualification of participating sites or referral laboratories will include use of standard equipment for usual-care testing (stress ECHO, stress nuclear, and exercise ECG) as defined in current practice guidelines, and greater than or equal to 64-slice MDCT technology for coronary CTA.[12,74-77]Similarly, all test acquisition protocols will adhere to best-practice standards as defined in current national practice guidelines.[12,74-77] Sites will be allowed to use their own standard acquisition protocols as long as they fall within the standard-of-care guidelines. Sample protocols are provided in the Manual of Operations and Procedures (MOP).

Interpretation

All studies will be interpreted by qualified physicians who have at least ACC COCATS (Core Cardiology Training Symposium) level 2 training[78] or equivalent. For nuclear studies, certification by the Certification Board of Nuclear Cardiology or Board Certification in nuclear medicine or radiology is also sufficient to qualify as a reader. Coronary CTA will be interpreted by physicians trained at least COCATS level 2 or equivalent, either Society of Cardiovascular Computed Tomography level 2 or the Certification Board of Computed Cardiovascular Tomography. However, because coronary CTA is a relatively new technology, and practice requirements and expertise may not have evolved to the same level as other diagnostic tests, reader qualification may include online review and evaluation of clinical cases consisting of paired coronary CTA and invasive angiography datasets.

All diagnostic tests will be interpreted and reported by diagnosticians at the sites in real time according to current guidelines to ensure timely availability of results for patient management. The diagnostic test reports will capture the major findings, including:

1. Presence and extent of CAD (CTA).
2. Resting LV function and perfusion (ECHO and nuclear scans) as either: normal, global dysfunction, regional dysfunction/scar, both, or not interpretable. Ejection fraction will be quantified. This information will be optionally gathered for CTA.
3. Functional capacity determined for exercise ECG, stress ECHO, or stress nuclear.

3.d Test Transfer and Storage

Test transfer will be accomplished by transmission of paper recordings for stress ECGs and the ECG component of ECHO and nuclear studies. For digital images, transmission may be accomplished by compact disc (CD) or over the Web. For Web transmission, an image-transfer system will be deployed at the site. The data-transfer software will provide encryption, lossless compression, and transmission capabilities for the submission of deidentified data sets via the Web to the central Test Data Repository hosted by the American College of Radiology Imaging Network (ACRIN) in Philadelphia.

Upon arrival in the central data repository, datasets will be processed as needed: paper ECGs converted to PDFs; CDs uploaded; and Web images decompressed, deencrypted and imported into the central imaging database, a networked long-term image-archiving and storage system. This will ensure maximal site flexibility as well as seamless and efficient transfer of imaging data from sites into a central data repository and will allow real-time quality control and feedback.

3.e Quality Assurance of Diagnostic Testing

Before beginning enrollment, eligible sites and readers will be qualified by the Diagnostic Testing Coordinating Center (DTCC) based on site and reader surveys, on successful transfer of 1 or more complete data sets with sufficient image quality and completeness for each modality, and participation in the CTA case review. Site qualification will be issued by the DTCC for each modality before subject enrollment.

During the study, technical quality assessment of image and test acquisition will be accomplished on all studies by central repository research technicians trained by the DTCC. This ongoing review will ensure the adequate quality and completeness of data sets (see details and definitions in the MOP) and will monitor radiation exposure in the CTA arm throughout the trial.

Expert overreading of the initial, randomized, noninvasive tests will be performed in approximately 20% of subjects by modality experts in the DTCC. To ensure protocol compliance early on and to account for low-accrual sites, the first 2 studies and ~10% of the remaining studies from each site will be overread in each modality. These will be selected by block randomization stratified by testing site and test modality.

Feedback to Clinical Sites, Remediation, and Disqualification

Sites whose performance does not consistently meet the quality criteria for both quality assurance (QA) and radiation exposure will be asked to undergo protocol review and retraining. Sites that fail to improve may be recommended for discontinuation of enrollment.

3.f Subsequent Medical Care

Care following the imaging studies will be provided by the local care physicians at their discretion. However, they will be encouraged to follow established guidelines for the management of CAD or for primary prevention. To this end, test-information sheets specific to each modality will be created and supplied to each enrolling site and physician and to relevant imaging site staff. These will consist of a brief literature review of diagnostic and prognostic indications of various test results.

In addition, primary and secondary prevention information sheets will be created and provided to each enrolling site and physician at the beginning of the trial. These will consist of brief summaries of relevant ACC/AHA Guidelines and will be referenced to the test-information sheets' results.

3.g Testing Risk and Benefits

To be eligible for entry into the study, each subject and his or her health care provider will have considered the risks and benefits of noninvasive testing and will have determined that the incremental information gained outweighs the potential risks. For example, although diagnostic testing provides important diagnostic and prognostic information and is noninvasive, it cannot be used indiscriminately. Associated risks include maximal exercise testing, use of pharmacologic stress and contrast agents, and radiation exposure on the order of 7 to 17 mSv for stress nuclear and CTA (although aggressive dose-reduction strategies can reduce this to ~2–6 mSv in CTA). Computed tomographic angiography also often involves the use of beta blockers to lower heart rate and an angiographic contrast agent.[79-81] (For comparison, the radiation exposure from a single-view chest x-ray is about 0.03 mSv).

Because the long-term risks and benefits of one form of testing vs. another are unknown, the PROMISE trial is collecting all relevant information to address these knowledge deficiencies, including test complications and estimated total biological radiation exposure for cardiovascular procedures during the duration of the trial. For radiation, doses will be estimated whenever possible using the site's specific scanning protocols for diagnostic testing and fluoroscopic time for invasive procedures. However, if these are unavailable or deemed unreliable, modeled estimates from the sites with such data or from the relevant literature will be used. We will also record the presence of incidental findings as reported on all initial tests and follow-up procedures (including surgical procedures) or testing performed to further evaluate any resulting new diagnoses of significant noncardiac disease.

E Study Procedures

E1 *Screening for Eligibility*

Screening of subjects for this trial will be conducted by the site investigator (or authorized designees) at each participating site. Written, informed-consent documentation will be obtained from each prospective trial subject once study eligibility is confirmed and before the first study procedure.

E2 *Schedule of Assessments*

Subjects will be screened and randomized at or before time of enrollment. Baseline medical history, blood work, and QOL will be assessed. Subsequently, subjects will have either a telephone call or clinic visit at 60 (+/- 14) days for outcome evaluation and recording of any test complications. After that, subjects will be contacted under DOFG supervision by either DOFG or its trained designees at 6 months postrandomization and at 6-month intervals for subsequent follow-up assessments until death, withdrawal, or the end of the trial.

Screening assessments are described below.

TABLE OF ASSESSMENTS	Screening/ Day 1	Day 1-30	Day 60[1]	6 mos	12 mos	18 mos	24 mos	30 mos	36 mos	42 mos	48+ mos
Informed Consent	X										
Confidential Patient Information Form	X										
Medical History	X		X[2]	X[2]	X[2]	X[2]	X[2]	X[2]	X[2]	X[2]	X[2]
Concomitant Medications	X		X	X	X	X	X	X	X	X	X
CV Risk	X			X	X	X	X	X	X	X	X
Pregnancy Test[3]	X										
Creatinine[4]	X										
Resting 12-lead ECG[5]	X										
Quality of Life	X			X	X		X		X		X
Economics	X			X	X	X	X	X	X	X	X
Biomarker Banking	X										
Randomization	X										
Initial Diagnostic Test		X									
Clinical Assessment			X	X	X	X	X	X	X	X	X
Endpoint Assessments			X	X	X	X	X	X	X	X	X
Test Safety Assessment			X								

mos=months; CV=cardiovascular; ECG=electrocardiogram

[1] +/- 14 days
[2] During medical history review, if subjects have received an additional diagnostic test, a cardiovascular procedure or have been hospitalized since the last visit, additional data will be collected from the practice or institution with subject consent

[3] Pregnancy test required only for female subjects of childbearing potential

[4] Creatinine blood draw required only for subjects without a recent normal value (within previous 90 days)

[5] Resting 12-lead ECG required if none available within past 30 days

Day 0/1 Screening/Randomization:

- The subject's relevant medical history (including concomitant medications) will be obtained.
- Coronary arterial disease risk factors will be assessed, including BP/hypertension, PAD (ratio of arm to leg systolic BPs or formal ABI), cerebrovascular disease (carotid bruits), diabetes, cholesterol (LDL, HDL), smoking, family history, sedentary life style, obesity.
- Factors increasing probability of CAD (including diabetes, PVD, and/or other CAD risk factors) will be documented.
- Cardiac symptoms and chest pain descriptors will be assessed.
- Eligible subjects will be asked to give consent for study participation.
- If the subject is of childbearing potential, a pregnancy test will be performed.
- Creatinine blood draw will be done if there is no creatinine measurement within the previous 90 days.

- Subjects will receive a 12-lead resting ECG unless one obtained within the past 30 days is available.
- Subjects will be administered baseline QOL questionnaires at the time of randomization.
- A blood sample will be drawn on subjects participating in biomarker and/or genomic banking (See Section E8).
- The subject will be randomized to the functional test of investigator or care provider choice or CTA. (*Note: functional test of choice must be selected and documented before placing the call to randomize the subject.)*

Days 1-30:

- The functional diagnostic test or CTA will be performed according to the randomized assignment.

Day 60 (+/- 14 days) Postrandomization Follow-up Assessment—site clinic visit or telephone call:

- Relevant interval medical history (including symptoms and concomitant medications) since last assessment will be obtained, including death, MI, major complications from cardiovascular procedure or testing (stroke, major bleeding, anaphylaxis, renal failure), unstable angina hospitalization.
- Test images and stress ECGs from the first test (and the randomized test, if different) will be uploaded to the DTCC. If applicable, the subject's first invasive coronary angiography report and images will be uploaded to the DTCC. All clinical test reports will be sent to the CCC at the DCRI.
- The results of any additional noninvasive tests or invasive catheterizations performed within the first 60 days will be collected.
- Radiation exposure will be assessed.
- Interval resource consumption, including hospitalizations, will be assessed.
- Patient satisfaction will be assessed.

Follow-up at 6 months postrandomization and every 6 months thereafter will be conducted by DOFG staff or its trained designees:

- Relevant interval medical history (including symptoms and concomitant medications) since last assessment will be obtained, including death, MI, major complications from study-related cardiovascular procedures (e.g. catheterization, PCI, CABG, CTA, stress ECHO, stress nuclear) or testing (stroke, major bleeding, anaphylaxis, renal failure), unstable angina hospitalization.
- Interval radiation exposure will be assessed.
- Interval resource consumption, including hospitalizations, will be assessed.
- Cardiovascular risk modification will be recorded.
- QOL questionnaires will be completed at 6 months and 12 months postrandomization and yearly thereafter.

E3 *Safety*

3.a Data and Safety Monitoring Board

A Data and Safety Monitoring Board (DSMB) will be appointed by the National Heart Lung and Blood Institute (NHLBI) to monitor subject safety and to review performance of the protocol. A DSMB charter that outlines the operating guidelines for the committee and the procedures for the interim evaluations of study data will be developed by the NHLBI and agreed upon at the initial meeting of the DSMB. Reports will be prepared regularly by the DCRI as requested by the DSMB chair. Depending upon the operational plan established by the DSMB, the report might include recruitment and retention rates, interim analyses, primary and secondary endpoints, and other information as requested by the committee. After each meeting, the DSMB will make recommendations to the NHLBI and the trial leadership about the continuation of the study.

3.b Study Coordinating Center or Food and Drug Administration Notification by Investigator

No reporting (other than through the InFORM system) is required.

3.c Definitions of Safety Events and Reporting

Because the only intervention in the trial is the randomized assignment of the initial test to be performed in a symptomatic subject with clinically indicated testing, the only safety events arising from the study are related to the initial test. Mild safety events are considered related to testing only up to 24 hours after the initial randomized test and will be collected and reported by site personnel. The site investigator/designee will document the safety events listed below occurring within 24 hours of initial testing and report them in the InFORM system.

In contrast, those severe events/complications related to cardiovascular testing or cardiovascular procedures that are also trial endpoints (e.g. peri-procedural MI, major bleeding, renal failure, and anaphylaxis requiring circulatory or respiratory support) will be collected throughout the duration of the trial and will be considered to be related to testing or a procedure if occurring within 72 hours.

For CTA:

1. Mild contrast reaction such as rash and hives (severe reactions including anaphylaxis or death are part of the primary endpoint)
2. Extravasation of contrast into the surrounding tissue of the extremity where the intravenous line was placed and contrast administered
3. Hemodynamic instability, including symptomatic bradycardia or hypotension, due to the beta blockade or nitrates given for the CTA scan acquisition
4. Acute bronchospasm due to the beta blockade given for the CTA scan

For exercise testing during exercise ECG, stress ECHO, or stress nuclear:

1. Exercise-induced hypotension with systolic BP fall greater than 20 mm Hg
2. Stress-induced symptoms that do not resolve within 20 minutes
3. Rapid atrial fibrillation that does not slow or convert with treatment

4. Ventricular tachycardia
5. Hemodynamic instability, defined as systolic BP less than 80 mm Hg
6. Hospital admission not otherwise captured by the primary endpoint, including that precipitated by any symptomatic event (chest pain, dyspnea, etc.), persistent or worsening ischemic ECG changes, any bradycardic or tachycardic arrhythmia, or any hemodynamic changes (hypertension or hypotension)

For stress nuclear:

1. The above events for exercise testing
2. Any events potentially related to the use of vasodilators such as dipyridamole or adenosine, including an anaphylactic reaction to contrast agent not requiring circulatory or respiratory support

For stress ECHO:

1. The above events for exercise testing
2. Stress-induced wall motion abnormality that does not resolve within 20 minutes (despite treatment)
3. Any anaphylactic reaction to contrast agent not requiring circulatory or respiratory support

E4 *Study Outcome Measurements and Ascertainment*

All-cause Mortality

All-cause mortality is used rather than cardiac mortality to eliminate the need for possibly difficult adjudication of causes of death, especially given the relatively low mortality expected.

Myocardial Infarction

Defined as either 1) an abnormal cardiac biomarker level (either troponin or CK-MB) greater than institutional upper limit of normal (ULN), and either ischemic discomfort lasting greater than 10 minutes or ECG changes indicative of ischemia or infarction, or 2) new abnormal Q waves consistent with infarction. Additionally *peri-procedural infarctions* are defined as greater than 3x ULN for serum CK-MB for PCI and greater than 5x ULN for CABG.

Unstable Angina Hospitalization

Defined as 1) ischemic discomfort or equivalent symptoms requiring hospitalization within 48 hours of symptoms, 2) lasting greater than or equal to 10 minutes at rest, or in an accelerating pattern, 3) accompanied by dynamic ST depression, ischemia on stress testing, or significant epicardial coronary artery stenosis, and 4) considered to be myocardial ischemia upon final diagnosis.

Major Complications From Cardiovascular Procedures and Diagnostic Testing That Occur Within 72 Hours

Defined as:

- Stroke is defined as an acute focal neurological deficit of sudden onset, not reversible within 24 hours, or that resolves in less than 24 hours with clear evidence of a new stroke on cerebral imaging.
- Bleeding is defined as major based on 1 or more of the following:
  - Transfusion of greater than or equal to 2 units heterologous packed red blood cells or whole blood
  - Decrease in hemoglobin level by greater than or equal to 2.0 g/L
  - Need for reoperation or invasive intervention (*e.g.* evacuation of wound hematoma)
  - Bleeding at a critical anatomic site (intracranial, intraspinal, intraocular, retroperitoneal, intraarticular, pericardial, or intramuscular with compartment syndrome)
- Renal Failure is defined as new requirement for renal replacement therapy.
- Anaphylaxis is defined as a severe contrast reaction requiring emergency respiratory and/or circulatory support.

Radiation Exposure Safety Endpoint

Cumulative radiation exposure will be collected as follows:

- CTA: the actual administered dose (computed tomography dose index volume and dose length product for CTA) will be recorded by sites and confirmed by extracting information directly from the images.
- Nuclear imaging: the injected/administered contrast agent dose will be recorded and standard tables used to convert into equivalent doses for appropriate comparison with CTA.
- Invasive coronary angiography and intervention: the actual administered radiation dose or fluoroscopy time will be recorded by sites and standard tables used to convert into equivalent doses for appropriate comparison with CT.

These measures will ensure accurate calculation of the actual cumulative radiation exposure in each arm for tests performed for the diagnostic work-up during the 60 days following enrolment. In addition, we will capture all cardiac diagnostic testing involving radiation performed during the entire follow-up period (CTA, nuclear cardiology, catheterization) and will estimate cumulative radiation exposure over the entire trial using our original data collection (average dose per test for each site) to extrapolate radiation exposure during follow-up.

E5 *Independent Clinical Event Adjudication Committee*

An independent clinical event adjudication committee will review and adjudicate all primary endpoint events in a blinded fashion based on the definitions presented above. The clinical secondary endpoint events and cumulative radiation exposure will not be adjudicated.

Primary outcome events will be documented through 60 (+/- 14) days after randomization by the site Investigator or authorized designees. After that, the DOFG or its designees will be responsible for documenting study events from 6 months until the end of the study.

E6 *Quality-of-life Assessments*

Baseline QOL interviews will be administered to subjects by site personnel as soon as possible after consent, preferably before the subject's randomization. Completed questionnaires will be sent directly to the EQOL CC for data processing. Follow-up QOL questionnaires will be administered to subjects via structured telephone interview by DOFG trained and supervised interviewers at 6 and 12 months postrandomization and annually thereafter until the end of the study. Proxy QOL questionnaires will be used when a subject has died in the follow-up interval or has become incapacitated; these questionnaires will include items that can be reliably obtained from a relative, caretaker, or medical record. Proxy questionnaires will be used if the subject is unable to participate in follow-ups via telephone interview.

Content of Health-related Quality-of-life Questionnaires

A battery of validated instruments will be used that build on a disease-specific core supplemented with generic measures to provide a comprehensive but brief assessment of health-related QOL.

Chest-pain–specific symptoms will be measured using the **Seattle Angina Questionnaire (SAQ)**, a 19-item instrument that assesses 5 dimensions of the impact of chest pain on QOL: physical limitations, angina stability, angina frequency, treatment satisfaction, and disease perception. The **Duke Activity Status Index (DASI)**, a 12-item scale that has been validated in cardiac patients against maximal oxygen uptake measured at exercise (VO_2 max), will be used as a disease-specific functional status assessment. The 4-item **Rose Dyspnea Scale** will be used to assess patients' levels of dyspnea with common activities.

The generic core instrument to be used is the **Medical Outcomes Study Short Form (SF-12)**. The SF-12 is composed of 8 scales (physical function, role function–physical, role function–emotional, general health, bodily pain, social function, psychological well-being/mental health, and vitality), a health transitions item, and 2 summary scores. Additionally, the entire scales for general health, psychological well-being, vitality, and social functioning from the SF-36 health survey will be used to provide better resolution of any treatment differences in these domains.

To assess effects of the 2 diagnostic strategies on the prevalence of depression, we will employ the **Patient Health Questionnaire (PHQ)**, a 9-item depression scale that has demonstrated good agreement with the clinical diagnosis of depression.

Patient-specific utilities will be assessed using the **EuroQoL-5D (EQ-5D)**, a standardized generic health-status measure that links specific health states to general population-based utilities. The EQ-5D consists of a 5-dimension health-state

assessment, which allows for definition of 243 discrete health states that can be mapped to population utility weights and a self-rating (0-100) "thermometer" of current health-related QOL. The EQ-5D will be collected as part of all QOL questionnaires.

Employment/productivity will include time lost from work and reduced productivity while at work as measured by the 6-item **Stanford Presenteeism Scale** and questions adapted from the NHLBI Bypass Angioplasty Revascularization Investigation Substudy in Economics and Quality of Life.

E7 *Economic Assessments*

Resource-use data to be collected on the study case report form (CRF) will include hospitalizations, emergency department visits, selected cardiac procedures, and tests. Hospital bills (detailed, summary ledger, and UB 04) will be collected by the EQOL CC at the DCRI for all hospitalizations identified throughout the length of the study. They will include care at clinical sites and at institutions not participating in PROMISE. In addition, cost-to-charge ratios will be obtained from each hospital where a PROMISE follow-up hospitalization is reported.

E8 *PROMISE Biorepositories*

8.a Imaging and Electrocardiograms

Since future developments in image interpretation and integration with clinical data will remain important in the diagnosis and management of chronic diseases of CAD, PROMISE will create a unique anonymized “Image and ECG Data Warehouse” with all initial noninvasive test images, ECG, and initial catheterization films, which will be linked to clinical information. Collection and release of data and images will be at the discretion of the NHLBI and is included as part of the subjects’ initial consent.

8.b Blood Biomarkers and Genomics

The PROMISE investigators also believe that an integrated approach to disease characterization and therapeutic responses will play an increasingly important role in the diagnosis and management of chronic diseases such as CAD. Accordingly, subjects will be asked to provide a blood sample for deposit into a biomarker repository at the time of randomization for future assessment of advanced molecular biomarkers (plasma, serum) such as troponin and high-sensitivity C-reactive protein relevant to disease characterization, risk stratification, characterization of treatment response, and adverse effects. In addition, subjects will be requested to separately consent to allow use of the biorepository sample for genetic testing (DNA).

F Statistical Plan

F1 *Sample Size Determination and Statistical Power*

Several design factors and research objectives were considered in selecting the target sample size for the study. First, the number of subjects was determined so there would be a sufficient number of endpoints to provide a high degree of power (greater than or equal to 90%) for testing the primary superiority hypothesis. Second, the statistical power for secondary endpoints was considered, including the composite clinical endpoints and the economic and QOL endpoints. Third, it was considered important for the sample size to be large enough to permit a prudent examination of diagnostic testing effects in selected subgroups of subjects where anatomic testing might be particularly advantageous or where the question of a benefit from CTA is particularly relevant. Important prespecified subgroups of interest in this study include those defined by age, sex, race, comorbidity, cardiovascular risk factors, the prerandomization choice of functional test, and characteristics of the precipitating symptoms. Fourth, the sample size was selected to provide a reasonable level of confidence for detecting clinically important outcome differences between the anatomic and functional testing strategies even if current projections of event rates and the hypothesized differences in clinical outcomes between the 2 arms prove to be optimistic. A fifth consideration was the adequacy of the sample size for assessing noninferiority of anatomic testing compared with functional testing in the event that the anatomic testing strategy is not demonstrated to be statistically superior to functional testing. Finally, although the study objectives are expressed in terms of testing specific hypotheses (i.e., that the anatomic testing strategy is superior [and if not superior, then noninferior]) to functional testing, another important objective of the trial is to estimate the magnitude of the difference in outcomes to within an acceptable level of statistical precision, regardless of whether either testing strategy is proven to be superior. Thus, the precision of the estimated difference in outcomes between the 2 arms of the trial (i.e., width of the confidence interval [CI]) has been considered in addition to the statistical power for the hypothesis tests.

Based on the distribution of coronary disease expected in this patient population (approximately 15% obstructive disease [i.e., greater than or equal to 50% stenosis of the left main coronary artery or greater than or equal to 70% stenosis of 1 or more of the other major epicardial coronary arteries], 40% nonobstructive disease, and 45% normal coronary arteries) and based on national claims data or published information from other databases, the event rate at 2.5 years (the average length of follow-up in PROMISE) for the primary composite endpoint in subjects randomized to the functional testing strategy was projected to be approximately 9%.

With this event-rate projection in the functional testing arm, a key driver of the sample size is the magnitude of benefit that can reasonably be expected to be achieved with the anatomic testing strategy. This determination requires careful consideration of multiple characteristics of CTA, including its likely incremental diagnostic and prognostic accuracy and its ability to more effectively detect nonobstructive CAD. A careful assessment of the impact of the advantages of CTA with respect to these test characteristics translates to a projection (hypothesis) that CTA will reduce the primary composite endpoint by 20% (from 9% to 7.2% at 2.5 years).

Based on the event rates for each arm discussed above, sample-size requirements were formulated to provide high power for detecting the postulated 20% relative risk reduction. Recognizing, however, that the actual event rates and the outcome differences between

the 2 testing strategies in PROMISE may vary somewhat from these estimates, sample-size requirements were calculated for several different combinations of event rates, effect sizes, and power levels in order to examine the sensitivity of the sample size to different event rates and outcome scenarios that might conceivably arise in this trial.

Since the primary treatment comparisons in this study will be based on time-to-event methodology using the log-rank test[82]or equivalently, the Cox proportional hazards model,[83] the approach used for calculating sample-size requirements for PROMISE was based on the sample-size methodology for the proportional hazards regression model outlined in Schoenfeld.[84]

To provide an adequate number of subjects for the trial that will be relatively robust in providing (1) excellent statistical power under various assumptions about the event rates in the functional testing arm and the magnitude of the benefit of anatomic testing compared with functional testing for reducing the primary endpoint, (2) adequate power for selected secondary endpoints, (3) adequate power for assessing noninferiority in the event that superiority is not demonstrated, and (4) a relatively high degree of precision for estimating the true effect of an anatomic vs. functional testing strategy regardless of whether the final result has a *P* value that is statistically significant, the study will enroll 10,000 subjects (5,000 per arm). This number will provide greater than 90% power for detecting a 20% reduction in the primary clinical endpoint if the 2.5-year event rate in the functional testing arm is 8% or higher and 80% power if the event rate is as low as 6%. This number of patients will also provide adequate power for detecting a smaller (16%–17%) reduction if the event rate in the functional testing arm is 8% to 9% or higher and acceptable power for selected secondary endpoints, allowing for up to a 3% loss to follow-up. Ten thousand patients will also provide 90% power for testing noninferiority with a margin of 1.10 (expressed as a hazard ratio of CTA vs. functional testing) if the functional testing arm event rate is 9%, 86% power if the event rate is 8%, and 81% power if the event rate is 7%, assuming that anatomic testing is only better than functional testing by 10%, an assumption that was felt to be reasonable for the noninferiority assessment. That is, we will have excellent power for demonstrating that anatomic testing is not worse than functional testing by more than 10% under these various assumptions.

In summary, 10,000 patients will provide excellent and robust statistical power for assessing clinically relevant and realistic outcome differences between the 2 testing strategies being studied in this trial.

F2 *Statistical Analysis Plan*

Statistical analysis will be performed at the PROMISE SDCC at DCRI. All major treatment comparisons between the randomized groups will be performed according to the principle of "intention-to-treat"; that is, subjects will be analyzed (and endpoints attributed) according to the diagnostic testing strategy to which subjects were randomized, regardless of subsequent additional testing or postrandomization treatment and medical care. Statistical comparisons will be performed using 2-sided significance tests. Additional perspective regarding the interpretation of the data will be provided through extensive use of CIs[85] and graphical displays.

2.a Analysis for the Primary Endpoint

The statistical comparison of the 2 randomized arms (anatomic vs. functional diagnostic testing) with respect to the primary composite endpoint (death, MI, major peri-procedural complications, or hospitalization for unstable angina) will be a “time-to event” analysis and therefore will be based on the time from randomization to the first occurrence of any of the components of the primary composite endpoint. The Cox proportional hazards model will be the primary analytic tool for assessing outcome differences between the 2 randomized arms.[83] To appropriately account for heterogeneity among the subjects, the overall comparison will be adjusted for a selected set of prognostically important baseline covariates that will be carefully defined and prespecified in the statistical analysis plan. The level of significance for the assessment of the primary endpoint will be α=0.05.

In addition to the statistical hypothesis testing, Kaplan-Meier “survival” (or event-free) estimates[86] will be calculated for each randomized arm as a function of follow-up time to display the event rates graphically. A hazard ratio and 95% CI for descriptively summarizing the difference in outcome between the 2 study arms will be computed using the Cox model.[83]

If the data provide evidence of an overall difference in outcome between the randomized arms, an assessment will be made of whether the effect is similar for all patients or whether it varies according to specific patient characteristics. In particular, this analysis will focus on whether the relative benefit differs according to subject age, sex, race, comorbidity, selected risk factors, characteristics of the precipitating symptoms, and the prerandomization specification of the functional test that would be used if the subject was randomized to the functional testing arm. This latter factor is built into the randomization scheme. For subjects for whom the prerandomization choice for functional testing (if the subject was assigned to the functional testing arm) was stress nuclear imaging (for example), the outcomes of these subjects in the CTA arm will be compared to the outcomes of corresponding subjects randomized to functional testing. In this way, CTA can be compared with stress nuclear imaging, as well as with each of the other functional testing modalities with the benefits of randomization in each comparison maintained by virtue of the stratified randomization scheme. These analyses will utilize the Cox model by testing for interactions between the randomized testing strategy and these specific baseline variables. In addition to the formal assessment of testing strategy by covariate interactions, effects of the diagnostic testing strategy characterized by a hazard ratio and 95% CI will be calculated and displayed for prospectively defined subgroups of subjects defined by the variables listed above. These descriptive hazard ratios will be carefully interpreted in conjunction with the formal interaction tests.

If the data do not provide statistical evidence that the CTA testing strategy is superior to functional testing with respect to the primary endpoint, a test for noninferiority of the anatomic testing strategy will be performed. This assessment will be based on a noninferiority margin of 1.10 (expressed as a hazard ratio for CTA vs. functional testing). The noninferiority assessment will be performed by comparing the upper limit of the 95% CI for the hazard ratio with the noninferiority margin. If the upper limit of the CI falls below 1.10, noninferiority will have been demonstrated. We emphasize that the superiority hypothesis will be assessed first, and if significant, the noninferiority assessment will not be performed. Only if superiority is not demonstrated will the noninferiority analysis then be performed.

2.b Analysis of Secondary Endpoints

Secondary endpoints that will be evaluated include (1) a composite endpoint consisting of death, MI, or hospitalization for unstable angina; (2) death or MI; (3) major peri-procedural complications (stroke, major bleeding, renal failure, anaphylaxis); (4) resource-use patterns, medical care costs and incremental cost effectiveness; and (5) QOL. In addition, major adverse cardiac events (events other than the endpoints listed above) will be monitored and reported.

The analysis of secondary endpoints 1 through 3 will be similar to that outlined for the superiority assessment of the primary endpoint, using time from randomization until the first occurrence of any component of the specific secondary endpoint (or censoring) as the response variable, and assessing group differences using the Cox proportional hazards model. The effect of the diagnostic testing strategy on these secondary clinical endpoints will be descriptively summarized using hazard ratios (with associated CIs) computed from the Cox model. Kaplan-Meier curves will be computed to graphically display the cumulative event rates of the 2 randomized arms as a function of time from randomization. We note that the analysis of secondary endpoint (3) above (major peri-procedural complications) will have to be interpreted cautiously, particularly if there should be a higher death rate in one arm compared with the other. To deal with this complexity and clarify and enhance the interpretation of this comparison, the analysis of this endpoint will be supplemented with further analyses by considering major peri-procedural complications and death as a combined endpoint.

Plans for the analysis of the QOL and economic endpoints are addressed below in Sections F2.g and F2.h.

2.c Analysis of Diagnostic Testing Core Data

The DTCC will implement robust QA programs to ensure uniformity and high-quality testing in support of the primary aims of PROMISE.

Two important components of information from the diagnostic test QA activity will be analyzed by the SDCC. The DTCC will review every subject’s initial diagnostic test for technical quality, and each test will be assigned a quality assessment using an ordinal categorical scale (for example, excellent, good, fair, poor, uninterpretable). Using simple frequency counts, the SDCC will tabulate a description of the distribution of this scale on an ongoing basis by type of test and by testing site in order to provide feedback to the sites on their performance, to inform the study leadership and the DSMB as to the quality of the diagnostic testing, and to flag potential problem areas, whether by testing site or across a given testing modality, for remedial attention. This quality measure will be compared among the different testing modalities using rank-based tests (e.g., Kruskal-Wallis nonparametric analysis of variance) and ordinal logistic regression to characterize, describe, and assess any differences in overall quality among the various testing modalities. In particular, the technical quality of the anatomic (CTA) tests will be compared with the quality of the functional tests. This comparison may be helpful in interpreting the comparisons of the 2 randomized arms with respect to the clinical outcomes.

The second important component of information from the diagnostic test QA activity is the quality of test interpretations. Approximately 20% of tests will be overread by the DTCC using a categorical level of coronary disease risk. The site interpretation of level of risk will be compared with the level of risk using the same scale assessed by the core

lab's overread of the test, and overall summary statistics of the agreement between the site and core lab assessments will be computed. Raw proportions of agreement (perfect agreement, and agreement differing by less than or equal to 1 category of risk) will be tabulated, and Kappa statistics will be used to characterize the level of agreement. The Kappa statistics are "chance corrected" (i.e., adjusted for agreement due to chance), and both unweighted and weighted Kappa statistics will be used in these analyses since more substantial disagreements in the assessment of risk for a given subject would be more serious than small disagreements. These agreement statistics will then be compared across the different testing modalities to determine whether the level of agreement between the site readings and the core lab readings varies with the type of test.

2.d **Analysis of Diagnostic Accuracy**

Although PROMISE is designed with the primary objective of evaluating initial anatomic vs. functional testing strategies with respect to clinical outcomes and thus represents a different paradigm than the traditional design to assess diagnostic accuracy, performance of supplementary analyses of diagnostic accuracy are prespecified. This cannot be done in a conventional manner as not all subjects will undergo invasive angiography (the "gold standard"), and those who do will not undergo angiography by random selection. Instead, the decision to verify disease will be based on test results and other characteristics of the subject. This nonrandom selection process will likely result in a strong verification bias,[87] often characterized by higher than true sensitivity and lower than true specificity results. (Of note, the trial cannot require angiography in a subset of subjects for purposes of determining testing accuracy, as this would provide additional information to these subjects' physicians and care givers above and beyond that provided by the randomized testing strategy, and therefore invalidate the trial results.) Correction of the verification bias is possible if the process leading to verification with angiography is known. However, this is rarely achievable, but the process can be modeled under the missing at random (MAR) assumption,[88] namely that disease status affects referral to angiography only through measured covariates and not the disease status itself. In view of the inherent limitations, the following steps will be taken with respect to evaluating diagnostic accuracy.

Accuracy rates will be assessed using conventional measures (sensitivity, specificity, and receiver operating characteristic [ROC] curves) in subjects undergoing cardiac catheterization, bringing to bear where applicable the latest statistical methods for dealing with verification bias. The probability of verification will be modeled with a logistic regression model using covariates that are predictors of referral to angiography. Corrected values of sensitivity, specificity, and the ROC curve will be estimated.[88,89] Assessment of the impact of potential departures from the MAR assumption on sensitivity and specificity will utilize the "test ignorance region" approach.[88] Alternatively, we will use the clinical outcome (rather than coronary angiography) as the "gold standard," such that measures of diagnostic accuracy, including time-dependent ROC curves, will be computed to describe and statistically compare the 2 arms of the trial with respect to these measures. A subgroup analysis of diagnostic accuracy will be performed comparing results at sites with high volume and extensive experience or expertise in diagnostic testing with results in less-experienced or lower-volume sites.

Imaging prognostic performance will also be evaluated by comparing the association of test results with subsequent clinical events, including the primary endpoint of the trial.

Finally, as described in Section F2.c above, concordance between the site interpretations of studies and core lab interpretations will be assessed.

We emphasize that all of these analyses of diagnostic accuracy will be strictly supplementary to the analysis of the primary and secondary clinical endpoints outlined in Sections F2.a and F2.b above.

2.e Analysis of Safety Events, Morbidity, Radiation Dose, and Incidental Findings

The frequency with which major safety cardiac events occur (events other than the primary and secondary endpoints) will be carefully tabulated and descriptively summarized. Statistical comparisons of the randomized arms with respect to these events will use chi-square or other appropriate 2-sample methods, depending on the nature of the event, interpreting such comparisons in the context of differences between the 2 randomized arms in the primary and major secondary clinical endpoints and bringing to bear clinical judgment as to the relative seriousness of these events.

Of particular interest in this trial is the amount of radiation exposure to which subjects are subjected in each of the randomized arms of the trial. Radiation exposure for the various tests will be collected as elements of the electronic CRF (eCRF) and through the documentation of radiation exposure submitted to the DTCC. All cardiac diagnostic testing involving radiation exposure (e.g., CTA, stress nuclear, cardiac catheterization) performed during the entire follow-up period will be captured so that cumulative radiation exposure for each patient can be calculated. The distribution of radiation exposure in each arm will be summarized (using medians and percentiles) and compared between the randomized arms and different testing modalities using the Wilcoxon rank-sum test.

Incidental findings (e.g., such things as lung nodules) that may be discovered with the anatomic (CTA) testing strategy in contrast to the functional testing modalities will be captured as part of the data collection and tabulated descriptively.

2.f Assessment of Prognostic Factors

With the large database of information that will be collected on the 10,000 subjects enrolled in this study, extensive regression modeling analyses will be performed, using primarily the Cox regression model, to identify and assess the factors (predictors) that are associated with the clinical outcomes of these subjects. These analyses will comprehensively evaluate the strength and shape of the relationships of numerous clinical factors with the clinical outcomes. While these analyses are more exploratory than the rigorous prespecified primary and secondary comparisons of the randomized arms of the trial, they will nonetheless be helpful in elucidating relationships and identifying the key factors that impact patient outcomes and any observed differences in outcomes between the diagnostic testing arms.

2.g Quality-of-life Analyses

For each of the QOL measures, data analysis will proceed in several stages. We will start by providing simple descriptive and comparative analyses by intention-to-treat. A nonparametric bootstrap will be used to estimate treatment differences with 95% CIs and *P* values. Because there is currently no consensus in the statistical literature about the best way to deal with the multiple comparisons problem arising from testing each individual scale at each time point separately, we propose 2 complementary

approaches. First, we will prespecify the angina frequency and disease perception/QOL scales from the SAQ as the CAD-specific measures of primary interest, since these measures most directly quantify the therapeutic goal of coronary diagnosis and therapy: to minimize symptoms and optimize patients' QOL. We will also specify cardiac functional status measured with the DASI as a primary outcome measure of interest. Other disease-specific and generic QOL measures will be assigned to a secondary (descriptive) status in our analyses. Second, we will fit a mixed effect longitudinal proportional odds model[90] that makes use of all available QOL data at each study assessment point to model the time profile (fixed effect) using a restricted cubic spline function. Using the fitted model, we can estimate the overall difference in the QOL measures as well as test the global hypothesis of no difference over time. We can also estimate the difference in the areas under the 2 QOL treatment curves (and test the hypothesis of no difference on average). In addition, we can estimate differences in QOL at the end of the study or at intermediate points such as at 1 year. Statistical power estimates for this part of our analysis show that we should have in excess of 90% power to detect ¼ standard deviation differences in the 3 principal QOL endpoints.

2.h Economic Analyses

The health economic analyses for PROMISE will consist of 2 major parts, namely an empirical intention-to-treat cost comparison and a cost-effectiveness analysis. Primary statistical comparisons between the 2 treatment arms of empirical costs will be performed by intention-to-treat analysis. Confidence limits around the observed cost differences will be constructed using bootstrap methods.

The cost-effectiveness analyses will estimate the incremental cost required to add an extra life year with the investigational anatomic arm relative to the control functional testing arm. In secondary analyses, we will incorporate utility weights to estimate the incremental cost per quality-adjusted life year gained with the CTA anatomic strategy, relative to the functional testing strategy. These analyses will be conducted from a societal perspective and will use a lifetime time horizon so that the estimated incremental cost-effectiveness and cost-utility ratios can be compared with societal benchmarks. We will also calculate within-trial cost-effectiveness/cost-utility ratios, although these ratios are limited in their value due to their failure to account for long-term benefits and costs and the absence of comparative benchmarks. Costs will be adjusted for inflation, and both costs and life expectancy will be discounted to present value at a 3% annual discount rate. Adjustments for censored data due to staggered entry will be made following the approach of Bang and Tsiatis.[90] Extensive sensitivity analyses will be performed.

F3 *Interim Analyses*

For safety and ethical reasons, interim examinations of key safety and endpoint data will be performed at regular intervals during the course of the trial. The primary objective of these analyses will be to evaluate the accumulating data for an unacceptably high frequency of negative clinical outcomes in either of the 2 randomized arms. In addition, the interim monitoring will also involve a review of subject recruitment, compliance with the study protocol, status of data collection, and other factors that reflect the overall progress and integrity of the study. The results of the interim analyses and status reports will be carefully and confidentially reviewed by the NHLBI-appointed DSMB. The DSMB will meet at approximately 6-month intervals to review the accumulating data.

To properly account for the repeated interim testing in PROMISE, a group sequential method similar to that proposed by O'Brien and Fleming[91] will be used as a guide for interpreting the interim analyses. This procedure requires large critical values early in the study, but relaxes (i.e., decreases) the critical value as the trial progresses. Because of the conservatism early in the trial, the critical value at the final analysis is near the "nominal" critical value. The actual method for this interim monitoring that will be employed in PROMISE is the "spending function" approach to group sequential testing developed by Lan and DeMets.[92] The Lan-DeMets approach only requires specification of the rate at which the Type I error (which in this trial will be α=0.05 for the primary endpoint) will be "spent." This procedure allows "spending" a portion of α at each interim analysis in such a way that at the end of the study, the total Type I error does not exceed 0.05. One such spending function generates boundaries that are nearly identical to the O'Brien-Fleming[91] boundaries. It is this approach that will be used in PROMISE, namely 2-sided, symmetric O'Brien-Fleming[91] type boundaries generated using the flexible Lan-DeMets[92] approach to group sequential testing. Since the number of looks and the increments between looks need not be predetermined, it allows considerable flexibility in the monitoring process for accommodating additional comparative examinations of the data in response to concerns of the DSMB that may arise during the course of the trial.

The analytic approach that will be used at the interim analyses for assessing outcome differences between the randomized arms will be the time-to-event analysis methods described previously, except that interpretation of statistical significance will be guided using the group sequential monitoring boundaries outlined above. At each interim analysis, the monitoring boundaries will be calculated using the fraction of the total number of primary events expected by the end of the trial.

Judgment concerning the continuation or termination of the study will involve not only the degree of statistical significance observed at the interim analyses, but also careful consideration of many other factors reflecting the progress and integrity of the trial, including how well event rates in the functional testing arm are tracking with the rates considered in the power calculations. As an aid in the interim assessments, the SDCC will supplement the group sequential analyses outlined above with calculations of conditional power, namely the conditional probability that a particular statistical comparison will or will not be significant at the end of the trial at the α level used in the design, given the hypothesized outcome difference between the randomized arms and the data observed to date. After a sufficient number of primary events have occurred (approximately 50% of the total projected events), conditional power for the primary treatment comparison will be computed and provided to the DSMB as a regular part of the interim study reports.

It should be emphasized that regardless of whether CTA demonstrates a statistically significant benefit compared with functional testing, the results of the study will be important to future clinical practice. Indeed, a nonstatistically significant effect for the primary endpoint is not necessarily a negative result in this study. The range of experience and expertise of the DSMB will enable them to put all important considerations into proper perspective, including the precision of estimates of clinical outcome differences between the testing strategies, and make well-reasoned recommendations to the NHLBI regarding continuation of the study.

G Data Handling and Record Keeping

Study Data Collection—All Subjects

The full study dataset will be collected for subjects who enter the randomized phase of the study. The primary data collection system for PROMISE will use the electronic data capture system InForm™.

Data Management and Quality

Any out-of-range values and missing or inconsistent key variables are flagged and addressed/answered at the site in real time during the data entry process. When a query is generated on a particular variable, a flag is set in a field in the database, enabling the system to track the queries and produce reports of outstanding queries. Queries can also be generated from manual review of the data forms. These queries will be entered into the database and tracked in the same manner as the computer-generated queries. At regular intervals, all data will be transferred from InForm™ to SAS for statistical summarization, data description, and data analysis. Further cross-checking of the data will be performed in SAS and discrepant observations flagged and appropriately resolved through a data-query system.

The SDCC will perform internal database quality-control checks and data audits throughout the course of the trial.

G1 *Confidentiality and Security*

All study data will be stored in locked, secure locations. Computerized data are accessible only by password, and a centralized monitoring system records and reports all access to data. The DCRI computer network is protected by a firewall. Electronic CRFs will be identified by study number only, to ensure subject anonymity. No subject identifiers will be used in the presentation of data. Study records that might identify subjects will be kept confidential as required by law. Except when required by law, subjects will not be identified by name, social security number, address, telephone number, or any other direct personal identifier in study records. This information will be retained by each individual center and will not be disclosed to the coordinating center except as needed for DCRI centralized clinical, QOL, and economic follow-up of the subjects. Subjects will be informed that the study physician and his or her study team will report the results of study-related tests to the Coordinating Center and to the National Institutes of Health (NIH). Subjects will be informed that their records may be reviewed in order to meet federal or state regulations. Reviewers may include the Food and Drug Administration, IRBs/IECs, or the NIH. Subjects will be informed that if their research record is reviewed, their entire medical record may also need to be reviewed. If an adverse event occurs, management of the event and subsequent care will be according to appropriate care practices at that site and will be implemented under the direction of the treating physician. All of the tests used in this study currently represent a reasonable standard of care for the subject population as demonstrated by ACC/AHA practice standards.

G2 *Training*

All investigator staff authorized to enter PROMISE Study data will receive training on the InForm™ system.

G3 *Electronic Case Report Form*

This study will use Web-based e-CRFs developed through a validated, Electronic Record, Electronic Signatures-compliant platform (21 CFR Part 11). Data will be entered into the InForm™ eCRF by authorized Investigator personnel, ACRIN, and the diagnostic testing core labs.

G4 *Records Retention*

Study records will be maintained by the site investigators for a period of 6 years following the expiration of the grant or length of time as required by local regulations.

H Study Monitoring, Auditing, and Inspecting

H1 *Study Monitoring Plan*

See E3.a

H2 *Auditing and Inspecting*

National Heart Lung and Blood Institute personnel or their designees may perform an audit at any time during or after completion of this study. All original study-related documentation will be made available to the designated auditor as required. A representative of the NIH or other government agency may choose to inspect a study center at any time before, during, or after completion of the clinical study. All pertinent original study data will be made available to responsible regulatory authorities for verification, audit, or inspection purposes.

I Study Administration

I1 *Organization and Participating Centers*

The PROMISE trial will be conducted under the following principal investigator (PI) leadership: Dr. Pamela Douglas as the overall and CCC PI, Dr. Kerry Lee as the SDCC PI, Dr. Daniel Mark as the EQOL CC PI, and Dr. Udo Hoffmann at Massachusetts General Hospital as the DTCC PI. The organization of the **PROMISE** trial and interrelationships between the NHLBI, the CCC, the SDCC, the various committees, and the clinical sites are outlined in the figure below.

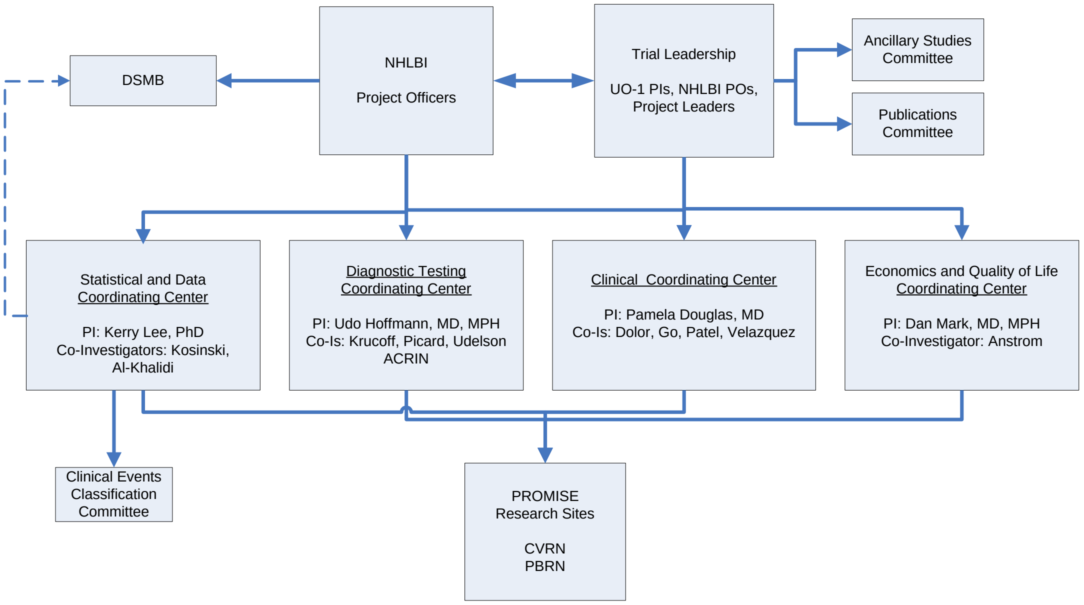

I2 *Funding Source*

The PROMISE trial is funded by the National Heart Lung and Blood Institute, National Institutes of Health.

I3 *Study Timetable*

The PROMISE timeline includes a 9-month start-up period, followed by approximately 42 months of enrollment, approximately 30 months of follow-up after the last subject is enrolled, and 6 months of closeout and data analysis.

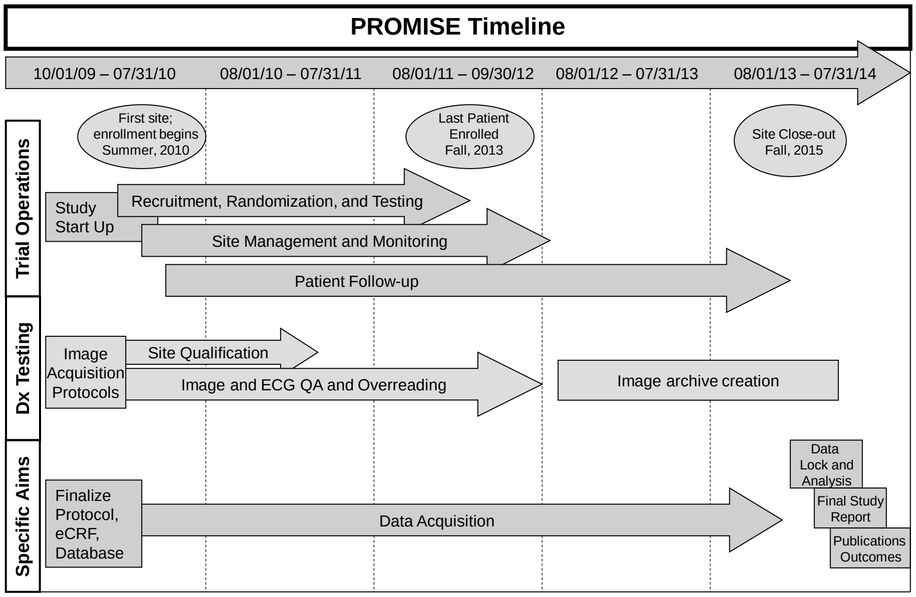
PROMISE Timeline
10/01/09 – 07/31/10
08/01/10 – 07/31/11
08/01/11 – 09/30/12
08/01/12 – 07/31/13
08/01/13 – 07/31/14
Trial Operations
First site; enrollment begins Summer, 2010
Last Patient Enrolled Fall, 2013
Site Close-out Fall, 2015
Study Start Up
Recruitment, Randomization, and Testing
Site Management and Monitoring
Patient Follow-up
Dx Testing
Image Acquisition Protocols
Site Qualification
Image and ECG QA and Overreading
Image archive creation
Specific Aims
Finalize Protocol, eCRF, Database
Data Acquisition
Data Lock and Analysis
Final Study Report
Publications Outcomes

J References

1. Rosamond W, Flegal K, Furie K, et al. Heart disease and stroke statistics--2008 update: a report from the American Heart Association Statistics Committee and Stroke Statistics Subcommittee. *Circulation*. 2008;117(4):e25-146.

2. Selker HP, Zalenski RJ, Antman EM, et al. An evaluation of technologies for identifying acute cardiac ischemia in the emergency department: a report from a National Heart Attack Alert Program Working Group. *Ann Emerg Med*. 1997;29(1):13-87.

3. Hemingway H, McCallum A, Shipley M, et al. Incidence and prognostic implications of stable angina pectoris among women and men. *JAMA*. 2006;295(12):1404-1411.

4. Daly CA, De Stavola B, Sendon JLL, et al. Predicting prognosis in stable angina--results from the Euro heart survey of stable angina: prospective observational study. *BMJ*. 2006;332(7536):262-267.

5. Sekhri N, Feder GS, Junghans C, et al. Incremental prognostic value of the exercise electrocardiogram in the initial assessment of patients with suspected angina: cohort study. *BMJ*. 2008;337:a2240.

6. Clayton TC, Lubsen J, Pocock SJ, et al. Risk score for predicting death, myocardial infarction, and stroke in patients with stable angina, based on a large randomised trial cohort of patients. *BMJ*. 2005;331(7521):869.

7. Timmis AD, Feder GS, Sekhri N, Hemingway H. *Are rapid access chest pain clinics effective and fair? Characteristics and outcomes of patients from six centres*. National Co-ordinating Centre for NHS Service Delivery and Organisation R & D; 2005:228. Available at: http://www.sdo.nihr.ac.uk/files/project/32-final-report.pdf [Accessed February 10, 2011].

8. Abstract 800: Downstream Care and Outcomes Following Outpatient Stress Testing in the United States: How Low is Too Low Risk? 2009. Available at: http://circ.ahajournals.org/cgi/content/meeting_abstract/120/18_MeetingAbstracts/S393?maxtoshow=&hits=10&RESULTFORMAT=&fulltext=mudrick&searchid=1&FIRSTINDEX=0&volume=120&issue=18+Supplement&resourcetype=HWCIT [Accessed February 10, 2011].

9. Sekhri N, Feder GS, Junghans C, Hemingway H, Timmis AD. How effective are rapid access chest pain clinics? Prognosis of incident angina and non-cardiac chest pain in 8762 consecutive patients. *Heart*. 2007;93(4):458-463.

10. Daly CA, Clemens F, Sendon JLL, et al. The clinical characteristics and investigations planned in patients with stable angina presenting to cardiologists in Europe: from the Euro Heart Survey of Stable Angina. *Eur. Heart J*. 2005;26(10):996-1010.

11. Cheitlin MD, Armstrong WF, Aurigemma GP, et al. ACC/AHA/ASE 2003 guideline update for the clinical application of echocardiography--summary article: a report of the American College of Cardiology/American Heart Association Task Force on Practice Guidelines (ACC/AHA/ASE Committee to Update the 1997 Guidelines for the Clinical Application of Echocardiography). *J. Am. Coll. Cardiol*. 2003;42(5):954-970.

12. Gibbons RJ, Balady GJ, Bricker JT, et al. ACC/AHA 2002 guideline update for exercise testing: summary article. A report of the American College of Cardiology/American Heart Association Task Force on Practice Guidelines (Committee to Update the 1997 Exercise Testing Guidelines). *J. Am. Coll. Cardiol*. 2002;40(8):1531-1540.

13. Klocke FJ, Baird MG, Lorell BH, et al. ACC/AHA/ASNC guidelines for the clinical use of cardiac radionuclide imaging--executive summary: a report of the American College of Cardiology/American Heart Association Task Force on Practice Guidelines (ACC/AHA/ASNC Committee to Revise the 1995 Guidelines for the Clinical Use of Cardiac Radionuclide Imaging). *J. Am. Coll. Cardiol*. 2003;42(7):1318-1333.

14. Pope JH, Aufderheide TP, Ruthazer R, et al. Missed diagnoses of acute cardiac ischemia in the emergency department. *N. Engl. J. Med*. 2000;342(16):1163-1170.

15. Patel MR, Peterson ED, Dai D, et al. Low diagnostic yield of elective coronary angiography. *N. Engl. J. Med*. 2010;362(10):886-895.

16. Budoff MJ, Dowe D, Jollis JG, et al. Diagnostic performance of 64-multidetector row coronary computed tomographic angiography for evaluation of coronary artery stenosis in individuals without known coronary artery disease: results from the prospective multicenter ACCURACY (Assessment by Coronary Computed Tomographic Angiography of Individuals Undergoing Invasive Coronary Angiography) trial. *J. Am. Coll. Cardiol*. 2008;52(21):1724-1732.

17. Miller JM, Rochitte CE, Dewey M, et al. Diagnostic performance of coronary angiography by 64-row CT. *N. Engl. J. Med*. 2008;359(22):2324-2336.

18. Stein PD, Yaekoub AY, Matta F, Sostman HD. 64-slice CT for diagnosis of coronary artery disease: a systematic review. *Am. J. Med*. 2008;121(8):715-725.

19. Mowatt G, Cook JA, Hillis GS, et al. 64-Slice computed tomography angiography in the diagnosis and assessment of coronary artery disease: systematic review and meta-analysis. *Heart*. 2008;94(11):1386-1393.

20. LaRosa JC, He J, Vupputuri S. Effect of statins on risk of coronary disease: a meta-analysis of randomized controlled trials. *JAMA*. 1999;282(24):2340-2346.

21. Fleischmann KE, Hunink MG, Kuntz KM, Douglas PS. Exercise echocardiography or exercise SPECT imaging? A meta-analysis of diagnostic test performance. *JAMA*. 1998;280(10):913-920.

22. Schuijf JD, Poldermans D, Shaw LJ, et al. Diagnostic and prognostic value of non-invasive imaging in known or suspected coronary artery disease. *Eur. J. Nucl. Med. Mol. Imaging*. 2006;33(1):93-104.

23. Garber AM, Solomon NA. Cost-effectiveness of alternative test strategies for the diagnosis of coronary artery disease. *Ann. Intern. Med*. 1999;130(9):719-728.

24. CORONARY COMPUTED TOMOGRAPHIC ANGIOGRAPHY FOR DETECTION OF CORONARY ARTERY DISEASE. 2008. Available at: http://www.icer-review.org/index.php/CCTA/View-category.html [Accessed February 10, 2011].

25. Schuetz GM, Zacharopoulou NM, Schlattmann P, Dewey M. Meta-analysis: noninvasive coronary angiography using computed tomography versus magnetic resonance imaging. *Ann. Intern. Med*. 2010;152(3):167-177.

26. Marcassa C, Bax JJ, Bengel F, et al. Clinical value, cost-effectiveness, and safety of myocardial perfusion scintigraphy: a position statement. *Eur. Heart J*. 2008;29(4):557-563.

27. Mahenthiran J, Bangalore S, Yao S, Chaudhry FA. Comparison of prognostic value of stress echocardiography versus stress electrocardiography in patients with suspected coronary artery disease. *Am. J. Cardiol*. 2005;96(5):628-634.

28. Yao SS, Qureshi E, Sherrid MV, Chaudhry FA. Practical applications in stress echocardiography: risk stratification and prognosis in patients with known or suspected ischemic heart disease. *J. Am. Coll. Cardiol*. 2003;42(6):1084-1090.

29. Hachamovitch R, Hayes S, Friedman JD, et al. Determinants of risk and its temporal variation in patients with normal stress myocardial perfusion scans: what is the warranty period of a normal scan? *J. Am. Coll. Cardiol*. 2003;41(8):1329-1340.

30. Metz LD, Beattie M, Hom R, et al. The prognostic value of normal exercise myocardial perfusion imaging and exercise echocardiography: a meta-analysis. *J. Am. Coll. Cardiol*. 2007;49(2):227-237.

31. Bamberg F, Sommer W, Hoffmann V. Meta-analysis and systematic review of the long-term predictive value of assessment of coronary atherosclerosis by contrast-enhanced coronary computed tomography angiography. *Journal of the American College of Cardiology*. In press.

32. Min JK, Shaw LJ, Devereux RB, et al. Prognostic value of multidetector coronary computed tomographic angiography for prediction of all-cause mortality. *J. Am. Coll. Cardiol*. 2007;50(12):1161-1170.

33. Ostrom MP, Gopal A, Ahmadi N, et al. Mortality incidence and the severity of coronary atherosclerosis assessed by computed tomography angiography. *J. Am. Coll. Cardiol*. 2008;52(16):1335-1343.

34. Hachamovitch R, Berman DS, Kiat H, et al. Exercise myocardial perfusion SPECT in patients without known coronary artery disease: incremental prognostic value and use in risk stratification. *Circulation*. 1996;93(5):905-914.

35. Berman DS, Kang X, Hayes SW, et al. Adenosine myocardial perfusion single-photon emission computed tomography in women compared with men. Impact of diabetes mellitus on incremental prognostic value and effect on patient management. *J. Am. Coll. Cardiol*. 2003;41(7):1125-1133.

36. Galassi AR, Azzarelli S, Tomaselli A, et al. Incremental prognostic value of technetium-99m-tetrofosmin exercise myocardial perfusion imaging for predicting outcomes in patients with suspected or known coronary artery disease. *Am. J. Cardiol*. 2001;88(2):101-106.

37. Hachamovitch R, Berman DS, Shaw LJ, et al. Incremental prognostic value of myocardial perfusion single photon emission computed tomography for the prediction of cardiac death: differential stratification for risk of cardiac death and myocardial infarction. *Circulation*. 1998;97(6):535-543.

38. Picano E, Severi S, Michelassi C, et al. Prognostic importance of dipyridamole-echocardiography test in coronary artery disease. *Circulation*. 1989;80(3):450-457.

39. Poldermans D, Fioretti PM, Boersma E, et al. Dobutamine-atropine stress echocardiography and clinical data for predicting late cardiac events in patients with suspected coronary artery disease. *Am. J. Med*. 1994;97(2):119-125.

40. Cortigiani L, Dodi C, Paolini EA, et al. Prognostic value of pharmacological stress echocardiography in women with chest pain and unknown coronary artery disease. *J. Am. Coll. Cardiol*. 1998;32(7):1975-1981.

41. Weiner DA, Ryan TJ, McCabe CH, et al. Prognostic importance of a clinical profile and exercise test in medically treated patients with coronary artery disease. *J. Am. Coll. Cardiol*. 1984;3(3):772-779.

42. Mark DB, Hlatky MA, Harrell FE, et al. Exercise treadmill score for predicting prognosis in coronary artery disease. *Ann. Intern. Med*. 1987;106(6):793-800.

43. Mark DB, Shaw L, Harrell FE, et al. Prognostic value of a treadmill exercise score in outpatients with suspected coronary artery disease. *N. Engl. J. Med*. 1991;325(12):849-853.

44. Min JK, Shaw LJ, Berman DS, Gilmore A, Kang N. Costs and clinical outcomes in individuals without known coronary artery disease undergoing coronary computed tomographic angiography from an analysis of Medicare category III transaction codes. *Am. J. Cardiol*. 2008;102(6):672-678.

45. Hoffmann U, Moselewski F, Nieman K, et al. Noninvasive assessment of plaque morphology and composition in culprit and stable lesions in acute coronary syndrome and stable lesions in stable angina by multidetector computed tomography. *J. Am. Coll. Cardiol*. 2006;47(8):1655-1662.

46. Min JK, Kang N, Shaw LJ, et al. Costs and clinical outcomes after coronary multidetector CT angiography in patients without known coronary artery disease: comparison to myocardial perfusion SPECT. *Radiology*. 2008;249(1):62-70.

47. Abstract 981: High-Sensitivity Troponin Reliably Excludes Acute Coronary Syndrome in Patients with Acute Chest Pain: Results from the Rule Out Myocardial Infarction by Computed Tomography (ROMICAT) Study. 2008. Available at: http://circ.ahajournals.org/cgi/content/meeting_abstract/118/18_MeetingAbstracts/S_637 [Accessed February 10, 2011].

48. Shah BR, Patel MR, Peterson ED, Douglas PS. Defining optimal research study design for cardiovascular imaging using computed tomography angiography as a model. *Am. J. Cardiol*. 2008;102(7):943-948.

49. Hachamovitch R, Di Carli MF. Methods and limitations of assessing new noninvasive tests: part I: Anatomy-based validation of noninvasive testing. *Circulation*. 2008;117(20):2684-2690.

50. Bonow RO. Sixth Annual Mario S. Verani, MD Memorial Lecture: Cardiovascular imaging--added value or added cost? *J Nucl Cardiol*. 2008;15(2):170-177.

51. Douglas PS. Improving imaging: our professional imperative. *J. Am. Coll. Cardiol*. 2006;48(10):2152-2155.

52. Douglas PS, Taylor A, Bild D, et al. Outcomes research in cardiovascular imaging: report of a workshop sponsored by the National Heart, Lung, and Blood Institute. *Circ Cardiovasc Imaging*. 2009;2(4):339-348.

53. Douglas PS, Khandheria B, Stainback RF, et al. ACCF/ASE/ACEP/AHA/ASNC/SCAI/SCCT/SCMR 2008 appropriateness criteria for stress echocardiography: a report of the American College of Cardiology Foundation Appropriateness Criteria Task Force, American Society of Echocardiography, American College of Emergency Physicians, American Heart Association, American Society of Nuclear Cardiology, Society for Cardiovascular Angiography and Interventions, Society of Cardiovascular Computed Tomography, and Society for Cardiovascular Magnetic Resonance endorsed by the Heart Rhythm Society and the Society of Critical Care Medicine. *J. Am. Coll. Cardiol*. 2008;51(11):1127-1147.

54. Brindis RG, Douglas PS, Hendel RC, et al. ACCF/ASNC appropriateness criteria for single-photon emission computed tomography myocardial perfusion imaging (SPECT MPI): a report of the American College of Cardiology Foundation Quality Strategic Directions Committee Appropriateness Criteria Working Group and the American Society of Nuclear Cardiology endorsed by the American Heart Association. *J. Am. Coll. Cardiol*. 2005;46(8):1587-1605.

55. Fryback DG, Thornbury JR. The efficacy of diagnostic imaging. *Med Decis Making*. 1991;11(2):88-94.

56. Detrano R, Guerci AD, Carr JJ, et al. Coronary calcium as a predictor of coronary events in four racial or ethnic groups. *N. Engl. J. Med*. 2008;358(13):1336-1345.

57. Bairey Merz CN, Shaw LJ, Reis SE, et al. Insights from the NHLBI-Sponsored Women's Ischemia Syndrome Evaluation (WISE) Study: Part II: gender differences in presentation, diagnosis, and outcome with regard to gender-based pathophysiology of atherosclerosis and macrovascular and microvascular coronary disease. *J. Am. Coll. Cardiol*. 2006;47(3 Suppl):S21-29.

58. Falk E, Shah PK, Fuster V. Coronary plaque disruption. *Circulation*. 1995;92(3):657-671.

59. Kern MJ, Meier B. Evaluation of the culprit plaque and the physiological significance of coronary atherosclerotic narrowings. *Circulation*. 2001;103(25):3142-3149.

60. Javitz HS, Ward MM, Watson JB, Jaana M. Cost of illness of chronic angina. *Am J Manag Care*. 2004;10(11 Suppl):S358-369.

61. Douglas P, Iskandrian AE, Krumholz HM, et al. Achieving quality in cardiovascular imaging: proceedings from the American College of Cardiology-Duke University Medical Center Think Tank on Quality in Cardiovascular Imaging. *J. Am. Coll. Cardiol*. 2006;48(10):2141-2151.

62. Douglas PS, Chen J, Gillam L, et al. Achieving Quality in Cardiovascular Imaging II: Proceedings From the Second American College of Cardiology-Duke University Medical Center Think Tank on Quality in Cardiovascular Imaging. *J Am Coll Cardiol Img*. 2009;2(2):231-240.

63. Lauer MS. CT angiography: first things first. *Circ Cardiovasc Imaging*. 2009;2(1):1-3.

64. Tunis SR, Stryer DB, Clancy CM. Practical clinical trials: increasing the value of clinical research for decision making in clinical and health policy. *JAMA*. 2003;290(12):1624-1632.

65. Lucas FL, DeLorenzo MA, Siewers AE, Wennberg DE. Temporal trends in the utilization of diagnostic testing and treatments for cardiovascular disease in the United States, 1993-2001. *Circulation*. 2006;113(3):374-379.

66. *Medicare Part B imaging services. Rapid spending growth and shift to physician offices indicate need for CMS to consider additional management practices*. Washington, DC: Government Accountability Office; 2008:55. Available at: http://www.gao.gov/new.items/d08452.pdf [Accessed February 10, 2011].

67. Hendel RC. Utilization Management of Cardiovascular Imaging: Pre-Certification and Appropriateness. *J Am Coll Cardiol Img*. 2008;1(2):241-248.

68. Sharples L, Hughes V, Crean A, et al. Cost-effectiveness of functional cardiac testing in the diagnosis and management of coronary artery disease: a randomised controlled trial. The CECaT trial. *Health Technol Assess*. 2007;11(49):iii-iv, ix-115.

69. Ladapo JA, Hoffmann U, Bamberg F, et al. Cost-effectiveness of coronary MDCT in the triage of patients with acute chest pain. *AJR Am J Roentgenol*. 2008;191(2):455-463.

70. Chow BJW, Abraham A, Wells GA, et al. Diagnostic accuracy and impact of computed tomographic coronary angiography on utilization of invasive coronary angiography. *Circ Cardiovasc Imaging*. 2009;2(1):16-23.

71. Hunink MGM, Krestin GP. Study design for concurrent development, assessment, and implementation of new diagnostic imaging technology. *Radiology*. 2002;222(3):604-614.

72. Meijboom WB, van Mieghem CAG, Mollet NR, et al. 64-slice computed tomography coronary angiography in patients with high, intermediate, or low pretest probability of significant coronary artery disease. *J. Am. Coll. Cardiol*. 2007;50(15):1469-1475.

73. Taylor AJ. Finding the pathway to improving patient outcomes with atherosclerosis imaging: who's motivated? *Am. Heart J*. 2007;154(6):1008-1010.

74. Pellikka PA, Nagueh SF, Elhendy AA, Kuehl CA, Sawada SG. American Society of Echocardiography recommendations for performance, interpretation, and application of stress echocardiography. *J Am Soc Echocardiogr*. 2007;20(9):1021-1041.

75. Abbara S, Arbab-Zadeh A, Callister TQ, et al. SCCT guidelines for performance of coronary computed tomographic angiography: a report of the Society of Cardiovascular Computed Tomography Guidelines Committee. *J Cardiovasc Comput Tomogr*. 2009;3(3):190-204.

76. Raff GL, Abidov A, Achenbach S, et al. SCCT guidelines for the interpretation and reporting of coronary computed tomographic angiography. *J Cardiovasc Comput Tomogr*. 2009;3(2):122-136.

77. Imaging Guidelines for Nuclear Cardiology Procedures: SPECT and Instrumentation. *Journal of Nuclear Cardiology*. 2007;14(6):913-913.

78. Beller GA, Bonow RO, Fuster V. ACCF 2008 Recommendations for Training in Adult Cardiovascular Medicine Core Cardiology Training (COCATS 3) (revision of the 2002 COCATS Training Statement). *J. Am. Coll. Cardiol*. 2008;51(3):335-338.

79. Ficaro EP, Zanzonico P, Stabin MG, et al. Variability in radiation dose estimates from nuclear and computed tomography diagnostic imaging. 2008. Available at: http://www.asnc.org/imageuploads/InformationStatementRadiationDosimetry2009.pdf [Accessed February 11, 2011].

80. Bedetti G, Botto N, Andreassi MG, et al. Cumulative patient effective dose in cardiology. *Br J Radiol*. 2008;81(969):699-705.

81. Einstein AJ, Henzlova MJ, Rajagopalan S. Estimating risk of cancer associated with radiation exposure from 64-slice computed tomography coronary angiography. *JAMA*. 2007;298(3):317-323.

82. Kalbfleisch J. *The statistical analysis of failure time data*. 2nd ed. Hoboken N.J.: J. Wiley; 2002.

83. Cox DR. Regression Models and Life-Tables. *Journal of the Royal Statistical Society. Series B (Methodological)*. 1972;34(2):187-220.

84. Schoenfeld DA. Sample-size formula for the proportional-hazards regression model. *Biometrics*. 1983;39(2):499-503.

85. Borenstein M. The case for confidence intervals in controlled clinical trials. *Control Clin Trials*. 1994;15(5):411-428.

86. Kaplan E, Meier P. Nonparametric estimation from incomplete observations. *Journal of the American Statistical Association*. 1958:457-481.

87. Begg CB, Greenes RA. Assessment of diagnostic tests when disease verification is subject to selection bias. *Biometrics*. 1983;39(1):207-215.

88. Kosinski AS, Barnhart HX. A global sensitivity analysis of performance of a medical diagnostic test when verification bias is present. *Stat Med*. 2003;22(17):2711-2721.

89. Alonzo TA, Pepe MS. Assessing accuracy of a continuous screening test in the presence of verification bias. *J Royal Statistical Soc C*. 2005;54(1):173-190.

90. Hedeker D, Gibbons RD. A random-effects ordinal regression model for multilevel analysis. *Biometrics*. 1994;50(4):933-944.

91. Bang H, Tsiatis AA. Median regression with censored cost data. *Biometrics*. 2002;58(3):643-649.

92. O'Brien PC, Fleming TR. A multiple testing procedure for clinical trials. *Biometrics*. 1979;35(3):549-556.

93. GORDON LAN KK, DEMETS DL. Discrete sequential boundaries for clinical trials. *Biometrika*. 1983;70(3):659 -663.

The **PROMISE** Trial FINAL Protocol

<u>PRO</u>spective **<u>M</u>**ulticenter **<u>I</u>**maging **<u>S</u>**tudy for **<u>E</u>**valuation of Chest Pain

**Sponsor:**	National Heart Lung and Blood Institute (NHLBI)
**<u>Clinical Coordinating Center</u>**	
**Principal Investigator:**	Pamela S. Douglas, MD
**Co-Investigators:**	Rowena Dolor, MD<br>Daniel Mudrick, MD<br>Manesh R. Patel, MD<br>Eric J. Velazquez, MD
**<u>Statistical/Data Coordinating Center</u>**	
**Principal Investigator:**	Kerry L. Lee, PhD
**Co-Investigators:**	Andrzej S. Kosinski, PhD<br>Hussein R. Al-Khalidi, PhD
**<u>Diagnostic Testing Coordinating Center</u>**	
**Principal Investigator:**	Udo Hoffmann, MD, MPH
**Co-Investigators:**	Mitchell Krucoff, MD<br>Michael Picard, MD<br>James Udelson, MD
**<u>Economics/Quality of Life</u>**	
**Principal Investigator:**	Daniel B. Mark, MD, MPH
**Co-Investigator:**	Kevin Anstrom, PhD
**Investigation of:**	Diagnostic testing strategies for suspected coronary artery disease (CAD), including use of coronary computed tomographic angiography (CTA)
**FINAL Date:**	**April 12, 2010**
**Amended:**	**February 22, 2011**<br>**April 30, 2013**

CONFIDENTIAL

This document is confidential and the property of Duke Clinical Research Institute (DCRI). No part of it may be transmitted, reproduced, published, or used by other persons without prior written authorization from the DCRI. This document may be disclosed to the appropriate institutional ethics committees/institutional review boards or to duly authorized representatives of the U.S. Food and Drug Administration or other regulatory authority under the condition that they maintain confidentiality.

Investigator Protocol Signature Page

I have read and understand the protocol and agree that it contains all the ethical, legal, and scientific information necessary to conduct this study. I will personally conduct the study as described. I will provide copies of the protocol to all physicians, nurses, and other professional personnel responsible to me who will participate in the study. I am aware that this protocol must be approved by the Institutional Review Board or Ethics Committee. I agree to adhere strictly to the attached protocol. I agree that clinical data entered on case report forms by me and my staff will be supplied to the Duke Clinical Research Institute (DCRI) and may be utilized by the DCRI in various ways, such as for submission to governmental regulatory authorities and/or in combination with clinical data gathered from other research sites, whenever applicable. I agree to allow DCRI monitors and auditors full access to all medical records at the research facility for subjects screened or randomized in the study. I agree to provide all subjects with informed consent forms, as required by government regulations and International Conference on Harmonisation guidelines.

Version Date: Amendment 2

____________________________________ ____________________________
Principal Investigator (print name) **Site Name and Number**

____________________________________ __________________________
Principal Investigator (signature) **Date**

Protocol Version and Amendment Tracking

Version Number/Amendment	Approval Date
1.0	April 12, 2010
Amendment 1	February 22, 2011
Amendment 2	April 30, 2013

TABLE OF CONTENTS

A INTRODUCTION ... 19

A1 STUDY SYNOPSIS ... 19
A2 PRIMARY HYPOTHESIS ... 20
A3 SIGNIFICANCE OF THE STUDY ... 21

B BACKGROUND ... 22

B1 PRIOR LITERATURE AND STUDIES ... 22
1.a Current Management of Patients with Chest Pain: Extent of the Problem ... 22
1.b Current Diagnostic Strategies Using Functional Tests are Inadequate ... 22
1.c Computed Tomographic Angiography is Highly Accurate in Detecting and Excluding Obstructive and Nonobstructive Coronary Artery Disease ... 23
1.d Additional Important Considerations in Comparing Test Characteristics ... 24
B2 RATIONALE: THE URGENT NEED FOR A RANDOMIZED TRIAL OF DIAGNOSTIC STRATEGIES IN SUBJECTS WITH SUSPECTED CORONARY ARTERY DISEASE ... 25

C STUDY OVERVIEW AND OBJECTIVES ... 26

C1 OVERVIEW OF THE PROMISE TRIAL ... 26
C2 PRIMARY AIM ... 27
C3 SECONDARY AIMS ... 27
C4 RATIONALE FOR THE SELECTION OF OUTCOME MEASURES ... 28
C5 RATIONALE FOR SELECTION OF TESTING IN EACH EXPERIMENTAL ARM ... 28

D INVESTIGATIONAL PLAN ... 30

D1 OVERVIEW OF TRIAL DESIGN ... 30
D2 SUBJECT POPULATION ... 30
2.a Inclusion Criteria ... 31
2.b Exclusion Criteria ... 31
2.c Ethical Considerations ... 32
2.d Subject Recruitment Plans and Consent Process ... 32
2.e Subject Randomization ... 33
2.f Risks and Benefits ... 33
D3 DIAGNOSTIC TESTING ... 34
3.a Approach to Diagnostic Testing ... 34
3.b Description of Testing To Be Performed ... 35
3.c Functional and Anatomic Testing ... 35
3.d Test Transfer and Storage ... 36
3.e Quality Assurance of Diagnostic Testing ... 36
3.f Subsequent Medical Care ... 37
3.g Testing Risk and Benefits ... 37

E STUDY PROCEDURES ... 38

E1 SCREENING FOR ELIGIBILITY ... 38
E2 SCHEDULE OF ASSESSMENTS ... 38
E3 SAFETY ... 41
3.a Data and Safety Monitoring Board ... 41
3.b Study Coordinating Center or Food and Drug Administration Notification by Investigator ... 41
3.c Definitions of Safety Events and Reporting ... 41
E4 STUDY OUTCOME MEASUREMENTS AND ASCERTAINMENT ... 42
E5 INDEPENDENT CLINICAL EVENT ADJUDICATION COMMITTEE ... 44
E6 QUALITY-OF-LIFE ASSESSMENTS ... 45

E7 ECONOMIC ASSESSMENTS 46
E8 PROMISE BIOREPOSITORIES 46
8.a Imaging and Electrocardiograms 46
8.b Blood Biomarkers and Genomics 46

F STATISTICAL PLAN 46

F1 SAMPLE SIZE DETERMINATION AND STATISTICAL POWER 46
F2 STATISTICAL ANALYSIS PLAN 48
2.a Analysis for the Primary Endpoint 48
2.b Analysis of Secondary Endpoints 49
2.c Analysis of Diagnostic Testing Core Data 50
2.d Analysis of Diagnostic Accuracy 51
2.e Analysis of Safety Events, Morbidity, Radiation Dose, and Incidental Findings 52
2.f Assessment of Prognostic Factors 52
2.g Quality-of-life Analyses 52
2.h Economic Analyses 53
F3 INTERIM ANALYSES 53

G DATA HANDLING AND RECORD KEEPING 54

G1 CONFIDENTIALITY AND SECURITY 55
G2 TRAINING 55
G3 ELECTRONIC CASE REPORT FORM 55
G4 RECORDS RETENTION 55

H STUDY MONITORING, AUDITING, AND INSPECTING 56

H1 STUDY MONITORING PLAN 56
H2 AUDITING AND INSPECTING 56

I STUDY ADMINISTRATION 57

I1 ORGANIZATION AND PARTICIPATING CENTERS 57
I2 FUNDING SOURCE 57
I3 STUDY TIMETABLE 57

J REFERENCES 59

Table of Revisions to NHLBI PROMISE Protocol, Version 2.0

Section	Original	Revision	Rationale
Throughout the document	Made grammatical and spelling corrections		Correctness
Title page; Investigator Protocol Signature Page; Protocol Version and Amendment Tracking page	version number: 1.0<br>date: February 22, 2011	version number: Amendment 2<br>date: April 30, 2013	Self-evident

Section	Original	Revision	Rationale
A1	**Secondary Endpoints**<br>• Death or MI or unstable angina hospitalization<br>• Death or MI<br>• Major complications from cardiovascular procedures and testing (stroke, major bleeding, anaphylaxis, and renal failure)<br>• Medical costs, resource use, and incremental cost effectiveness<br>• Health-related quality of life	**Secondary Endpoints**<br>• Death or MI or unstable angina hospitalization<br>• Death or MI<br>• Major complications from cardiovascular procedures and testing (stroke, major bleeding, anaphylaxis, and renal failure requiring dialysis)<br>• Composite endpoint of the primary endpoint or invasive catheterization without obstructive CAD<br>• Invasive catheterization without obstructive CAD (Defined as no stenosis >=50% in any major epicardial vessel including side branches ≥ 2 mm in diameter, on the first cath performed <= 60 days after randomization))<br>• Medical costs, resource use, and incremental cost effectiveness<br>• Health-related quality of life (QOL)	Addition of secondary endpoint
A1	The trial will have an approximate 42-month enrollment period and 2-year minimum follow-up period. All subjects will be followed from enrollment until either death or the end of the follow-up period.	The trial will have an approximate 36-month enrollment period and 1-year minimum follow-up period. All subjects will be followed from enrollment until either death or the end of the follow-up period.	Clarify study timeline and patient follow-up period.

Section	Original	Revision	Rationale
A1	**End-of-Study Definition**<br><br>2-year minimum follow-up of all patients, with expected 30-month average follow-up for clinical status assessment.	**End-of-Study Definition**<br><br>1-year minimum follow-up of all patients, with expected 24-month average follow-up for clinical status assessment.	Clarify study timeline and patient follow-up period.
A1	**Number of Subjects**<br><br>Approximately 10,000 subjects will be enrolled, which is expected to provide 90% power to detect a 20% reduction in the primary composite event rate in the anatomic testing arm as compared with functional testing.	**Number of Subjects**<br><br>Approximately 10,000 subjects will be enrolled.	Clarification
A1	**Number of Sites**<br><br>**~200-250**	**Number of Sites**<br><br>**~220**	Clarification

Section	Original	Revision	Rationale
1d	Therefore, in response to these needs, the PROMISE trial, a multicenter, randomized, pragmatic trial, is comparing 2 state-of-the-art diagnostic strategies in symptomatic subjects at clinically determined, low-intermediate risk for CAD who require nonurgent testing. The “anatomic” testing strategy uses coronary CTA (greater than or equal to 64-slice) as the initial test. The “functional” stress testing strategy uses physician-selected stress imaging (ECHO or nuclear) or exercise ECG as the initial test. Ten thousand subjects will be randomized over approximately 42 months and followed for a minimum of 2 years (average 2.5 years). All subsequent diagnostic and therapeutic management decisions will be based on the latest clinical practice guideline recommendations and will be at the discretion of the treating care team.	Therefore, in response to these needs, the PROMISE trial, a multicenter, randomized, pragmatic trial, is comparing 2 state-of-the-art diagnostic strategies in symptomatic subjects at clinically determined, low-intermediate risk for CAD who require nonurgent testing. The “anatomic” testing strategy uses coronary CTA (greater than or equal to 64-slice) as the initial test. The “functional” stress testing strategy uses physician-selected stress imaging (echo or nuclear) or exercise ECG as the initial test. Ten thousand subjects will be randomized over approximately 36 months and followed for a minimum of 1 year (average 2 years). All subsequent diagnostic and therapeutic management decisions will be based on the latest clinical practice guideline recommendations and will be at the discretion of the treating care team.	Clarify study timeline and patient follow-up period.
C1	Subjects will be randomized over approximately 42 months and followed for 24 to 48 months (or until the study ends) at 200 to 250 North American primary-care, cardiology, and acute-care practice sites, reflecting the physician specialties and community settings where the vast majority of chest-pain patients receive care.	Subjects will be randomized over approximately 36 months and followed for 12 to 36 months (or until the study ends) at 200 to 250 North American primary-care, cardiology, and acute-care practice sites, reflecting the physician specialties and community settings where the vast majority of chest-pain patients receive care.	Clarify study timeline and patient follow-up period.
C3	The secondary aims are to compare the following clinical and economic outcomes in subjects randomized to initial	The secondary aims are to compare the following clinical and economic outcomes in subjects randomized to initial	Addition of secondary endpoint

Section	Original	Revision	Rationale
	anatomic versus functional diagnostic testing:<br>• Death or MI or unstable angina hospitalization<br>• Death or MI<br>• Major complications from cardiovascular procedures and testing (stroke, major bleeding, anaphylaxis, and renal failure requiring dialysis)<br>• Medical costs, resource use, and incremental cost effectiveness<br>• Health-related quality of life (QOL)<br>In addition, cumulative radiation exposure will be assessed as a secondary safety endpoint.	anatomic versus functional diagnostic testing:<br>• Death or MI or unstable angina hospitalization<br>• Death or MI<br>• Major complications from cardiovascular procedures and testing (stroke, major bleeding, anaphylaxis, and renal failure requiring dialysis)<br>• Composite endpoint of the primary endpoint or invasive catheterization without obstructive CAD<br>• Invasive catheterization without obstructive CAD (Defined as no stenosis >=_50% in any major epicardial vessel including side branches ≥ 2 mm in diameter, on the first cath performed <= 60 days after randomization))<br>• Medical costs, resource use, and incremental cost effectiveness<br>• Health-related quality of life (QOL)	

Section	Original	Revision	Rationale
C4	The use of a composite clinical endpoint and a subject follow-up of at least 2 years are essential to testing PROMISE's hypothesis of clinical superiority of CTA.	The use of a composite clinical endpoint and a subject follow-up of at least 1 year are essential to testing PROMISE's hypothesis of clinical superiority of CTA.	Change in study timeline and patient follow-up period
D1		Figure 1 changed to incorporate changes to secondary endpoint.	Addition of secondary endpoint
E2		Table changed to reflect follow-up of no more than 36 months.	Change in study timeline and patient follow-up period.
E2	• QOL questionnaires will be completed at 6 months, and annually for a minimum of 12 and maximum of 24 months post-randomization (for patients enrolled in QOL).	• QOL questionnaires will be completed at 6 months, 12 months, and 24 months post-randomization (among patients followed for 24 months).	Change in study timeline and patient follow-up period
E4	***Myocardial Infarction***<br>Defined as either 1) an abnormal cardiac biomarker level (either troponin or CK-MB) greater than institutional upper limit of normal (ULN), and either ischemic discomfort lasting greater than 10 minutes or ECG changes indicative of ischemia or infarction, or 2) new abnormal Q waves consistent with infarction. Additionally *peri-procedural infarctions* are defined as greater than 3x ULN for serum CK-MB for PCI and greater than 5x ULN for CABG.	***Myocardial Infarction***<br>Defined as either 1) an abnormal cardiac biomarker level (either troponin or CK-MB) greater than institutional upper limit of normal (ULN), and either ischemic discomfort lasting greater than 10 minutes or ECG changes indicative of ischemia or infarction, or 2) new abnormal Q waves consistent with infarction. Additionally *peri-procedural infarctions* are defined as greater than 3x ULN for serum CK-MB for PCI and greater	Clarification

Section	Original	Revision	Rationale
		than 5x ULN for CABG.<br>• an abnormal cardiac biomarker level > institutional ULN (either troponin or CK-MB), and either ischemic discomfort lasting > 10 minutes or ECG changes indicative of ischemia or infarction,<br>*OR*<br>• new abnormal Q waves consistent with infarction.<br><br>Additionally *peri-procedural infarctions* are defined as >3x upper limit of normal for serum CK-MB for PCI and >5x upper limit of normal for CABG.	
E4	***Unstable Angina Hospitalization***<br>Defined as 1) ischemic discomfort or equivalent symptoms requiring hospitalization within 48 hours of symptoms, 2) lasting greater than or equal to 10 minutes at rest, or in an accelerating pattern, 3) accompanied by dynamic ST depression, ischemia on stress testing, or significant epicardial coronary artery stenosis, and 4) considered to be myocardial ischemia upon final diagnosis.	***Unstable Angina Hospitalization***<br>Defined as 1) ischemic discomfort or equivalent symptoms requiring hospitalization within 48 hours of symptoms, 2) lasting greater than or equal to 10 minutes at rest, or in an accelerating pattern, 3) accompanied by dynamic ST depression, ischemia on stress testing, or significant epicardial coronary artery stenosis, and 4) considered to be myocardial ischemia upon final diagnosis.<br>Is defined as an event in which the final diagnosis is unstable angina or acute coronary syndrome	Clarification

Section	Original	Revision	Rationale
		due to myocardial ischemia and has the following criteria:<br>• ischemic discomfort or equivalent symptoms requiring hospitalization within 48 hours of symptoms, lasting ≥ 10 minutes at rest<br>• ***OR***<br>• ischemic discomfort or equivalent symptoms occurring in an accelerated pattern within 48 hours of hospitalization<br>***AND ANY OF THE FOLLOWING***<br>• accompanied by dynamic ST depression<br>• ischemia on stress testing<br>• significant epicardial coronary artery stenosis	
E4		***Addition***<br>***Invasive catheterization without obstructive coronary artery disease***<br>Only the results of the initial (first) invasive cardiac catheterization that occurs within the first <u>60 days</u> following randomization will be considered for the secondary endpoints which include invasive angiography without obstructive disease. The absence of obstructive disease is defined as no stenosis >= 50% in any	Addition of secondary endpoint

Section	Original	Revision	Rationale
		major epicardial vessel including side branches ≥ 2 mm in diameter.	
E5	An independent clinical event adjudication committee will review and adjudicate all primary endpoint events in a blinded fashion based on the definitions presented above. The clinical secondary endpoint events and cumulative radiation exposure will not be adjudicated.	An independent clinical event adjudication committee will review and adjudicate all primary endpoint events and the catheterization secondary endpoints in a blinded fashion based on the definitions presented above. If the invasive cardiac catheterization report is inconclusive, the CEC will review the catheterization films for a visual assessment of CAD. The other clinical secondary endpoint events and cumulative radiation exposure will not be adjudicated.	Addition of secondary endpoint
E6	Baseline QOL interviews will be administered to subjects by site personnel as soon as possible after consent, preferably before the subject's randomization. Completed questionnaires will be sent directly to the EQOL CC for data processing. Follow-up QOL questionnaires will be administered to subjects via structured telephone interview by DOFG trained and supervised interviewers at 6 and 12 months postrandomization and annually thereafter until the end of the study. Proxy QOL questionnaires will be used when a subject has died in the follow-up interval	Baseline QOL interviews will be administered to ~6000 subjects enrolled (prior to July 2012) by site personnel as soon as possible after consent, preferably before the subject's randomization. Completed questionnaires will be sent directly to the EQOL CC for data processing. Follow-up QOL questionnaires will be administered to via structured telephone interview by DOFG trained and supervised interviewers at at 6 months, 12 months, and 24 months post-	Change in study timeline.

Section	Original	Revision	Rationale
	or has become incapacitated; these questionnaires will include items that can be reliably obtained from a relative, caretaker, or medical record. Proxy questionnaires will be used if the subject is unable to participate in follow-ups via telephone interview.	randomization (among patients followed for 24 months). Proxy QOL questionnaires will be used when a subject has died in the follow-up interval or has become incapacitated; these questionnaires will include items that can be reliably obtained from a relative, caretaker, or medical record. Proxy questionnaires will be used if the subject is unable to participate in follow-ups via telephone interview.	
F2, 2c	Two important components of information from the diagnostic test QA activity will be analyzed by the SDCC. The DTCC will review every subjects' initial diagnostic test for technical quality	Two important components of information from the diagnostic test QA activity will be analyzed by the SDCC. The DTCC will review the first 8000 subjects' initial diagnostic test for technical quality.	Change in study timeline
F2, 2c	The second important component of information from the diagnostic test QA activity is the quality of test interpretations. Approximately 20% of tests will be overread by the DTCC using a categorical level of coronary disease risk.	The second important component of information from the diagnostic test QA activity is the quality of test interpretations. Approximately 10% of tests will be overread by the DTCC using a categorical level of coronary disease risk.	Change in study timeline
F3		References corrected	Correction
I3		Study Timeline figure adjusted	Change in study timeline and patient follow-up period.
J	93. GORDON LAN KK, DEMETS DL. Discrete	93. Lan GKK, DeMets DL. Discrete sequential	Correction

Section	Original	Revision	Rationale
	sequential boundaries for clinical trials. *Biometrika*. 1983;70(3):659 -663.	boundaries for clinical trials. *Biometrika*. 1983;70(3):659 -663.	

TABLE OF ABBREVIATIONS

ABI	ankle brachial index
ACC	American College of Cardiology
ACRIN	American College of Radiology Imaging Network
ACS	acute coronary syndrome
AHA	American Heart Association
BMI	body mass index
BP	blood pressure
CABG	coronary artery bypass graft
CAD	coronary artery disease
CCC	Clinical Coordinating Center
CD	compact disc
CI	confidence interval
CK-MB	creatine kinase-myocardial band
COCATS	Core Cardiology Training Symposium
CTA	coronary tomographic angiography
DASI	Duke Activity Status Index
DCRI	Duke Clinical Research Institute
DOFG	Duke Clinical Research Institute Outcomes and Follow-up Group
DSMB	Data and Safety Monitoring Board
DTCC	Diagnostic Testing Coordinating Center
ECG	electrocardiogram
ECHO	echocardiogram
eCRF	electronic case report form
EQOL CC	Economics and Quality of Life Coordinating Center
g/L	grams per liter
HDL	high-density lipoprotein
IEC	institutional ethics committee
IRB	institutional review board
IVRS	interactive voice response system
LDL	low-density lipoprotein
LV	left ventricular
MAR	missing at random
MI	myocardial infarction

MOP	manual of procedures
mSv	millisievert
NCDR	National Cardiovascular Data Registry
NHLBI	National Heart Lung and Blood Institute (U.S.)
PAD	peripheral arterial disease
PCI	percutaneous coronary intervention
PI	principal investigator
PROMISE	PROspective Multicenter Imaging Study for Evaluation of Chest Pain
QA	quality assurance
QOL	quality of life
ROC	receiver operating characteristic
SAQ	Seattle Angina Questionnaire
SDCC	Statistical and Data Coordinating Center
SF-12	Medical Outcomes Study Short Form
ULN	upper limit of normal

A Introduction

A1 *Study Synopsis*

**Sponsor**	National Heart Lung and Blood Institute (NHLBI)
**Protocol Title**	The PROMISE Trial - **PRO**spective **M**ulticenter **I**maging **S**tudy for **E**valuation of Chest Pain
**Diagnosis and Main Criterion for Inclusion**	Symptomatic subjects without known coronary artery disease (CAD) for whom a nonemergent, noninvasive cardiovascular diagnostic test for CAD is planned.
**Primary Study Objective**	To determine whether an initial noninvasive anatomic imaging strategy with coronary computed tomographic angiography (CTA) will improve clinical outcomes in subjects with symptoms concerning for CAD relative to an initial functional testing strategy.
**Primary Endpoint**	Time to first event using the composite of the following major cardiovascular events:<br>• Death<br>• Myocardial infarction (MI)<br>• Major complications from cardiovascular procedures including testing (stroke, major bleeding, anaphylaxis and renal failure)<br>• Unstable angina hospitalization
**Secondary Endpoints**	• Death or MI or unstable angina hospitalization<br>• Death or MI<br>• Major complications from cardiovascular procedures and testing (stroke, major bleeding, anaphylaxis, and renal failure requiring dialysis)<br>• Composite endpoint of the primary endpoint or invasive catheterization without obstructive CAD<br>• Invasive catheterization without obstructive CAD (Defined as no stenosis >= 50% in any major epicardial vessel including side branches ≥ 2 mm in diameter, on the first cath performed <= 60 days after randomization))<br>• Medical costs, resource use, and incremental cost effectiveness<br>• Health-related quality of life (QOL)

**Secondary Safety Endpoint**	Cumulative radiation exposure
**Primary Hypothesis**	An initial anatomic testing strategy will provide information that will result in superior long-term health outcomes as compared with an initial functional testing strategy.
**Study Design**	Pragmatic randomized trial of clinical effectiveness of diagnostic testing strategies for CAD, to be performed in outpatient settings, including urgent care, primary care, and cardiology offices. Qualifying patients presenting with new or worsening symptoms suspicious for clinically significant CAD who require diagnostic testing and have not been previously evaluated for this episode of symptoms will be randomized to an initial strategy of either anatomic or functional testing. All subsequent decisions regarding additional testing, medications, and/or procedures will be at the discretion of the responsible clinical care team.<br>Within the functional testing arm, the subject's care team will select the specific test to be performed (exercise electrocardiogram, stress nuclear imaging, or stress echocardiogram) consistent with "usual care" in that practice setting. The subject's care team will be provided with "Information sheets" summarizing current standards for test interpretation and preventive care, but specific medical treatment will not be mandated by the trial.
**Duration of Study Participation**	The trial will have an approximate 36-month enrollment period and 1-year minimum follow-up period. All subjects will be followed from enrollment until either death or the end of the follow-up period.
**End-of-Study Definition**	1-year minimum follow-up of all patients, with expected 24-month average follow-up for clinical status assessment.
**Number of Subjects**	Approximately 10,000 subjects will be enrolled.
**Number of Sites**	~220
**Study Follow-up**	The sites will conduct the initial study follow-up at 60 (+/-14) days. Follow-up contact at 6 months post-randomization and every 6 months thereafter will be conducted centrally by the Duke Clinical Research Institute or its designees.

A2 ***Primary Hypothesis***

The PROspective Multicenter Imaging Study for Evaluation of Chest Pain (PROMISE) hypothesis is that an initial anatomic testing strategy utilizing 64-slice or better coronary computed tomographic angiography (CTA) technology in subjects with low-to-intermediate risk for coronary artery disease (CAD) will reduce the composite primary endpoint (all-cause death, myocardial infarction [MI], major peri-procedural

complications, and hospitalization for unstable angina) when compared with an initial functional testing strategy over an average of 2.5 years of follow-up.

A3 *Significance of the Study*

PROMISE is the first large randomized trial comparing the 2 major alternative noninvasive diagnostic strategies for the initial assessment of stable symptoms suspicious for possible CAD. Community-based practices are anticipated to contribute substantially to subject enrollment, which will enhance generalizability of findings in contrast to previous smaller studies that relied on “expert” centers staffed by cardiologists and radiologists. This trial will provide an unbiased comparison of usual-care testing strategies with new CTA technology that is expected to yield definitive and unique evidence regarding the benefits and risks of these alternative approaches, with the primary endpoint being superior health outcomes.

B Background

B1 *Prior Literature and Studies*

1.a Current Management of Patients with Chest Pain: Extent of the Problem

Chest pain is a common presentation, with ~4 million Americans newly diagnosed with angina[1] and more than 6 million presenting to emergency rooms with chest pain annually.[2] The annual incidence of new angina ranges from 1% in middle-aged women to nearly 4% in elderly men in the U.S.,[1] with rates in diabetics of up to 10%.[3] Overall mortality is doubled compared with the general population, and rates range from less than 1% per year for those ultimately diagnosed as "nonanginal" to almost 10% per year for those with CAD and an unfavorable clinical risk profile.[4-8] (See Table 1.) These data suggest that between 4 and 5 million Americans require noninvasive testing for chest-pain symptoms every year. The high event rates in unselected but symptomatic populations support performance of a randomized trial to define optimal testing strategies in this at-risk group and to guide care.

Table 1: Event Rates in Populations with New Onset Chest Pain

Study	Population	Endpoint	Lowest event rates	Highest event rates	Follow-up
Daly BMJ 2006[4]	3,031 (58%M)	Death, MI	2.3% overall	3.9% with angiographic CAD	1 year
Mudrick Circ 2009[8]	98,872	Death, MI, Stroke	1.07%	N/A	1 year
Sekhri Heart 2007[9]	8,762 (52%M)	Death, MI, Unstable angina hospitalization	0.83% noncardiac CP	8.62% angina	1 year
			2.73% noncardiac CP	16.52% angina	3 years
Sekhri BMJ 2008[5]	8,176 (53%M)	Coronary death, MI	3-9% nonspecific CP	16-19% typical CP	2.5 years
Clayton BMJ 2005[6]	7,311 (79%M)	Death, MI, stroke	4% lowest risk decile	35% highest risk decile	5 years

MI=myocardial infarction; CAD=coronary artery disease; N/A=not applicable; CP=chest pain

In patients presenting with symptoms suggestive of CAD, such as chest pain or exertional dyspnea, the clinical risk profile and presentation are often insufficient to definitively exclude CAD and/or are not sufficient to implement invasive evaluation or revascularization without additional information. Thus, noninvasive diagnostic testing is required in the majority of such patients. Consistent with these data, an exercise electrocardiogram (ECG) was performed in 65% of all British chest-pain patients,[7] rising to over 90% in those eventually diagnosed with angina. In the Euro Heart Survey of Chronic Stable Angina, 76% of patients received an exercise ECG, while 18% had a stress imaging study, and 64% had a resting echocardiogram. A coronary angiogram was performed or planned in 41%.[10]

1.b Current Diagnostic Strategies Using Functional Tests are Inadequate

American College of Cardiology (ACC)/American Heart Association (AHA) Guidelines and current noninvasive diagnostic testing includes exercise ECG, stress echo and stress nuclear imaging, all of which are based on detecting stress-induced myocardial ischemia caused by obstructive CAD.[11-13] All are also associated with high rates of

false-positive and false-negative results in the detection of significant CAD (defined as a left main obstruction greater than or equal to 50% and/or any major epicardial vessel obstruction greater than or equal to 70%), limited prognostic discrimination, relatively short “warranty period” for a negative test, and an inability to detect possibly significant nonobstructive CAD. The literature regarding functional test characteristics is summarized below in Table 2, along with reports of clinical event rates after testing.

False-negative test results may delay needed treatments and lead to worse clinical outcomes, while false-positive test results may lead to unnecessary treatment and procedures. Evidence of the inadequacies of current diagnostic strategies includes the occurrence of undetected MI (2.1%) and unstable angina (2.3%) in patients with suspected acute coronary syndrome (ACS) who are discharged from the emergency room.[14] A missed diagnosis of MI was associated with a 9.8% 30-day mortality vs. only 5.7% in hospitalized patients, or a relative risk of 1.9. Other relevant evidence comes from an analysis of the large ACC National Cardiovascular Data Registry, which suggests that misdiagnosis of chest pain is common in community practice.[15] Of nearly 400,000 patients without known CAD who underwent elective diagnostic catheterization, 84% had prior noninvasive testing, which was positive in 82%. Nevertheless, only 38% of patients overall, and 41% of those with positive tests, had obstructive CAD (left main stenosis greater than or equal to 50%; major epicardial vessel greater than or equal to 70%). Thus current clinical practice, as guided by functional stress testing, results in a pattern of referral to invasive angiography, which does not lead to revascularization in the majority of patients, despite the inherent risks and expense of an invasive test.

1.c Computed Tomographic Angiography is Highly Accurate in Detecting and Excluding Obstructive and Nonobstructive Coronary Artery Disease

The recent technological advance of coronary CTA permits direct visualization of the coronary arteries, allowing noninvasive detection of significant stenoses and with great accuracy.[16-19] Computed tomographic angiography also extends the spectrum of disease by detecting nonobstructive lesions and visualizing coronary plaque, important prognostic predictors. Computed tomographic angiography test characteristics and associated event rates are shown in Table 2 below. The superior ability of CTA to detect obstructive CAD means that patients at greatest risk will be more accurately identified and treated with life-saving therapies proven to reduce death and MI by about 30%,[20] while those without disease will not be subjected to unnecessary additional, perhaps invasive, testing.

Table 2: Diagnostic Test Characteristics and Resulting Event Rates

	Stress Nuclear	Stress Echo	Exercise ECG	CTA
**TEST CHARACTERISTICS** (CAD with stenosis ≥ 70%; LM ≥ 50%)				
Sensitivity	76%–94%[21-24]	87%–88%[21-24]	52%[21]	85%–99%[25]
Specificity	62%-88%[21-24]	77%-84%[21-24]	71%[21]	82%–95%[25]

Event Rate after Negative Study (Prognostic Accuracy)	0.5%–2.0%/yr[26-30]	0.5%–2.0%/yr[26-30]	3%/yr[5]	< 0.3%–0.4%/yr without plaque; 1.1%without significant stenosis[31-33]
Event Rate after Positive Study (Prognostic Accuracy)				1.6%/yr with any plaque; 11.9% with any stenosis[31]
Detection of Nonobstructive CAD	N/A	N/A	N/A	Yes[31]
**OVERALL EVENT RATES** (1 yr; all patients)				
Death	0.45%–2.5%[34-37]	2.9%–6.6%[38-40]	0.5%–5.0%[41-43]	0.3%–2.2%[32,33,44-47]
Nonfatal MI	0.9%–1.3%[34-37]	0.9%–1.3%[38-40]	-	0.4%–0.6%[32,33,44-47]
Unstable Angina Hospitalization	3.8%	-	-	0.2&–4.2%

Echo=echocardiogram; ECG=electrocardiogram; CTA=computed tomographic angiography; CAD=coronary artery disease; LM=left main; yr=year; N/A=not applicable; MI=myocardial infarction

1.d Additional Important Considerations in Comparing Test Characteristics

Limited Evidence Base to Guide Diagnostic Testing

The majority of the studies discussed above were conducted in single academic centers and are plagued by numerous sources of bias (verification, selection, etc.), making evaluation of true accuracy very difficult.[48,49] Data from academic research settings provide only a rough indication of actual performance in the real world.[50-52] Even these data, however, are based on convenience samples of subjects already referred for a particular test rather than the true population of interest.[50] The absence of high-quality unbiased comparative data on test performance and outcomes has led to the unusual situation of conflicting ACC/AHA Guidelines, with each of these relevant documents recommending the initial use of a different test: treadmill ECG, stress echo, or stress perfusion imaging.[11-13,53,54] Further, very few studies have addressed Fryback and Thornberry's highest levels of evidence for assessing diagnostic testing: subject outcome efficacy and societal efficacy.[55] This situation has resulted in clinical confusion, performance of multiple tests, and marked variation in clinical practice that can only be addressed by high-quality effectiveness research based on improving patient outcomes.

Obstructive CAD Relatively Uncommon; Nonobstructive CAD Common, Clinically Important, and Difficult to Detect with Functional Tests

The National Cardiovascular Data Registry (NCDR) analysis shows that the absence of significant obstructive disease is common in patients undergoing diagnostic

catheterization. Of the 62% of patients without significant CAD, two-thirds had nonobstructive CAD (20%-70% epicardial and less than 50% left main) and one-third had no CAD (defined as stenosis less than 20%).[15] A similar pattern has been noted by others: in 400 patients at low-intermediate risk for CAD undergoing CTA, 47% had nonobstructive CAD, 13% had obstructive stenoses, and 27% had no disease, with 13% of scans being inconclusive.[45] These data are similar to Ostrom et al[33] and the Michigan Advanced Cardiovascular Imaging Consortium findings. In this registry of over 4000 subjects very similar to PROMISE's target population, it was noted by the investigators that 40% had nonobstructive disease, 41% had normal anatomy, and only 14% had lesions greater than or equal to 70% (written communication from Gil Raff, 2008). Finally, only 14% of the subjects in a multicenter CTA study, Assessment by Coronary Computed Tomographic Angiography of Individuals Undergoing Invasive Coronary Angiography (ACCURACY), had obstructive disease (defined as stenosis greater than or equal to 70%) on catheterization.[16] Thus, current clinical data suggest that nonobstructive CAD will be frequent and obstructive CAD relatively uncommon in the PROMISE cohort.

Long-term outcomes in subjects with nonobstructive disease on angiography, such as was found in 39% of the NCDR cohort,[15] have been found to be intermediate between subjects with obstructive CAD or with no disease.[56,57] This is consistent with the known prognostic power of coronary calcium scoring,[56] and an observed 2x increase in death in patients with nonobstructive disease compared with those with normal coronary anatomy on CTA.[33,45] The knowledge that most MIs arise from nonobstructive lesions[58,59] provides biologic plausibility to these observed differences in outcomes. Since functional testing relies upon inducing ischemia due to limited coronary flow reserve in the presence of obstructive lesions, it cannot detect potentially important nonobstructive disease. Patients receiving these tests will have undetected nonobstructive disease. In contrast, nonobstructive disease is routinely detected by CTA, yielding information that may be important for properly adjusting the intensity of preventive therapy to reflect the presence of coronary plaque.

Therefore, in response to these needs, the PROMISE trial, a multicenter, randomized, pragmatic trial, is comparing 2 state-of-the-art diagnostic strategies in symptomatic subjects at clinically determined, low-intermediate risk for CAD who require nonurgent testing. The "anatomic" testing strategy uses coronary CTA (greater than or equal to 64-slice) as the initial test. The "functional" stress testing strategy uses physician-selected stress imaging (echo or nuclear) or exercise ECG as the initial test. Ten thousand subjects will be randomized over approximately 36 months and followed for a minimum of 1 year (average approximately 2 years). All subsequent diagnostic and therapeutic management decisions will be based on the latest clinical practice guideline recommendations and will be at the discretion of the treating care team.

B2 *Rationale: The Urgent Need for a Randomized Trial of Diagnostic Strategies in Subjects with Suspected Coronary Artery Disease*

Millions of patients undergo different diagnostic testing for suspected CAD in the U.S. every year despite little or no evidence that it will improve their outcomes. The remarkable imprecision and inefficiency of current evaluation strategies is perhaps best documented by the high rate (up to 60%) of invasive coronary angiograms in which no

significant CAD is detected,[15] while the U.S. economic burden of caring for patients with chest pain exceeds $75 billion/year.[60] Limitations of current tests, an inadequate evidence base, and escalating costs have led all major professional societies, Centers for Medicare and Medicaid Services, and National Institutes of Health to publicly demand the development of new evidence to optimize the clinical use of diagnostic testing, reduce unnecessary invasive procedures and control costs. Further, all stakeholders have called for further investigation of the appropriate role of coronary CTA, a novel diagnostic imaging technology that may potentially change the standard of care in this common but challenging situation.[48,52,61-63]

The problem at hand must be addressed by an effectiveness trial, as examination of efficacy only (i.e., optimal performance at expert sites) would fail to capture the actual performance of diagnostic strategies in the community where the vast majority of these tests are performed and where future clinical decisions based on trial results will impact patient outcomes. The concept of a pragmatic clinical trial, as proposed by Tunis et l,[64] is an ideal format to acquire the quality of evidence required to address the needs of clinical decision makers. The PROMISE study embodies all the characteristics of such a trial, including (1) incorporation of clinically relevant alternatives, including usual clinical testing and usual clinical care; (2) a diverse study population; (3) heterogeneous practice settings; and (4) use of a broad range of health outcomes rather than focusing solely on cost. Each of these features is essential to establish an evidence-based standard of care for the large number of patients who require diagnostic testing for symptoms of CAD.

Thus, the ideal approach to addressing these problems is a randomized trial of different testing strategies that is adequately powered to demonstrate superior clinical outcomes, as only improved patient health would be sufficiently motivating to change practice and justify the routine use and reimbursement of a new technology. Because test-reimbursement policies can be expected to evolve and change in response to many nonmedical forces in the economy, conclusions based primarily on the differential costs of alternative testing strategies are unstable over the long term. The ideal trial must also directly target the clinical outcomes that are most relevant to assessing value. Finally, the ideal trial must ensure the broadest possible applicability of its results by demonstrating real-world effectiveness across the appropriate spectrum of tests, practice settings, and caregiver specialties and expertise, which are pertinent to the clinical decisions needed to care for the vast majority of chest-pain patients nationwide. The PROMISE trial design has been shaped by these objectives.

C Study Overview and Objectives

C1 *Overview of the PROMISE Trial*

PROMISE is a multicenter, randomized, pragmatic trial comparing 2 state-of-the-art diagnostic strategies in approximately 10,000 symptomatic, low-to-intermediate–risk subjects with suspected CAD who require nonurgent testing. One testing arm will use an “anatomic” testing strategy with coronary CTA (greater than or equal to 64-slice) as the initial test. The other arm, or “functional” testing strategy, will use either stress imaging (echo or nuclear) or exercise ECG as the initial test. All subsequent diagnostic and therapeutic management will be at the discretion of the treating care team. The trial will encourage adherence to evidence-based practice and document actual therapies used, but will not mandate specific care plans, which will be left to the discretion of the clinical

care team. Subjects will be randomized over approximately 36 months and followed for 12 to 36 months (or until the study ends) at 200 to 250 North American primary-care, cardiology, and acute-care practice sites, reflecting the physician specialties and community settings where the vast majority of chest-pain patients receive care.

C2 *Primary Aim*

The primary aim of PROMISE is to determine if an initial anatomic testing strategy with CTA (greater than or equal to 64-slice; anatomic care) in symptomatic subjects with low-to-intermediate risk for CAD will reduce the composite primary endpoint (all-cause death, MI, major peri-procedural complications, and hospitalization for unstable angina) when compared with an initial functional testing strategy (stress echo, stress nuclear, or exercise ECG), over an average of 2.5 years of follow-up (range approximately 2–4 years, or until the end of the study).

The primary endpoint is time to the first event in a composite of major cardiovascular events including:

- Death
- MI
- Major complications from cardiovascular procedures and testing (stroke, major bleeding, anaphylaxis, and renal failure requiring dialysis)
- Unstable angina hospitalization

C3 *Secondary Aims*

The secondary aims are to compare the following clinical and economic outcomes in subjects randomized to initial anatomic versus functional diagnostic testing:

- Death or MI or unstable angina hospitalization
- Death or MI
- Major complications from cardiovascular procedures and testing (stroke, major bleeding, anaphylaxis, and renal failure requiring dialysis)
- Composite endpoint of the primary endpoint or invasive catheterization without obstructive CAD
- Invasive catheterization without obstructive CAD (Defined as no stenosis >= 50% in any major epicardial vessel including side branches ≥ 2 mm in diameter, on the first cath performed <= 60 days after randomization))
- Medical costs, resource use, and incremental cost effectiveness
- Health-related quality of life (QOL)

In addition, cumulative radiation exposure will be assessed as a secondary safety endpoint.

C4 *Rationale for the Selection of Outcome Measures*

Rationale for Clinical Assessments

Because patients with CAD remain at risk for major adverse events related to their disease over their lifetimes, consideration of long-term outcomes is essential to exploring the impact of test selection on health outcomes and costs. The endpoints of all-cause death, nonfatal MI, major complications related to cardiovascular procedures, and unstable angina hospitalizations are clinically relevant and together reflect the success or failure of the diagnostic testing strategy to accurately detect or exclude disease, guide procedural care, and provide useful long-term prognostic information with high confidence. Any event that could potentially be influenced by the anatomic information provided by CTA, such as percutaneous coronary intervention (PCI), is excluded as an endpoint regardless of when during the follow-up period it occurred. Conversely, major peri-procedural complications are included, as these are a potentially important cause of harm resulting from a need for an invasive study due to inaccurate initial diagnostic tests. The use of a composite clinical endpoint and a subject follow-up of at least 1 year are essential to testing PROMISE's hypothesis of clinical superiority of CTA.

Rationale for Economic and QOL Assessments

The use of noninvasive diagnostic testing for the assessment of CAD has grown at a far faster rate than other medical care, including related procedures such as cardiac catheterization or revascularization.[65] Cardiac diagnostic testing now costs over $4 billion annually for Medicare Part B alone.[66] Although these unsustainable testing costs mandate the development of better evidence, recent efforts by payers to reduce costs have largely addressed the number of tests performed, or payment per test, rather than the value derived for guiding patient care. Thus testing is increasingly driven by onerous administrative practices such as prior authorization designed to discourage physicians from providing indicated care[67] or drastic reductions in reimbursement. This default approach is not in the best interests of either patients or our increasingly dysfunctional health care system.

The studies that have examined the costs and cost effectiveness of functional vs. anatomic testing strategies to date are limited by the lack of reliable long-term effectiveness and cost data.[24,44,68,69] Thus, a formal examination of the incremental cost of testing and its value for patients is urgently needed and is an essential part of the PROMISE trial.

C5 *Rationale for Selection of Testing in Each Experimental Arm*

Functional Testing Arm: Stress Nuclear, Stress Echo, and Exercise ECG

Each of these procedures is a well-established and accepted strategy to diagnose CAD. Each has also been in routine clinical use for over 20 years and is well supported by class I, level of evidence B ACC/AHA practice guideline recommendations for use in suspected CAD. The guidelines further document specific indications as well as diagnostic and prognostic performance. (See also Table 2.) A national claims database (United HealthCare) documenting contemporary U.S. testing patterns shows that stress

nuclear exams constitute 61% of tests, with stress echo at 18% and exercise ECG at 21%. Computed tomographic angiography and stress magnetic resonance imaging comprised less than 1% each. Seventy-nine percent of tests were performed with exercise stress.[8] The lack of a single dominant diagnostic strategy for current practice, even in this insured population, highlights the essential need for flexibility and breadth in the choice of functional tests to fully reflect real-world diagnostic practices and maximize generalizability and clinical impact. Thus it is essential that exercise ECG, stress echo, and stress nuclear imaging are all included in the functional testing arm.

Anatomic Testing Arm: CTA

Because CTA is quick, robust, readily available, and accurate, it may lead to a major practice shift in the evaluation of chest pain where a strategy of imaging coronary anatomy is preferred over the current practice of first searching for inducible ischemia using functional testing. In this sense its widespread adoption would represent substantial change, as it may improve diagnostic testing in an unexpected way (through anatomic rather than functional information) and may alter the indications for testing, thereby expanding the patient population being tested. Further, there is evidence of increasing physician confidence over time,[70] a hallmark of an improved diagnostic testing strategy.[71] This growing experience indicates that the greatest strength of CTA may lie in excluding obstructive CAD in lower-prevalence cohorts—exactly the symptomatic population being studied in PROMISE.[72] Finally, because of its visual strength, CTA has an inherent important motivational ability that has been shown to improve prescription and adherence to effective preventive strategies, an important goal of any testing strategy.[73] On the other hand, CTA cannot be performed in all patients, as calcified lesions and temporal and spatial resolution can be limiting. However, evidence suggests that CTA is superior to functional testing in reducing false-positive and false-negative test results, identifying nonobstructive disease, improving the prognostic accuracy of a negative test, and extending the “warranty period” of test results, all of which strongly suggest clinical superiority over functional testing.

D Investigational Plan

D1 *Overview of Trial Design*

PROMISE is a multicenter, randomized, pragmatic trial comparing 2 state-of-the-art diagnostic strategies in approximately 10,000 symptomatic, low-to-intermediate CAD-risk subjects with suspected CAD who require nonurgent testing. The following discussion includes trial assessments, outcomes, substudies, organization and operations, sites and site management, timeline, and potential criticisms. See the figure below for a schematic summary of the trial design.

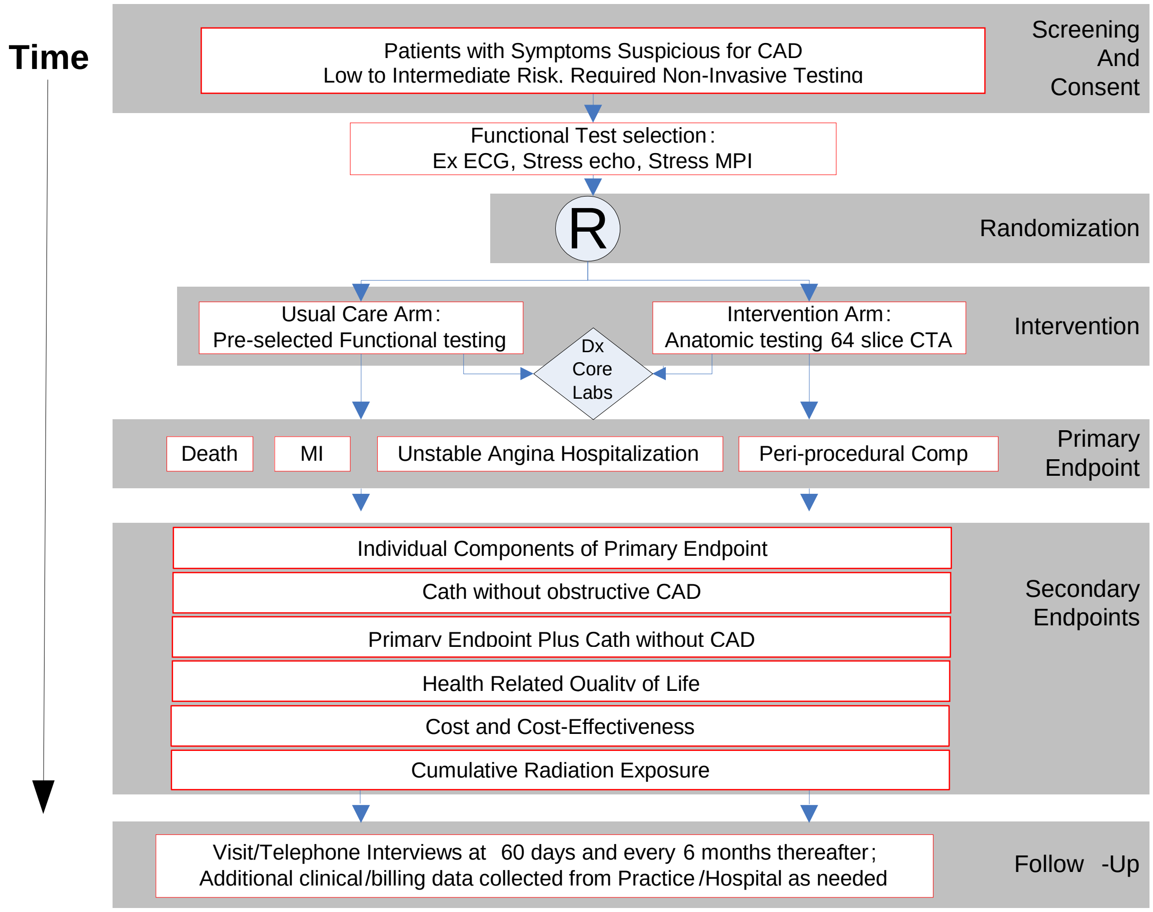

D2 *Subject Population*

To optimize the generalizability of the study results and reflect current patterns of care, subjects considered for enrollment into PROMISE will be outpatients without known CAD who are symptomatic, and whose physician has decided that the subject requires nonurgent, noninvasive cardiovascular testing to further evaluate suspected CAD. This population is purposefully and carefully chosen to be directly reflective of the population in which elective noninvasive cardiac diagnostic testing is currently being used.

PROMISE's inclusion criteria stipulate that all subjects will be symptomatic and will, in the judgment of the physician caring for the subject, require an elective noninvasive test

for symptoms suspicious for CAD. Thus, whether or not the subject chooses to participate in the trial, he or she will likely undergo testing. All of the modalities in PROMISE are clinically well established and performed routinely and safely across the U.S. No experimental treatment or testing is involved, and there should be equipoise about the feasibility to perform either functional OR anatomic testing. **The trial intervention is simply the random assignment of the initial test.**

Justification of Population

Since subjects are symptomatic and have already been determined to require outpatient noninvasive testing, this is an ideal population in which to compare the results of different testing strategies. We include subjects presenting in primary care offices, cardiology offices, and urgent care settings without suspected ACS to ensure inclusion of all subject profiles; however, patients undergoing testing as part of a rule-out-ACS protocol are not eligible. To ensure broad generalizability, we are using a patient-centric design and not limiting enrollment to a single care setting or single type of provider (cardiology). PROMISE reflects the care settings, patient groups, and providers who will eventually apply its results.

2.a Inclusion Criteria

1. New or worsening chest-pain syndrome or equivalent symptoms suspicious for clinically significant CAD
2. No prior cardiac evaluation for this episode of symptoms
3. Planned noninvasive testing for diagnosis
4. Men age greater than or equal to 55 years and women age greater than or equal to 65 years
5. If age in men 45–54 years or women 50–64 years, then must have increased probability of CAD due to 1 or more of the following risk factors:
    - Diabetes mellitus requiring medical treatment
    - Peripheral arterial disease (PAD), defined as documented peripheral arterial stenosis greater than or equal to 50%, treated medically or invasively
    - Cerebrovascular disease (stroke), defined as documented carotid stenosis greater than or equal to 50%, treated medically or invasively
    - Ongoing tobacco use
    - Hypertension
    - Abnormal ankle-brachial index (ABI), defined as less than 0.9
    - Dyslipidemia
6. Serum creatinine less than or equal to 1.5 mg/dL within the past 90 days
7. Negative urine/serum pregnancy test for female subjects of childbearing potential

2.b Exclusion Criteria

1. Diagnosed or suspected ACS requiring hospitalization or urgent or emergent testing; elevated troponin or creatine kinase-myocardial band (CK-MB);

outpatients who have completed a rule-out ACS protocol are eligible provided they have negative biomarkers x 2 and a nondiagnostic or normal ECG.

2. Hemodynamically or clinically unstable condition (systolic blood pressure [BP] less than 90 mm Hg, severe atrial or ventricular arrhythmias, or persistent resting chest pain felt to be ischemic despite adequate therapy)
3. Known CAD with prior clinical history of MI, PCI, coronary artery bypass graft (CABG) or any angiographic evidence of CAD greater than or equal to 50% lesion in a major epicardial vessel
4. Any invasive coronary angiography or noninvasive anatomic or functional cardiovascular test for detection of CAD, including CTA and exercise ECG, within the previous 12 months (+/- 30 days); prior resting ECG and/or resting echo do not constitute an exclusion to participation
5. Known significant congenital, valvular (greater than or equal to moderate) or cardiomyopathic process (hypertrophic cardiomyopathy or reduced systolic left ventricular [LV] function [LV ejection fraction less than 40%]) that could explain cardiac symptoms
6. Contraindication to a CTA, including, but not limited to:
    a. Allergy to iodinated contrast agent
    b. Pregnancy
7. Any other contraindications that would preclude performing a CTA per local site practice, such as 1 or more of the following:
    a. Inability to receive beta blockers if heart rate is greater than 65 beats per minute
    b. Agatston score greater than 800
    c. Body mass index (BMI) greater than 40
    d. Cardiac arrhythmia
8. Life expectancy less than 2 years
9. Unable to provide written informed consent or participate in long-term–follow-up

2.c Ethical Considerations

Human Subjects' Involvement and Characteristics

All human adult subjects who meet inclusion criteria and who do not meet any of the exclusion criteria will be considered eligible for this trial. Subjects who are within vulnerable populations will be included at the discretion of the site institutional review boards (IRBs).

2.d Subject Recruitment Plans and Consent Process

Diagnostic testing for the assessment of CAD symptoms is ordered by physicians of all specialties; is performed in multiple settings, including physician offices, hospital outpatient departments, and diagnostic testing facilities; and is interpreted by physicians of multiple specialties, including primary care physicians, cardiologists, and radiologists. Therefore, subjects will be recruited from multiple clinical settings, including outpatient

clinics, urgent care centers, and testing facilities. Sites that do not perform the full range of diagnostic testing, including CTA, will be paired with regional referral centers for echo, nuclear, and/or CTA testing where subjects may undergo the necessary testing. Existing clinical referral and research networks will be used to identify and enroll subjects.

Subjects will be recruited from 200 to 250 clinical sites. All potential PROMISE sites will obtain IRB/Institutional Ethics Committee (IEC) approval of the protocol and the associated consent form and any recruitment tools. Written informed consent will be obtained from each subject before to any study procedures are performed. Assessment of CAD risk will begin during screening to ensure eligibility. It will include medical history and physical exam and laboratory testing for the presence/absence of major cardiac risk factors, including BP/hypertension, diabetes, cholesterol (low-density lipoprotein [LDL], high-density lipoprotein [HDL]), smoking, family history, sedentary life style, obesity, cerebrovascular and PAD history, and ABI.

Subjects will be identified and enrolled in a step-wise process as follows:

1. All subjects being considered for outpatient noninvasive testing for the initial evaluation of symptoms suspicious for clinically significant obstructive CAD will be screened.
2. Subjects meeting inclusion criteria and not having any of the exclusion criteria will be approached to participate in the study and have the study explained to them.
3. Subjects agreeing to participate after having all their questions answered will be asked to document their agreement on the study-specific IRB-approved consent form with a signature.

2.e Subject Randomization

Eligible subjects who have given written informed consent and meet all inclusion and no exclusion criteria will be randomly assigned in equal proportions (1:1) to either the anatomic or functional diagnostic testing arm of the trial. A computer-generated permuted block randomization schedule with stratification by clinical site will be used in the trial. Before randomization, the managing caregiver, site investigator, or authorized designee will be asked to indicate the functional test he or she would plan to use if the subject were randomized to the functional testing arm. This information will be tracked and used as another stratification factor in the randomization scheme to facilitate comparisons of anatomic vs. functional testing according to the type of functional test. Subject randomization will be accomplished by telephone through a centralized toll-free interactive voice response system (IVRS).

If a subject is randomized but does not undergo the planned initial diagnostic test within 30 days of randomization, that subject will still be followed and included in the intention-to-treat statistical comparisons according to his or her randomized treatment assignment.

2.f Risks and Benefits

Potential Risks

Protocol-specific risks associated with the PROMISE trial are minimal. All PROMISE enrolled subjects will, by inclusion criteria, require noninvasive testing for their symptoms. Thus all subjects will have compelling medical reasons for performance of

the noninvasive test arm to which they are randomized. All care in PROMISE is part of routine clinical practice and, because the only intervention being performed in PROMISE is the random assignment of initial diagnostic test, safety considerations and adverse events are limited to complications arising from initial testing. Reporting of these safety events will be the responsibility of the site investigator.

Drawing Blood

Risks associated with drawing blood include momentary discomfort and/or bruising, infection, excess bleeding, clotting, or fainting.

Potential Loss of Confidentiality

In any clinical trial, there is a possible risk to subjects as to the potential loss of confidentiality. To prevent this from occurring, the Clinical Coordinating Center (CCC) and the Statistical and Data Coordinating Center (SDCC) at the Duke Clinical Research Institute (DCRI) have strict procedures in place to ensure that all study data are confidential and anonymized except as required for data collection by the Economics and Quality of Life Coordinating Center (EQOL CC) and the DCRI Outcomes and Follow-up Group (DOFG). For all data received by DCRI, subjects will be identified only by unique code numbers. The link to these codes will be maintained at the SDCC. Trial records that identify subjects will be kept secure and confidential as required by law. Federal privacy regulations provide safeguards for privacy, security, and authorized access.

If the DOFG finds that a subject has undergone diagnostic testing or a diagnostic or therapeutic procedure or has been hospitalized, the DOFG will obtain the subject's test results or discharge summary, and the EQOL CC will obtain relevant medical billing information on behalf of the trial. Once received at the SDCC, these documents will be anonymized, removing the subject's name, personal identifiers, and local physician's name and will be identified by unique study enrollment numbers.

Potential Benefits

The PROMISE trial results should improve the care of future subjects requiring diagnostic testing for suspected CAD. In addition, the trial will deliver high-quality data on radiation exposure, incidental findings, and other clinically important "side effects" of the testing strategies that will be examined in a large real-world experience.

D3 *Diagnostic Testing*

3.a Approach to Diagnostic Testing

An important goal of PROMISE is the broadest possible applicability of its results. Therefore, all commonly used tests are included in the functional testing arm, a designation that generally implies a multiplicity of approaches. This inclusiveness will facilitate enrollment by ensuring that all eligible subjects will have ready access to high-quality testing in both arms. Because the preferred type of functional test will be recorded by the IVRS before randomization, the trial subjects, each of whom has been selected by his or her managing clinician to undergo a different form of functional testing, can later be divided into 3 cohorts for prespecified subgroup analyses. In each cohort, half will be randomized to undergo that prespecified functional test, while the other half

will be randomized to undergo CTA, forming paired CTA-functional test subgroups for each functional test type.

We will ensure that the standard of care of testing and imaging performance and interpretation consistent with good medical practice is maintained throughout the trial by requiring that sites have expertise and qualify in all modalities, by providing recommended imaging protocols and by providing timely quality feedback and expert overreading in a subset of studies, again for all modalities.

3.b Description of Testing To Be Performed

Subjects will initially be evaluated according to their physicians' specific protocols for management of subjects with an indication for nonurgent, noninvasive cardiovascular testing. Typically, the standard evaluation will include the relevant past and current medical history, a physical examination, and a resting 12-lead ECG and may include cardiac biomarkers (troponin and/or CK-MB) as well as other routinely obtained blood testing. Subjects who meet eligibility criteria will have their physicians' preferred functional tests designated as part of the stratification requirements for randomization.

Functional Testing Arm

For subjects randomized to usual care, the preselected functional test will be performed as the initial test. This includes stress nuclear imaging, stress echo, or exercise ECG.

Anatomic Testing Arm

In subjects randomized to the anatomic testing arm, a contrast-enhanced coronary CTA will be performed as the initial test.

Subsequent Care

The results of all tests will be provided to the care team in the usual manner for that testing laboratory, and, depending on the results, subjects may or may not undergo medical treatment and/or additional noninvasive functional, anatomic, or invasive testing (coronary angiography), and/or coronary revascularization, at the care team's discretion. It is assumed that patient management will be additionally informed and guided by the test findings. The local physician will resume care of the subject and make all subsequent clinical decisions (e.g., need for further evaluation or admission) based upon his or her cumulative clinical assessment of the subject, including findings revealed on the noninvasive testing.

3.c Functional and Anatomic Testing and Cardiac Catheterization

Equipment and Protocols

Criteria for qualification of participating sites or referral laboratories will include use of standard equipment for usual-care testing (stress echo, stress nuclear, and exercise ECG) as defined in current practice guidelines, and greater than or equal to 64-slice MDCT technology for coronary CTA.[12,74-77]Similarly, all test acquisition protocols will adhere to best-practice standards as defined in current national practice guidelines.[12,74-77] Sites will be allowed to use their own standard acquisition protocols as long as they

fall within the standard-of-care guidelines. Sample protocols are provided in the Manual of Operations and Procedures (MOP).

Interpretation

All studies will be interpreted by qualified physicians who have at least ACC COCATS (Core Cardiology Training Symposium) level 2 training[78] or equivalent. For nuclear studies, certification by the Certification Board of Nuclear Cardiology or Board Certification in nuclear medicine or radiology is also sufficient to qualify as a reader. Coronary CTA will be interpreted by physicians trained at least COCATS level 2 or equivalent, either Society of Cardiovascular Computed Tomography level 2 or the Certification Board of Computed Cardiovascular Tomography. However, because coronary CTA is a relatively new technology, and practice requirements and expertise may not have evolved to the same level as other diagnostic tests, reader qualification may include online review and evaluation of clinical cases consisting of paired coronary CTA and invasive angiography datasets.

All diagnostic tests will be interpreted and reported by diagnosticians at the sites in real time according to current guidelines to ensure timely availability of results for patient management. The diagnostic test reports will capture the major findings, including:

1. Presence and extent of CAD (CTA).
2. Resting LV function and perfusion (echo and nuclear scans) as either: normal, global dysfunction, regional dysfunction/scar, both, or not interpretable. Ejection fraction will be quantified. This information will be optionally gathered for CTA.
3. Functional capacity determined for exercise ECG, stress echo, or stress nuclear.

3.d Test Transfer and Storage

Test transfer will be accomplished by transmission of paper recordings for stress ECGs and the ECG component of echo and nuclear studies. For digital images including invasive coronary angiography, transmission may be accomplished by compact disc (CD) or over the Web. For Web transmission, an image-transfer system will be deployed at the site. The data-transfer software will provide encryption, lossless compression, and transmission capabilities for the submission of deidentified data sets via the Web to the central Test Data Repository hosted by the American College of Radiology Imaging Network (ACRIN) in Philadelphia.

Upon arrival in the central data repository, datasets will be processed as needed: paper ECGs converted to PDFs; CDs uploaded; and Web images decompressed, deencrypted and imported into the central imaging database, a networked long-term image-archiving and storage system. This will ensure maximal site flexibility as well as seamless and efficient transfer of imaging data from sites into a central data repository and will allow real-time quality control and feedback.

3.e Quality Assurance of Diagnostic Testing

Before beginning enrollment, eligible sites and readers will be qualified by the Diagnostic Testing Coordinating Center (DTCC) based on site and reader surveys, on successful transfer of 1 or more complete data sets with sufficient image quality and completeness for each modality, and participation in the CTA case review. Site qualification will be issued by the DTCC for each modality before subject enrollment.

During the study, technical quality assessment of image and test acquisition will be accomplished on all studies by central repository research technicians trained by the DTCC or by the CCC for invasive coronary angiography. This ongoing review will ensure the adequate quality and completeness of data sets (see details and definitions in the MOP) and will monitor radiation exposure in the CTA arm throughout the trial.

Expert overreading of the initial, randomized, noninvasive tests and first invasive angiogram within 60 days of randomization will be performed in approximately 10% of subjects by modality experts in the DTCC and CCC. To ensure protocol compliance early on and to account for low-accrual sites, the first 2 studies and ~10% of the remaining studies from each site will be overread in each modality. These will be selected by block randomization stratified by testing site and test modality.

In addition, to strengthen the link between site investigator reported invasive catheterization without obstructive disease and angiographic findings, a sample of invasive angiograms determined by site investigators to be without obstructive disease will undergo core lab QCA and the results compared.

Feedback to Clinical Sites, Remediation, and Disqualification

Sites whose performance does not consistently meet the quality criteria for both quality assurance (QA) and radiation exposure will be asked to undergo protocol review and retraining up until March 31, 2013. Sites that fail to improve may be recommended for discontinuation of enrollment.

3.f Subsequent Medical Care

Care following the imaging studies will be provided by the local care physicians at their discretion. However, they will be encouraged to follow established guidelines for the management of CAD or for primary prevention. To this end, test-information sheets specific to each modality will be created and supplied to each enrolling site and physician and to relevant imaging site staff. These will consist of a brief literature review of diagnostic and prognostic indications of various test results.

In addition, primary and secondary prevention information sheets will be created and provided to each enrolling site and physician at the beginning of the trial. These will consist of brief summaries of relevant ACC/AHA Guidelines and will be referenced to the test-information sheets' results.

3.g Testing Risk and Benefits

To be eligible for entry into the study, each subject and his or her health care provider will have considered the risks and benefits of noninvasive testing and will have determined that the incremental information gained outweighs the potential risks. For example, although diagnostic testing provides important diagnostic and prognostic information and is noninvasive, it cannot be used indiscriminately. Associated risks include maximal exercise testing, use of pharmacologic stress and contrast agents, and radiation exposure on the order of 7 to 17 mSv for stress nuclear and CTA (although aggressive dose-reduction strategies can reduce this to ~2–6 mSv in CTA). Computed tomographic angiography also often involves the use of beta blockers to lower heart rate and an angiographic contrast agent.[79-81] (For comparison, the radiation exposure from a single-view chest x-ray is about 0.03 mSv).

Because the long-term risks and benefits of one form of testing vs. another are unknown, the PROMISE trial is collecting all relevant information to address these knowledge deficiencies, including test complications and estimated total biological radiation exposure for cardiovascular procedures during the duration of the trial. For radiation, doses will be estimated whenever possible using the site's specific scanning protocols for diagnostic testing and fluoroscopic time for invasive procedures. However, if these are unavailable or deemed unreliable, modeled estimates from the sites with such data or from the relevant literature will be used. We will also record the presence of incidental findings as reported on all initial tests and follow-up procedures (including surgical procedures) or testing performed to further evaluate any resulting new diagnoses of significant noncardiac disease.

E Study Procedures

E1 *Screening for Eligibility*

Screening of subjects for this trial will be conducted by the site investigator (or authorized designees) at each participating site. Written, informed-consent documentation will be obtained from each prospective trial subject once study eligibility is confirmed and before the first study procedure.

E2 *Schedule of Assessments*

Subjects will be screened and randomized at or before time of enrollment. Baseline medical history, blood work, and QOL will be assessed. Subsequently, subjects will have either a telephone call or clinic visit at 60 (+/- 14) days for outcome evaluation and recording of any test complications. After that, subjects will be contacted under DOFG supervision by either DOFG or its trained designees at 6 months postrandomization and at 6-month intervals for subsequent follow-up assessments until death, withdrawal, or the end of the trial.

Screening assessments are described below.

TABLE OF ASSESSMENTS	Screening/ Day 1	Day 1-30	Day 60[1]	6 mos	12 mos	18 mos	24 mos	30 mos	36 mos
Informed Consent	X								
Confidential Patient Information Form	X								
Medical History	X		X[2]	X[2]	X[2]	X[2]	X[2]	X[2]	X[2]
Concomitant Medications	X		X	X	X	X	X	X	X
CV Risk	X			X	X	X	X	X	X
Pregnancy Test[3]	X								
Creatinine[4]	X								
Resting 12-lead ECG[5]	X								
Quality of Life[6]	X			X	X		X		
Economics	X			X	X	X	X	X	X
Biomarker Banking	X								
Randomization	X								
Initial Diagnostic Test		X							
Clinical Assessment			X	X	X	X	X	X	X
Endpoint Assessments			X	X	X	X	X	X	X
Test Safety Assessment			X						

mos=months; CV=cardiovascular; ECG=electrocardiogram

[1] +/- 14 days
[2] During medical history review, if subjects have received an additional diagnostic test, a cardiovascular procedure or have been hospitalized since the last visit, additional data will be collected from the practice or institution with subject consent

[3] Pregnancy test required only for female subjects of childbearing potential

[4] Creatinine blood draw required only for subjects without a recent normal value (within previous 90 days)

[5] Resting 12-lead ECG required if none available within past 30 days

[6] Quality of Life assessments will be performed in ~ 6000 subjects enrolled (prior to July 2012).

Day 0/1 Screening/Randomization:

- The subject's relevant medical history (including concomitant medications) will be obtained.
- Coronary arterial disease risk factors will be assessed, including BP/hypertension, PAD (ratio of arm to leg systolic BPs or formal ABI), cerebrovascular disease (carotid bruits), diabetes, cholesterol (LDL, HDL), smoking, family history, sedentary life style, obesity.
- Factors increasing probability of CAD (including diabetes, PVD, and/or other CAD risk factors) will be documented.
- Cardiac symptoms and chest pain descriptors will be assessed.
- Eligible subjects will be asked to give consent for study participation.
- If the subject is of childbearing potential, a pregnancy test will be performed.
- Creatinine blood draw will be done if there is no creatinine measurement within the previous 90 days.

- Subjects will receive a 12-lead resting ECG unless one obtained within the past 30 days is available.
- Subjects will be administered baseline QOL questionnaires at the time of randomization.
- A blood sample will be drawn on subjects participating in biomarker and/or genomic banking (See Section E8).
- The subject will be randomized to the functional test of investigator or care provider choice or CTA. (*Note: functional test of choice must be selected and documented before placing the call to randomize the subject.)*

Days 1-30:

- The functional diagnostic test or CTA will be performed according to the randomized assignment.

Day 60 (+/- 14 days) Postrandomization Follow-up Assessment—site clinic visit or telephone call:

- Relevant interval medical history (including symptoms and concomitant medications) since last assessment will be obtained, including death, MI, major complications from cardiovascular procedure or testing (stroke, major bleeding, anaphylaxis, renal failure), unstable angina hospitalization.
- Test images and stress ECGs from the first test (and the randomized test, if different) will be uploaded to the DTCC. If applicable, the subject's first invasive coronary angiography report and images will be uploaded to the DTCC. All clinical test reports will be sent to the CCC at the DCRI.
- The results of any additional noninvasive tests or invasive catheterizations performed within the first 60 days will be collected.
- Radiation exposure will be assessed.
- Interval resource consumption, including hospitalizations, will be assessed.
- Patient satisfaction will be assessed.

Follow-up at 6 months postrandomization and every 6 months thereafter will be conducted by DOFG staff or its trained designees:

- Relevant interval medical history (including symptoms and concomitant medications) since last assessment will be obtained, including death, MI, major complications from study-related cardiovascular procedures (e.g. catheterization, PCI, CABG, CTA, stress echo, stress nuclear) or testing (stroke, major bleeding, anaphylaxis, renal failure), unstable angina hospitalization.
- Interval radiation exposure will be assessed.
- Interval resource consumption, including hospitalizations, will be assessed.
- Cardiovascular risk modification will be recorded.
- QOL questionnaires will be completed at 6 months, 12 months, and 24 months post-randomization (among patients followed for 24 months).

E3 *Safety*

3.a Data and Safety Monitoring Board

A Data and Safety Monitoring Board (DSMB) will be appointed by the National Heart Lung and Blood Institute (NHLBI) to monitor subject safety and to review performance of the protocol. A DSMB charter that outlines the operating guidelines for the committee and the procedures for the interim evaluations of study data will be developed by the NHLBI and agreed upon at the initial meeting of the DSMB. Reports will be prepared regularly by the DCRI as requested by the DSMB chair. Depending upon the operational plan established by the DSMB, the report might include recruitment and retention rates, interim analyses, primary and secondary endpoints, and other information as requested by the committee. After each meeting, the DSMB will make recommendations to the NHLBI and the trial leadership about the continuation of the study.

3.b Study Coordinating Center or Food and Drug Administration Notification by Investigator

No reporting (other than through the InFORM system) is required.

3.c Definitions of Safety Events and Reporting

Because the only intervention in the trial is the randomized assignment of the initial test to be performed in a symptomatic subject with clinically indicated testing, the only safety events arising from the study are related to the initial test. Mild safety events are considered related to testing only up to 24 hours after the initial randomized test and will be collected and reported by site personnel. The site investigator/designee will document the safety events listed below occurring within 24 hours of initial testing and report them in the InFORM system.

In contrast, those severe events/complications related to cardiovascular testing or cardiovascular procedures that are also trial endpoints (e.g. peri-procedural MI, major bleeding, renal failure, and anaphylaxis requiring circulatory or respiratory support) will be collected throughout the duration of the trial and will be considered to be related to testing or a procedure if occurring within 72 hours.

For CTA:

1. Mild contrast reaction such as rash and hives (severe reactions including anaphylaxis or death are part of the primary endpoint)
2. Extravasation of contrast into the surrounding tissue of the extremity where the intravenous line was placed and contrast administered
3. Hemodynamic instability, including symptomatic bradycardia or hypotension, due to the beta blockade or nitrates given for the CTA scan acquisition
4. Acute bronchospasm due to the beta blockade given for the CTA scan

For exercise testing during exercise ECG, stress echo, or stress nuclear:

1. Exercise-induced hypotension with systolic BP fall greater than 20 mm Hg
2. Stress-induced symptoms that do not resolve within 20 minutes
3. Rapid atrial fibrillation that does not slow or convert with treatment

4. Ventricular tachycardia
5. Hemodynamic instability, defined as systolic BP less than 80 mm Hg
6. Hospital admission not otherwise captured by the primary endpoint, including that precipitated by any symptomatic event (chest pain, dyspnea, etc.), persistent or worsening ischemic ECG changes, any bradycardic or tachycardic arrhythmia, or any hemodynamic changes (hypertension or hypotension)

For stress nuclear:

1. The above events for exercise testing
2. Any events potentially related to the use of vasodilators such as dipyridamole or adenosine, including an anaphylactic reaction to contrast agent not requiring circulatory or respiratory support

For stress echo:

1. The above events for exercise testing
2. Stress-induced wall motion abnormality that does not resolve within 20 minutes (despite treatment)
3. Any anaphylactic reaction to contrast agent not requiring circulatory or respiratory support

E4 *Study Outcome Measurements and Ascertainment*

All-cause Mortality

All-cause mortality is used rather than cardiac mortality to eliminate the need for possibly difficult adjudication of causes of death, especially given the relatively low mortality expected.

Myocardial Infarction

Defined as either 1) an abnormal cardiac biomarker level (either troponin or CK-MB) greater than institutional upper limit of normal (ULN), and either ischemic discomfort lasting greater than 10 minutes or ECG changes indicative of ischemia or infarction, or 2) new abnormal Q waves consistent with infarction. Additionally *peri-procedural infarctions* are defined as greater than 3x ULN for serum CK-MB for PCI and greater than 5x ULN for CABG.

- an abnormal cardiac biomarker level > institutional ULN (either troponin or CK-MB), and either ischemic discomfort lasting > 10 minutes or ECG changes indicative of ischemia or infarction,

OR

- new abnormal Q waves consistent with infarction.

Additionally *peri-procedural infarctions* are defined as >3x upper limit of normal for serum CK-MB for PCI and >5x upper limit of normal for CABG.

Unstable Angina Hospitalization

Defined as 1) ischemic discomfort or equivalent symptoms requiring hospitalization within 48 hours of symptoms, 2) lasting greater than or equal to 10 minutes at rest, or in an accelerating pattern, 3) accompanied by dynamic ST depression, ischemia on stress testing, or significant epicardial coronary artery stenosis, and 4) considered to be myocardial ischemia upon final diagnosis.

Is defined as an event in which the final diagnosis is unstable angina or acute coronary syndrome due to myocardial ischemia and has the following criteria:

- ischemic discomfort or equivalent symptoms requiring hospitalization within 48 hours of symptoms, lasting ≥ 10 minutes at rest

OR

- ischemic discomfort or equivalent symptoms occurring in an accelerated pattern within 48 hours of hospitalization

AND ANY OF THE FOLLOWING

- accompanied by dynamic ST depression
- ischemia on stress testing
- significant epicardial coronary artery stenosis

Major Complications From Cardiovascular Procedures and Diagnostic Testing That Occur Within 72 Hours

Defined as:

- Stroke is defined as an acute focal neurological deficit of sudden onset, not reversible within 24 hours, or that resolves in less than 24 hours with clear evidence of a new stroke on cerebral imaging.

- Bleeding is defined as major based on 1 or more of the following:
  - Transfusion of greater than or equal to 2 units heterologous packed red blood cells or whole blood
  - Decrease in hemoglobin level by greater than or equal to 2.0 g/L
  - Need for reoperation or invasive intervention (*e.g.* evacuation of wound hematoma)
  - Bleeding at a critical anatomic site (intracranial, intraspinal, intraocular, retroperitoneal, intraarticular, pericardial, or intramuscular with compartment syndrome)

- Renal Failure is defined as new requirement for renal replacement therapy.

- Anaphylaxis is defined as a severe contrast reaction requiring emergency respiratory and/or circulatory support.

Invasive catheterization without obstructive coronary artery disease

Only the results of the initial (first) invasive cardiac catheterization that occurs within the first 60 days following randomization will be considered for the secondary endpoints which include invasive angiography without obstructive disease. The absence of obstructive disease is defined as no stenosis >= 50% in any major epicardial vessel including side branches ≥ 2 mm in diameter.

Radiation Exposure Safety Endpoint

Cumulative radiation exposure will be collected as follows:

- CTA: the actual administered dose (computed tomography dose index volume and dose length product for CTA) will be recorded by sites and confirmed by extracting information directly from the images.
- Nuclear imaging: the injected/administered contrast agent dose will be recorded and standard tables used to convert into equivalent doses for appropriate comparison with CTA.
- Invasive coronary angiography and intervention: the actual administered radiation dose or fluoroscopy time will be recorded by sites and standard tables used to convert into equivalent doses for appropriate comparison with CT.

These measures will ensure accurate calculation of the actual cumulative radiation exposure in each arm for tests performed for the diagnostic work-up during the 60 days following enrolment. In addition, we will capture all cardiac diagnostic testing involving radiation performed during the entire follow-up period (CTA, nuclear cardiology, catheterization) and will estimate cumulative radiation exposure over the entire trial using our original data collection (average dose per test for each site) to extrapolate radiation exposure during follow-up.

E5 *Independent Clinical Event Adjudication Committee*

An independent clinical event adjudication committee will review and adjudicate all primary endpoint events and the catheterization secondary endpoints in a blinded fashion based on the definitions presented above. If the invasive cardiac catheterization report is inconclusive, the CEC will review the catheterization films for a visual assessment of CAD. The other clinical secondary endpoint events and cumulative radiation exposure will not be adjudicated.

Primary outcome events will be documented through 60 (+/- 14) days after randomization by the site Investigator or authorized designees. After that, the DOFG or its designees will be responsible for documenting study events from 6 months until the end of the study.

E6 *Quality-of-life Assessments*

Baseline QOL interviews will be administered to ~6000 subjects enrolled (prior to July 2012) by site personnel as soon as possible after consent, preferably before the subject's randomization. Completed questionnaires will be sent directly to the EQOL CC for data processing. Follow-up QOL questionnaires will be administered via structured telephone interview by DOFG trained and supervised interviewers at 6 months, 12 months, and 24 months post-randomization (among patients followed for 24 months). Proxy QOL questionnaires will be used when a subject has died in the follow-up interval or has become incapacitated; these questionnaires will include items that can be reliably obtained from a relative, caretaker, or medical record. Proxy questionnaires will be used if the subject is unable to participate in follow-ups via telephone interview.

Content of Health-related Quality-of-life Questionnaires

A battery of validated instruments will be used that build on a disease-specific core supplemented with generic measures to provide a comprehensive but brief assessment of health-related QOL.

Chest-pain–specific symptoms will be measured using the **Seattle Angina Questionnaire (SAQ)**, a 19-item instrument that assesses 5 dimensions of the impact of chest pain on QOL: physical limitations, angina stability, angina frequency, treatment satisfaction, and disease perception. The **Duke Activity Status Index (DASI)**, a 12-item scale that has been validated in cardiac patients against maximal oxygen uptake measured at exercise (VO_2 max), will be used as a disease-specific functional status assessment. The 4-item **Rose Dyspnea Scale** will be used to assess patients' levels of dyspnea with common activities.

The generic core instrument to be used is the **Medical Outcomes Study Short Form (SF-12)**. The SF-12 is composed of 8 scales (physical function, role function–physical, role function–emotional, general health, bodily pain, social function, psychological well-being/mental health, and vitality), a health transitions item, and 2 summary scores. Additionally, the entire scales for general health, psychological well-being, vitality, and social functioning from the SF-36 health survey will be used to provide better resolution of any treatment differences in these domains.

To assess effects of the 2 diagnostic strategies on the prevalence of depression, we will employ the **Patient Health Questionnaire (PHQ)**, a 9-item depression scale that has demonstrated good agreement with the clinical diagnosis of depression.

Patient-specific utilities will be assessed using the **EuroQoL-5D (EQ-5D)**, a standardized generic health-status measure that links specific health states to general population-based utilities. The EQ-5D consists of a 5-dimension health-state assessment, which allows for definition of 243 discrete health states that can be mapped to population utility weights and a self-rating (0-100) "thermometer" of current health-related QOL. The EQ-5D will be collected as part of all QOL questionnaires.

Employment/productivity will include time lost from work and reduced productivity while at work as measured by the 6-item **Stanford Presenteeism Scale** and questions adapted from the NHLBI Bypass Angioplasty Revascularization Investigation Substudy in Economics and Quality of Life.

E7 *Economic Assessments*

Resource-use data to be collected on the study case report form (CRF) will include hospitalizations, emergency department visits, selected cardiac procedures, and tests. Hospital bills (detailed, summary ledger, and UB 04) will be collected by the EQOL CC at the DCRI for all hospitalizations identified throughout the length of the study. They will include care at clinical sites and at institutions not participating in PROMISE. In addition, cost-to-charge ratios will be obtained from each hospital where a PROMISE follow-up hospitalization is reported.

E8 *PROMISE Biorepositories*

8.a Imaging and Electrocardiograms

Since future developments in image interpretation and integration with clinical data will remain important in the diagnosis and management of chronic diseases of CAD, PROMISE will create a unique anonymized “Image and ECG Data Warehouse” with all initial noninvasive test images, stress ECGs, and initial catheterization films, which will be linked to clinical information. Collection and release of data and images will be at the discretion of the study team including NHLBI and is included as part of the subjects’ initial consent.

8.b Blood Biomarkers and Genomics

The PROMISE investigators also believe that an integrated approach to disease characterization and therapeutic responses will play an increasingly important role in the diagnosis and management of chronic diseases such as CAD. Accordingly, subjects will be asked to provide a blood sample for deposit into a biomarker repository at the time of randomization for future assessment of advanced molecular biomarkers (plasma, serum) such as troponin and high-sensitivity C-reactive protein relevant to disease characterization, risk stratification, characterization of treatment response, and adverse effects. In addition, subjects will be requested to separately consent to allow use of the biorepository sample for genetic testing (DNA).

F Statistical Plan

F1 *Sample Size Determination and Statistical Power*

Several design factors and research objectives were considered in selecting the target sample size for the study. First, the number of subjects was determined so there would be a sufficient number of endpoints to provide a high degree of power (greater than or equal to 90%) for testing the primary superiority hypothesis. Second, the statistical power for secondary endpoints was considered, including the composite clinical endpoints and the economic and QOL endpoints. Third, it was considered important for the sample size to be large enough to permit a prudent examination of diagnostic testing effects in selected subgroups of subjects where anatomic testing might be particularly advantageous or where the question of a benefit from CTA is particularly relevant.

Important prespecified subgroups of interest in this study include those defined by age, sex, race, comorbidity, cardiovascular risk factors, the prerandomization choice of functional test, and characteristics of the precipitating symptoms. Fourth, the sample size was selected to provide a reasonable level of confidence for detecting clinically important outcome differences between the anatomic and functional testing strategies even if current projections of event rates and the hypothesized differences in clinical outcomes between the 2 arms prove to be optimistic. A fifth consideration was the adequacy of the sample size for assessing noninferiority of anatomic testing compared with functional testing in the event that the anatomic testing strategy is not demonstrated to be statistically superior to functional testing. Finally, although the study objectives are expressed in terms of testing specific hypotheses (i.e., that the anatomic testing strategy is superior [and if not superior, then noninferior]) to functional testing, another important objective of the trial is to estimate the magnitude of the difference in outcomes to within an acceptable level of statistical precision, regardless of whether either testing strategy is proven to be superior. Thus, the precision of the estimated difference in outcomes between the 2 arms of the trial (i.e., width of the confidence interval [CI]) has been considered in addition to the statistical power for the hypothesis tests.

Based on the distribution of coronary disease expected in this patient population (approximately 15% obstructive disease [i.e., greater than or equal to 50% stenosis of the left main coronary artery or greater than or equal to 70% stenosis of 1 or more of the other major epicardial coronary arteries], 40% nonobstructive disease, and 45% normal coronary arteries) and based on national claims data or published information from other databases, the event rate at 2.5 years (the average length of follow-up in PROMISE) for the primary composite endpoint in subjects randomized to the functional testing strategy was projected to be approximately 9%.

With this event-rate projection in the functional testing arm, a key driver of the sample size is the magnitude of benefit that can reasonably be expected to be achieved with the anatomic testing strategy. This determination requires careful consideration of multiple characteristics of CTA, including its likely incremental diagnostic and prognostic accuracy and its ability to more effectively detect nonobstructive CAD. A careful assessment of the impact of the advantages of CTA with respect to these test characteristics translates to a projection (hypothesis) that CTA will reduce the primary composite endpoint by 20% (from 9% to 7.2% at 2.5 years).

Based on the event rates for each arm discussed above, sample-size requirements were formulated to provide high power for detecting the postulated 20% relative risk reduction. Recognizing, however, that the actual event rates and the outcome differences between the 2 testing strategies in PROMISE may vary somewhat from these estimates, sample-size requirements were calculated for several different combinations of event rates, effect sizes, and power levels in order to examine the sensitivity of the sample size to different event rates and outcome scenarios that might conceivably arise in this trial.

Since the primary treatment comparisons in this study will be based on time-to-event methodology using the log-rank test[82]or equivalently, the Cox proportional hazards model,[83] the approach used for calculating sample-size requirements for PROMISE was based on the sample-size methodology for the proportional hazards regression model outlined in Schoenfeld.[84]

To provide an adequate number of subjects for the trial that will be relatively robust in providing (1) excellent statistical power under various assumptions about the event rates in the functional testing arm and the magnitude of the benefit of anatomic testing compared with functional testing for reducing the primary endpoint, (2) adequate power

for selected secondary endpoints, (3) adequate power for assessing noninferiority in the event that superiority is not demonstrated, and (4) a relatively high degree of precision for estimating the true effect of an anatomic vs. functional testing strategy regardless of whether the final result has a *P* value that is statistically significant, the study will enroll 10,000 subjects (5,000 per arm). This number will provide greater than 90% power for detecting a 20% reduction in the primary clinical endpoint if the 2.5-year event rate in the functional testing arm is 8% or higher and 80% power if the event rate is as low as 6%. This number of patients will also provide adequate power for detecting a smaller (16%–17%) reduction if the event rate in the functional testing arm is 8% to 9% or higher and acceptable power for selected secondary endpoints, allowing for up to a 3% loss to follow-up. Ten thousand patients will also provide 90% power for testing noninferiority with a margin of 1.10 (expressed as a hazard ratio of CTA vs. functional testing) if the functional testing arm event rate is 9%, 86% power if the event rate is 8%, and 81% power if the event rate is 7%, assuming that anatomic testing is only better than functional testing by 10%, an assumption that was felt to be reasonable for the noninferiority assessment. That is, we will have excellent power for demonstrating that anatomic testing is not worse than functional testing by more than 10% under these various assumptions.

In summary, 10,000 patients will provide excellent and robust statistical power for assessing clinically relevant and realistic outcome differences between the 2 testing strategies being studied in this trial.

F2 *Statistical Analysis Plan*

Statistical analysis will be performed at the PROMISE SDCC at DCRI. All major treatment comparisons between the randomized groups will be performed according to the principle of "intention-to-treat"; that is, subjects will be analyzed (and endpoints attributed) according to the diagnostic testing strategy to which subjects were randomized, regardless of subsequent additional testing or postrandomization treatment and medical care. Statistical comparisons will be performed using 2-sided significance tests. Additional perspective regarding the interpretation of the data will be provided through extensive use of CIs[85] and graphical displays.

2.a **Analysis for the Primary Endpoint**

The statistical comparison of the 2 randomized arms (anatomic vs. functional diagnostic testing) with respect to the primary composite endpoint (death, MI, major peri-procedural complications, or hospitalization for unstable angina) will be a “time-to event” analysis and therefore will be based on the time from randomization to the first occurrence of any of the components of the primary composite endpoint. The Cox proportional hazards model will be the primary analytic tool for assessing outcome differences between the 2 randomized arms.[83] To appropriately account for heterogeneity among the subjects, the overall comparison will be adjusted for a selected set of prognostically important baseline covariates that will be carefully defined and prespecified in the statistical analysis plan. The level of significance for the assessment of the primary endpoint will be $\alpha=0.05$.

In addition to the statistical hypothesis testing, Kaplan-Meier “survival” (or event-free) estimates[86] will be calculated for each randomized arm as a function of follow-up time to display the event rates graphically. A hazard ratio and 95% CI for descriptively

summarizing the difference in outcome between the 2 study arms will be computed using the Cox model.[83]

If the data provide evidence of an overall difference in outcome between the randomized arms, an assessment will be made of whether the effect is similar for all patients or whether it varies according to specific patient characteristics. In particular, this analysis will focus on whether the relative benefit differs according to subject age, sex, race, comorbidity, selected risk factors, characteristics of the precipitating symptoms, and the prerandomization specification of the functional test that would be used if the subject was randomized to the functional testing arm. This latter factor is built into the randomization scheme. For subjects for whom the prerandomization choice for functional testing (if the subject was assigned to the functional testing arm) was stress nuclear imaging (for example), the outcomes of these subjects in the CTA arm will be compared to the outcomes of corresponding subjects randomized to functional testing. In this way, CTA can be compared with stress nuclear imaging, as well as with each of the other functional testing modalities with the benefits of randomization in each comparison maintained by virtue of the stratified randomization scheme. These analyses will utilize the Cox model by testing for interactions between the randomized testing strategy and these specific baseline variables. In addition to the formal assessment of testing strategy by covariate interactions, effects of the diagnostic testing strategy characterized by a hazard ratio and 95% CI will be calculated and displayed for prospectively defined subgroups of subjects defined by the variables listed above. These descriptive hazard ratios will be carefully interpreted in conjunction with the formal interaction tests.

If the data do not provide statistical evidence that the CTA testing strategy is superior to functional testing with respect to the primary endpoint, a test for noninferiority of the anatomic testing strategy will be performed. This assessment will be based on a noninferiority margin of 1.10 (expressed as a hazard ratio for CTA vs. functional testing). The noninferiority assessment will be performed by comparing the upper limit of the 95% CI for the hazard ratio with the noninferiority margin. If the upper limit of the CI falls below 1.10, noninferiority will have been demonstrated. We emphasize that the superiority hypothesis will be assessed first, and if significant, the noninferiority assessment will not be performed. Only if superiority is not demonstrated will the noninferiority analysis then be performed.

2.b Analysis of Secondary Endpoints

Secondary endpoints that will be evaluated include (1) a composite endpoint consisting of death, MI, or hospitalization for unstable angina; (2) death or MI; (3) major peri-procedural complications (stroke, major bleeding, renal failure, anaphylaxis); (4) a composite of the primary endpoint or invasive catheterization without obstructive CAD, (5) invasive catheterization without obstructive CAD, (6) resource-use patterns, medical care costs and incremental cost effectiveness; and (7) QOL. In addition, major adverse cardiac events (events other than the endpoints listed above) will be monitored and reported.

The analysis of secondary endpoints 1 through 4 will be similar to that outlined for the superiority assessment of the primary endpoint, using time from randomization until the first occurrence of any component of the specific secondary endpoint (or censoring) as the response variable, and assessing group differences using the Cox proportional hazards model. The effect of the diagnostic testing strategy on these secondary clinical endpoints will be descriptively summarized using hazard ratios (with associated CIs)

computed from the Cox model. Kaplan-Meier curves will be computed to graphically display the cumulative event rates of the 2 randomized arms as a function of time from randomization. We note that the analysis of secondary endpoint (3) above (major peri-procedural complications) will have to be interpreted cautiously, particularly if there should be a higher death rate in one arm compared with the other. To deal with this complexity and clarify and enhance the interpretation of this comparison, the analysis of this endpoint will be supplemented with further analyses by considering major peri-procedural complications and death as a combined endpoint.

Plans for the analysis of the QOL and economic endpoints are addressed below in Sections F2.g and F2.h.

2.c **Analysis of Diagnostic Testing Core Data**

The DTCC will implement robust QA programs to ensure uniformity and high-quality testing in support of the primary aims of PROMISE.

Two important components of information from the diagnostic test QA activity will be analyzed by the SDCC. The DTCC will review the first 8000 subjects' initial diagnostic test for technical quality, and each test will be assigned a quality assessment using an ordinal categorical scale (for example, excellent, good, fair, poor, uninterpretable). Using simple frequency counts, the SDCC will tabulate a description of the distribution of this scale on an ongoing basis by type of test and by testing site in order to provide feedback to the sites on their performance, to inform the study leadership and the DSMB as to the quality of the diagnostic testing, and to flag potential problem areas, whether by testing site or across a given testing modality, for remedial attention. This quality measure will be compared among the different testing modalities using rank-based tests (e.g., Kruskal-Wallis nonparametric analysis of variance) and ordinal logistic regression to characterize, describe, and assess any differences in overall quality among the various testing modalities. In particular, the technical quality of the anatomic (CTA) tests will be compared with the quality of the functional tests. This comparison may be helpful in interpreting the comparisons of the 2 randomized arms with respect to the clinical outcomes.

The second important component of information from the diagnostic test QA activity is the quality of test interpretations. Approximately 10% of tests will be overread by the DTCC using a categorical level of coronary disease risk. The site interpretation of level of risk will be compared with the level of risk using the same scale assessed by the core lab's overread of the test, and overall summary statistics of the agreement between the site and core lab assessments will be computed. Raw proportions of agreement (perfect agreement, and agreement differing by less than or equal to 1 category of risk) will be tabulated, and Kappa statistics will be used to characterize the level of agreement. The Kappa statistics are "chance corrected" (i.e., adjusted for agreement due to chance), and both unweighted and weighted Kappa statistics will be used in these analyses since more substantial disagreements in the assessment of risk for a given subject would be more serious than small disagreements. These agreement statistics will then be compared across the different testing modalities to determine whether the level of agreement between the site readings and the core lab readings varies with the type of test.

Another important component of information to characterize data quality for the secondary endpoints that involve non-obstructive CAD will be the review of selected

coronary angiograms to determine the degree to which site-reported assessments of non-obstructive coronary disease are concordant with carefully-performed core lab QCA. The same type of agreement statistics as described above, including raw proportions of agreement and chance-corrected Kappa statistics, will be used to describe and characterize the accuracy of the site-reported assessments of non-obstructive CAD.

Analysis of Diagnostic Accuracy

Although PROMISE is designed with the primary objective of evaluating initial anatomic vs. functional testing strategies with respect to clinical outcomes and thus represents a different paradigm than the traditional design to assess diagnostic accuracy, performance of supplementary analyses of diagnostic accuracy are prespecified. This cannot be done in a conventional manner as not all subjects will undergo invasive angiography (the “gold standard”), and those who do will not undergo angiography by random selection. Instead, the decision to verify disease will be based on test results and other characteristics of the subject. This nonrandom selection process will likely result in a strong verification bias,[87] often characterized by higher than true sensitivity and lower than true specificity results. (Of note, the trial cannot require angiography in a subset of subjects for purposes of determining testing accuracy, as this would provide additional information to these subjects’ physicians and care givers above and beyond that provided by the randomized testing strategy, and therefore invalidate the trial results.) Correction of the verification bias is possible if the process leading to verification with angiography is known. However, this is rarely achievable, but the process can be modeled under the missing at random (MAR) assumption,[88] namely that disease status affects referral to angiography only through measured covariates and not the disease status itself. In view of the inherent limitations, the following steps will be taken with respect to evaluating diagnostic accuracy.

Accuracy rates will be assessed using conventional measures (sensitivity, specificity, and receiver operating characteristic [ROC] curves) in subjects undergoing cardiac catheterization, bringing to bear where applicable the latest statistical methods for dealing with verification bias. The probability of verification will be modeled with a logistic regression model using covariates that are predictors of referral to angiography. Corrected values of sensitivity, specificity, and the ROC curve will be estimated.[88,89] Assessment of the impact of potential departures from the MAR assumption on sensitivity and specificity will utilize the “test ignorance region” approach.[88] Alternatively, we will use the clinical outcome (rather than coronary angiography) as the “gold standard,” such that measures of diagnostic accuracy, including time-dependent ROC curves, will be computed to describe and statistically compare the 2 arms of the trial with respect to these measures. A subgroup analysis of diagnostic accuracy will be performed comparing results at sites with high volume and extensive experience or expertise in diagnostic testing with results in less-experienced or lower-volume sites.

Imaging prognostic performance will also be evaluated by comparing the association of test results with subsequent clinical events, including the primary endpoint of the trial. Finally, as described in Section F2.c above, concordance between the site interpretations of studies and core lab interpretations will be assessed.

We emphasize that all of these analyses of diagnostic accuracy will be strictly supplementary to the analysis of the primary and secondary clinical endpoints outlined in Sections F2.a and F2.b above.

2.d Analysis of Safety Events, Morbidity, Radiation Dose, and Incidental Findings

The frequency with which major safety cardiac events occur (events other than the primary and secondary endpoints) will be carefully tabulated and descriptively summarized. Statistical comparisons of the randomized arms with respect to these events will use chi-square or other appropriate 2-sample methods, depending on the nature of the event, interpreting such comparisons in the context of differences between the 2 randomized arms in the primary and major secondary clinical endpoints and bringing to bear clinical judgment as to the relative seriousness of these events.

Of particular interest in this trial is the amount of radiation exposure to which subjects are subjected in each of the randomized arms of the trial. Radiation exposure for the various tests will be collected as elements of the electronic CRF (eCRF) and through the documentation of radiation exposure submitted to the DTCC. All cardiac diagnostic testing involving radiation exposure (e.g., CTA, stress nuclear, cardiac catheterization) performed during the entire follow-up period will be captured so that cumulative radiation exposure for each patient can be calculated. The distribution of radiation exposure in each arm will be summarized (using medians and percentiles) and compared between the randomized arms and different testing modalities using the Wilcoxon rank-sum test.

Incidental findings (e.g., such things as lung nodules) that may be discovered with the anatomic (CTA) testing strategy in contrast to the functional testing modalities will be captured as part of the data collection and tabulated descriptively.

2.e Assessment of Prognostic Factors

With the large database of information that will be collected on the 10,000 subjects enrolled in this study, extensive regression modeling analyses will be performed, using primarily the Cox regression model, to identify and assess the factors (predictors) that are associated with the clinical outcomes of these subjects. These analyses will comprehensively evaluate the strength and shape of the relationships of numerous clinical factors with the clinical outcomes. While these analyses are more exploratory than the rigorous prespecified primary and secondary comparisons of the randomized arms of the trial, they will nonetheless be helpful in elucidating relationships and identifying the key factors that impact patient outcomes and any observed differences in outcomes between the diagnostic testing arms.

2.f Quality-of-life Analyses

For each of the QOL measures, data analysis will proceed in several stages. We will start by providing simple descriptive and comparative analyses by intention-to-treat. A nonparametric bootstrap will be used to estimate treatment differences with 95% CIs and *P* values. Because there is currently no consensus in the statistical literature about the best way to deal with the multiple comparisons problem arising from testing each individual scale at each time point separately, we propose 2 complementary approaches. First, we will prespecify the angina frequency and disease perception/QOL scales from the SAQ as the CAD-specific measures of primary interest, since these measures most directly quantify the therapeutic goal of coronary diagnosis and therapy: to minimize symptoms and optimize patients' QOL. We will also specify cardiac functional status measured with the DASI as a primary outcome measure of interest. Other disease-specific and generic QOL measures will be assigned to a secondary

(descriptive) status in our analyses. Second, we will fit a mixed effect longitudinal proportional odds model[90] that makes use of all available QOL data at each study assessment point to model the time profile (fixed effect) using a restricted cubic spline function. Using the fitted model, we can estimate the overall difference in the QOL measures as well as test the global hypothesis of no difference over time. We can also estimate the difference in the areas under the 2 QOL treatment curves (and test the hypothesis of no difference on average). In addition, we can estimate differences in QOL at the end of the study or at intermediate points such as at 1 year. Statistical power estimates for this part of our analysis show that we should have in excess of 90% power to detect ¼ standard deviation differences in the 3 principal QOL endpoints.

2.g **Economic Analyses**

The health economic analyses for PROMISE will consist of 2 major parts, namely an empirical intention-to-treat cost comparison and a cost-effectiveness analysis. Primary statistical comparisons between the 2 treatment arms of empirical costs will be performed by intention-to-treat analysis. Confidence limits around the observed cost differences will be constructed using bootstrap methods.

The cost-effectiveness analyses will estimate the incremental cost required to add an extra life year with the investigational anatomic arm relative to the control functional testing arm. In secondary analyses, we will incorporate utility weights to estimate the incremental cost per quality-adjusted life year gained with the CTA anatomic strategy, relative to the functional testing strategy. These analyses will be conducted from a societal perspective and will use a lifetime time horizon so that the estimated incremental cost-effectiveness and cost-utility ratios can be compared with societal benchmarks. We will also calculate within-trial cost-effectiveness/cost-utility ratios, although these ratios are limited in their value due to their failure to account for long-term benefits and costs and the absence of comparative benchmarks. Costs will be adjusted for inflation, and both costs and life expectancy will be discounted to present value at a 3% annual discount rate. Adjustments for censored data due to staggered entry will be made following the approach of Bang and Tsiatis.[91] Extensive sensitivity analyses will be performed.

F3 *Interim Analyses*

For safety and ethical reasons, interim examinations of key safety and endpoint data will be performed at regular intervals during the course of the trial. The primary objective of these analyses will be to evaluate the accumulating data for an unacceptably high frequency of negative clinical outcomes in either of the 2 randomized arms. In addition, the interim monitoring will also involve a review of subject recruitment, compliance with the study protocol, status of data collection, and other factors that reflect the overall progress and integrity of the study. The results of the interim analyses and status reports will be carefully and confidentially reviewed by the NHLBI-appointed DSMB. The DSMB will meet at approximately 6-month intervals to review the accumulating data.

To properly account for the repeated interim testing in PROMISE, a group sequential method similar to that proposed by O'Brien and Fleming[92] will be used as a guide for interpreting the interim analyses. This procedure requires large critical values early in the study, but relaxes (i.e., decreases) the critical value as the trial progresses. Because of the conservatism early in the trial, the critical value at the final analysis is near the

"nominal" critical value. The actual method for this interim monitoring that will be employed in PROMISE is the “spending function” approach to group sequential testing developed by Lan and DeMets.[93] The Lan-DeMets approach only requires specification of the rate at which the Type I error (which in this trial will be α=0.05 for the primary endpoint) will be "spent." This procedure allows "spending" a portion of α at each interim analysis in such a way that at the end of the study, the total Type I error does not exceed 0.05. One such spending function generates boundaries that are nearly identical to the O'Brien-Fleming[92] boundaries. It is this approach that will be used in PROMISE, namely 2-sided, symmetric O'Brien-Fleming type boundaries generated using the flexible Lan-DeMets[93] approach to group sequential testing. Since the number of looks and the increments between looks need not be predetermined, it allows considerable flexibility in the monitoring process for accommodating additional comparative examinations of the data in response to concerns of the DSMB that may arise during the course of the trial.

The analytic approach that will be used at the interim analyses for assessing outcome differences between the randomized arms will be the time-to-event analysis methods described previously, except that interpretation of statistical significance will be guided using the group sequential monitoring boundaries outlined above. At each interim analysis, the monitoring boundaries will be calculated using the fraction of the total number of primary events expected by the end of the trial.

Judgment concerning the continuation or termination of the study will involve not only the degree of statistical significance observed at the interim analyses, but also careful consideration of many other factors reflecting the progress and integrity of the trial, including how well event rates in the functional testing arm are tracking with the rates considered in the power calculations. It should be emphasized that regardless of whether CTA demonstrates a statistically significant benefit compared with functional testing, the results of the study will be important to future clinical practice. Indeed, a non-statistically significant effect for the primary endpoint is not necessarily a negative result in this study. The range of experience and expertise of the DSMB will enable them to put all important considerations into proper perspective, including the precision of estimates of clinical outcome differences between the testing strategies, and make well-reasoned recommendations to the NHLBI regarding continuation of the study.

G Data Handling and Record Keeping

Study Data Collection—All Subjects

The full study dataset will be collected for subjects who enter the randomized phase of the study. The primary data collection system for PROMISE will use the electronic data capture system InForm™.

Data Management and Quality

Any out-of-range values and missing or inconsistent key variables are flagged and addressed/answered at the site in real time during the data entry process. When a query is generated on a particular variable, a flag is set in a field in the database, enabling the system to track the queries and produce reports of outstanding queries. Queries can also be generated from manual review of the data forms. These queries will be entered into the database and tracked in the same manner as the computer-generated queries. At regular intervals, all data will be transferred from InForm™ to SAS for statistical

summarization, data description, and data analysis. Further cross-checking of the data will be performed in SAS and discrepant observations flagged and appropriately resolved through a data-query system.

The SDCC will perform internal database quality-control checks and data audits throughout the course of the trial.

G1 *Confidentiality and Security*

All study data will be stored in locked, secure locations. Computerized data are accessible only by password, and a centralized monitoring system records and reports all access to data. The DCRI computer network is protected by a firewall. Electronic CRFs will be identified by study number only, to ensure subject anonymity. No subject identifiers will be used in the presentation of data. Study records that might identify subjects will be kept confidential as required by law. Except when required by law, subjects will not be identified by name, social security number, address, telephone number, or any other direct personal identifier in study records. This information will be retained by each individual center and will not be disclosed to the coordinating center except as needed for DCRI centralized clinical, QOL, and economic follow-up of the subjects. Subjects will be informed that the study physician and his or her study team will report the results of study-related tests to the Coordinating Center and to the National Institutes of Health (NIH). Subjects will be informed that their records may be reviewed in order to meet federal or state regulations. Reviewers may include the Food and Drug Administration, IRBs/IECs, or the NIH. Subjects will be informed that if their research record is reviewed, their entire medical record may also need to be reviewed. If an adverse event occurs, management of the event and subsequent care will be according to appropriate care practices at that site and will be implemented under the direction of the treating physician. All of the tests used in this study currently represent a reasonable standard of care for the subject population as demonstrated by ACC/AHA practice standards.

G2 *Training*

All investigator staff authorized to enter PROMISE Study data will receive training on the InForm™ system.

G3 *Electronic Case Report Form*

This study will use Web-based e-CRFs developed through a validated, Electronic Record, Electronic Signatures-compliant platform (21 CFR Part 11). Data will be entered into the InForm™ eCRF by authorized Investigator personnel, ACRIN, and the diagnostic testing core labs.

G4 *Records Retention*

Study records will be maintained by the site investigators for a period of 6 years following the expiration of the grant or length of time as required by local regulations.

H Study Monitoring, Auditing, and Inspecting

H1 *Study Monitoring Plan*

See E3.a

H2 *Auditing and Inspecting*

National Heart Lung and Blood Institute personnel or their designees may perform an audit at any time during or after completion of this study. All original study-related documentation will be made available to the designated auditor as required. A representative of the NIH or other government agency may choose to inspect a study center at any time before, during, or after completion of the clinical study. All pertinent original study data will be made available to responsible regulatory authorities for verification, audit, or inspection purposes.

I Study Administration

I1 *Organization and Participating Centers*

The PROMISE trial will be conducted under the following principal investigator (PI) leadership: Dr. Pamela Douglas as the overall and CCC PI, Dr. Kerry Lee as the SDCC PI, Dr. Daniel Mark as the EQOL CC PI, and Dr. Udo Hoffmann at Massachusetts General Hospital as the DTCC PI. The organization of the **PROMISE** trial and interrelationships between the NHLBI, the CCC, the SDCC, the various committees, and the clinical sites are outlined in the figure below.

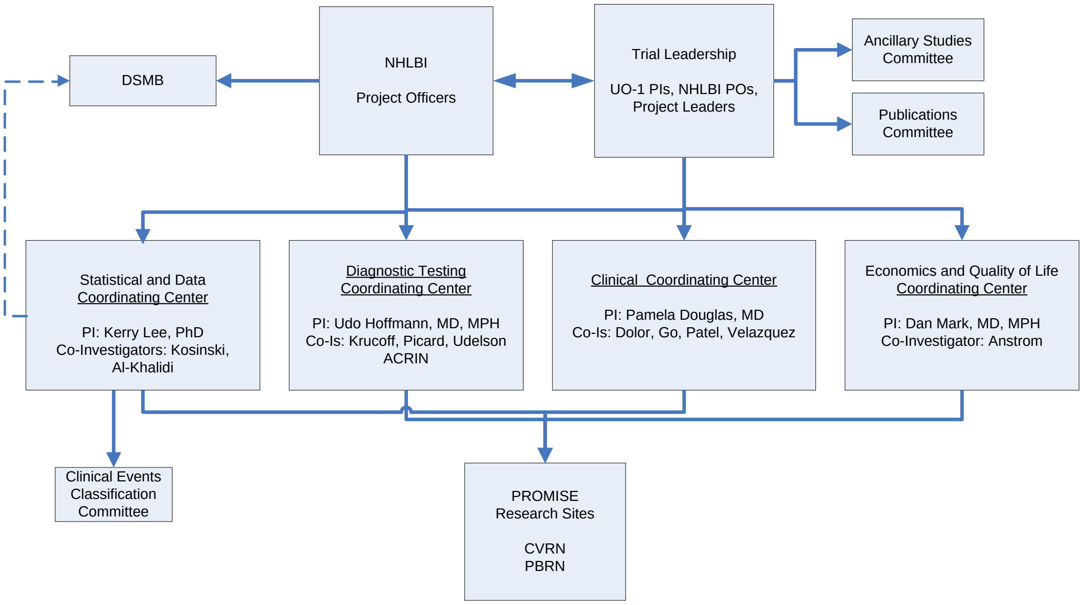

I2 *Funding Source*

The PROMISE trial is funded by the National Heart Lung and Blood Institute, National Institutes of Health.

I3 *Study Timetable*

The PROMISE timeline includes a 9-month start-up period, followed by approximately 36 months of enrollment, approximately 12 months of follow-up after the last subject is enrolled, and 3 months of closeout and data analysis.

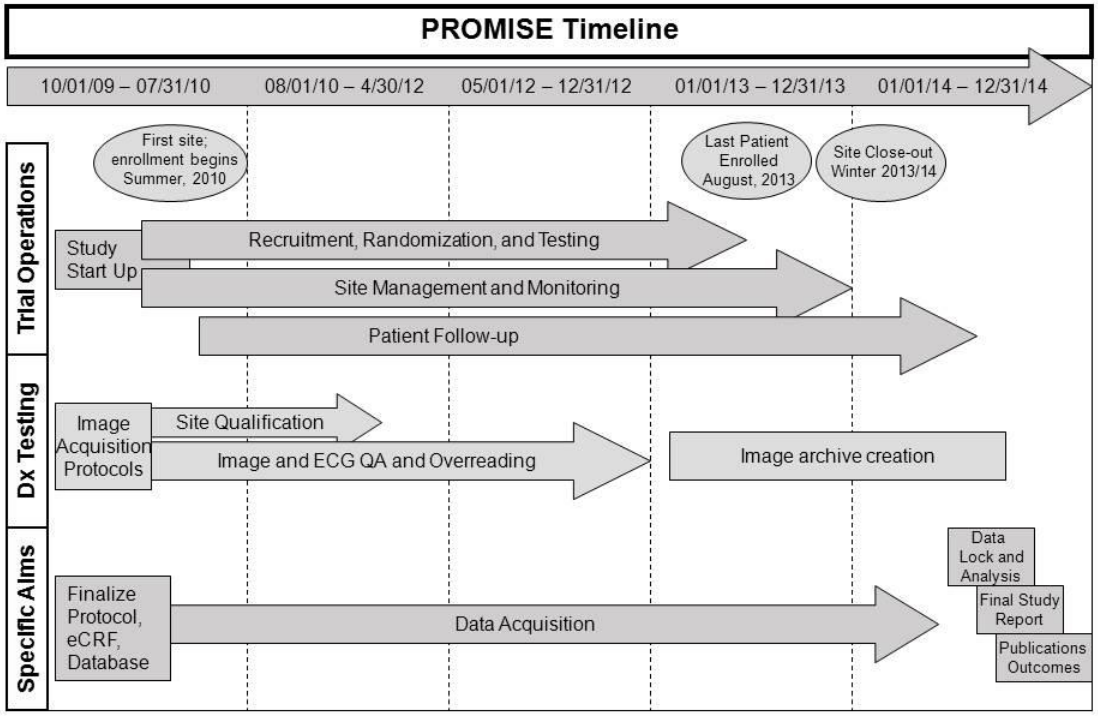
PROMISE Timeline
10/01/09 – 07/31/10
08/01/10 – 4/30/12
05/01/12 – 12/31/12
01/01/13 – 12/31/13
01/01/14 – 12/31/14
Trial Operations
First site; enrollment begins Summer, 2010
Last Patient Enrolled August, 2013
Site Close-out Winter 2013/14
Study Start Up
Recruitment, Randomization, and Testing
Site Management and Monitoring
Patient Follow-up
Dx Testing
Image Acquisition Protocols
Site Qualification
Image and ECG QA and Overreading
Image archive creation
Specific Aims
Finalize Protocol, eCRF, Database
Data Acquisition
Data Lock and Analysis
Final Study Report
Publications Outcomes

J References

1. Rosamond W, Flegal K, Furie K, et al. Heart disease and stroke statistics--2008 update: a report from the American Heart Association Statistics Committee and Stroke Statistics Subcommittee. *Circulation*. 2008;117(4):e25-146.

2. Selker HP, Zalenski RJ, Antman EM, et al. An evaluation of technologies for identifying acute cardiac ischemia in the emergency department: a report from a National Heart Attack Alert Program Working Group. *Ann Emerg Med*. 1997;29(1):13-87.

3. Hemingway H, McCallum A, Shipley M, et al. Incidence and prognostic implications of stable angina pectoris among women and men. *JAMA*. 2006;295(12):1404-1411.

4. Daly CA, De Stavola B, Sendon JLL, et al. Predicting prognosis in stable angina--results from the Euro heart survey of stable angina: prospective observational study. *BMJ*. 2006;332(7536):262-267.

5. Sekhri N, Feder GS, Junghans C, et al. Incremental prognostic value of the exercise electrocardiogram in the initial assessment of patients with suspected angina: cohort study. *BMJ*. 2008;337:a2240.

6. Clayton TC, Lubsen J, Pocock SJ, et al. Risk score for predicting death, myocardial infarction, and stroke in patients with stable angina, based on a large randomised trial cohort of patients. *BMJ*. 2005;331(7521):869.

7. Timmis AD, Feder GS, Sekhri N, Hemingway H. *Are rapid access chest pain clinics effective and fair? Characteristics and outcomes of patients from six centres*. National Co-ordinating Centre for NHS Service Delivery and Organisation R & D; 2005:228. Available at: http://www.sdo.nihr.ac.uk/files/project/32-final-report.pdf [Accessed February 10, 2011].

8. Abstract 800: Downstream Care and Outcomes Following Outpatient Stress Testing in the United States: How Low is Too Low Risk? 2009. Available at: http://circ.ahajournals.org/cgi/content/meeting_abstract/120/18_MeetingAbstracts/S393?maxtoshow=&hits=10&RESULTFORMAT=&fulltext=mudrick&searchid=1&FIRSTINDEX=0&volume=120&issue=18+Supplement&resourcetype=HWCIT [Accessed February 10, 2011].

9. Sekhri N, Feder GS, Junghans C, Hemingway H, Timmis AD. How effective are rapid access chest pain clinics? Prognosis of incident angina and non-cardiac chest pain in 8762 consecutive patients. *Heart*. 2007;93(4):458-463.

10. Daly CA, Clemens F, Sendon JLL, et al. The clinical characteristics and investigations planned in patients with stable angina presenting to cardiologists in Europe: from the Euro Heart Survey of Stable Angina. *Eur. Heart J*. 2005;26(10):996-1010.

11. Cheitlin MD, Armstrong WF, Aurigemma GP, et al. ACC/AHA/ASE 2003 guideline update for the clinical application of echocardiography--summary article: a report of the American College of Cardiology/American Heart Association Task Force on Practice Guidelines (ACC/AHA/ASE Committee to Update the 1997 Guidelines for the Clinical Application of Echocardiography). *J. Am. Coll. Cardiol*. 2003;42(5):954-970.

12. Gibbons RJ, Balady GJ, Bricker JT, et al. ACC/AHA 2002 guideline update for exercise testing: summary article. A report of the American College of Cardiology/American Heart Association Task Force on Practice Guidelines (Committee to Update the 1997 Exercise Testing Guidelines). *J. Am. Coll. Cardiol*. 2002;40(8):1531-1540.

13. Klocke FJ, Baird MG, Lorell BH, et al. ACC/AHA/ASNC guidelines for the clinical use of cardiac radionuclide imaging--executive summary: a report of the American College of Cardiology/American Heart Association Task Force on Practice Guidelines (ACC/AHA/ASNC Committee to Revise the 1995 Guidelines for the Clinical Use of Cardiac Radionuclide Imaging). *J. Am. Coll. Cardiol*. 2003;42(7):1318-1333.

14. Pope JH, Aufderheide TP, Ruthazer R, et al. Missed diagnoses of acute cardiac ischemia in the emergency department. *N. Engl. J. Med*. 2000;342(16):1163-1170.

15. Patel MR, Peterson ED, Dai D, et al. Low diagnostic yield of elective coronary angiography. *N. Engl. J. Med*. 2010;362(10):886-895.

16. Budoff MJ, Dowe D, Jollis JG, et al. Diagnostic performance of 64-multidetector row coronary computed tomographic angiography for evaluation of coronary artery stenosis in individuals without known coronary artery disease: results from the prospective multicenter ACCURACY (Assessment by Coronary Computed Tomographic Angiography of Individuals Undergoing Invasive Coronary Angiography) trial. *J. Am. Coll. Cardiol*. 2008;52(21):1724-1732.

17. Miller JM, Rochitte CE, Dewey M, et al. Diagnostic performance of coronary angiography by 64-row CT. *N. Engl. J. Med*. 2008;359(22):2324-2336.

18. Stein PD, Yaekoub AY, Matta F, Sostman HD. 64-slice CT for diagnosis of coronary artery disease: a systematic review. *Am. J. Med*. 2008;121(8):715-725.

19. Mowatt G, Cook JA, Hillis GS, et al. 64-Slice computed tomography angiography in the diagnosis and assessment of coronary artery disease: systematic review and meta-analysis. *Heart*. 2008;94(11):1386-1393.

20. LaRosa JC, He J, Vupputuri S. Effect of statins on risk of coronary disease: a meta-analysis of randomized controlled trials. *JAMA*. 1999;282(24):2340-2346.

21. Fleischmann KE, Hunink MG, Kuntz KM, Douglas PS. Exercise echocardiography or exercise SPECT imaging? A meta-analysis of diagnostic test performance. *JAMA*. 1998;280(10):913-920.

22. Schuijf JD, Poldermans D, Shaw LJ, et al. Diagnostic and prognostic value of non-invasive imaging in known or suspected coronary artery disease. *Eur. J. Nucl. Med. Mol. Imaging*. 2006;33(1):93-104.

23. Garber AM, Solomon NA. Cost-effectiveness of alternative test strategies for the diagnosis of coronary artery disease. *Ann. Intern. Med*. 1999;130(9):719-728.

24. CORONARY COMPUTED TOMOGRAPHIC ANGIOGRAPHY FOR DETECTION OF CORONARY ARTERY DISEASE. 2008. Available at: http://www.icer-review.org/index.php/CCTA/View-category.html [Accessed February 10, 2011].

25. Schuetz GM, Zacharopoulou NM, Schlattmann P, Dewey M. Meta-analysis: noninvasive coronary angiography using computed tomography versus magnetic resonance imaging. *Ann. Intern. Med*. 2010;152(3):167-177.

26. Marcassa C, Bax JJ, Bengel F, et al. Clinical value, cost-effectiveness, and safety of myocardial perfusion scintigraphy: a position statement. *Eur. Heart J*. 2008;29(4):557-563.

27. Mahenthiran J, Bangalore S, Yao S, Chaudhry FA. Comparison of prognostic value of stress echocardiography versus stress electrocardiography in patients with suspected coronary artery disease. *Am. J. Cardiol*. 2005;96(5):628-634.

28. Yao SS, Qureshi E, Sherrid MV, Chaudhry FA. Practical applications in stress echocardiography: risk stratification and prognosis in patients with known or suspected ischemic heart disease. *J. Am. Coll. Cardiol*. 2003;42(6):1084-1090.

29. Hachamovitch R, Hayes S, Friedman JD, et al. Determinants of risk and its temporal variation in patients with normal stress myocardial perfusion scans: what is the warranty period of a normal scan? *J. Am. Coll. Cardiol*. 2003;41(8):1329-1340.

30. Metz LD, Beattie M, Hom R, et al. The prognostic value of normal exercise myocardial perfusion imaging and exercise echocardiography: a meta-analysis. *J. Am. Coll. Cardiol*. 2007;49(2):227-237.

31. Bamberg F, Sommer W, Hoffmann V. Meta-analysis and systematic review of the long-term predictive value of assessment of coronary atherosclerosis by contrast-enhanced coronary computed tomography angiography. *Journal of the American College of Cardiology*. In press.

32. Min JK, Shaw LJ, Devereux RB, et al. Prognostic value of multidetector coronary computed tomographic angiography for prediction of all-cause mortality. *J. Am. Coll. Cardiol*. 2007;50(12):1161-1170.

33. Ostrom MP, Gopal A, Ahmadi N, et al. Mortality incidence and the severity of coronary atherosclerosis assessed by computed tomography angiography. *J. Am. Coll. Cardiol*. 2008;52(16):1335-1343.

34. Hachamovitch R, Berman DS, Kiat H, et al. Exercise myocardial perfusion SPECT in patients without known coronary artery disease: incremental prognostic value and use in risk stratification. *Circulation*. 1996;93(5):905-914.

35. Berman DS, Kang X, Hayes SW, et al. Adenosine myocardial perfusion single-photon emission computed tomography in women compared with men. Impact of diabetes mellitus on incremental prognostic value and effect on patient management. *J. Am. Coll. Cardiol*. 2003;41(7):1125-1133.

36. Galassi AR, Azzarelli S, Tomaselli A, et al. Incremental prognostic value of technetium-99m-tetrofosmin exercise myocardial perfusion imaging for predicting outcomes in patients with suspected or known coronary artery disease. *Am. J. Cardiol*. 2001;88(2):101-106.

37. Hachamovitch R, Berman DS, Shaw LJ, et al. Incremental prognostic value of myocardial perfusion single photon emission computed tomography for the prediction of cardiac death: differential stratification for risk of cardiac death and myocardial infarction. *Circulation*. 1998;97(6):535-543.

38. Picano E, Severi S, Michelassi C, et al. Prognostic importance of dipyridamole-echocardiography test in coronary artery disease. *Circulation*. 1989;80(3):450-457.

39. Poldermans D, Fioretti PM, Boersma E, et al. Dobutamine-atropine stress echocardiography and clinical data for predicting late cardiac events in patients with suspected coronary artery disease. *Am. J. Med*. 1994;97(2):119-125.

40. Cortigiani L, Dodi C, Paolini EA, et al. Prognostic value of pharmacological stress echocardiography in women with chest pain and unknown coronary artery disease. *J. Am. Coll. Cardiol*. 1998;32(7):1975-1981.

41. Weiner DA, Ryan TJ, McCabe CH, et al. Prognostic importance of a clinical profile and exercise test in medically treated patients with coronary artery disease. *J. Am. Coll. Cardiol*. 1984;3(3):772-779.

42. Mark DB, Hlatky MA, Harrell FE, et al. Exercise treadmill score for predicting prognosis in coronary artery disease. *Ann. Intern. Med*. 1987;106(6):793-800.

43. Mark DB, Shaw L, Harrell FE, et al. Prognostic value of a treadmill exercise score in outpatients with suspected coronary artery disease. *N. Engl. J. Med*. 1991;325(12):849-853.

44. Min JK, Shaw LJ, Berman DS, Gilmore A, Kang N. Costs and clinical outcomes in individuals without known coronary artery disease undergoing coronary computed tomographic angiography from an analysis of Medicare category III transaction codes. *Am. J. Cardiol*. 2008;102(6):672-678.

45. Hoffmann U, Moselewski F, Nieman K, et al. Noninvasive assessment of plaque morphology and composition in culprit and stable lesions in acute coronary syndrome and stable lesions in stable angina by multidetector computed tomography. *J. Am. Coll. Cardiol*. 2006;47(8):1655-1662.

46. Min JK, Kang N, Shaw LJ, et al. Costs and clinical outcomes after coronary multidetector CT angiography in patients without known coronary artery disease: comparison to myocardial perfusion SPECT. *Radiology*. 2008;249(1):62-70.

47. Abstract 981: High-Sensitivity Troponin Reliably Excludes Acute Coronary Syndrome in Patients with Acute Chest Pain: Results from the Rule Out Myocardial Infarction by Computed Tomography (ROMICAT) Study. 2008. Available at: http://circ.ahajournals.org/cgi/content/meeting_abstract/118/18_MeetingAbstracts/S_637 [Accessed February 10, 2011].

48. Shah BR, Patel MR, Peterson ED, Douglas PS. Defining optimal research study design for cardiovascular imaging using computed tomography angiography as a model. *Am. J. Cardiol*. 2008;102(7):943-948.

49. Hachamovitch R, Di Carli MF. Methods and limitations of assessing new noninvasive tests: part I: Anatomy-based validation of noninvasive testing. *Circulation*. 2008;117(20):2684-2690.

50. Bonow RO. Sixth Annual Mario S. Verani, MD Memorial Lecture: Cardiovascular imaging--added value or added cost? *J Nucl Cardiol*. 2008;15(2):170-177.

51. Douglas PS. Improving imaging: our professional imperative. *J. Am. Coll. Cardiol*. 2006;48(10):2152-2155.

52. Douglas PS, Taylor A, Bild D, et al. Outcomes research in cardiovascular imaging: report of a workshop sponsored by the National Heart, Lung, and Blood Institute. *Circ Cardiovasc Imaging*. 2009;2(4):339-348.

53. Douglas PS, Khandheria B, Stainback RF, et al. ACCF/ASE/ACEP/AHA/ASNC/SCAI/SCCT/SCMR 2008 appropriateness criteria for stress echocardiography: a report of the American College of Cardiology Foundation Appropriateness Criteria Task Force, American Society of Echocardiography, American College of Emergency Physicians, American Heart Association, American Society of Nuclear Cardiology, Society for Cardiovascular Angiography and Interventions, Society of Cardiovascular Computed Tomography, and Society for Cardiovascular Magnetic Resonance endorsed by the Heart Rhythm Society and the Society of Critical Care Medicine. *J. Am. Coll. Cardiol*. 2008;51(11):1127-1147.

54. Brindis RG, Douglas PS, Hendel RC, et al. ACCF/ASNC appropriateness criteria for single-photon emission computed tomography myocardial perfusion imaging (SPECT MPI): a report of the American College of Cardiology Foundation Quality Strategic Directions Committee Appropriateness Criteria Working Group and the American Society of Nuclear Cardiology endorsed by the American Heart Association. *J. Am. Coll. Cardiol*. 2005;46(8):1587-1605.

55. Fryback DG, Thornbury JR. The efficacy of diagnostic imaging. *Med Decis Making*. 1991;11(2):88-94.

56. Detrano R, Guerci AD, Carr JJ, et al. Coronary calcium as a predictor of coronary events in four racial or ethnic groups. *N. Engl. J. Med*. 2008;358(13):1336-1345.

57. Bairey Merz CN, Shaw LJ, Reis SE, et al. Insights from the NHLBI-Sponsored Women's Ischemia Syndrome Evaluation (WISE) Study: Part II: gender differences in presentation, diagnosis, and outcome with regard to gender-based pathophysiology of atherosclerosis and macrovascular and microvascular coronary disease. *J. Am. Coll. Cardiol*. 2006;47(3 Suppl):S21-29.

58. Falk E, Shah PK, Fuster V. Coronary plaque disruption. *Circulation*. 1995;92(3):657-671.

59. Kern MJ, Meier B. Evaluation of the culprit plaque and the physiological significance of coronary atherosclerotic narrowings. *Circulation*. 2001;103(25):3142-3149.

60. Javitz HS, Ward MM, Watson JB, Jaana M. Cost of illness of chronic angina. *Am J Manag Care*. 2004;10(11 Suppl):S358-369.

61. Douglas P, Iskandrian AE, Krumholz HM, et al. Achieving quality in cardiovascular imaging: proceedings from the American College of Cardiology-Duke University Medical Center Think Tank on Quality in Cardiovascular Imaging. *J. Am. Coll. Cardiol*. 2006;48(10):2141-2151.

62. Douglas PS, Chen J, Gillam L, et al. Achieving Quality in Cardiovascular Imaging II: Proceedings From the Second American College of Cardiology-Duke University Medical Center Think Tank on Quality in Cardiovascular Imaging. *J Am Coll Cardiol Img*. 2009;2(2):231-240.

63. Lauer MS. CT angiography: first things first. *Circ Cardiovasc Imaging*. 2009;2(1):1-3.

64. Tunis SR, Stryer DB, Clancy CM. Practical clinical trials: increasing the value of clinical research for decision making in clinical and health policy. *JAMA*. 2003;290(12):1624-1632.

65. Lucas FL, DeLorenzo MA, Siewers AE, Wennberg DE. Temporal trends in the utilization of diagnostic testing and treatments for cardiovascular disease in the United States, 1993-2001. *Circulation*. 2006;113(3):374-379.

66. *Medicare Part B imaging services. Rapid spending growth and shift to physician offices indicate need for CMS to consider additional management practices*. Washington, DC: Government Accountability Office; 2008:55. Available at: http://www.gao.gov/new.items/d08452.pdf [Accessed February 10, 2011].

67. Hendel RC. Utilization Management of Cardiovascular Imaging: Pre-Certification and Appropriateness. *J Am Coll Cardiol Img*. 2008;1(2):241-248.

68. Sharples L, Hughes V, Crean A, et al. Cost-effectiveness of functional cardiac testing in the diagnosis and management of coronary artery disease: a randomised controlled trial. The CECaT trial. *Health Technol Assess*. 2007;11(49):iii-iv, ix-115.

69. Ladapo JA, Hoffmann U, Bamberg F, et al. Cost-effectiveness of coronary MDCT in the triage of patients with acute chest pain. *AJR Am J Roentgenol*. 2008;191(2):455-463.

70. Chow BJW, Abraham A, Wells GA, et al. Diagnostic accuracy and impact of computed tomographic coronary angiography on utilization of invasive coronary angiography. *Circ Cardiovasc Imaging*. 2009;2(1):16-23.

71. Hunink MGM, Krestin GP. Study design for concurrent development, assessment, and implementation of new diagnostic imaging technology. *Radiology*. 2002;222(3):604-614.

72. Meijboom WB, van Mieghem CAG, Mollet NR, et al. 64-slice computed tomography coronary angiography in patients with high, intermediate, or low pretest probability of significant coronary artery disease. *J. Am. Coll. Cardiol*. 2007;50(15):1469-1475.

73. Taylor AJ. Finding the pathway to improving patient outcomes with atherosclerosis imaging: who's motivated? *Am. Heart J*. 2007;154(6):1008-1010.

74. Pellikka PA, Nagueh SF, Elhendy AA, Kuehl CA, Sawada SG. American Society of Echocardiography recommendations for performance, interpretation, and application of stress echocardiography. *J Am Soc Echocardiogr*. 2007;20(9):1021-1041.

75. Abbara S, Arbab-Zadeh A, Callister TQ, et al. SCCT guidelines for performance of coronary computed tomographic angiography: a report of the Society of Cardiovascular Computed Tomography Guidelines Committee. *J Cardiovasc Comput Tomogr*. 2009;3(3):190-204.

76. Raff GL, Abidov A, Achenbach S, et al. SCCT guidelines for the interpretation and reporting of coronary computed tomographic angiography. *J Cardiovasc Comput Tomogr*. 2009;3(2):122-136.

77. Imaging Guidelines for Nuclear Cardiology Procedures: SPECT and Instrumentation. *Journal of Nuclear Cardiology*. 2007;14(6):913-913.

78. Beller GA, Bonow RO, Fuster V. ACCF 2008 Recommendations for Training in Adult Cardiovascular Medicine Core Cardiology Training (COCATS 3) (revision of the 2002 COCATS Training Statement). *J. Am. Coll. Cardiol*. 2008;51(3):335-338.

79. Ficaro EP, Zanzonico P, Stabin MG, et al. Variability in radiation dose estimates from nuclear and computed tomography diagnostic imaging. 2008. Available at: http://www.asnc.org/imageuploads/InformationStatementRadiationDosimetry2009.pdf [Accessed February 11, 2011].

80. Bedetti G, Botto N, Andreassi MG, et al. Cumulative patient effective dose in cardiology. *Br J Radiol*. 2008;81(969):699-705.

81. Einstein AJ, Henzlova MJ, Rajagopalan S. Estimating risk of cancer associated with radiation exposure from 64-slice computed tomography coronary angiography. *JAMA*. 2007;298(3):317-323.

82. Kalbfleisch J. *The statistical analysis of failure time data*. 2nd ed. Hoboken N.J.: J. Wiley; 2002.

83. Cox DR. Regression Models and Life-Tables. *Journal of the Royal Statistical Society. Series B (Methodological)*. 1972;34(2):187-220.

84. Schoenfeld DA. Sample-size formula for the proportional-hazards regression model. *Biometrics*. 1983;39(2):499-503.

85. Borenstein M. The case for confidence intervals in controlled clinical trials. *Control Clin Trials*. 1994;15(5):411-428.

86. Kaplan E, Meier P. Nonparametric estimation from incomplete observations. *Journal of the American Statistical Association*. 1958:457-481.

87. Begg CB, Greenes RA. Assessment of diagnostic tests when disease verification is subject to selection bias. *Biometrics*. 1983;39(1):207-215.

88. Kosinski AS, Barnhart HX. A global sensitivity analysis of performance of a medical diagnostic test when verification bias is present. *Stat Med*. 2003;22(17):2711-2721.

89. Alonzo TA, Pepe MS. Assessing accuracy of a continuous screening test in the presence of verification bias. *J Royal Statistical Soc C*. 2005;54(1):173-190.

90. Hedeker D, Gibbons RD. A random-effects ordinal regression model for multilevel analysis. *Biometrics*. 1994;50(4):933-944.

91. Bang H, Tsiatis AA. Median regression with censored cost data. *Biometrics*. 2002;58(3):643-649.

92. O'Brien PC, Fleming TR. A multiple testing procedure for clinical trials. *Biometrics*. 1979;35(3):549-556.

93. Lan GKK, DeMets DL. Discrete sequential boundaries for clinical trials. *Biometrika*. 1983;70(3):659 -663.

Statistical Analysis Plan for PROMISE

22 December 2014
Duke Clinical Research Institute

Protocol Date: April 12, 2010 (amended April 30, 2013)
Sponsor: National Heart, Lung and Blood Institute

Table of Contents

1. Study Title 3
2. Study Overview 3
3. Objectives 3
3.1 Primary Objective 3
3.2 Secondary Objectives 4
4. Subject Randomization 4
5. Primary Analysis Population 5
6. General Methodology 5
7. Endpoint Analyses 5
7.1 Primary Endpoint 5
7.2 Secondary Endpoints 7
7.2.1 Secondary Endpoint #1 7
7.2.1.1 Secondary Endpoint #1 – Alternate Definition of Non-Obstructive CAD 7
7.2.2 Secondary Endpoint #2 8
7.2.3 Secondary Endpoint #3 8
7.2.3.1 Descriptive Comparisons of (a) Death, and (b) Myocardial Infarction 8
7.2.4 Secondary Endpoint #4 8
7.2.5 Secondary Endpoint #5 9
7.2.5.1 Secondary Endpoint #5 – Alternate Definition of Non-Obstructive CAD 10
7.2.6 Secondary Endpoint #6 10
7.2.7 Secondary Endpoint #7 11
7.2.8 Secondary Endpoint #8 12
8. Subgroup Analyses 13
9. Safety Evaluation 14
10. Incidental Findings 15
11. Interim Analyses 16
12. Per-Protocol and 'As-Tested Analyses 16
13. Classification of Test Results 16
14. Analysis of Diagnostic Accuracy 17
15. Prognostic (Predictive) Ability of the Diagnostic Tests 17
16. Broader Assessment of Prognostic Factors 18
17. Changes from the Study Protocol Reflected in this Analysis Plan 19
18. Overall Interpretation and Impact of PROMISE 19
Appendix A 20
Appendix B - References 21

1. Study Title

PROspective **M**ulticenter **I**maging **S**tudy for **E**valuation of Chest Pain (PROMISE).

2. Study Overview

PROMISE is a multi-center, randomized pragmatic trial comparing two state-of-the-art diagnostic testing strategies in approximately 10,000 symptomatic, low to intermediate risk subjects with suspected coronary artery disease (CAD) who require non-urgent diagnostic testing. The investigational arm will use an "anatomic" testing strategy with coronary computed tomographic angiography (CTA) (≥64 slice) as the initial test. The usual care or "functional" testing strategy will use either stress imaging (echocardiography or nuclear) or an exercise ECG as the initial test. All subsequent diagnostic and therapeutic management will be at the discretion of the treating care team. The trial will encourage adherence to evidence-based practice and document actual therapies used, but will not mandate specific care plans, which will be left to the discretion of the clinical care team. Subjects will be randomized over approximately 36 months (starting July 2010) and followed for 12-48 months at approximately 200 North American primary care, cardiology and acute care practice sites, reflecting the physician specialties and community settings where the vast majority of chest pain patients receive care.

3. Objectives

3.1 Primary Objective

The primary objective of PROMISE is to determine whether an initial anatomic testing strategy with CTA (≥64 slice) in symptomatic subjects with low to intermediate risk for CAD who require non-urgent diagnostic testing will improve clinical outcomes compared to an initial functional testing strategy (stress echo, stress nuclear or exercise ECG; usual care), over an average of 2 years of follow-up (range 1 to 4 years).

The primary endpoint is time to the first event in a composite of major cardiovascular events including:

- Death
- Myocardial infarction
- Major complications from cardiovascular procedures and testing (stroke, major bleeding, anaphylaxis, and renal failure requiring dialysis)
- Unstable angina hospitalization

3.2 Secondary Objectives

The secondary objectives are to compare the following clinical, economic, and quality-of-life outcomes in subjects randomized to initial anatomic versus functional diagnostic testing:

1. Composite endpoint consisting of each of the components of the primary endpoint plus the addition of an invasive cardiac catheterization without obstructive CAD (as defined below in secondary objective #5)
2. Death or myocardial infarction or unstable angina hospitalization
3. Death or myocardial infarction
4. Major complications from cardiovascular procedures and testing (stroke, major bleeding, anaphylaxis, and renal failure requiring dialysis)
5. Invasive cardiac catheterization without obstructive CAD (defined as no stenosis ≥50% in any major epicardial vessel, including side branches ≥2mm in diameter, on the first catheterization performed ≤90 days after randomization)
6. Medical costs, resource use, and incremental cost effectiveness
7. Radiation exposure (defined as cumulative radiation exposure of all cardiovascular tests or procedures related to testing or management of atherosclerotic CAD and performed within 90 days following randomization)
8. Health related quality of life

To aid in the interpretation of the primary and secondary endpoint comparisons, the individual components consisting of (a) death and (b) myocardial infarction will also be compared between the anatomic and functional testing arms.

4. Subject Randomization

All of the testing modalities in the study are clinically well established and performed routinely and safely across the US. No experimental treatment or testing is involved. The trial intervention is simply the random assignment of the initial diagnostic test.

Eligible subjects who have given written informed consent and meet all inclusion and no exclusion criteria will be randomly assigned in equal proportions (1:1) to either the anatomic or functional diagnostic testing arm of the trial using permuted blocks, stratified by clinical site. Prior to randomization, the managing caregiver, site investigator or authorized designee will be asked to indicate the functional test s/he would plan to use if the subject were randomized to the functional testing arm. This information will be tracked and used as an additional stratification factor in the randomization scheme to facilitate comparisons of anatomic vs. functional testing according to the type of functional test. Subject randomization will be accomplished by telephone through a centralized toll-free Interactive Voice Response System (IVRS).

If a subject is randomized but does not undergo the planned initial diagnostic test, that subject will still be followed and included in the statistical comparisons.

5. Primary Analysis Population

All randomized subjects will be included in the analysis population for assessing the primary and secondary endpoints. For all major analyses, subjects will be analyzed according to the diagnostic testing strategy to which they were randomized, regardless of subsequent additional testing or post-randomization treatment and medical care.

6. General Methodology

Medians, 25th and 75th percentiles will be presented for continuous variables; the number and percentage of patients in each category will be presented for nominal variables. Comparisons of continuous variables between the two randomized arms (anatomic versus functional diagnostic testing) (for example with respect to baseline characteristics) will be performed using the Wilcoxon rank-sum test, and comparisons of nominal variables will be performed using the chi-square test, unless otherwise specified. Appropriate statistical models and tests will be used to examine the effect of the testing strategies on the primary and secondary endpoints in the study. For time-to-event endpoints, the Cox proportional hazards model[1] will be used unless specified otherwise. For comparing groups with respect to binary outcomes, logistic regression[2] will be used. Statistical comparisons will be performed using two-sided significance tests. Additional perspective regarding the interpretation of the data will be provided through extensive use of confidence intervals and graphical displays, including plots of Kaplan-Meier[3] event rate estimates. Analyses will be performed using SAS software version 9.2 or higher (SAS Institute, Inc., Cary, NC).

7. Endpoint Analyses

7.1 Primary Endpoint

<u>The Primary Endpoint</u>: Time from randomization to the first event in a composite of major cardiovascular events consisting of:

- Death (all-cause)
- Myocardial infarction
- Major complications from cardiovascular procedures and testing (stroke, major bleeding, anaphylaxis, and renal failure requiring dialysis)
- Unstable angina hospitalization

<u>Response Variable Definition:</u>

Time from randomization to the first event among the components of the primary composite endpoint is measured (in days) for those who experienced an event and is calculated as the date of the first event minus the date of randomization. For

patients who do not experience any of the component events or who withdraw consent or drop out of the study before it is complete, time from randomization to the date of last contact will be used in the analysis, and those patients will be considered as censored observations in the time-to-event analysis. All possible events are carefully reviewed and adjudicated by a clinical events committee (CEC) blinded to the randomized testing assignment. Definitions for each of the events are outlined in the study protocol as well as in the CEC charter. To qualify as an endpoint, the major complications from cardiovascular testing and procedures must occur during or within 72 hours following the test or procedure.

Statistical Methods: The Cox proportional hazards model[1] (PROC PHREG in SAS) will be the primary analytic tool for assessing outcome differences between the two randomized arms with respect to the primary composite endpoint. To appropriately account for heterogeneity among the subjects, the primary analysis for comparing the diagnostic testing arms will involve covariate adjustment using the pre-specified baseline prognostic variables listed in Appendix A. A hazard ratio and 95% confidence interval (CI) summarizing the difference in outcome between the two randomized arms will be computed using the Cox model[1]. The level of significance for the assessment of the primary endpoint will be α=0.05.

In addition to the statistical hypothesis testing, cumulative event rates will be calculated according to the method of Kaplan and Meier[3] for each randomized arm as a function of follow-up time (time from randomization), and the estimated event probabilities will be displayed graphically.

Given the relevance of clinical outcomes during the first year following non-invasive testing, and the study plan that all patients were to be followed for a minimum of 12 months, a comparison of the randomized arms based solely on the first 12 months of follow-up will be performed using the methods described above. For this analysis, patients not experiencing a primary event during the first year will have their follow-up censored after 12 months. The information from this analysis is only supplementary, however, to the primary comparison of the randomized arms, which is based on the total duration of follow-up available for each patient.

Non-inferiority Analysis: If the data (based on the entire follow-up experience) do not provide statistical evidence that the CTA testing strategy is superior to functional testing with respect to the primary endpoint, a test for non-inferiority of the CTA testing strategy will be performed[4]. This assessment will be based on a pre-specified non-inferiority margin of 1.10 (expressed as a hazard ratio for CTA vs. functional testing). The non-inferiority assessment will be performed by comparing the upper limit of the two-sided 95% confidence interval for the hazard ratio with the non-inferiority margin. If the upper limit of the confidence interval falls below 1.10, non-inferiority will have been demonstrated. The p-value associated with the non-inferiority test will be reported as well. We emphasize that the superiority hypothesis will be assessed first, and if significant, the non-

inferiority assessment will not be performed. Only if superiority is not demonstrated will the non-inferiority analysis be performed.

7.2 Secondary Endpoints

The secondary endpoints for the study will be analyzed and interpreted in the order in which they are presented below.

7.2.1 Secondary Endpoint #1

Time from randomization to the first event in a composite consisting of the following:

- Each component of the primary endpoint
- Invasive cardiac catheterization without obstructive CAD (defined as no stenosis ≥50% in any major epicardial coronary artery, including side branches ≥2mm in diameter, on the first catheterization performed ≤90 days following randomization)

Note: The obstructive CAD definition used in PROMISE is based on the Guideline and National Cardiovascular Data Registry definition aimed at identifying patients with anatomy suitable for either percutaneous or surgical revascularization. This definition is meant to represent the anatomic threshold for clinical action with respect to possible revascularization. In PROMISE, the classification of non-obstructive CAD is based on visual assessment and is adjudicated by a clinical events committee blinded to the randomized testing strategy.

The analysis of this secondary endpoint will be similar to and use the same methods as described for the primary endpoint in Section 7.1, including adjustment for the same set of covariates.

By definition, the only patients who can experience the component consisting of catheterization without obstructive CAD are those who undergo a cardiac catheterization within 90 days following randomization and meet the anatomic criterion defined above. All other patients (those who have a catheterization which reveals anatomically obstructive CAD, and those not undergoing cardiac catheterization within 90 days of randomization) will have the identical time to first event or censoring for this secondary composite endpoint as for the primary composite endpoint.

7.2.1.1 Secondary Endpoint #1 – Alternate Definition of Non-Obstructive CAD

A supplementary analysis of secondary endpoint #1 will be performed using a definition of non-obstructive CAD which incorporates coronary pressure assessment (function) as measured by *fractional flow reserve* (FFR) where available. In this case, invasive cardiac catheterization without obstructive CAD for an individual patient is defined as FFR>0.80 for all major coronary arteries, or if catheterization was performed but FFR data are not available, the anatomic

criterion of <50% stenosis in all major coronary arteries as defined in 7.2.1 above will apply. With the exception of this alternate definition of non-obstructive CAD, each patient's time to their first event or censoring for this analysis will be calculated as in 7.2.1 above.

7.2.2 Secondary Endpoint #2

Time from randomization to the first event in a composite of the following major clinical events:

- Death
- Myocardial infarction
- Unstable angina hospitalization

The analysis of this secondary endpoint will also use the same methods described for the primary endpoint in Section 7.1.

7.2.3 Secondary Endpoint #3

Time from randomization to the first event in a composite of the following major cardiovascular events:

- Death
- Myocardial infarction

The analysis of this secondary endpoint will also be similar to the approach described for the primary endpoint in Section 7.1.

7.2.3.1 Descriptive Comparisons of (a) Death, and (b) Myocardial Infarction

Although the individual components of the primary and secondary endpoints listed above consisting of (a) death and (b) myocardial infarction are not specifically listed as secondary endpoints in the study protocol, the incidence of each of these components will be summarized and compared between the anatomic and functional testing arms to aid in the interpretation of the primary and secondary endpoint comparisons described above.

7.2.4 Secondary Endpoint #4

Time from randomization to the first event in a composite of the following major complications from cardiovascular procedures or testing including:

- Stroke
- Major bleeding
- Anaphylaxis
- Renal failure requiring dialysis

In contrast to secondary endpoints 1 through 3, this particular endpoint and the comparisons between the randomized arms can be affected by the *competing risk* of death. For example, whether a patient experiences a stroke, major bleeding,

or any of the other component outcomes of this endpoint might be unobservable because the patient died before one of those events occurred. This can be problematic, particularly if there is a higher death rate in one randomized arm compared to the other arm. Therefore, the analysis of this endpoint will employ the competing risks methodology of Fine and Gray[5] and make use of the cumulative incidence function (i.e., the marginal failure sub-distribution associated with the component events of this endpoint) rather than treating death as a censoring event and using the Kaplan-Meier method of estimating the event-rate function. (We note that in the presence of competing risks, the Kaplan-Meier method of estimating the event-rate function can be biased, because the assumption that a subject will experience the event of interest if the follow-up period is simply long enough is not appropriate.) The analysis of this endpoint will be performed using the proportional hazards model for the sub-distribution of interest, i.e., for modeling the cumulative incidence of the events comprising the components of this endpoint. This will be accomplished using the features of the Fine-Gray[5] approach for handling competing risks data that are available as part of the PHREG procedure in the latest version of SAS. Assuming there is an adequate number of these major complications, the cumulative incidence functions for the two arms will be estimated, statistically compared, and graphically presented using these features in SAS. In addition, the cumulative incidence of each individual complication comprising this composite outcome will be tabulated to aid in the interpretation of any differences that emerge between testing strategies with respect to these major complications. Again we note that to qualify as an endpoint, the major complications from cardiovascular testing and procedures must occur during or within 72 hours following the test or procedure.

Finally, the competing risk analysis of this endpoint will be supplemented with further analyses by considering these major complications or death as a composite endpoint and using the same methodology as described in Section 7.1.

If the number of major complications proves to be small, the frequency in each randomized arm will be reported and compared with an appropriate two-sample test procedure such as Fisher's Exact Test.

7.2.5 Secondary Endpoint #5

Incidence of:

- Invasive cardiac catheterization without anatomic obstructive CAD

This endpoint is defined in Section 7.2.1 above. By definition, the only patients who can experience this endpoint (catheterization without obstructive CAD) are those who undergo a cardiac catheterization within 90 days following randomization. All other patients (those who have a catheterization which reveals anatomically obstructive CAD ($\geq$50% stenosis), and those followed for at least 90 days after randomization who do not undergo cardiac catheterization in that time interval clearly do not meet this endpoint. Although the major interest is simply whether or not an invasive catheterization without obstructive CAD occurred within 90 days following randomization (irrespective of the timing when it occurred), the analysis can be complicated by the fact that some patients may die or withdraw consent prior to the 90-day time-point and therefore are not observable for the entire 90-day period. While the number of such patients is

expected to be small, they will be properly taken into account by using the Fine-Gray competing risk methodology[5] described in section 7.2.4 above as implemented in the SAS procedure PHREG. Patients whose length of follow-up is more than 90 days (which will be most of the study patients) will be censored at 90 days in this analysis. The 90-day cumulative incidence rate of this event for each randomized arm will be calculated as part of the analysis to aid in the interpretation of the results.

7.2.5.1 Secondary Endpoint #5 – Alternate Definition of Non-Obstructive CAD

A supplementary analysis of secondary endpoint #5 will be performed using the alternate definition of non-obstructive CAD described in Section 7.2.1.1, which incorporates *fractional flow reserve* (FFR) where available. In this case invasive cardiac catheterization without obstructive CAD for an individual patient is defined as FFR>0.80 for all major coronary arteries, or if catheterization was performed but FFR data are not available, the anatomic criterion of <50% stenosis in all major coronary arteries as defined in Section 7.2.1 will apply. All other aspects of the analysis will be identical to the description in 7.2.5 above.

7.2.6 Secondary Endpoint #6

This is actually not a single endpoint, but rather a set of economic outcomes consisting of:

- Medical costs, resource use, and incremental cost effectiveness

The analytic approach to compare the economic impact of the two testing strategies will consist of two major parts, namely an empirical cost comparison of the testing strategies as randomized and a cost-effectiveness analysis. Primary statistical comparisons between the two randomized arms of empirical costs will include confidence limits around the observed cost differences constructed using bootstrap methods[6].

The cost-effectiveness analysis will estimate the incremental cost required to add an extra life year with the investigational anatomic arm relative to the control functional testing arm. In secondary analyses, we will incorporate utility weights to estimate the incremental cost per quality-adjusted life year gained with the CTA anatomic strategy, relative to the functional testing strategy. These analyses will be conducted from a societal perspective and will use a lifetime time horizon so that the estimated incremental cost-effectiveness and cost-utility ratios can be compared with societal benchmarks. We will also calculate within-trial cost-effectiveness/cost-utility ratios, although these ratios are limited in their value due to their failure to account for long-term benefits and costs and the absence of comparative benchmarks. Costs will be adjusted for inflation, and both costs and life expectancy will be discounted to present value at a 3% annual discount rate. Adjustments for censored data due to staggered entry will be made following the approach of Bang and Tsiatis[7]. Extensive sensitivity analyses will be performed.

Additional details of the economic analyses will be described in a separate analysis plan prepared by the Economics and Quality of Life (EQOL) Coordinating Center at the Duke Clinical Research Institute.

7.2.7 Secondary Endpoint #7

- Cumulative radiation exposure

The radiation exposure endpoint (as described in Section 3.2) is defined as the cumulative radiation exposure from all cardiovascular diagnostic testing and procedures related to testing or management of atherosclerotic CAD and performed within 90 days following randomization. The testing and procedures will include CTA, stress nuclear imaging, diagnostic cardiac catheterization, and catheterization with percutaneous coronary intervention (PCI). For each patient, the amount of radiation exposure will be calculated as detailed below.

a. For the initial diagnostic test (either CTA or stress nuclear imaging), radiation exposure for each patient was measured by the sites and documented by the Diagnostic Testing Coordinating Center (DTCC) using standard methodology[8].
b. Radiation exposure for all subsequent diagnostic tests (CTA or nuclear imaging) will be estimated using multiple imputation based on the type of test performed, the CTA scanner or tracer protocol (if available), the radiation exposure as observed for the initial tests in the trial, and patient demographics.
c. For patients undergoing cardiac catheterization, radiation will be estimated (imputed) based on data from the literature[9]. The imputed value will depend on whether the catheterization was diagnostic only (in which case the imputed value will be 7mSv), or whether a PCI was performed (in which case the imputed value will be 15 mSv).

Handling Missing Data: Any patients with missing radiation data for the initial diagnostic test will have their exposure estimated using multiple imputation as in (b) above based on the diagnostic test received and the radiation exposure measurements available from the other patients. If a patient did not undergo any diagnostic test or cardiac procedure, their radiation dose will be zero.

Specific Analyses:

(1) Comparison of the diagnostic testing arms as randomized with respect to *cumulative radiation exposure from all cardiovascular diagnostic tests and procedures* performed within 90 days following randomization. Patients who did not undergo either a diagnostic test or cardiac procedure within 90 days or who underwent a functional test with no radiation will be assigned an exposure value of zero.

(2) Comparison of the diagnostic testing arms as randomized with respect to *radiation exposure of the initial diagnostic test* performed within 90 days following randomization. This comparison will include all randomized patients. Any patients who did not undergo either a CTA, a nuclear imaging test, or a cardiac catheterization as the initial diagnostic test performed within the 90-day period will be assigned an exposure value of zero.

(3) Comparison of radiation exposure of the initial diagnostic test *according to the initial non-invasive test performed* (CTA vs. nuclear imaging). This comparison will be performed (i) in all patients whose initial diagnostic test was either CTA or nuclear imaging, and (ii) in patients undergoing either test in the randomization stratum consisting of patients where the enrolling site specified that if the patient was randomized to a functional test, stress nuclear imaging would be performed.

Statistical Methods: The distribution of cumulative radiation exposure in each randomized arm will be descriptively summarized (using means and standard deviations, medians, percentiles, and cumulative distribution functions) and statistically compared between the randomized arms and between different testing modalities using appropriate two-sample test methodology.

There is a sub-study within PROMISE on radiation exposure (PROMISE-SAFER) in which the radiation exposure data will be analyzed in greater detail. PROMISE-SAFER will have a separate analytic plan developed by the DCRI and the SAFER investigators at Columbia University.

7.2.8 Secondary Endpoint #8

- Health-related quality of life (QOL).

Again, this is not a single outcome, but rather a comprehensive assessment of multiple domains of quality of life. For each of the QOL measures, data analysis will proceed in several stages. We will start by providing simple descriptive and comparative analyses of the testing strategies as randomized. A nonparametric bootstrap will be used to estimate treatment differences with 95% CIs and p-values. Because there is currently no consensus in the statistical literature about the best way to deal with the multiple comparisons problem arising from testing each individual scale at each time point separately, two complementary approaches will be employed. First, we pre-specify the angina frequency and disease perception/QOL scales from the Seattle Angina Questionnaire (SAQ) as the CAD-specific measures of primary interest, since those measures most directly quantify the therapeutic goal of coronary diagnosis and therapy, namely to minimize symptoms and optimize patients' QOL. We also specify cardiac functional status measured with the Duke Activity Status Index[10] (DASI) as a primary QOL outcome measure of interest. Other disease-specific and generic QOL measures are therefore assigned to a secondary (descriptive) status in the analyses. Second, we will fit a mixed effects longitudinal proportional odds model[11] that makes use of all available QOL data at each study assessment point to model the time profile (fixed effect) using a restricted cubic spline function. Using the fitted model, we can estimate the overall difference in the QOL measures as well as test the global hypothesis of no difference over time. We can also estimate the difference in the areas under the two QOL curves corresponding to the two randomized arms (and test the hypothesis of no difference on average). In addition, we will estimate differences in QOL at the end of the study or at intermediate points such as at 1 year. A separate more detailed analytic plan

for the quality of life analyses will also be developed by the EQOL Coordinating Center.

8. Subgroup Analyses

An assessment will be made as to whether the effect (if any) of testing strategy is similar for all patients, or whether it varies according to specific patient characteristics. In particular, these analyses will focus on whether the relative effect of testing strategy differs according to the following baseline variables:

- Age (<65 years vs. ≥65 years)
- Gender (Male vs. Female)
- Race (White vs. Non-white)
- Baseline pre-test assessment of the risk of significant epicardial stenosis (≥70%) or left main stenosis ≥50% as assessed by the site investigator [low risk (≤30% likelihood), intermediate risk (31-70% likelihood), and high risk (>70% likelihood)]
- Coronary disease risk equivalent (history of either diabetes, cerebrovascular disease, or peripheral artery disease) vs. none of these factors
- Characteristics of the precipitating symptoms (Angina type classification—typical, atypical or non-anginal pain[12]), as reported for each patient by the enrolling site
- Combined Diamond-Forrester and CASS score[12-14] indicating the pre-test likelihood of underlying CAD, categorized as low (<10%), intermediate (10-90%, and high (>90%)
- Pre-randomization choice of the functional test to be used if the subject was randomized to the functional testing arm. Specification of this choice was required of the site investigator prior to randomization and was built into the randomization scheme as a stratification factor to ensure that the benefits of randomization in this subgroup comparison were maintained.

These analyses will utilize the Cox model and will be accomplished by testing for interactions between the randomized testing strategy and these specific baseline variables. In addition to the formal assessment of the randomized testing strategy by covariate interactions, effects of the diagnostic testing strategy characterized by a hazard ratio and 95% confidence interval will be calculated and displayed using a forest plot for the subgroups of subjects defined by the variables listed above. These descriptive hazard ratios will be carefully interpreted in conjunction with the formal interaction tests.

The effect of diagnostic testing strategy may also be examined in other subgroups of clinical interest (in addition to those listed above).

9. Safety Evaluation

The overall safety profiles of the diagnostic testing strategies evaluated in PROMISE include:

- Major complications (these are the major complications that are components of the primary endpoint and therefore will be addressed and reported in the primary study report)
- Minor complications (briefly discussed below; will be reported in a separate comprehensive report of overall safety in the trial)
- Radiation exposure (discussed above in connection with Secondary Endpoint #7)

We note that for safety and ethical reasons, interim examinations of key safety data were performed at regular intervals during the course of the trial. These interim assessments were prepared for review by the Data and Safety Monitoring Board (DSMB).

Because the only intervention in the trial is the randomized assignment of the initial test to be performed, the only safety events arising from the study are related to the initial test. Mild safety events are considered as related to testing only up to 24 hours after the initial randomized test or the first test if it is other than the randomized test, and have been collected and reported by site personnel using the InForm™ electronic data capture system. These safety events include the following:

For CTA:

1. Mild contrast reaction such as rash and hives (severe reactions including anaphylaxis or death are part of the primary endpoint)
2. Extravasation of contrast into the surrounding tissue of the extremity where the IV was placed and contrast administered
3. Hemodynamic instability, including symptomatic bradycardia or hypotension, due to the beta blockade or nitrates given for the CTA scan acquisition
4. Acute bronchospasm due to the beta blockade given for the CTA scan

For exercise testing during exercise ECG:

1. Exercise-induced hypotension with a drop in systolic BP > 20mmHg
2. Stress-induced symptoms that do not resolve within 20 minutes
3. Rapid atrial fibrillation that does not slow or convert with treatment
4. Ventricular tachycardia
5. Hemodynamic instability defined as systolic BP < 80 mmHg

6. Hospital admission not otherwise captured by the primary endpoint, including that precipitated by any symptomatic event (chest pain, dyspnea, etc), persistent or worsening ischemic ECG changes, any bradycardic or tachycardic arrhythmia or any hemodynamic changes (hyper- or hypo-tension)

For stress nuclear:

1. The above events for exercise testing
2. Any events potentially related to the use of vasodilators such as dypridamole or adenosine.

For stress echo:

1. The above events for exercise testing
2. Stress induced wall motion abnormality that does not resolve within 20 minutes (despite treatment)
3. Any events potentially related to the use of dobutamine as a stress agent
4. Any anaphylactic reaction to contrast agent not requiring circulatory or respiratory support

As stated in Section 7.2.4, severe events/complications related to cardiovascular testing or cardiovascular procedures which are also trial endpoints (e.g. peri-procedural MI, major bleeding, renal failure, and anaphylaxis requiring circulatory or respiratory support) will be collected throughout the duration of the trial and will be considered to be related to testing or a procedure if occurring during or within 72 hours following the test or procedure.

The frequency with which major safety events occur will be carefully tabulated and descriptively summarized. Statistical comparisons of the randomized arms with respect to these events will use chi-square or other appropriate two-sample methods, including logistic regression, depending on the nature of the event. Such comparisons will be interpreted in the context of differences between the two randomized arms in the primary and major secondary clinical endpoints, using clinical judgment as to the relative seriousness of the safety events.

10. Incidental Findings

Incidental findings (e.g., such things as lung nodules) that may be discovered with either the anatomic (CTA) testing strategy or the functional testing modalities (stress ECHO and stress Nuclear) will be tabulated and summarized descriptively. A separate analysis plan will document the specific details as to how this information will be analyzed and presented.

11. Interim Analyses

The clinical study protocol included provision for interim analyses of efficacy outcomes through use of an alpha-spending function with O'Brien-Fleming type monitoring boundaries as outlined in section F3 of the protocol. However, no interim analyses involving comparisons of the randomized groups with respect to the primary and secondary endpoints were performed. Therefore, there is no need for any statistical adjustment to the level of significance for the final primary analysis.

12. Per-Protocol and 'As-Tested' Analyses

Although the principal comparisons of the diagnostic testing strategies will be performed with the arms of the trial defined as randomized, it is expected that a small percentage of patients randomized to the anatomic testing arm will receive a functional test first, and a small percentage of patients randomized to functional testing will be evaluated initially using CTA. Therefore, supplementary comparisons of the testing strategies will be performed based on (a) only the patients who receive as their first test, the test to which they were randomized ("per-protocol" analysis), and (b) groups defined according to the first test actually received. The first test could be either CTA, one of the functional tests, or a cardiac catheterization. The comparisons of outcomes of all of these groups will be performed using the same methodology described in Section 7. It is possible in this large study that there will be some patients who did not undergo any diagnostic test. For completeness of the comparisons, the outcomes of those patients will also be descriptively summarized.

13. Classification of Test Results (Positive vs. Negative)

Among the information that will be reported from the trial is the percent of patients in the PROMISE study population whose diagnostic test was considered ***positive***, whether randomized to CTA or to a functional test. For reporting this information, the following definitions will be used.

- **CTA:** For CTA, the test will be considered positive if there is a $\geq$70% stenosis in either the left anterior descending (LAD), left circumflex (LCX), or right coronary artery (RCA), or a $\geq$50% stenosis in the left main coronary artery.
- **Functional Testing:**
    - **Stress Nuclear Imaging** will be considered positive if there is a reversible perfusion defect (inducible ischemia) during stress in at least one territory.
    - **Stress Echo** will be considered positive if there is a reversible wall motion abnormality during stress in at least one territory.
    - **Stress ECG** will be considered positive if there are significant ST-segment changes consistent with ischemia.

These classifications will be based on site-reported assessments.

14. Analysis of Diagnostic Accuracy

Although PROMISE was designed with the primary objective of evaluating initial anatomic vs. functional testing strategies with respect to clinical outcomes and thus represents a different paradigm than the traditional design to assess diagnostic accuracy, performance of the tests with respect to diagnostic accuracy will be examined in supplementary analyses. These analyses cannot be done in a conventional manner as not all subjects will undergo invasive angiography (the "gold standard"), and those who do will not undergo angiography by random selection. Instead, the decision to verify disease will be based on test results and other characteristics of the subject. This nonrandom selection process will likely result in a strong verification bias[15], often characterized by higher than true sensitivity and lower than true specificity results. (Of note, the trial cannot require angiography in a subset of subjects for purposes of determining testing accuracy, as this would provide additional information to these subjects' physicians and care givers above and beyond that provided by the randomized testing strategy, and therefore could confound the trial results.) Correction of the verification bias is possible if the process leading to verification with angiography is known. However, this is rarely achievable, but the process can be modeled under the missing at random (MAR) assumption[16], namely that disease status affects referral to angiography only through measured covariates and not the disease status itself. In view of the inherent limitations, the following steps will be taken with respect to evaluating diagnostic accuracy.

Using the definitions stated in the previous section as to what constitutes a positive test, diagnostic accuracy rates will be assessed using conventional measures (sensitivity and specificity in subjects undergoing cardiac catheterization, bringing to bear appropriate statistical methods for dealing with verification bias. The probability of verification will be modeled with a logistic regression model using covariates that are predictors of referral to angiography. Corrected values of sensitivity and specificity will be estimated[16,17]. Assessment of the impact of potential departures from the MAR assumption on sensitivity and specificity will utilize the "test ignorance region" approach[16]. A subgroup analysis of diagnostic accuracy will be performed comparing results at sites with high volume and extensive experience or expertise in diagnostic testing with results in less-experienced or lower-volume sites. Other subgroups such as MD specialty will also be of interest.

We emphasize that these analyses of diagnostic accuracy will be strictly supplementary to the analysis of the primary and secondary clinical endpoints outlined in Sections 7.1 and 7.2. Separate analysis plans will be prepared that pre-specify in greater detail the steps of the diagnostic accuracy determinations.

15. Prognostic (Predictive) Ability of the Diagnostic Tests

The relationships of the diagnostic test results with clinical outcomes of the patients enrolled in PROMISE will be examined to assess the extent to which a

positive test result (as defined in Section 13) is associated with an increased risk of clinical events. Using the Cox regression model, the strength of the association of a positive test with primary endpoint events will be statistically assessed in all patients undergoing a diagnostic test. The effect of a positive versus negative test will be characterized using a hazard ratio and 95% confidence interval generated from the Cox model. Additionally a covariate-adjusted assessment of this relationship will be performed to see whether differences in the outcomes of patients with a positive vs. negative test persist after adjusting for other readily available clinical factors. Kaplan-Meier curves characterizing event rates for patients with a positive vs. negative test will provide further descriptive summaries.

Since the degree of association of a positive test with clinical outcomes may depend on the type of test performed (anatomic vs. functional), the interaction between type of test and whether the test is positive or negative will also be examined with the Cox model. Other measurements from the tests in addition to whether the test is simply positive or negative, including the degree of positivity, will be jointly examined using this approach.

16. Broader Assessment of Prognostic Factors

With the large database of information that will be collected on the 10,000 subjects enrolled in this study, extensive regression modeling analyses will be performed that goes beyond a prognostic assessment of the test results. These analyses will use primarily the Cox regression model to identify and assess the factors (predictors) that are associated with the clinical outcomes of these subjects. These analyses will comprehensively evaluate the strength and shape of the relationships of numerous baseline clinical factors with the clinical outcomes, in addition to characterizing the prognostic relationships of the measures that arise from the diagnostic tests. The incremental prognostic value of test results over traditional clinical descriptors will be of particular interest.

For continuous clinical variables, we will examine the relationships between individual variables and major clinical outcomes using a flexible model-fitting approach involving restricted cubic spline functions (cubic polynomials)[17]. These functions will be graphically and statistically examined to assess the linearity of the relationship with the log hazard ratio. Where relations are nonlinear, the shape will be characterized either using spline functions or an appropriate transformation of the data[18]. We will also examine whether the prognostic relationship of any important predictor variable differs for particular levels of other important descriptors by testing for interactions among the prognostic clinical variables. While these analyses are more exploratory than the rigorous pre-specified primary and secondary comparisons of the randomized arms of the trial, they will nonetheless be helpful in elucidating relationships and identifying the key factors that impact patient outcomes and any observed differences in outcomes between the diagnostic testing arms. Separate analysis plans will be prepared that will pre-specify all of the various steps of the modeling process including the specific set(s) of variables that will be considered, the specific outcomes of interest, the approach to model development and validation, and the

assessment of the predictive performance of the resulting statistical model(s) and any prognostic score(s) developed from the analyses.

17. Changes from the Study Protocol Reflected in this Analysis Plan

- As stated in Section 11 of this document, the interim analyses for which provision was included in the study protocol were not conducted.

Other changes include:

- Supplementary analyses of secondary endpoints #1 and #5 using a definition of non-obstructive CAD based on fractional flow reserve (FFR) data when available, with anatomic criteria used only if FFR was not measured.
- The use of competing risk methodology for secondary endpoints #4 and #5 to appropriately account for the competing risk of death and compute cumulative incidence estimates rather than Kaplan-Meier estimates (which may be biased due to the competing risk).

18. Overall Interpretation and Impact of PROMISE

Although Sections 3.1 and 3.2 list the primary objective and eight different secondary objectives of the trial, the overall impact and interpretation of the trial will be based on the results of the analyses, in the following order:

- Primary endpoint – superiority assessment
- Primary endpoint – non-inferiority assessment (if needed)
- Secondary endpoint #1: Primary endpoint plus invasive cardiac catheterization without obstructive CAD
- Economic outcomes
- Radiation safety

While the analyses of secondary clinical endpoints 2 through 5 listed in Section 3.2 will be important and are certainly of great interest, those endpoints are components (building blocks) of the primary endpoint and secondary endpoint #1. Therefore, a major role of those secondary analyses will be to aid in the interpretation of the analysis of the primary endpoint and key secondary endpoint #1. The real impact of the trial will rest upon the results of the analysis of the primary endpoint, followed by the outcome of key secondary endpoint #1, followed by the economic outcomes and then the radiation safety results. All other analyses will be ancillary and supportive of those pivotal results.

Appendix A

List of covariates to be included in the primary endpoint analysis:

- Age
- Sex
- Coronary disease risk equivalent (history of either diabetes, cerebrovascular disease (CVD), or peripheral artery disease (PAD))

In addition, the stratification factor used in the randomization, namely the choice of the functional test to be used if the patient was randomized to the functional testing strategy, will be included as a covariate. This particular factor is included to preserve consistency in the analysis with the stratification used in the randomization, and not because it is expected to be prognostic.

Cerebrovascular disease (CVD) will be considered present if the patient has a history of any of the following: stroke, TIA, carotid artery stenosis $\geq$ 50%, or history of carotid revascularization.

Age will be included in the Cox model analysis as a continuous variable, modeled linearly (i.e., assuming linearity in terms of the log hazard ratio).

Every effort will be made to have complete data for each of these variables in 100% of the randomized patients. Despite these vigorous efforts, if the information on CVD or PAD is missing for any patient, it will be assumed that those conditions are absent. It is expected that data will be complete for the other variables listed above.

Appendix B - References

1. Cox DR. Regression models and life-tables (with discussion). *J Royal Statist Soc B* 1972;34:187-220.
2. Walker SH, Duncan DB. Estimation of the probability of an event as a function of several independent variables. *Biometrika* 1967;54:167-178.
3. Kaplan EL, Meier P. Nonparametric estimation from incomplete observations. *J Am Statist Assn* 1958;53:457-481.
4. Rothman MD, Wiens BL, Chan ISF. *Design and Analysis of Non-Inferiority Trials.* Chapman & Hall/CRC, Boca Raton, FL, 2012.
5. Fine JP, Gray RJ. A proportional hazards model for the subdistribution of a competing risk. *J Amer Statist Assoc* 1999;94:496-509.
6. Efron B, Tibshirani R. *An Introduction to the Bootstrap.* Chapman and Hall, New York, 1993.
7. Bang H, Tsiatis AA. Median regression with censored cost data. *Biometrics* 2002;58:643-649.
8. Gerber TC, Carr JJ, Arai AE, Dixon RL, Ferrari VA, Gomes AS, Heller GV, McCollough CH, McNitt-Gray MF, Mettler FA, Mieres JH, Morin RL, Yester MV. Ionizing radiation in cardiac imaging: a science advisory from the American Heart Association Committee on Cardiac Imaging of the Council on Clinical Cardiology and Committee on Cardiovascular Imaging and Intervention of the Council on Cardiovascular Radiology and Intervention. *Circulation* 2009;119(7):1056-1065.
9. Mettler FA Jr, Huda W, Yoshizumi TT, Mahesh M. Effective doses in radiology and diagnostic nuclear medicine: a catalog. *Radiology* 2008;248(1):254-263.
10. Hlatky MA, Boineau RE, Higginbotham MB, Lee KL, Mark DB, Califf RM, Cobb FR, Pryor DB. A brief self-administered questionnaire to determine functional capacity (the Duke Activity Status Index). *Am J Cardiol* 1989;64:651-654.
11. Hedeker D, Gibbons RD. A random-effects ordinal regression model for multilevel analysis. *Biometrics*. 1994;50(4):933-944.
12. Diamond GA, Forrester JS. Analysis of probability as an aid in the clinical diagnosis of coronary-artery disease. *N Engl J Med* 1979;300:1350-1358.
13. Fihn SD, Gardin JM, Abrams J, Berra K, Blankenship JC, Dallas AP, Douglas PS, Foody JM, Gerber TC, Hinderliter AL, King SB, Kligfield PD, Krumholz HM, Kwong RY, Lim MJ, Linderbaum JA, Mack MJ, Munger MA, Prager RL, Sabik JE, Shaw LJ, Sikkema JD, Smith CR, Smith SC, Spertus JA, Williams SV. 2012 ACCF/AHA/ACP/AATS/PCNA/SCAI/STS Guideline for the Diagnosis and Management of Patients With Stable Ischemic Heart Disease: Executive Summary: A Report of the American College of Cardiology Foundation/American Heart Association Task Force on Practice Guidelines, and the American College of Physicians, American Association for Thoracic Surgery, Preventive Cardiovascular Nurses Association, Society for Cardiovascular Angiography and Interventions, and Society of Thoracic Surgeons. *J Am Coll Cardiol* 2013;60 (24):2564-2603.
14. Chaitman BR, Bourassa MG, Davis K, Rogers WJ, Tyras DH, Berger R, Kennedy JW, Fisher L, Judkins MP, Mock MB, Killip T. Angiographic

prevalence of high-risk coronary artery disease in patient subsets (CASS). *Circulation* 1981;64:360-367.

15. Begg CB, Greenes RA. Assessment of diagnostic tests when disease verification is subject to selection bias. *Biometrics*. 1983;39(1):207-215.
16. Kosinski AS, Barnhart HX. A global sensitivity analysis of performance of a medical diagnostic test when verification bias is present. *Stat Med*. 2003;22(17):2711-2721.
17. Alonzo TA, Pepe MS. Assessing accuracy of a continuous screening test in the presence of verification bias. *J Royal Statistical Soc C*. 2005;54(1):173-190.
18. Harrell FE Jr. *Regression Modeling Strategies—With Applications to Linear Models, Logistic Regression, and Survival Analysis.* Springer-Verlag, New York, 2001.
