# Supplementary material for: Prognostic Value of Plaque Volume in Patients With First Diagnosis of Coronary Artery Disease: A Substudy of the PROMISE Randomized Clinical Trial
Source: JAMA Cardiol. 2026 Feb 11;11(3):259–67. doi: 10.1001/jamacardio.2025.5520 (PMC12895320; doi:10.1001/jamacardio.2025.5520)
Supplement: Supplement 2. — eMethods eResults eTable 1. Baseline demographics by the median total plaque burden. eTable 2. Description of quantitative plaque measures. eTable 3. Baseline characteristics by plaque volume and burden measures eTable 4. Median PV measures and qualitative CT measures eTable 5. Multivariable assessment of quantitative plaque volume/burden quartiles and MACE. eTable 6. Multivariable assessment of quantitative plaque volume/burden and MACE eTable 7. Univariable and multivariable assessment of quantitative plaque volume/burden and non-fatal MI and cardiovascular death. eTable 8. Crude MACE Rates (All-cause death, MI, UAP) stratified by Plaque Volume/Burden Quartiles and CAC categories eTable 9. Cox regression hazard ratios (All-cause death, MI, UAP) for continuous plaque volume and plaque burden across CAC categories eTable 10. Univariable and multivariable assessment of quantitative plaque volume/burden data-driven thresholds and MACE. eFigure. Consort diagram. [file jamacardiol-e255520-s002.pdf]

Supplemental Online Content

Karády J, Mayrhofer T, Brendel JM, et al. Prognostic value of plaque volume in patients with first diagnosis of coronary artery disease: a substudy of the PROMISE randomized clinical trial. *JAMA Cardiol*. Published online February 11, 2026. doi:10.1001/jamacardio.2025.5520

eMethods

eResults

eTable 1. Baseline demographics by the median total plaque burden.
eTable 2. Description of quantitative plaque measures.
eTable 3. Baseline characteristics by plaque volume and burden measures
eTable 4. Median PV measures and qualitative CT measures
eTable 5. Multivariable assessment of quantitative plaque volume/burden quartiles and MACE.
eTable 6. Multivariable assessment of quantitative plaque volume/burden and MACE
eTable 7. Univariable and multivariable assessment of quantitative plaque volume/burden and non-fatal MI and cardiovascular death.
eTable 8. Crude MACE Rates (All-cause death, MI, UAP) stratified by Plaque Volume/Burden Quartiles and CAC categories
eTable 9. Cox regression hazard ratios (All-cause death, MI, UAP) for continuous plaque volume and plaque burden across CAC categories
eTable 10. Univariable and multivariable assessment of quantitative plaque volume/burden data-driven thresholds and MACE.

eFigure. Consort diagram.

This supplemental material has been provided by the authors to give readers additional information about their work.

eMethods.

CT image acquisition and analysis

CAC scoring and qualitative CCTA plaque analysis

CT-adapted Leaman score was generated with the use of 3 weighting factors: 1) coronary plaque location, accounting for coronary artery dominance; 2) plaque type, with multiplication factor 1 for calcified plaque and 1.5 for noncalcified and partially calcified plaque; 3) degree of stenosis with multiplication factor 0.615 for <50% stenosis and 1 for ≥50% stenosis lesions.[6]

© 2026 Karády J et al. *JAMA Cardiology*.

eResults

Sensitivity analysis of LAPB>4%:

198/4267 (4.6%) had LAPD>4%; Adjusted HR for age, sex, race, ASCVD risk, and statin use, continuous CAC, stenosis ≥50%, high-risk plaque for the composite outcome (all-cause death, non-fatal myocardial infarction, or hospitalization for unstable angina): 1.96, CI 95%: 1.07-3.57, p=0.029.

eTable 1. Baseline demographics by the median total plaque burden.

Mean±SD, median [IQR], or n/N(%)	Total population (n=4,267)	< 27% total plaque burden (n=2,134)	≥ 27% total plaque burden (n=2,133)	p
**Demographics**				
Age, years	60.4 ± 8.2	59.0 ± 7.8	61.8 ± 8.3	**<.001**
Female Sex	2,199/4,267 (51.5)	1,276/2,134 (59.8)	923/2,133 (43.3)	**<.001**
Race				
White	3,289/4,223 (77.9)	1,600/2,114 (75.7)	1,689/2,109 (80.1)	**<.001**
Black	427/4,223 (10.1)	252/2,114 (11.9)	175/2,109 (8.3)	**<.001**
Asian	128/4,223 (3.0)	59/2,114 (2.8)	69/2,109 (3.3)	0.37
Racial or ethnic minority	952/4,241 (22.5)	522/2,122 (24.6)	430/2,119 (20.3)	**<.001**
Ethnicity				
Hispanic or Latino	304/4,223 (7.2)	166/2,114 (7.9)	138/2,109 (6.5)	0.11
Not Hispanic or Latino	3,919/4,223 (92.8)	1,948/2,114 (92.2)	1,971/2,109 (93.5)	0.11
**Cardiac Risk Factors**				
BMI, kg/m^2	30.3 ± 5.9	30.4 ± 6.0	30.2 ± 5.7	0.28
Hypertension	2,716/4,267 (63.7)	1,302/2,134 (61.0)	1,414/2,133 (66.3)	**<.001**
Diabetes	865/4,267 (20.3)	366/2,134 (17.2)	499/2,133 (23.4)	**<.001**
Dyslipidemia	2,862/4,267 (67.1)	1,384/2,134 (64.9)	1,478/2,133 (69.3)	**.002**
Smoking (ever)	2,184/4,266 (51.2)	980/2,133 (45.9)	1,204/2,133 (56.5)	**<.001**
Family history of premature CAD	1,400/4,253 (32.9)	659/2,128 (31.0)	741/2,125 (34.9)	**.007**
History of depression	840/4,267 (19.7)	449/2,134 (21.0)	391/2,133 (18.3)	0.03
Participate in physical activity	2,049/4,258 (48.1)	1,031/2,128 (48.5)	1,018/2,130 (47.8)	0.69
Peripheral artery disease	211/4,266 (5.0)	89/2,134 (4.2)	122/2,133 (5.7)	**.020**
CAD equivalent	1,018/4,267 (23.9)	435/2,134 (20.4)	583/2,133 (27.3)	**<.001**
Sedentary lifestyle	2,209/4,258 (51.9)	1,097/2,128 (51.6)	1,112/2,120 (52.2)	0.69
Metabolic syndrome	1,558/4,267 (36.5)	736/2,134 (34.5)	822/2,133 (38.5)	**.006**
No risk factor	111/4,267 (2.6)	60/2,134 (2.8)	51/2,133 (2.4)	0.44
Risk factor burden	2.35 [2.32-2.38]	2.20 [2.15-2.24]	2.50 [2.46-2.55]	**<.001**
**Medication Use**				
Aspirin	1,840/4,080 (45.1)	833/2,020 (41.2)	1,007/2,060 (48.9)	**<.001**
Statin	1,857/4,080 (45.5)	833/2,020 (41.2)	1,024/2,060 (49.7)	**<.001**
Beta-blocker	996/4,080 (24.4)	488/2,020 (24.2)	508/2,060 (24.7)	0.72
ACEi or ARB	1,738/4,080 (42.6)	817/2,020 (40.5)	921/2,060 (44.7)	**.006**
**ASCVD risk**				
ASCVD risk (2013), %	11.0 [6.1-19.1]	8.4 [4.7-14.6]	13.6 [8.1-22.7]	**<.001**
ASCVD risk categories				**<.001**
<5%	795/4,221 (18.8)	573/2,114 (27.1)	222/2,107 (10.5)	
5% to <7.5%	614/4,221 (14.6)	381/2,114 (18.0)	233/2,107 (11.0)	
7.5% to <20%	1,846/4,221 (43.7)	839/2,114 (39.7)	1,007/2,107 (47.8)	
≥20%	966/4,221 (22.9)	321/2,114 (15.2)	645/2,107 (30.6)	

© 2026 Karády J et al. *JAMA Cardiology*.

ACEi = angiotensin converting enzyme inhibitor; ARB = Angiotensin II receptor blocker; ASCVD = atherosclerotic cardiovascular disease; BMI = body mass index; CAD = coronary artery disease; CVD = cardiovascular disease.

© 2026 Karády J et al. *JAMA Cardiology*.

eTable 2. Description of quantitative plaque measures.

	Plaque Volume (mm^3)				Plaque Burden (%)			
**All patients (n=4,267)**	**Total**	**Calcified**	**Non-calcified**	**Low attenuation**	**Total**	**Calcified**	**Non-calcified**	**Low attenuation**
Mean ± SD	139 ± 256	50.3 ± 116	88.6 ± 166	4.27 ± 16.0	25.0 ± 21.6	8.41 ± 9.99	16.6 ± 16.5	0.74 ± 2.10
Median [IQR]	39.8 [0.00-167]	6.66 [0.00-50]	22.5 [0.00-101]	0.03 [0.00-2.0]	27.0 [0.00-43]	4.49 [0.00-15]	14.1 [0.00-29]	0.02 [0.00-0.52]
Range	0.00-5,060	0.00-2,999	0.00-2647	0.00-488	0.00-82.3	0.00-60.5	0.00-82.3	0.00-32.2
**Patients with plaque (n=2,754)**	**Total**	**Calcified**	**Non-calcified**	**Low attenuation**	**Total**	**Calcified**	**Non-calcified**	**Low attenuation**
Mean ± SD	215±291	78.0±137	137±190	6.62±19.5	38.7±13.9	13.0±9.71	25.6±13.8	1.15±2.52
Median [IQR]	114 [44.8-268]	29.6 [7.97-93]	71.0 [26.2-172]	0.91 [0.06-5.09]	38.4 [28.2-47.9]	11.6 [5.03-19.1]	24.9 [15.2-34.8]	0.27 [0.03-1.13]
Range	0.78-5,060	0.00-2,999	0.00-2,647	0.00-488	3.84-82.3	0.00-60.5	0.00-82.3	0.00-32.2

© 2026 Karády J et al. *JAMA Cardiology*.

eTable 3. Baseline characteristics by plaque volume and burden measures.

Median [IQR]	Plaque volume (mm^3)								Plaque burden (%)							
	**Total**	**p**	**Calcified**	**p**	**Non-calcified**	**p**	**Low attenuation**	**p**	**Total**	**p**	**Calcified**	**p**	**Non-calcified**	**p**	**Low attenuation**	**p**
Age		**<.001**		**<.001**		**<.001**		**<.001**		**<.001**		**<0.001**		**<0.001**		**.002**
<65	25.3 [0.00-133]		2.59 [0.00-32.9]		15.0 [0.00-90.9]		0.00 [0.00-1.84]		24.1 [0.00-40.3]		1.99 [0.00-12.0]		12.3 [0.00-28.6]		0.00 [0.00-0.55]	
65-74	75.6 [6.69-237]		22.8 [0.13-90.2]		40.3 [1.44-130]		0.16 [0.00-2.44]		33.7 [13.5-46.5]		10.1 [0.14-19.4]		17.3 [1.52-30.0]		0.06 [0.00-0.48]	
≥75	123 [32.5-297]		47.9 [6.56-140]		57.8 [11.6-151]		0.31 [0.00-2.56]		33.3 [23.0-46.9]		15.1 [4.60-22.5]		19.3 [7.07-30.5]		0.08 [0.00-0.47]	
Sex		**<.001**		**<.001**		**<.001**		**<.001**		**<.001**		**<0.001**		**<0.001**		**<.001**
Female	13.3 [0.00-90.8]		1.41 [0.00-27.2]		5.03 [0.00-54.4]		0.00 [0.00-0.59]		19.7 [0.00-39.7]		1.39 [0.00-14.6]		5.70 [0.00-25.4]		0.00 [0.00-0.22]	
Male	85.5 [0.00-258]		16.1 [0.00-77.7]		52.6 [0.00-173]		0.47 [0.00-5.02]		32.0 [0.00-44.2]		6.15 [0.00-15.1]		20.1 [0.00-31.8]		0.15 [0.00-0.93]	
Race																
White	45.9 [0.00-183]	**<.001**	8.75 [0.00-28.8]	**<.001**	26.1 [0.00-109]	**<.001**	0.06 [0.00-2.31]	**<.001**	28.0 [0.00-43.0]	**<.001**	5.30 [0.00-15.4]	**<.001**	14.9 [0.00-29.1]	**.003**	0.03 [0.00-0.57]	**<.001**
Black	6.94 [0.00-91.6]	**<.001**	0.00 [0.00-16.3]	**<.001**	3.88 [0.00-66.4]	**<.001**	0.00 [0.00-0.81]	**<.001**	14.6 [0.00-39.2]	**<.001**	0.00 [0.00-9.46]	**<.001**	5.41 [0.00-26.5]	**<.001**	0.00 [0.00-0.31]	**<.001**
Asian	36.9 [0.00-191]	0.45	8.83 [0.00-45.2]	0.65	26.6 [0.00-138]	0.33	0.06 [0.00-2.25]	0.57	30.7 [0.00-43.9]	0.23	5.01 [0.00-15.7]	**0.04**	17.8 [0.00-30.7]	0.19	0.05 [0.00-0.60]	0.54
Ethnicity																
Hispanic or Latino	23.4 [0.00-140]	0.06	3.55 [0.00-44.2]	**0.04**	13.2 [0.00-88.5]	0.09	0.00 [0.00-1.47]	**0.04**	24.2 [0.00-40.1]	0.06	3.02 [0.00-13.9]	**0.03**	10.2 [0.00-28.9]	0.13	0.00 [0.00-0.33]	**0.03**
**Cardiac Risk Factors**																
BMI (kg/m^2)		**0.03**		0.13		**.001**		**<.001**		0.07		**0.01**		**.004**		**<.001**
<18.5	28.4 [0.00-113]		9.31 [0.00-47.4]		15.7 [0.00-62.8]		0.00 [0.00-0.67]		25.4 [0.00-52.4]		8.35 [0.00-16.5]		8.09 [0.00-39.9]		0.00 [0.00-0.41]	
18.5 – 24.9	28.1 [0.00-135]		5.30 [0.00-47.5]		12.7 [0.00-75.6]		0.00 [0.00-0.63]		25.2 [0.00-43.1]		4.76 [0.00-17.0]		8.95 [0.00-26.3]		0.00 [0.00-0.16]	
25.0 – 29.9	46.0 [0.00-184]		8.14 [0.00-58.0]		27.5 [0.00-115]		0.06 [0.00-2.28]		28.7 [0.00-43.9]		5.42 [0.00-15.4]		15.7 [0.00-29.9]		0.03 [0.00-0.58]	
≥30.0	38.9 [0.00-163]		5.75 [0.00-43.9]		23.3 [0.00-104]		0.06 [0.00-2.67]		26.5 [0.00-41.2]		3.83 [0.00-13.2]		14.7 [0.00-29.1]		0.03 [0.00-0.64]	
Hypertension		**<.001**		**<.001**		**<.001**		**<.001**		**<.001**		**<.001**		**.004**		**<.001**
Yes	49.3 [0.00-188]		9.55 [0.00-58.3]		29.4 [0.00-114]		0.06 [0.00-2.53]		28.2 [0.00-43.5]		5.38 [0.00-15.6]		15.2 [0.00-29.3]		0.04 [0.00-0.58]	
No	26.2 [0.00-131]		3.22 [0.00-34.1]		14.8 [0.00-87.2]		0.00 [0.00-1.38]		24.3 [0.00-41.3]		2.81 [0.00-13.1]		12.0 [0.00-28.6]		0.00 [0.00-0.41]	
Dyslipidemia		**<.001**		**<.001**		**<.001**		**0.02**		**.003**		**<.001**		**0.02**		0.06
Yes	46.2 [0.00-184]		8.64 [0.00-57.5]		26.6 [0.00-109]		0.06 [0.00-2.09]		28.2 [0.00-42.9]		5.47 [0.00-15.4]		14.9 [0.00-29.0]		0.03 [0.00-0.53]	
No	29.4 [0.00-136]		2.94 [0.00-33.8]		16.6 [0.00-93.2]		0.00 [0.00-1.88]		24.3 [0.00-42.1]		2.18 [0.00-12.9]		12.3 [0.00-29.2]		0.00 [0.00-0.49]	
Smoking (ever)		**<.001**		**<.001**		**<.001**		**<.001**		**<.001**		**<.001**		**<.001**		**<.001**
Yes	59.1 [0.00-205]		12.5 [0.00-64.3]		36.6 [0.00-130]		0.13 [0.00-2.84]		30.8 [0.00-44.4]		6.68 [0.00-16.1]		17.1 [0.00-29.9]		0.06 [0.00-0.42]	
No	22.4 [0.00-121]		2.53 [0.00-33.4]		12.2 [0.00-77.0]		0.00 [0.00-1.38]		23.2 [0.00-44.4]		2.19 [0.00-12.8]		10.0 [0.00-28.0]		0.00 [0.00-0.42]	
Family history of premature CAD		**.004**		**.002**		**.007**		**0.04**		**<.001**		**<.001**		**<.001**		0.07
Yes	49.1 [0.00-183]		10.5 [0.00-56.4]		27.7 [0.00-113]		0.06 [0.00-2.27]		29.3 [0.00-44.1]		6.19 [0.00-15.4]		15.6 [0.00-29.9]		0.03 [0.00-0.57]	
No	35.7 [0.00-158]		5.22 [0.00-47.3]		20.6 [0.00-97.5]		0.03 [0.00-1.91]		26.1 [0.00-41.6]		3.68 [0.00-14.4]		13.4 [0.00-28.6]		0.01 [0.00-0.49]	
Peripheral artery disease		**<.001**		**<.001**		**.002**		0.11		**<.001**		**<.001**		**0.05**		0.29
Yes	84.3 [0.00-256]		22.3 [0.00-78.4]		39.6 [0.00-144]		0.13 [0.00-2.53]		31.9 [0.00-47.3]		10.6 [0.00-19.2]		18.0 [0.00-29.6]		0.05 [0.00-0.57]	
No	38.3 [0.00-163]		6.25 [0.00-48.2]		21.8 [0.00-99.1]		0.03 [0.00-2.03]		26.8 [0.00-42.3]		4.20 [0.00-14.7]		13.9 [0.00-29.0]		0.01 [0.00-0.52]	
Diabetes		**<.001**		**<.001**		**<.001**		**<.001**		**<.001**		**<.001**		**<.001**		**<.001**
Yes	68.1 [0.00-221]		13.6 [0.00-67.1]		39.5 [0.00-138]		0.28 [0.00-3.56]		31.9 [0.00-45.5]		7.60 [0.00-17.1]		18.9 [0.00-30.7]		0.10 [0.00-0.77]	
No	32.8 [0.00-149]		3.69 [0.00-36.1]		18.9 [0.00-94.7]		0.00 [0.00-1.75]		25.7 [0.00-41.5]		3.70 [0.00-14.2]		12.9 [0.00-28.5]		0.00 [0.00-0.46]	
**Medication Use**																
Aspirin		**<.001**		**<.001**		**<.001**		**<.001**		**<.001**		**<.001**		**<.001**		**<.001**
Yes	58.1 [0.00-208]		13.6 [0.00-67.1]		35.0 [0.00-124]		0.13 [0.00-2.75]		30.4 [0.00-44.1]		7.41 [0.00-16.5]		16.3 [0.00-29.2]		0.06 [0.00-0.60]	
No	27.9 [0.00-137]		3.69 [0.00-36.1]		16.6 [0.00-89.0]		0.00 [0.00-1.75]		25.2 [0.00-41.5]		2.91 [0.00-13.4]		12.5 [0.00-29.0]		0.00 [0.00-0.46]	
Statin		**<.001**		**<.001**		**<.001**		**<.001**		**<.001**		**<.001**		**<.001**		**<.001**
Yes	58.6 [0.00-204]		13.4 [0.00-69.6]		33.0 [0.00-128]		0.13 [0.00-2.59]		30.4 [0.00-44.2]		7.41 [0.00-16.6]		16.4 [0.00-29.4]		0.06 [0.00-0.58]	
No	29.1 [0.00-136]		3.41 [0.00-36.1]		16.4 [0.00-89.6]		0.00 [0.00-1.78]		24.7 [0.00-41.2]		2.59 [0.00-13.4]		11.7 [0.00-28.9]		0.00 [0.00-0.48]	
Beta-blocker		0.18		0.06		0.32		0.16		0.68		0.07		0.97		0.2
Yes	45.8 [0.00-180]		10.9 [0.00-56.5]		23.0 [0.00-105]		0.06 [0.00-2.53]		27.7 [0.00-41.7]		5.93 [0.00-15.6]		14.3 [0.00-28.5]		0.03 [0.00-0.56]	
No	39.8 [0.00-168]		6.61 [0.00-49.9]		23.6 [0.00-103]		0.03 [0.00-1.97]		27.4 [0.00-43.0]		4.37 [0.00-14.8]		14.4 [0.00-29.2]		0.01 [0.00-0.52]	
**ASCVD risk**																
**ASCVD risk (2013)**		**<.001**		**<.001**				**<.001**		**<.001**		**<.001**		**<.001**		**<.001**
<5%	0.00 [0.00-35.5]		0.00 [0.00-5.50]		0.00 [0.00-21.8]		0.00 [0.00-0.63]		0.00 [0.00-30.5]		0.00 [0.00-5.80]		0.00 [0.00-19.5]		0.00 [0.00-0.03]	
5-to <7.5%	8.36 [0.00-71.0]		0.00 [0.00-15.8]		3.41 [0.00-51.1]		0.00 [0.00-0.69]		15.4 [0.00-36.3]		0.00 [0.00-10.2]		4.20 [0.00-26.0]		0.00 [0.00-0.31]	
7.5 to <20%	52.5 [0.00-177]		9.73 [0.00-50.8]		32.4 [0.00-115]		0.13 [0.00-2.81]		30.3 [0.00-44.1]		5.47 [0.00-14.9]		17.1 [0.00-30.6]		0.06 [0.00-0.72]	
≥20%	125 [30.3-329]		42.3 [5.06-125]		71.8 [14.1-199]		0.59 [0.00-4.41]		36.2 [22.5-47.0]		11.9 [2.98-20.2]		20.7 [7.20-31.8]		0.14 [0.00-0.71]	

ASCVD = atherosclerotic cardiovascular disease; BMI = body mass index; CAD = coronary artery disease.

© 2026 Karády J et al. *JAMA Cardiology*.

eTable 4. Median PV measures and qualitative CT measures.

	Plaque volume (mm^3)								Plaque burden (%)							
**All patients (n=4,267)**	**Total**	**p**	**Calcified**	**p**	**Non-calcified**	**p**	**Low attenuation**	**p**	**Total**	**p**	**Calcified**	**p**	**Non-calcified**	**p**	**Low attenuation**	**p**
**CAC score**		**<.001**		**<.001**		**<.001**		**<.001**		**<.001**		**<.001**		**<.001**		**<.001**
CAC = 0	0.00 [0.00-0.00]		0.00 [0.00-0.00]		0.00 [0.00-0.00]		0.00 [0.00-0.00]		0.00 [0.00-0.00]		0.00 [0.00-0.00]		0.00 [0.00-0.00]		0.00 [0.00-0.00]	
CAC 1-100	54.0 [26.1-111]		11.8 [4.91-22.9]		39.3 [14.8-90.0]		0.38 [0.00-2.78]		34.4 [25.4-43.6]		7.57 [3.37-13.6]		25.2 [14.8-35.3]		0.21 [0.00-1.18]	
CAC 101-400	177 [103-274]		62.1 [42.2-93.5]		98.2 [46.2-187]		1.12 [0.16-5.44]		39.3 [29.3-48.8]		14.5 [9.77-20.2]		22.8 [13.9-32.3]		0.26 [0.05-1.01]	
CAC >400	435 [265-715]		197 [126-308]		223 [94.8-419]		3.53 [0.60-11.4]		45.2 [36.3-54.3]		20.8 [15.4-27.4]		23.0 [14.1-31.6]		0.35 [0.07-0.92]	
**Stenosis (%)**		**<.001**		**<.001**		**<.001**		**<.001**		**<.001**		**<.001**		**<.001**		**<.001**
No plaque	0.00 [0.00-0.00]		0.00 [0.00-0.00]		0.00 [0.00-0.00]		0.00 [0.00-0.00]		0.00 [0.00-0.00]		0.00 [0.00-0.00]		0.00 [0.00-0.00]		0.00 [0.00-0.00]	
Mild plaque (stenosis 1–49%)	87.0 [35.9-192]		22.3 [6.09-64.1]		52.7 [20.4-124]		0.59 [0.03-3.53]		35.6 [26.3-44.7]		10.7 [4.42-17.9]		23.2 [16.9-32.4]		0.21 [0.01-0.97]	
Moderate plaque (stenosis 50–69%)	271 [136-448]		87.1 [31.2-177]		166 [73.1-290]		2.63 [0.38-10.3]		46.5 [38.6-55.0]		15.6 [8.18-23.8]		30.1 [20.9-39.2]		0.40 [0.09-1.68]	
Severe plaque (stenosis ≥70% or ≥50% LM)	362 [194-704]		109 [43.2-214]		235 [111-462]		5.77 [1.44-18.4]		51.2 [43.0-61.1]		15.4 [7.61-22.9]		34.8 [23.0-45.0]		0.69 [0.25-2.49]	
Plaque stenosis ≥50%	302 [159-563]	**<.001**	96.9 [31.8-197]	**<.001**	195 [88.4-360]	**<.001**	3.91 [0.69-12.4]	**<.001**	48.7 [39.8-58.2]	**<.001**	15.6 [8.06-23.5]	**<.001**	31.7 [22.0-41.6]	**<.001**	0.55 [0.15-1.96]	**<.001**
**Any Plaque**	114 [45-268]	**<.001**	29.6 [7.97-93.1]	**<.001**	71.0 [26.2-172]	**<.001**	0.91 [0.06-5.09]	**<.001**	38.4 [28.2-47.9]	**<.001**	11.6 [5.03-19.1]	**<.001**	24.9 [15.2-34.8]	**<.001**	0.27 [0.03-1.13]	**<.001**
**High risk plaque features**																
Low HU plaque	270 [121-477]	**<.001**	52.7 [11.0-138]	**<.001**	202 [88.2-344]	**<.001**	6.97 [1.84-16.0]	**<.001**	47.0 [36.9-56.1]	**<.001**	10.3 [3.58-16.8]	**<.001**	34.6 [25.9-43.9]	**<.001**	1.07 [0.31-3.34]	**<.001**
Napkin-Ring Sign	314 [154-564]	**<.001**	61.5 [15.9-181]	**<.001**	216 [109-386]	**<.001**	7.13 [2.03-18.5]	**<.001**	49.8 [40.7-57.6]	**<.001**	10.7 [4.77-17.7]	**<.001**	36.4 [26.2-45.5]	**<.001**	0.93 [0.34-2.78]	**<.001**
Positive Remodeling	204 [101-404]	**<.001**	47.8 [13.6-122]	**<.001**	144 [64.2-287]	**<.001**	3.92 [0.59-11.7]	**<.001**	45.4 [35.3-54.1]	**<.001**	11.1 [4.80-18.4]	**<.001**	32.2 [22.3-42.4]	**<.001**	0.72 [0.17-2.18]	**<.001**
Low HU + Positive Remodeling	303 [137-529]	**<.001**	52.8 [11.0-149]	**<.001**	217 [96.6-373]	**<.001**	8.25 [2.88-18.8]	**<.001**	49.5 [40.8-58.2]	**<.001**	9.94 [3.49-16.4]	**<.001**	38.1 [28.3-45.1]	**<.001**	1.38 [0.41-3.44]	**<.001**
Any HRP feature	200 [97.6-398]	**<.001**	47.8 [13.6-121]	**<.001**	142 [62.4-280]	**<.001**	3.84 [0.56-11.6]	**<.001**	44.7 [35.0-53.6]	**<.001**	11.1 [4.77-18.4]	**<.001**	31.5 [22.0-41.4]	**<.001**	0.69 [0.16-2.11]	**<.001**
**Leaman score**		**<.001**		**<.001**		**<.001**		**<.001**		**<.001**		**<.001**		**<.001**		**<.001**
0	0.00 [0.00-0.00]		0.00 [0.00-0.00]		0.00 [0.00-0.00]		0.00 [0.00-0.00]		0.00 [0.00-0.00]		0.00 [0.00-0.00]		0.00 [0.00-0.00]		0.00 [0.00-0.00]	
1–5	39.0 [18.5-76.5]		7.81 [2.06-21.6]		27.2 [9.84-52.7]		0.13 [0.00-1.41]		35.0 [25.3-44.7]		9.10 [2.60-16.8]		23.4 [13.4-35.2]		0.12 [0.00-0.89]	
>5	184 [88.2-362]		55.6 [18.9-133]		111 [49.3-225]		1.80 [0.25-7.64]		39.9 [30.6-49.5]		12.9 [6.36-20.2]		25.5 [16.3-34.7]		0.35 [0.07-1.25]	

CAC = coronary artery calcium; HRP = high-risk plaque; HU = Hounsfield unit; LM = left main

© 2026 Karády J et al. *JAMA Cardiology*.

eTable 5. Multivariable assessment of quantitative plaque volume/burden quartiles and MACE.

	**Univariable**			**Multivariable Model 1***			**Multivariable Model 2†**		
All patients (n=4,267)	**HR**	**95% CI**	**p**	**HR**	**95% CI**	**p**	**HR**	**95% CI**	**p**
**TPV**									
1st Quartile	Ref	–	–	Ref	–	–	Ref	–	–
2nd Quartile	2.50	1.27–4.92	**.008**	2.23	1.12–4.44	**0.02**	2.20	0.99–4.91	0.05
3rd Quartile	3.96	2.12–7.40	**<.001**	3.42	1.79–6.52	**<.001**	3.21	1.50–6.86	**.003**
4th Quartile	6.48	3.60–11.69	**<.001**	4.99	2.63–9.46	**<.001**	3.23	1.41–7.40	**.006**
**CPV**									
1st Quartile	Ref	–	–	Ref	–	–	Ref	–	–
2nd Quartile	2.35	1.25–4.43	**.008**	1.96	1.02–3.78	**0.04**	2.04	0.97–4.32	0.06
3rd Quartile	3.92	2.20–6.99	**<.001**	3.44	1.89–6.23	**<.001**	2.94	1.45–5.97	**.003**
4th Quartile	4.96	2.84–8.65	**<.001**	3.62	1.96–6.69	**<.001**	2.28	1.04–4.98	**0.04**
**NCPV**									
1st Quartile	Ref	–	–	Ref	–	–	Ref	–	–
2nd Quartile	2.02	1.00–4.11	0.05	1.74	0.84–3.60	0.14	1.87	0.82–4.27	0.14
3rd Quartile	4.59	2.49–8.48	**<.001**	4.02	2.14–7.55	**<.001**	3.59	1.70–7.60	**.001**
4th Quartile	6.39	3.54–11.52	**<.001**	4.86	2.57–9.20	**<.001**	3.28	1.45–7.41	**.004**
**LAPV**									
1st Quartile	Ref	–	–	Ref	–	–	Ref	–	–
2nd Quartile	3.60	2.09–6.20	**<.001**	3.15	1.80–5.52	**<.001**	2.57	1.38–4.79	**.003**
3rd Quartile	3.39	1.95–5.88	**<.001**	2.70	1.51–4.81	**.001**	2.01	1.04–3.87	**0.04**
4th Quartile	5.09	3.05–8.50	**<.001**	4.02	2.32–6.97	**<.001**	2.37	1.22–4.60	**0.01**
**TPB**									
1st Quartile	Ref	–	–	Ref	–	–	Ref	–	–
2nd Quartile	2.35	1.18–4.64	**0.01**	1.99	0.99–4.00	0.05	2.16	0.98–4.79	**0.06**
3rd Quartile	4.96	2.71–9.11	**<.001**	3.94	2.10–7.39	**<.001**	3.39	1.60–7.17	**.001**
4th Quartile	5.67	3.12–10.32	**<.001**	4.15	2.21–7.79	**<.001**	2.83	1.28–6.29	**0.01**
**CPB**									
1st Quartile	Ref	–	–	Ref	–	–	Ref	–	–
2nd Quartile	3.88	2.18–6.90	**<.001**	3.04	1.66–5.55	**<.001**	2.79	1.37–5.68	**.005**
3rd Quartile	3.65	2.04–6.53	**<.001**	2.84	1.54–5.22	**.001**	2.43	1.18–5.00	**0.02**
4th Quartile	3.69	2.06–6.61	**<.001**	2.82	1.52–5.24	**.001**	1.98	0.93–4.25	**0.08**
**NCPB**									
1st Quartile	Ref	–	–	Ref	–	–	Ref	–	–
2nd Quartile	2.96	1.54–5.70	**.001**	2.41	1.23–4.73	**0.01**	2.47	1.13–5.37	**0.02**
3rd Quartile	4.15	2.23–7.74	**<.001**	3.26	1.70–6.24	**<.001**	2.84	1.32–6.12	**.008**
4th Quartile	5.86	3.23–10.63	**<.001**	4.33	2.33–8.06	**<.001**	3.09	1.43–6.69	**.004**
**LAPB**									
1st Quartile	Ref	–	–	Ref	–	–	Ref	–	–
2nd Quartile	3.87	2.26–6.61	**<.001**	3.33	1.91–6.22	**<.001**	2.75	1.49–5.10	**.001**
3rd Quartile	4.80	2.87–8.04	**<.001**	3.59	2.07–6.22	**<.001**	2.34	1.23–4.43	**.009**
4th Quartile	3.35	1.92–5.84	**<.001**	2.83	1.58–5.04	**<.001**	1.79	0.91–3.53	**0.09**

© 2026 Karády J et al. *JAMA Cardiology*.

***Model 1** is adjusted for age, sex, race, ASCVD risk, and statin use

† **Model 2** is adjusted for components of **Model 1** plus continuous CAC, stenosis ≥50%, and high-risk plaque features.

NCPB = non-calcified plaque burden; TPB = total plaque burden; TPV = total plaque volume

© 2026 Karády J et al. *JAMA Cardiology*.

eTable 6. Multivariable assessment of quantitative plaque volume/burden and MACE

	Multivariable Model 3*			Multivariable Model 4†			Multivariable Model 5‡		
All patients (n=4,267)	**HR**	**95% CI**	**p**	**HR**	**95% CI**	**p**			
**Plaque volume (per 100 mm³)**									
Total	1.06	0.99-1.13	0.16	1.03	0.98-1.08	0.32	1.03	0.98 - 1.08	0.22
Calcified	1.02	0.89-1.17	0.76	1.05	0.96-1.15	0.25	1.05	0.96-1.14	0.31
Non-calcified	1.09	1.01-1.17	**0.02**	1.03	0.94-1.12	0.51	1.05	0.97-1.13	0.27
Low attenuation	2.86	1.60-5.14	**<.001**	1.87	0.92-3.78	0.08	2.01	1.03-3.91	**0.04**
**Plaque burden (per 10%)**									
Total	1.33	1.19-1.47	**<.001**	1.18	1.06-1.31	**.003**	1.20	1.08-1.34	**.001**
Calcified	1.26	1.02-1.54	**0.03**	1.11	0.92-1.33	0.28	1.10	0.91-1.33	0.31
Non-calcified	1.35	1.21-1.52	**<.001**	1.19	1.05-1.34	**.006**	1.22	1.09-1.37	**.001**
Low attenuation	2.45	1.37-4.36	**.002**	1.47	0.74-2.92	0.27	1.66	0.87-3.15	0.12

(eTable 4. continued)

	Multivariable Model 6§		
All patients (n=4,267)	**HR**	**95% CI**	**p**
**Plaque volume (per 100 mm³)**			
Total	1.06	1.02-1.10	**.006**
Calcified	1.10	1.03-1.19	**.008**
Non-calcified	1.08	1.01-1.16	**0.03**
Low attenuation	2.48	1.30-4.73	**.006**
**Plaque burden (per 10%)**			
Total	1.25	1.13-1.38	**<.001**
Calcified	1.23	1.03-1.46	**0.02**
Non-calcified	1.25	1.12-1.41	**<.001**
Low attenuation	1.65	0.87-3.12	0.13

(Models 1 and 2 are displayed in the main results.
Model 1 is adjusted for age, sex, race, ASCVD risk, and statin use.
Model 2 is adjusted for components of Model 1 plus continuous CAC, stenosis ≥50%, high-risk plaque features).
* Model 3 is adjusted for components of Model 1 plus continuous CAC
†Model 4 is adjusted for components of Model 1 plus stenosis ≥50%, high-risk plaque features
‡ Model 5 is adjusted for components of Model 1 plus stenosis ≥50%
§ Model 6 is adjusted for components of Model 1 plus high-risk plaque features

© 2026 Karády J et al. *JAMA Cardiology*.

eTable 7. Univariable and multivariable assessment of quantitative plaque volume/burden and **non-fatal MI and cardiovascular death**.

	Univariable			Multivariable Model 1*			Multivariable Model 2†		
All patients (n=4,267)	**HR**	**95% CI**	**p**	**HR**	**95% CI**	**p**	**HR**	**95% CI**	**p**
**Plaque volume (per 100 mm^3)**									
Total	1.06	1.00-1.13	**0.04**	1.02	0.94-1.11	0.59	0.93	0.81-1.07	0.31
Calcified	1.10	0.97-1.24	0.13	1.03	0.85-1.23	0.79	0.83	0.60-1.17	0.29
Non-calcified	1.10	1.00-1.21	**0.05**	1.04	0.92-1.18	0.53	0.93	0.78-1.11	0.43
Low attenuation	1.82	1.04-3.18	**0.04**	2.45	0.99-6.03	0.05	1.61	0.56-4.64	0.38
**Plaque burden (per 10%)**									
Total	1.29	1.14-1.47	**<.001**	1.22	1.06-1.40	**.005**	1.14	0.95-1.37	0.17
Calcified	1.31	1.03-1.66	**0.03**	1.15	0.88-1.51	0.30	1.04	0.73-1.49	0.83
Non-calcified	1.32	1.14-1.53	**<.001**	1.26	1.08-1.48	**.004**	1.17	0.95-1.44	0.14
Low attenuation	2.05	0.95-4.40	0.07	2.17	0.93-5.10	0.07	1.79	0.59-5.38	0.30

(eTable 5. continued)

	Multivariable Model 3‡			Multivariable Model 4§			Multivariable Model 5||n		
All patients (n=4,267)	**HR**	**95% CI**	**p**	**HR**	**95% CI**	**p**	**HR**	**95% CI**	**p**
**Plaque volume (per 100 mm^3)**									
Total	1.00	0.89-1.12	0.99	0.96	0.85-1.07	0.44	0.97	0.87-1.08	0.59
Calcified	0.92	0.71-1.19	0.52	0.93	0.72-1.20	0.58	0.94	0.73-1.20	0.61
Non-calcified	1.03	0.89-1.18	0.71	0.94	0.79-1.11	0.45	0.97	0.83-1.12	0.66
Low attenuation	2.40	0.98-5.91	0.06	1.66	0.57-4.80	0.35	1.80	0.65-4.94	0.26
**Plaque burden (per 10%)**									
Total	1.24	1.06-1.46	**.008**	1.13	0.96-1.33	0.13	1.16	0.99 -1.35	0.06
Calcified	1.16	0.82-1.62	0.40	1.03	0.77-1.38	0.84	1.03	0.77-1.38	0.82
Non-calcified	1.28	1.07-1.53	**.006**	1.17	0.97-1.40	0.10	1.20	1.01-1.43	**0.04**
Low attenuation	2.61	1.04-6.56	**0.04**	1.60	0.58-4.39	0.36	1.81	0.70-4.67	0.22

(eTable 5. continued)

	Multivariable Model 6¶		
All patients (n=4,267)	**HR**	**95% CI**	**p**
**Plaque volume (per 100 mm^3)**			
Total	0.99	0.90-1.10	0.90
Calcified	0.99	0.80-1.24	0.95
Non-calcified	0.99	0.85-1.15	0.88
Low attenuation	2.01	0.74-5.45	0.17
**Plaque burden (per 10%)**			
Total	1.17	1.01-1.36	**0.04**
Calcified	1.10	0.83-1.46	0.50
Non-calcified	1.20	1.01-1.43	**0.04**
Low attenuation	1.73	0.66-4.54	0.26

© 2026 Karády J et al. *JAMA Cardiology*.

*Model 1 is adjusted for age, sex, race, ASCVD risk, and statin use
† Model 2 is adjusted for components of Model 1 plus continuous CAC, stenosis ≥50%, high-risk plaque features
‡ Model 3 is adjusted for components of Model 1 plus continuous CAC
§ Model 4 is adjusted for components of Model 1 plus stenosis ≥50%, high-risk plaque features
|| Model 5 is adjusted for components of Model 1 plus stenosis ≥50%
¶ Model 6 is adjusted for components of Model 1 plus high-risk plaque features

© 2026 Karády J et al. *JAMA Cardiology*.

eTable 8. Crude MACE Rates (All-cause death, MI, UAP) stratified by plaque volume/Burden quartiles and CAC categories among all patients (n=4,267).

	CAC 0	CAC 1-100	CAC 101-400	CAC >400
**Total Plaque volume**				
1st Quartile (n=1299)	0.8 (10/1299)	---	---	---
2nd Quartile (n=805)	2.6 (3/114)	1.7 (10/602)	5.4 (4/74)	0.0 (0/15)
3rd Quartile (n=824)	7.7 (4/52)	3.2 (13/405)	3.6 (11/304)	4.8 (3/63)
4th Quartile (n=796)	9.1 (1/11)	2.9 (3/104)	6.6 (18/271)	6.3 (26/410)
**Calcified Plaque volume**				
1st Quartile (n=1369)	0.9 (12/1351)	0.0 (0/18)	---	---
2nd Quartile (n=782)	5.0 (6/119)	1.9 (12/629)	7.1 (2/28)	0.0 (0/6)
3rd Quartile (n=803)	0.0 (0/4)	3.1 (14/453)	5.1 (16/312)	5.9 (2/34)
4th Quartile (n=770)	0.0 (0/2)	0.0 (0/11)	4.8 (15/309)	6.0 (27/448)
**Non-calcified Plaque volume**				
1st Quartile (n=1301)	0.8 (10/1299)	0.0 (0/1)	0.0 (0/1)	---
2nd Quartile (n=806)	2.5 (2/81)	1.5 (8/540)	3.7 (5/134)	0.0 (0/51)
3rd Quartile (n=811)	2.6 (2/76)	3.4 (13/382)	4.0 (10/252)	7.9 (8/101)
4th Quartile (n=806)	20.0 (4/20)	2.7 (5/188)	6.9 (18/262)	6.2 (21/336)
**Low attenuation Plaque volume**				
1st Quartile (n=1772)	0.8 (10/1337)	2.6 (8/311)	1.2 (1/84)	0.0 (0/40)
2nd Quartile (n=660)	6.8 (3/44)	2.4 (8/331)	4.1 (8/194)	7.7 (7/91)
3rd Quartile (n=634)	2.3 (1/44)	2.7 (7/261)	4.8 (9/186)	5.6 (8/143)
4th Quartile (n=658)	7.8 (4/51)	1.4 (3/208)	8.1 (15/185)	6.5 (14/214)
**Total Plaque burden**				
1st Quartile (n=1299)	0.8 (10/1299)	---	---	---
2nd Quartile (n=820)	1.7 (1/59)	1.5 (7/475)	3.4 (7/205)	3.7 (3/81)
3rd Quartile (n=818)	6.5 (4/62)	2.9 (11/379)	3.6 (8/221)	7.7 (12/156)
4th Quartile (n=787)	5.4 (3/56)	3.1 (8/257)	8.1 (18/223)	5.6 (14/251)
**Calcified Plaque burden**				
1st Quartile (n=1369)	0.9 (12/1351)	0.0 (0/18)	---	---
2nd Quartile (n=786)	5.4 (6/111)	2.7 (15/552)	10.1 (10/99)	4.2 (1/24)
3rd Quartile (n=791)	0.0 (0/10)	2.0 (7/357)	5.0 (15/301)	6.5 (8/123)
4th Quartile (n=778)	0.0 (0/4)	2.2 (4/184)	3.2 (8/249)	5.9 (20/341)
**Non-calcified Plaque burden**				
1st Quartile (n=1301)	0.8 (10/1299)	0.0 (0/1)	0.0 (0/1)	---
2nd Quartile (n=815)	0.0 (0/20)	1.6 (6/384)	2.1 (5/235)	6.8 (12/176)
3rd Quartile (n=810)	2.2 (1/45)	2.0 (7/343)	5.5 (13/235)	5.3 (10/187)
4th Quartile (n=798)	6.2 (7/112)	3.4 (13/383)	8.4 (15/178)	5.6 (7/125)
**Low attenuation Plaque burden**				
1st Quartile (n=1772)	0.8 (10/1337)	2.6 (8/311)	1.2 (1/84)	0.0 (0/40)
2nd Quartile (n=666)	8.3 (2/24)	3.1 (8/256)	4.0 (9/225)	6.8 (11/161)
3rd Quartile (n=639)	5.4 (2/37)	2.1 (5/242)	7.7 (14/181)	6.7 (12/179)
4th Quartile (n=647)	5.1 (4/78)	1.7 (5/302)	5.7 (9/159)	5.6 (6/108)

© 2026 Karády J et al. *JAMA Cardiology*.

eTable 9. Cox regression hazard ratios (All-cause death, MI, UAP) for continuous plaque volume and plaque burden across CAC categories

	CAC 0 (n=1476)			CAC 1-100 (n=1111)			CAC 101-400 (n=649)			CAC >400 (n=488)		
All patients	**HR**	**95% CI**	**p**	**HR**	**95% CI**	**p**	**HR**	**95% CI**	**p**	**HR**	**95% CI**	**p**
**Plaque Volume (per 100 mm^3)**												
Total	1.37	1.11-1.69	.003	1.22	0.91-1.62	0.18	1.25	1.10-1.42	<.001	1.03	0.96-1.09	0.42
Calcified	0.90	0.03-25.2	0.95	3.06	0.52-18.0	0.22	0.83	0.37-1.85	0.65	1.07	0.98-1.18	0.12
Non-calcified	1.73	1.31-2.29	<.001	1.21	0.89-1.64	0.23	1.34	1.17-1.53	<.001	1.01	0.90-1.12	0.92
Low attenuation	4.21	0.89-20.0	0.07	1.12	0.04-29.7	0.95	4.50	2.51-8.04	<.001	1.50	0.60-3.73	0.38
**Plaque Burden (per 10%)**												
Total	1.45	1.20-1.76	<.001	1.32	1.00-1.73	0.05	1.42	1.10-1.83	.006	1.15	0.87-1.51	0.33
Calcified	0.94	0.05-18.4	0.97	0.98	0.58-1.65	0.94	0.66	0.40-1.09	0.11	1.20	0.82-1.75	0.35
Non-calcified	1.48	1.22-1.80	<.001	1.29	1.00-1.67	0.05	1.56	1.23-1.97	<.001	1.05	0.79-1.39	0.73
Low attenuation	3.40	1.41-8.26	.007	0.40	0.04-4.31	0.45	5.00	2.54-9.80	<.001	2.05	0.24-17.4	0.51

© 2026 Karády J et al. *JAMA Cardiology*.

eTable 10. Univariable and multivariable assessment of quantitative plaque volume/burden data-driven thresholds and **MACE**.

	**Multivariable Model 3‡**			**Multivariable Model 4§**			**Multivariable Model 5||n**		
All patients (n=4,267)	**HR**	**95% CI**	**p**	**HR**	**95% CI**	**p**	**HR**	**95% CI**	**p**
TPV ≥ 87.2 mm^3	3.11	1.94-4.96	**<.001**	1.94	1.22 3.09	**.005**	2.11	1.34-3.33	**.001**
TPB ≥ 35.3%	2.87	1.86-4.44	**<.001**	1.91	1.23-2.96	**.004**	2.05	1.33-3.16	**.001**
NCPB ≥ 19.7%	2.52	1.64-3.85	**<.001**	1.76	1.15-2.71	**.009**	1.91	1.26-2.89	**.002**

(eTable 6. continued)

	**Multivariable Model 6¶**		
All patients (n=4,267)	**HR**	**95% CI**	**p**
TPV ≥ 87.2 mm^3	2.38	1.52-3.70	**<.001**
TPB ≥ 35.3%	2.32	1.53-3.51	**<.001**
NCPB ≥19.7%	2.05	1.35-3.02	**.001**

‡ Model 3 is adjusted for components of Model 1 plus continuous CAC
§ Model 4 is adjusted for components of Model 1 plus stenosis ≥50% and high-risk plaque features
|| Model 5 is adjusted for components of Model 1 plus stenosis ≥50%
¶ Model 6 is adjusted for components of Model 1 plus high-risk plaque features
MACE = major adverse cardiac events defined as hospitalization for unstable angina pectoris, non-fatal myocardial infarction, all-cause death. NCPB = non-calcified plaque burden; TPB = total plaque burden; TPV = total plaque volume.

© 2026 Karády J et al. *JAMA Cardiology*.

eFigure. Consort diagram.

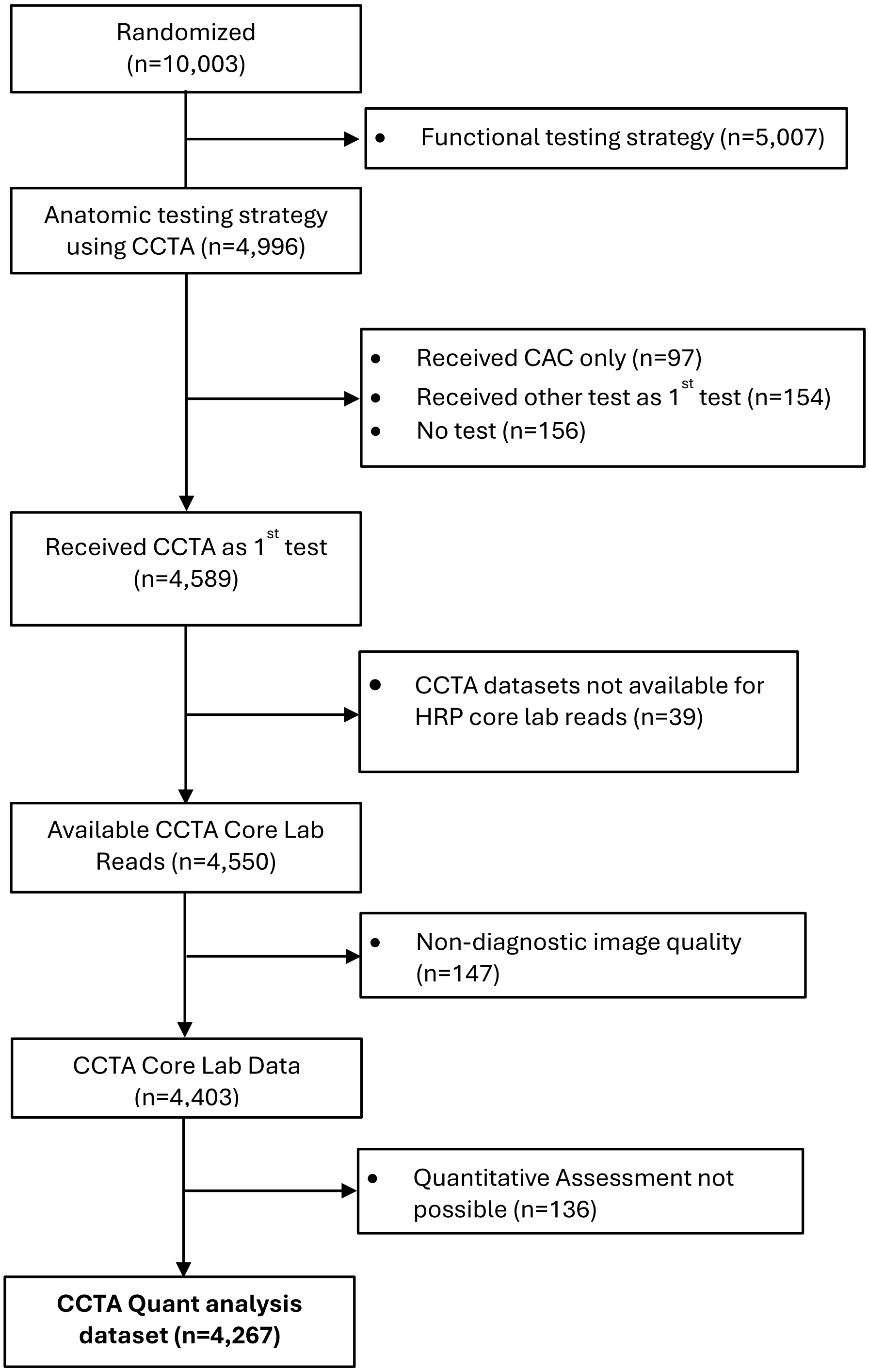

CAC= coronary artery calcium; CCTA= coronary computed tomography angiography.

© 2026 Karády J et al. *JAMA Cardiology*.
