# Supplementary material for: Prognostic Value of Plaque Volume in Patients With First Diagnosis of Coronary Artery Disease: A Substudy of the PROMISE Randomized Clinical Trial
Source: JAMA Cardiol. 2026 Feb 11;11(3):259–67. doi: 10.1001/jamacardio.2025.5520 (PMC12895320; doi:10.1001/jamacardio.2025.5520)
Supplement: Supplement 3. — Data sharing statement [file jamacardiol-e255520-s003.pdf]

Data Sharing Statement

Karády. Prognostic Value of Plaque Volume in Patients With First Diagnosis of Coronary Artery Disease. *JAMA Cardiol*. Published February 11, 2026. doi:10.1001/jamacardio.2025.5520

Data
Additional Information: ClinicalTrials.gov: NCT01174550
Data available: No
